# Supplementary figures and images for: Active RNAP pre-initiation sites are highly mutated by cytidine deaminases in yeast, with AID targeting small RNA genes
Source: eLife. 2014 Sep 19;3:e03553. doi: 10.7554/eLife.03553 (PMC4359381; doi:10.7554/eLife.03553)

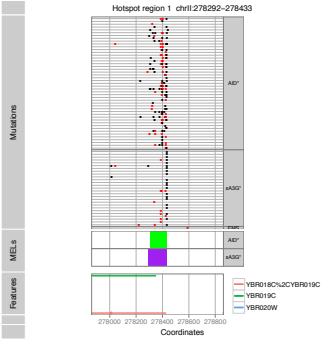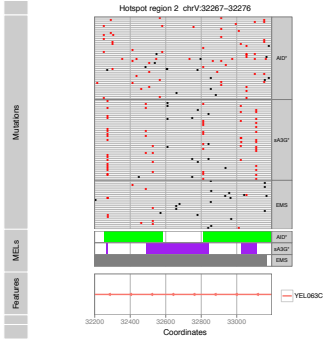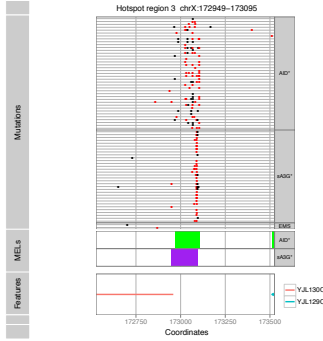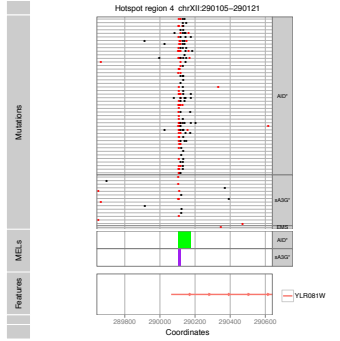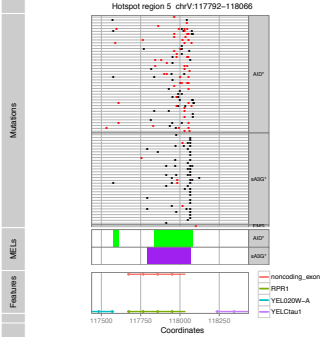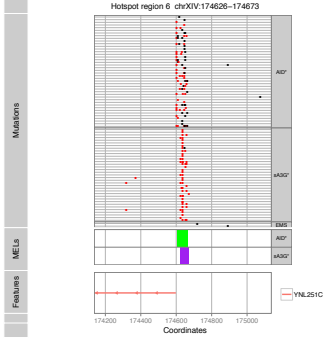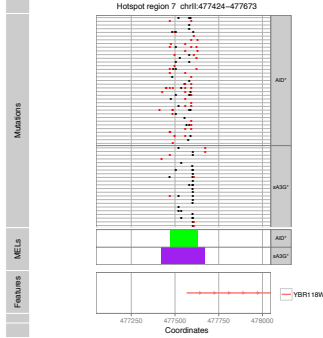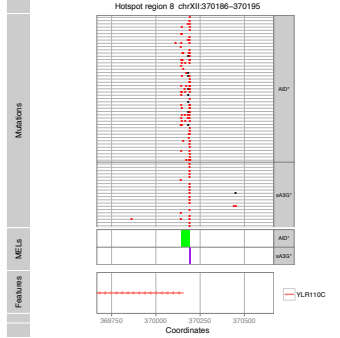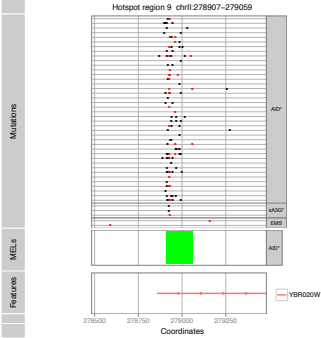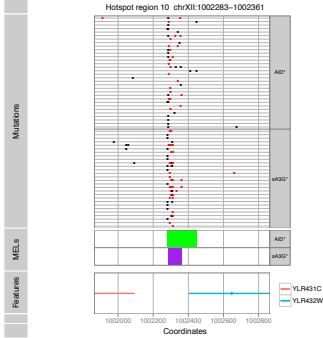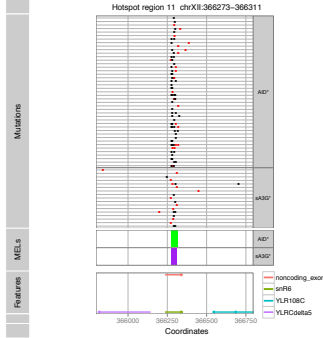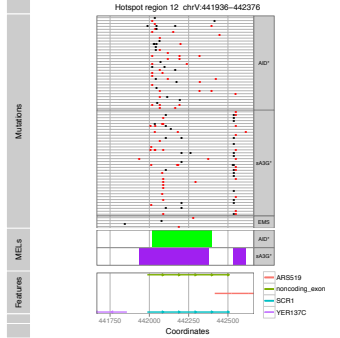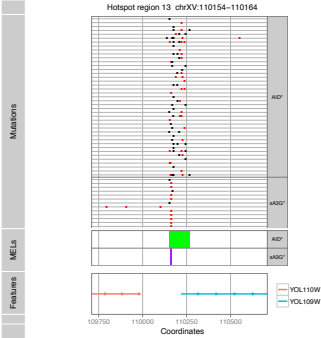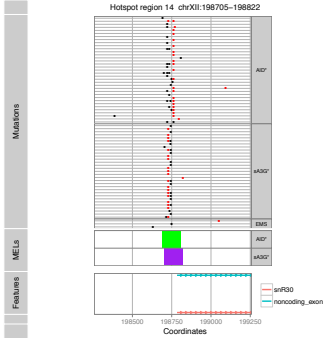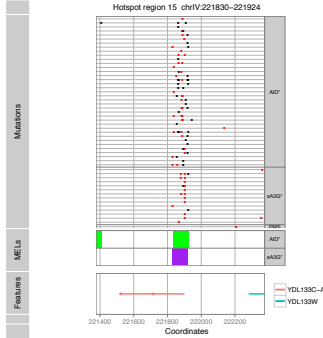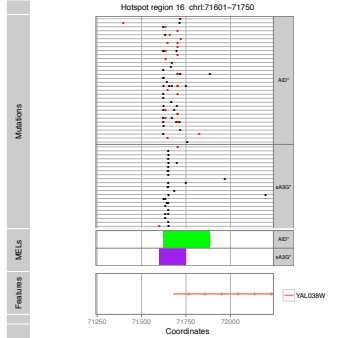

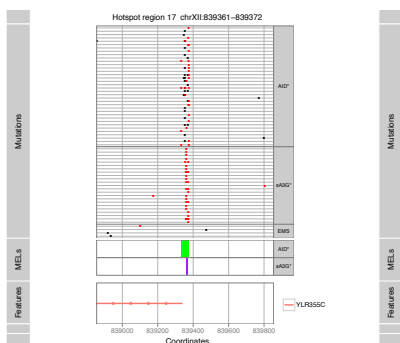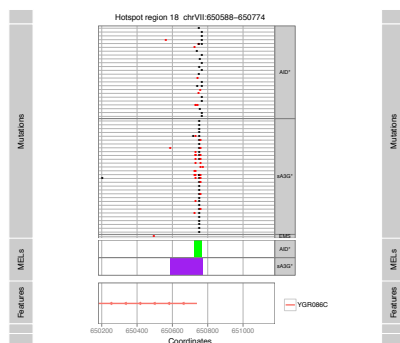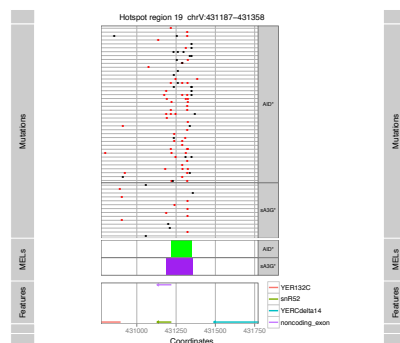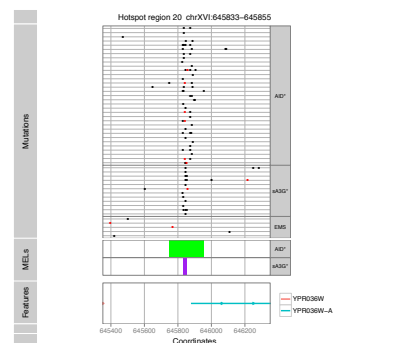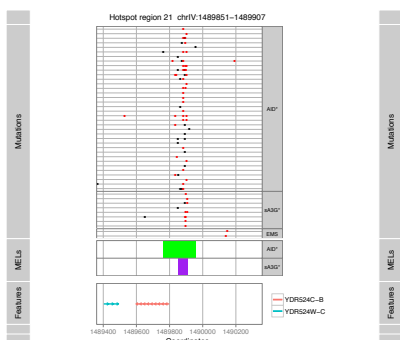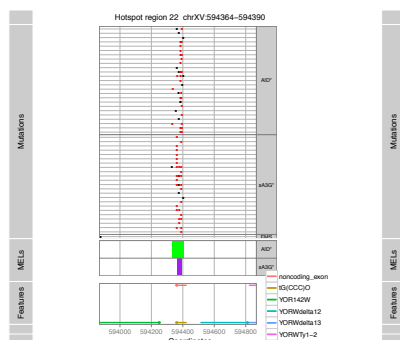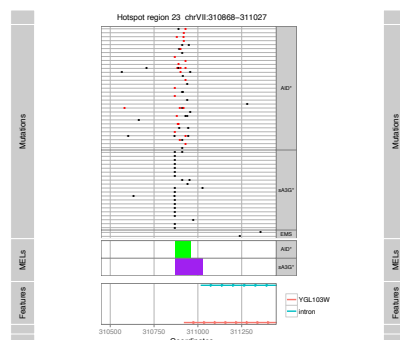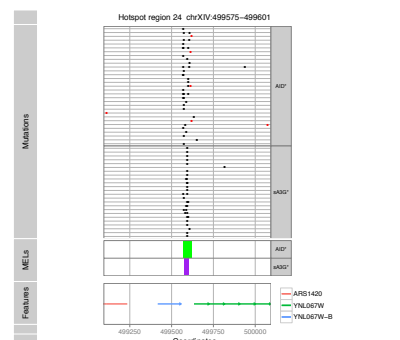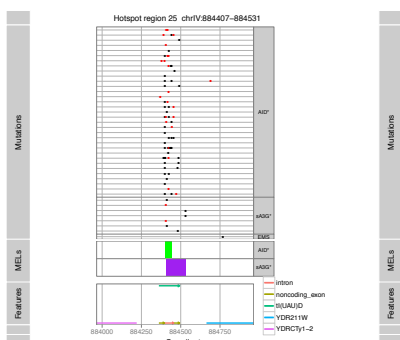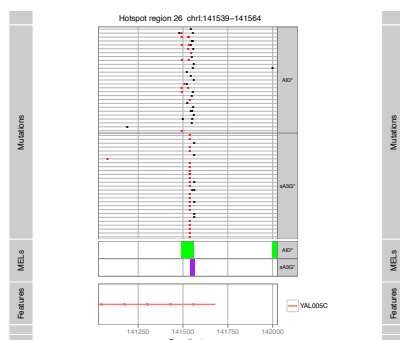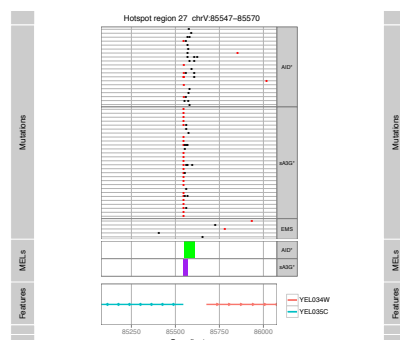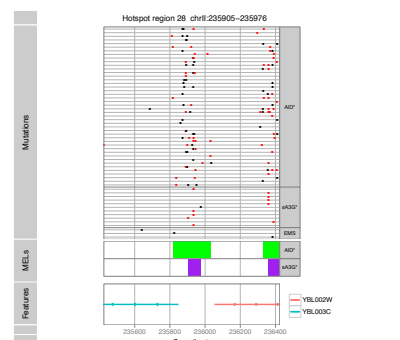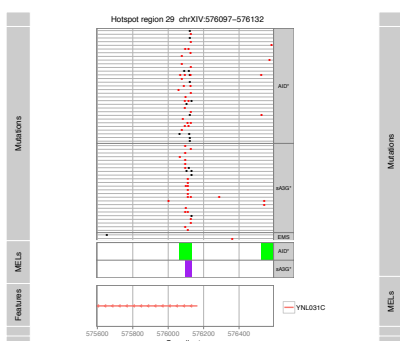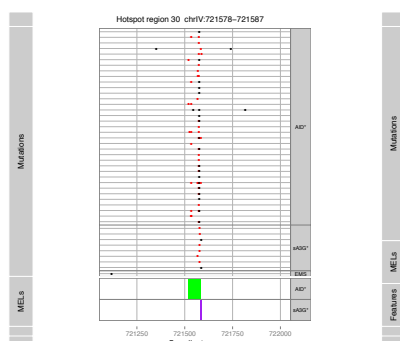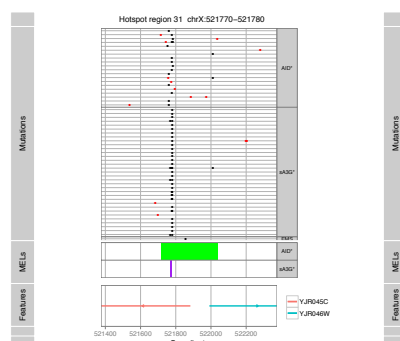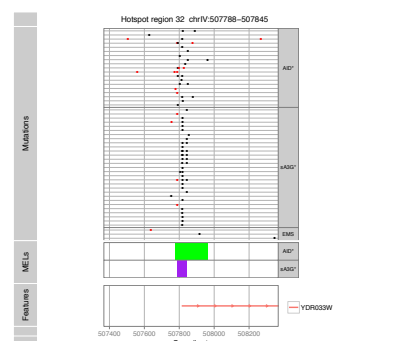

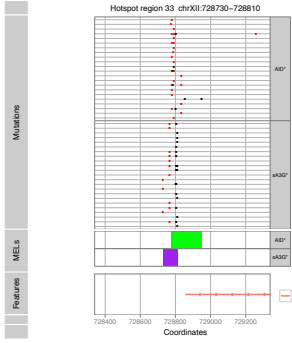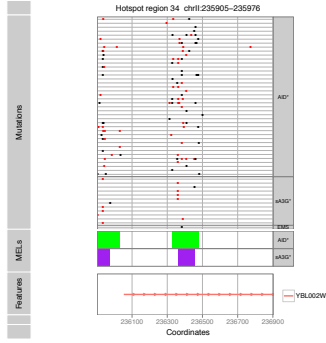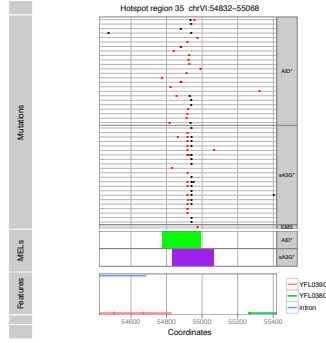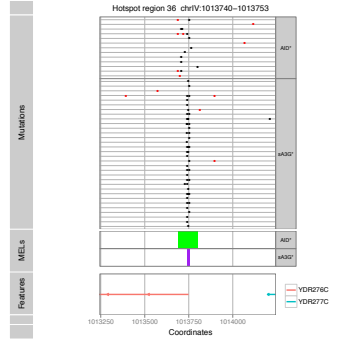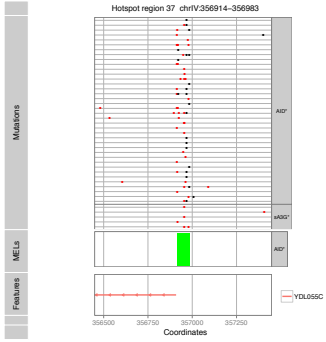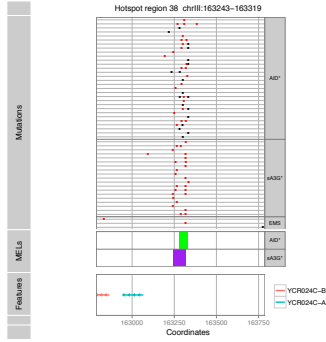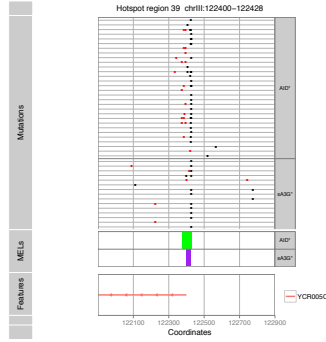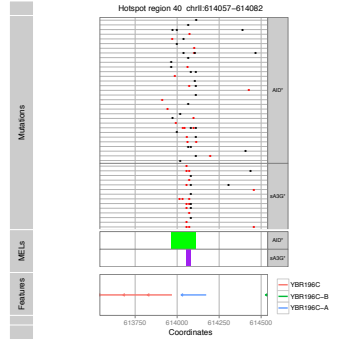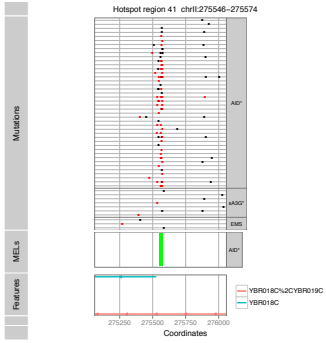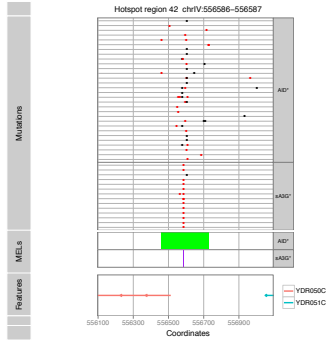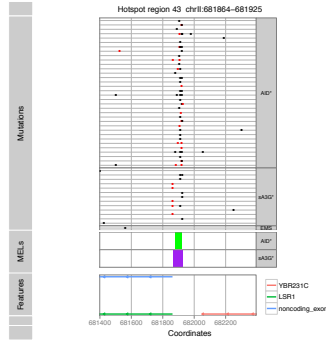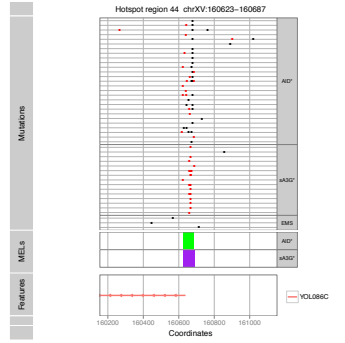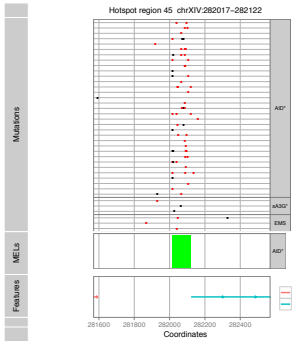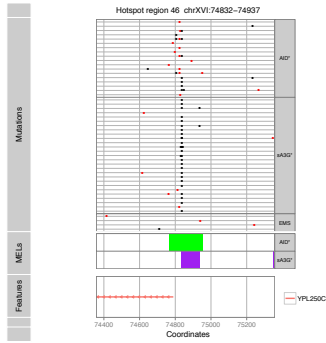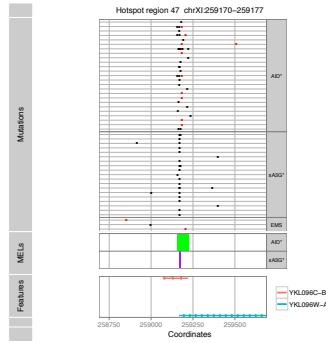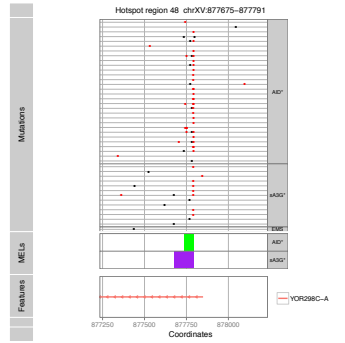

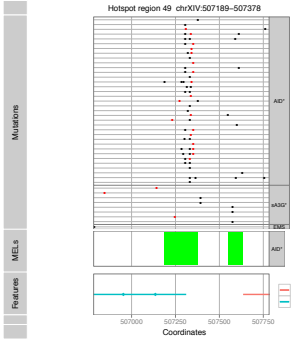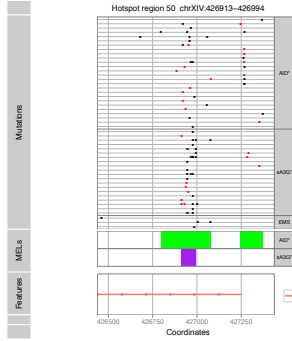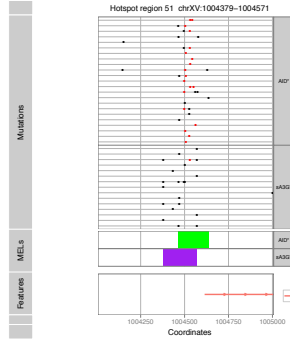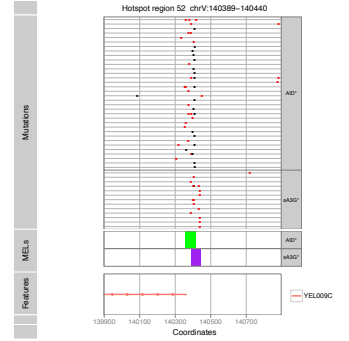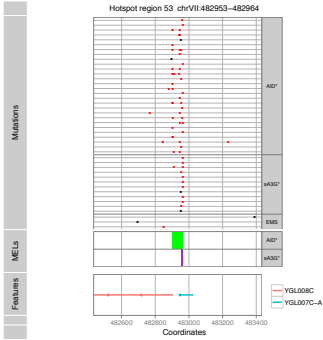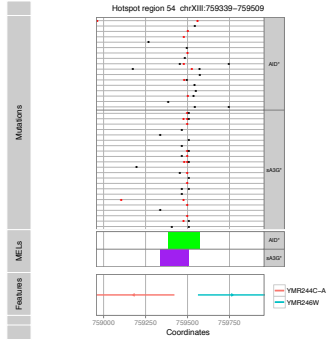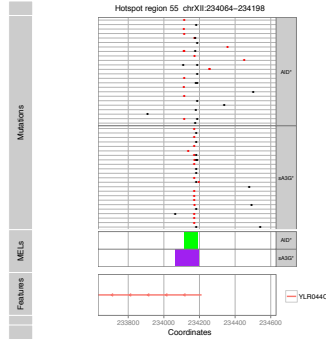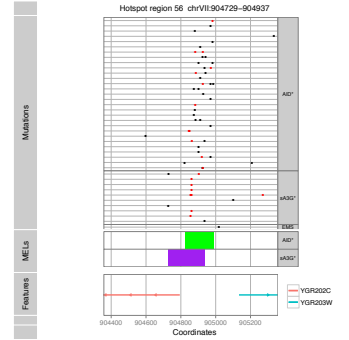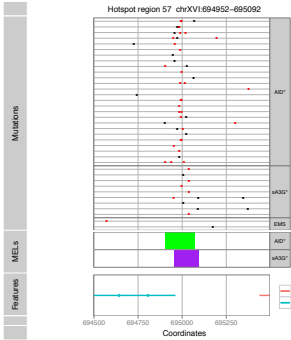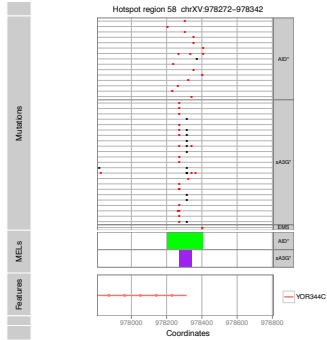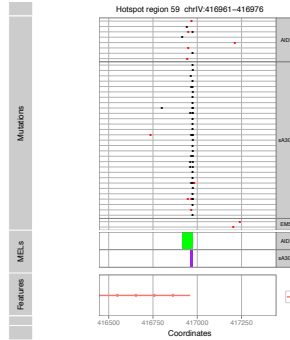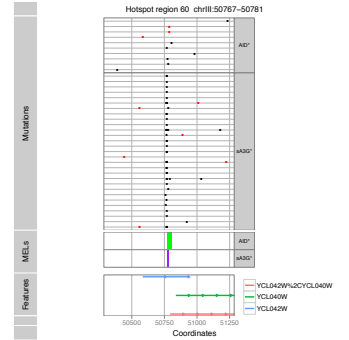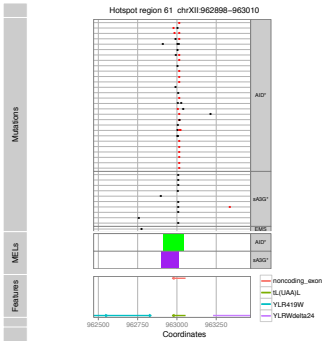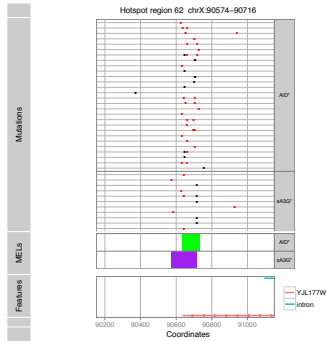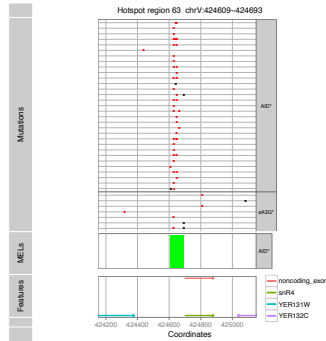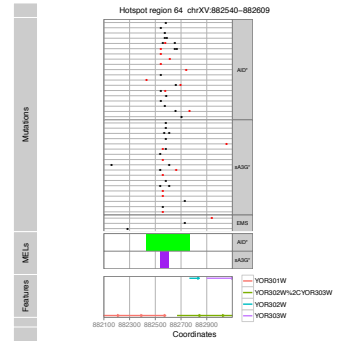

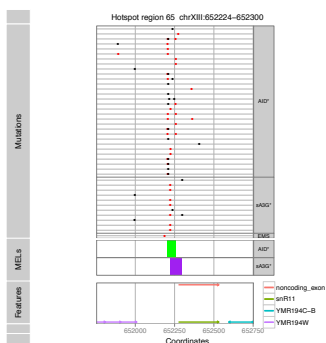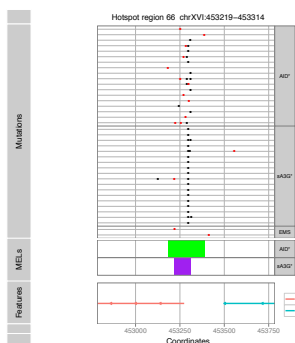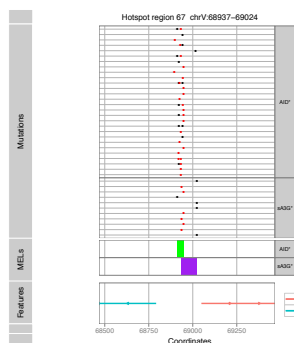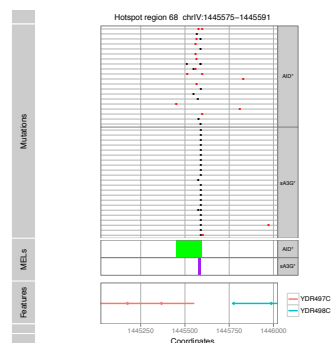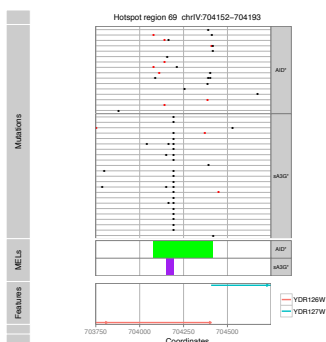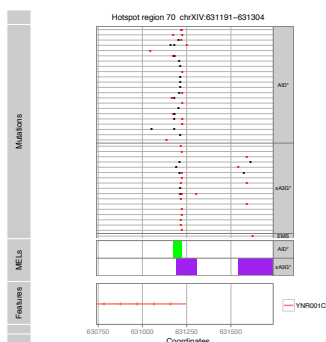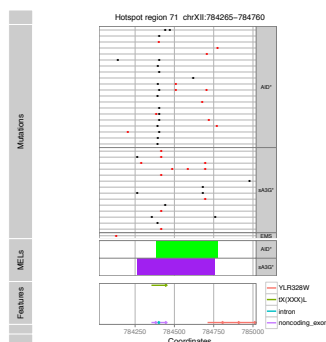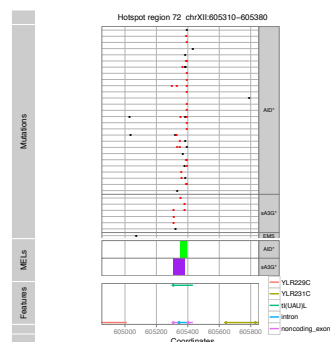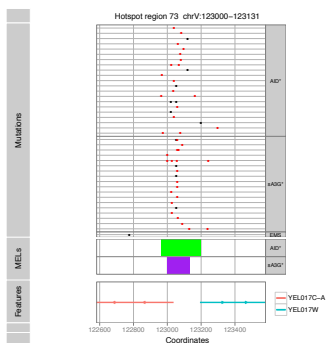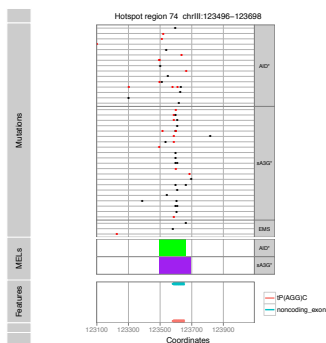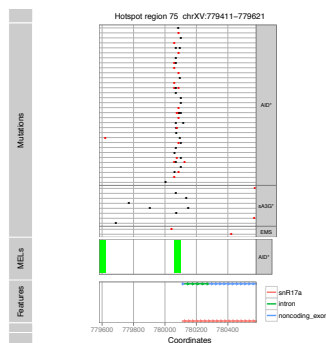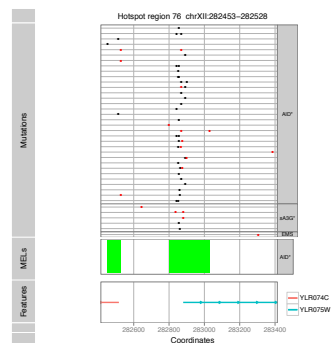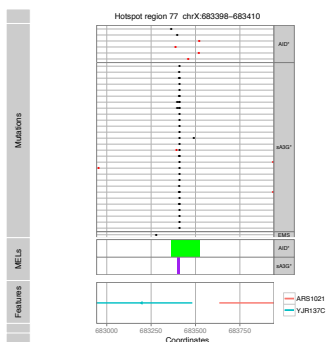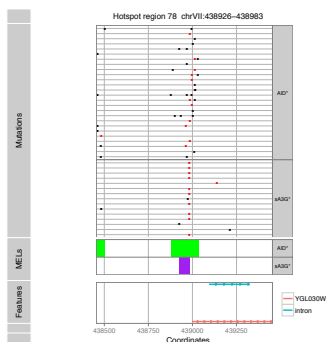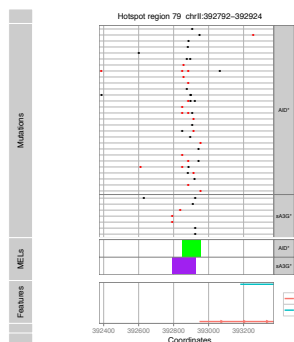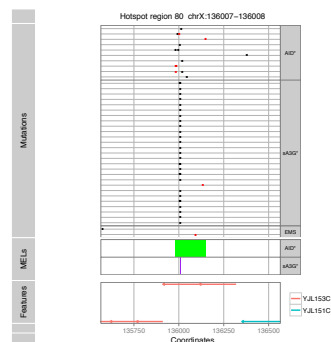



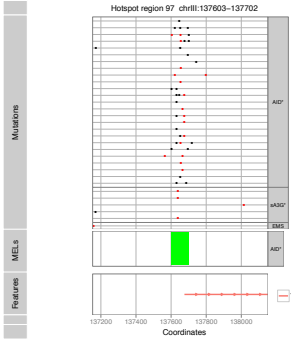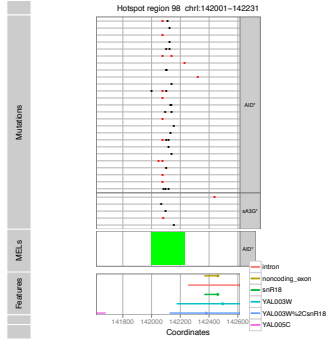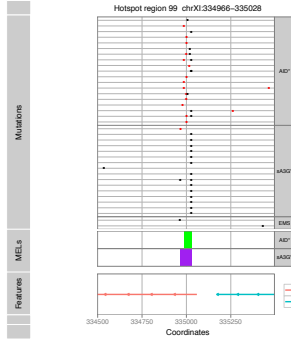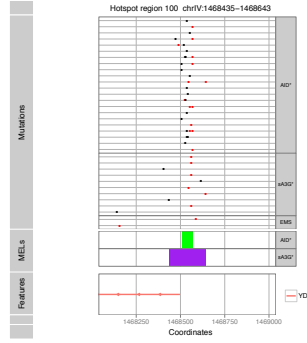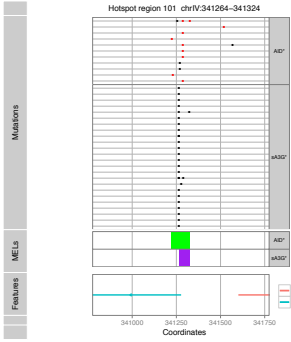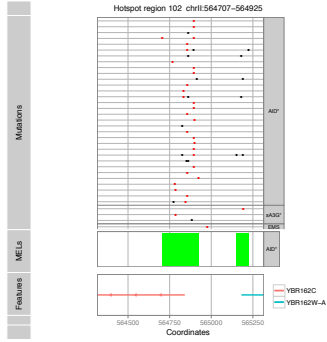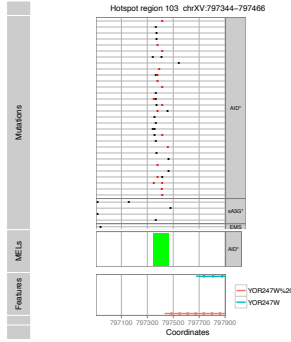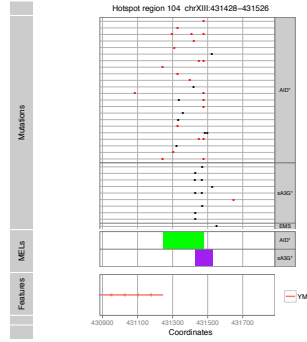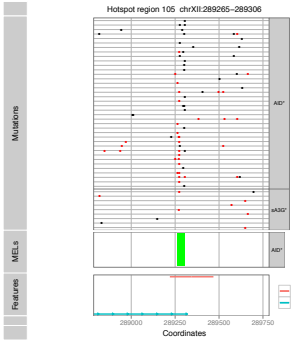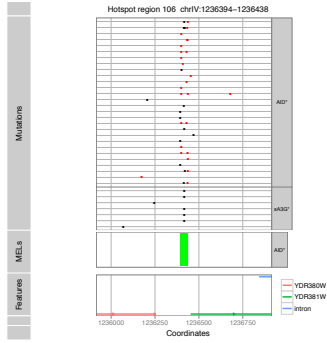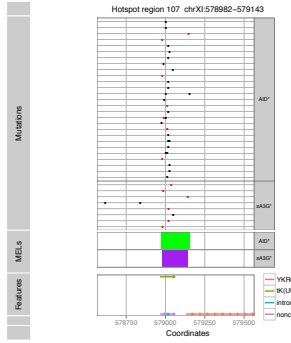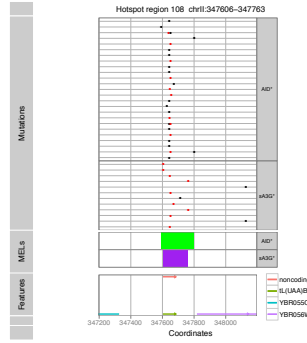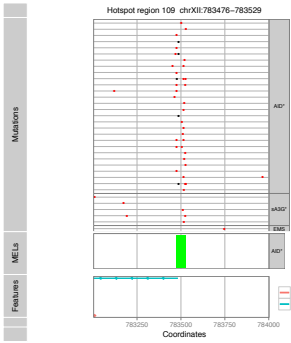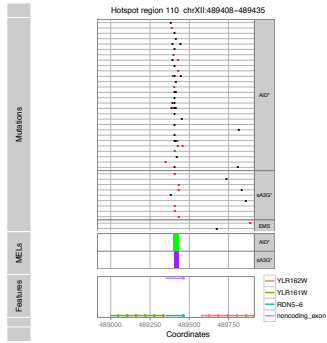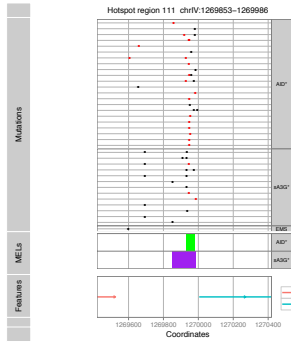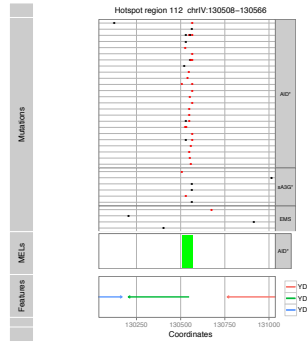

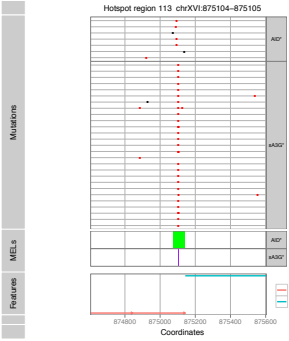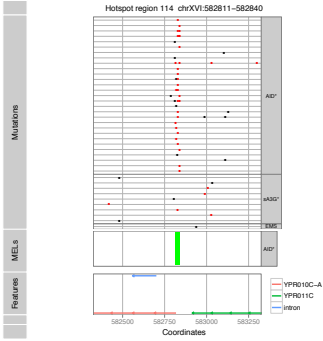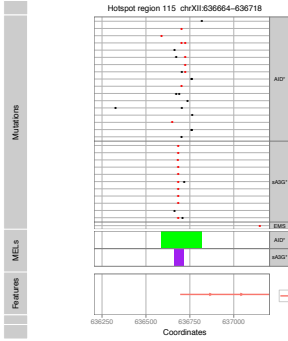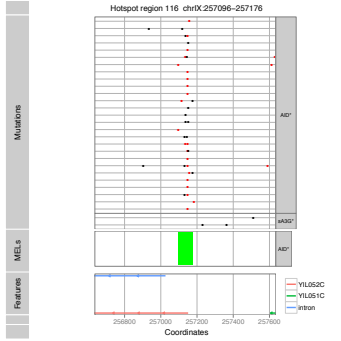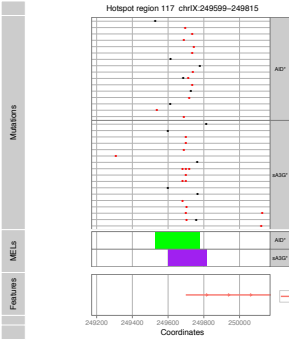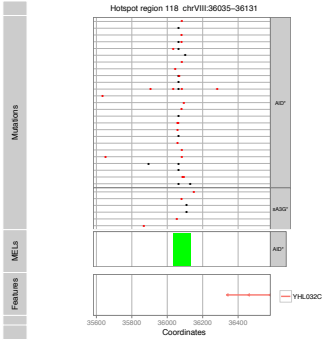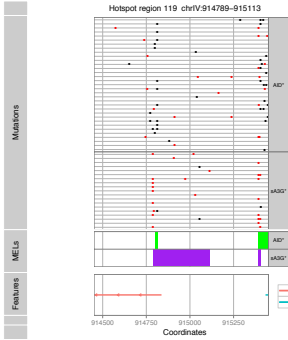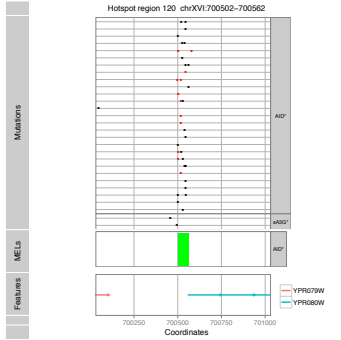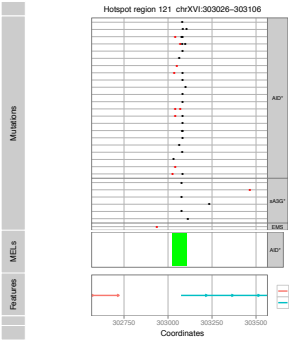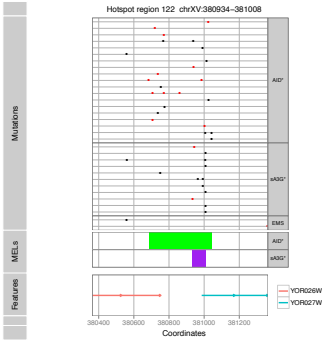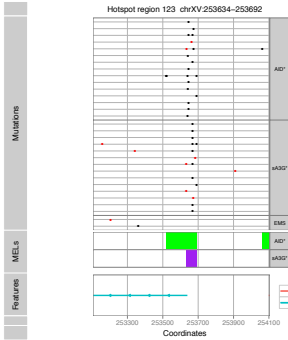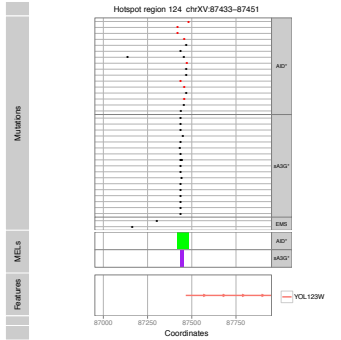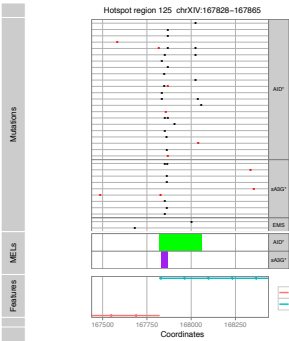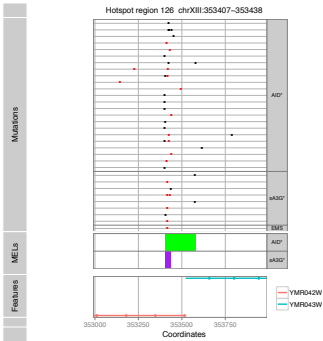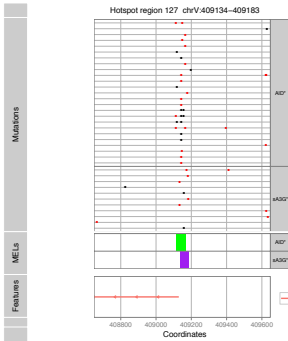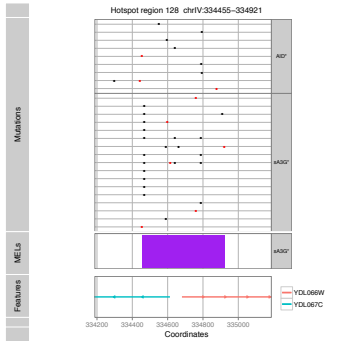

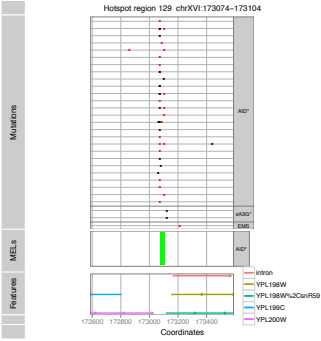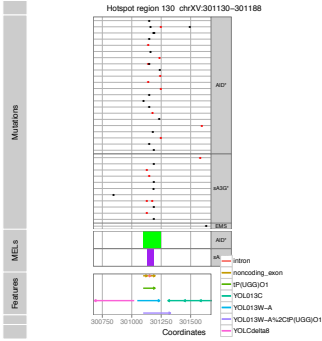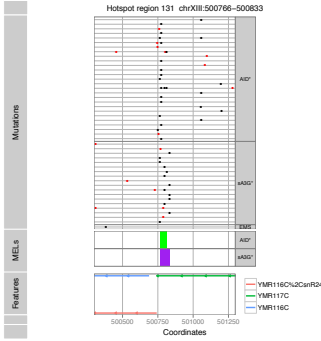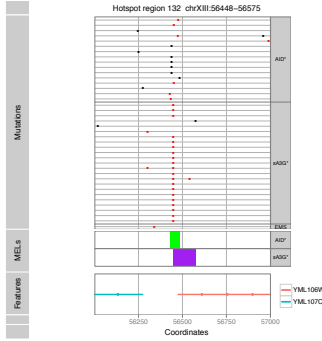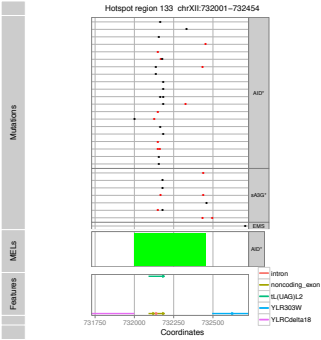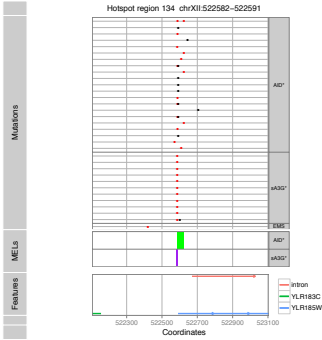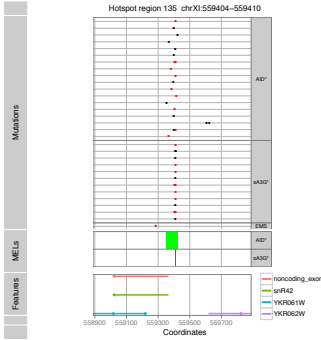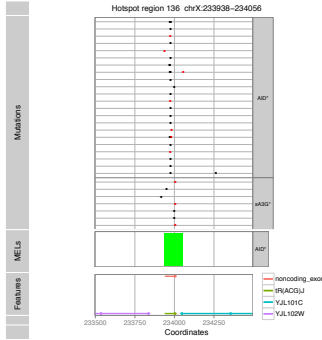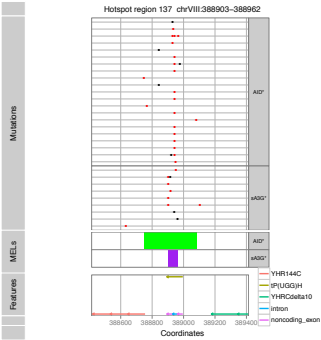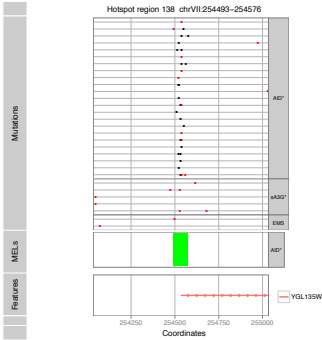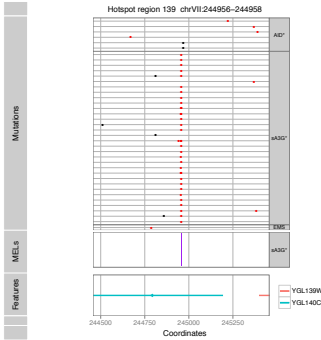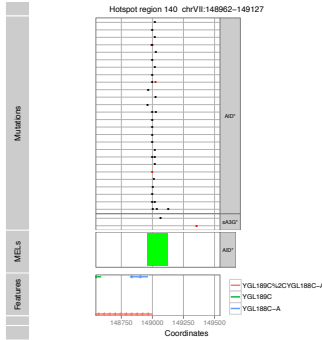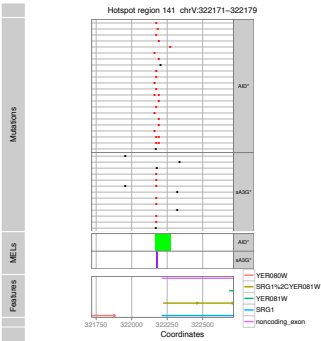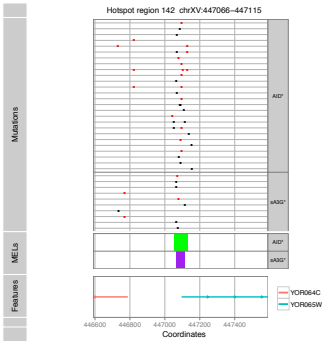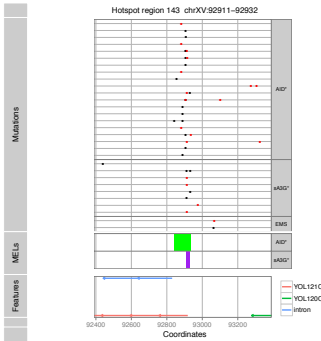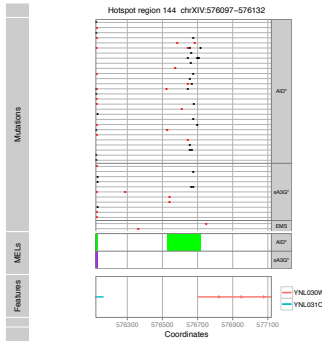

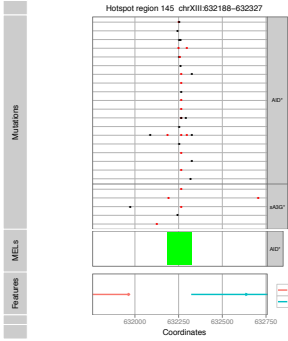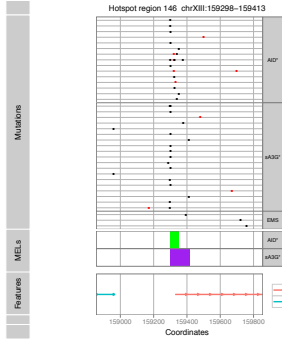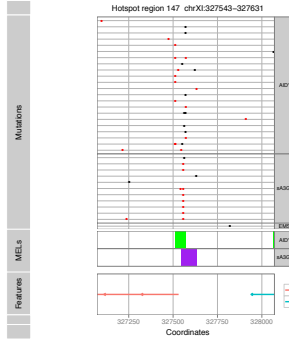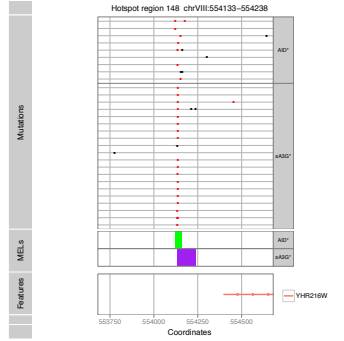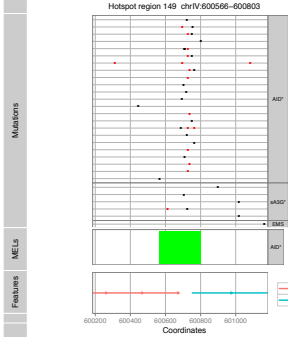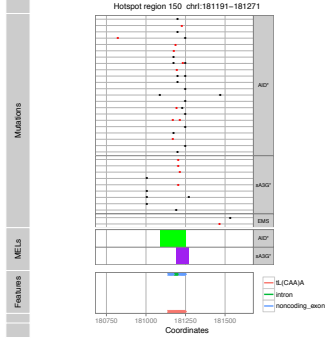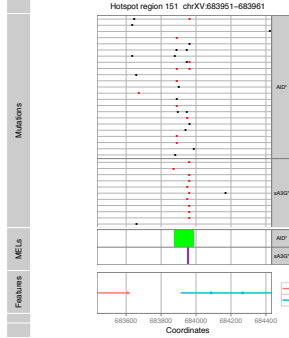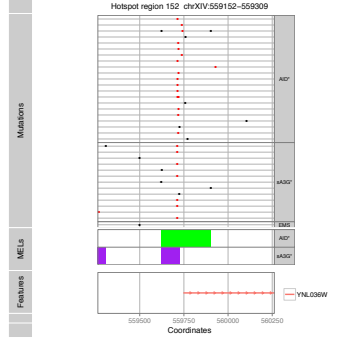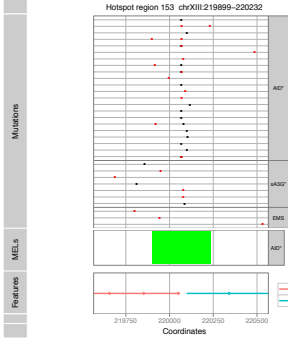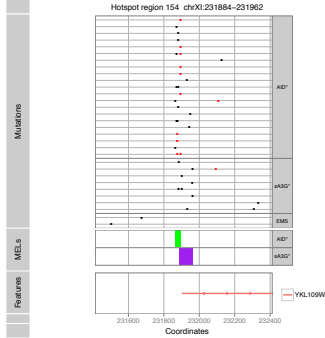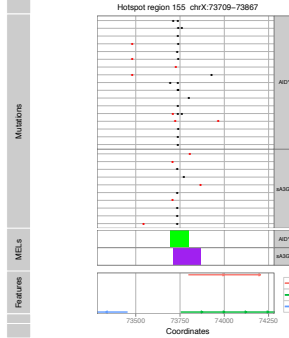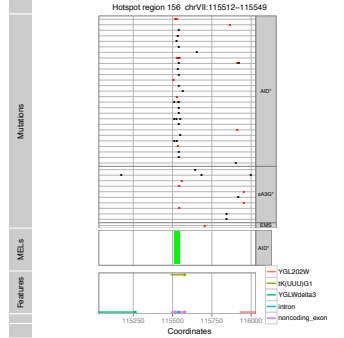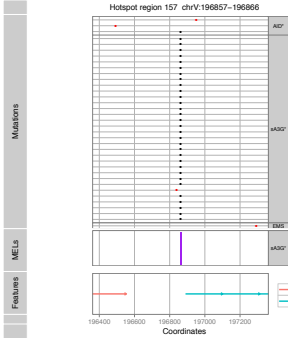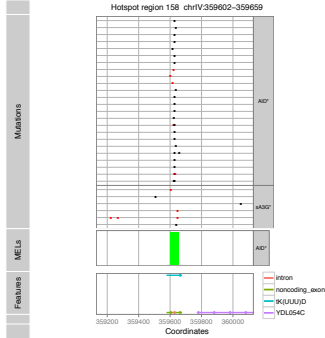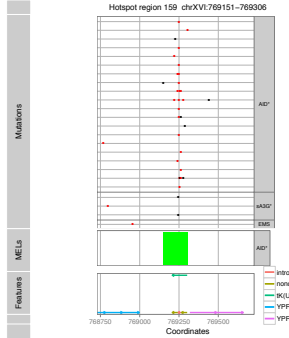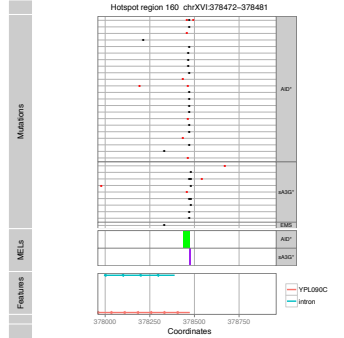

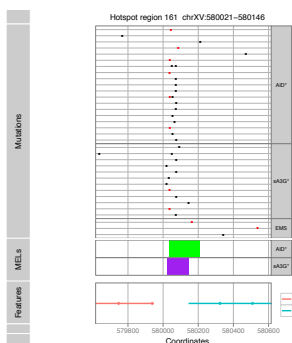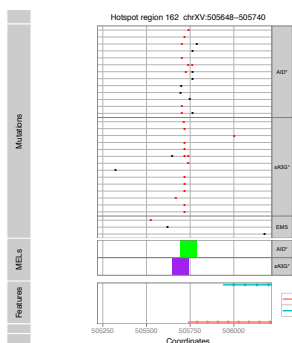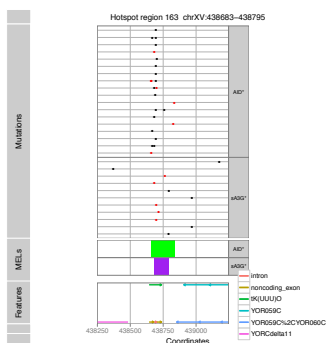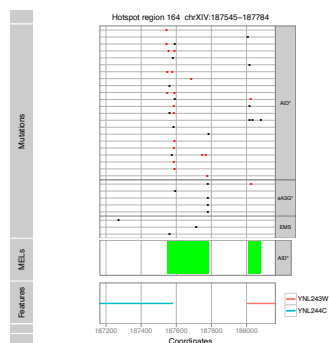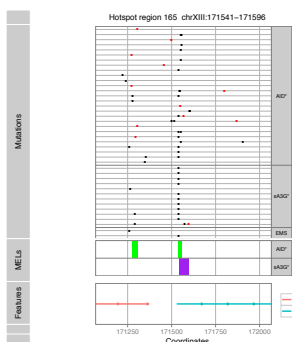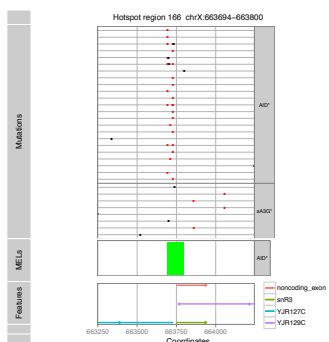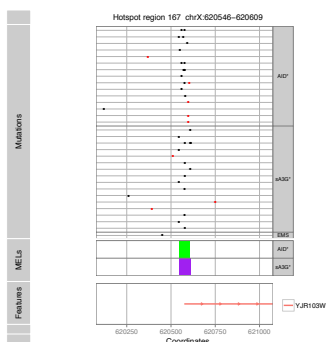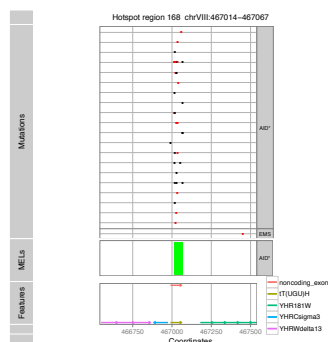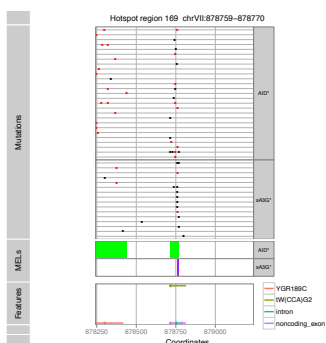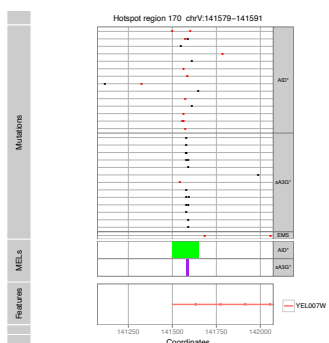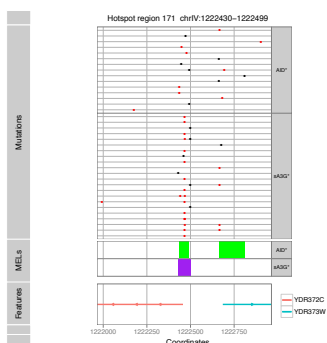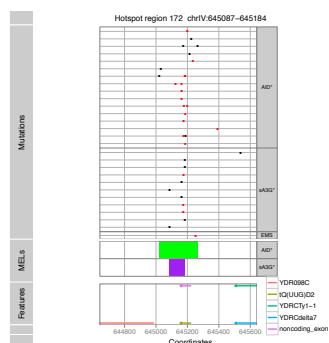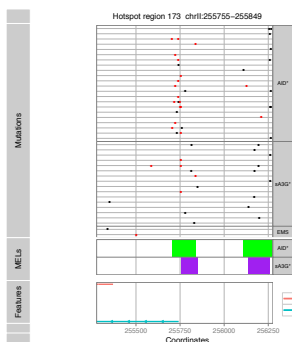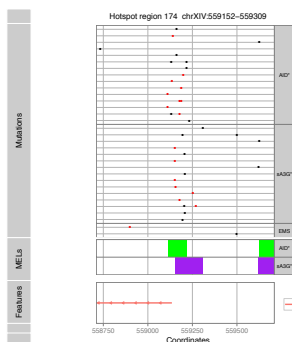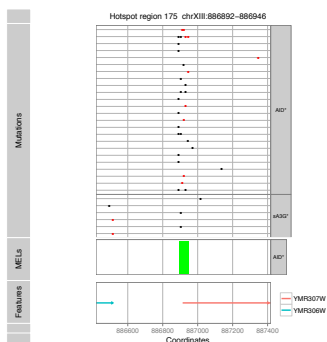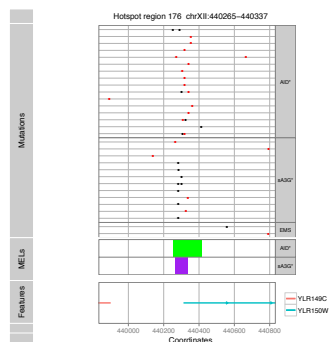

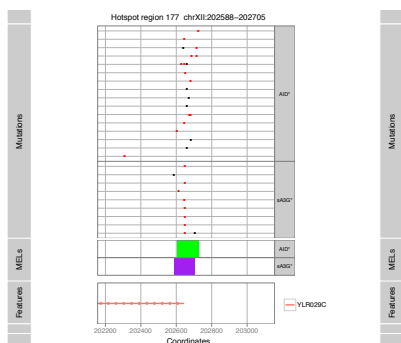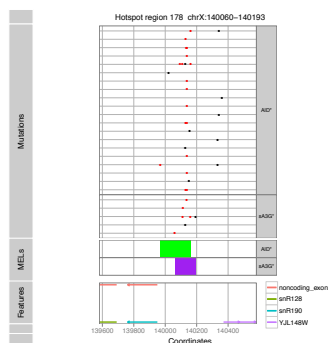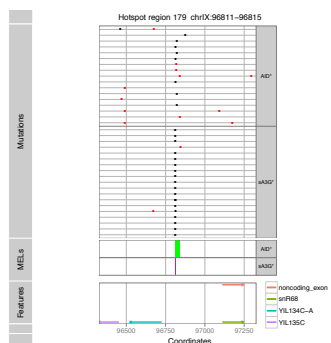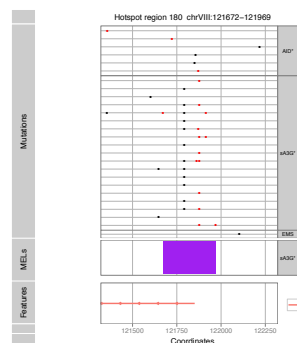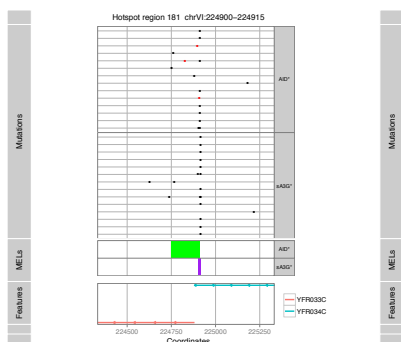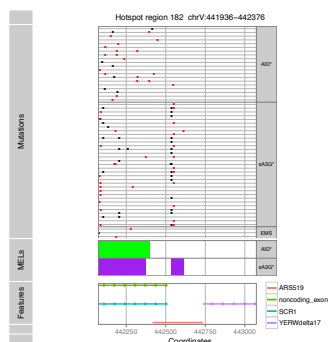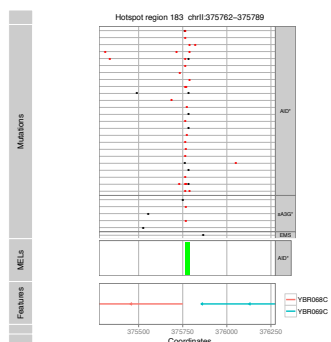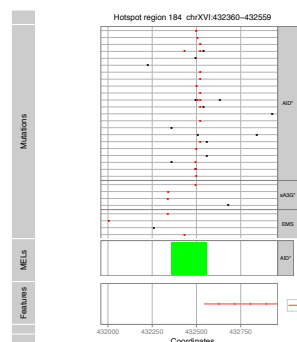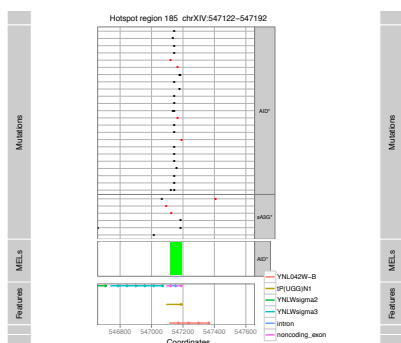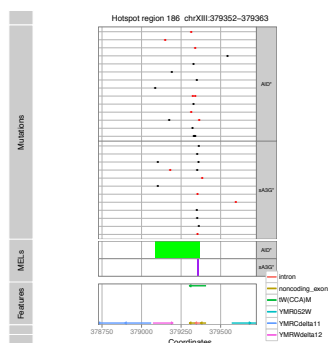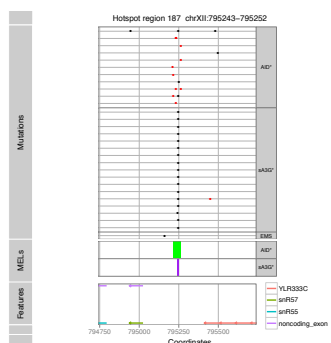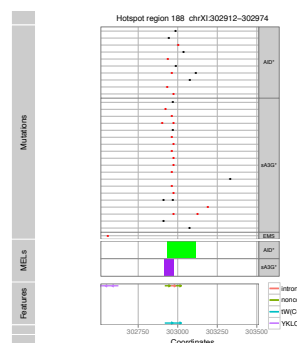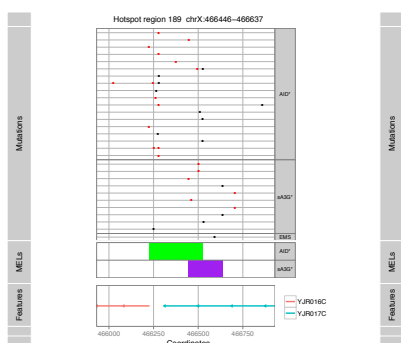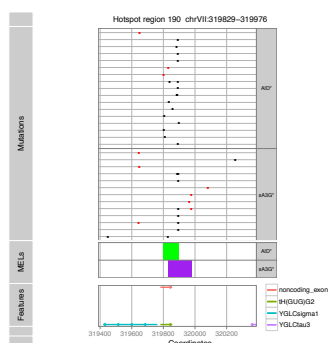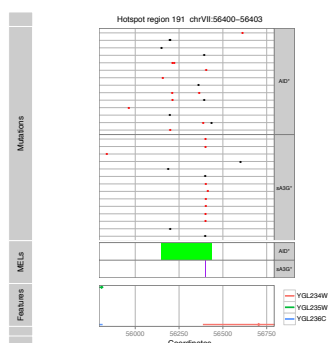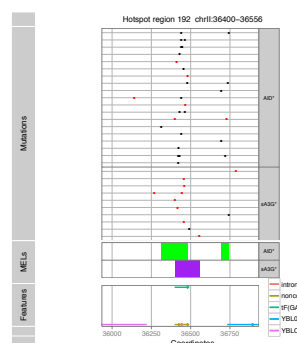

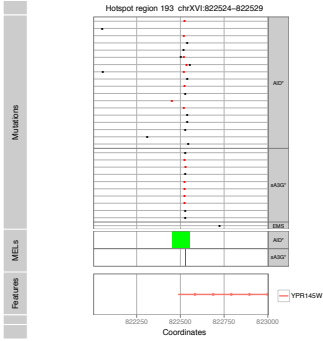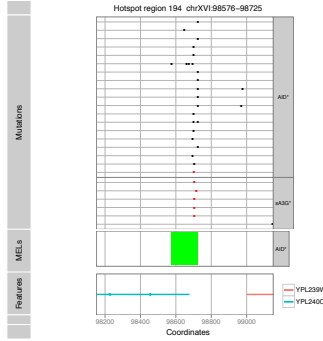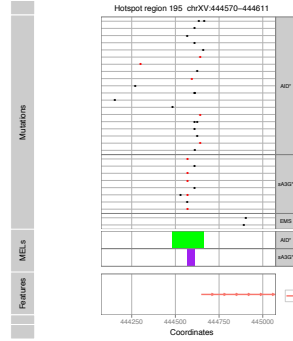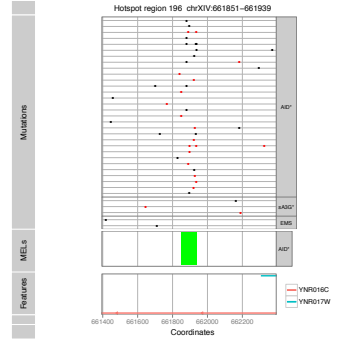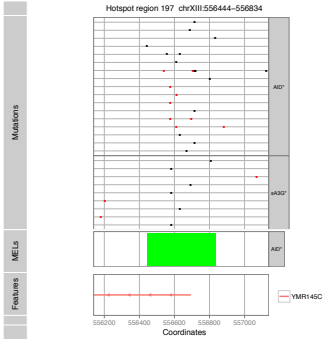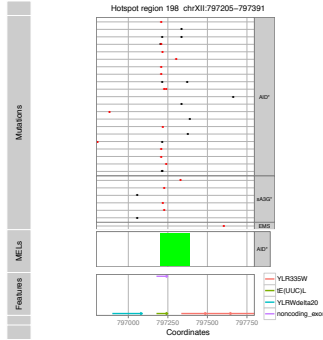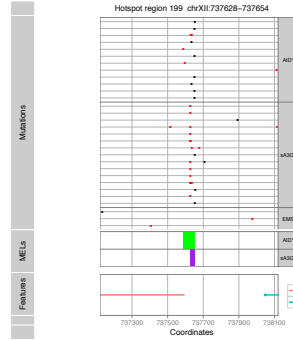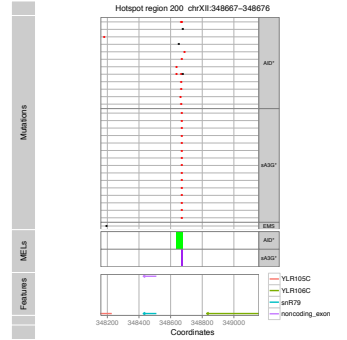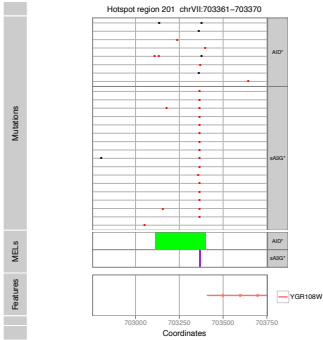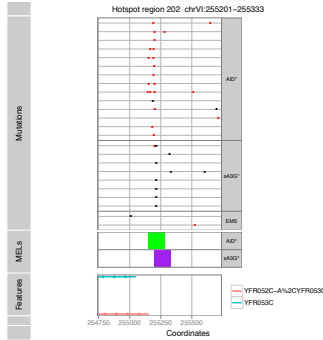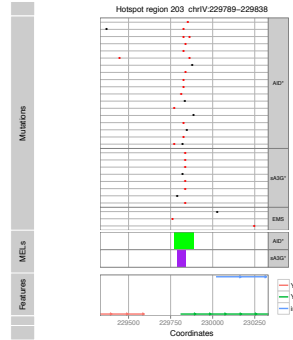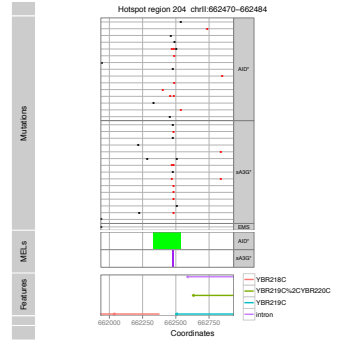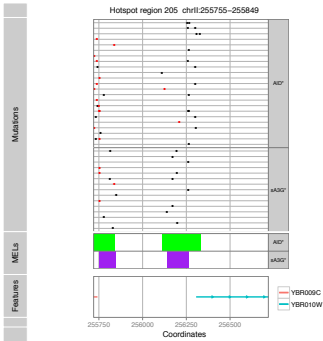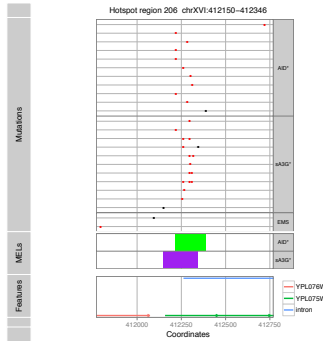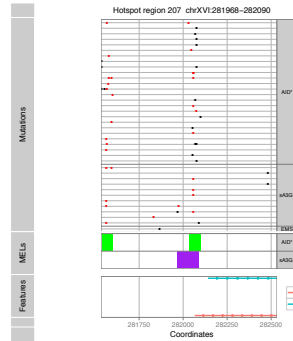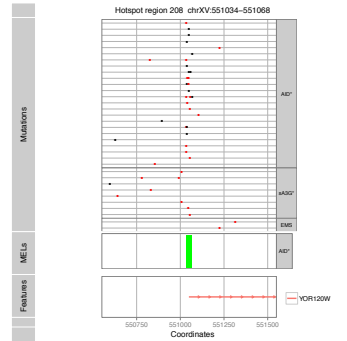

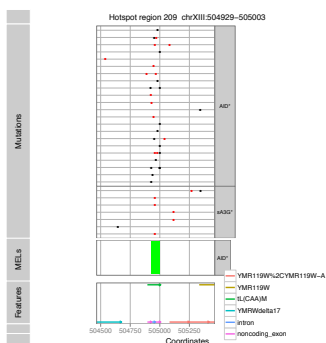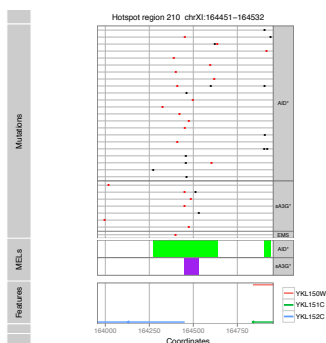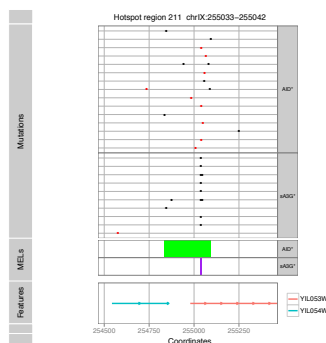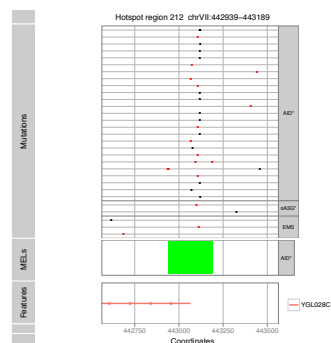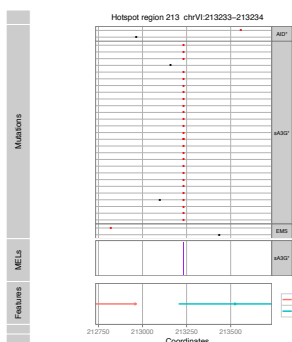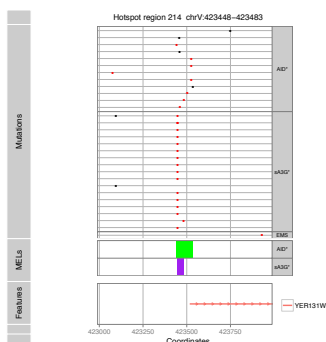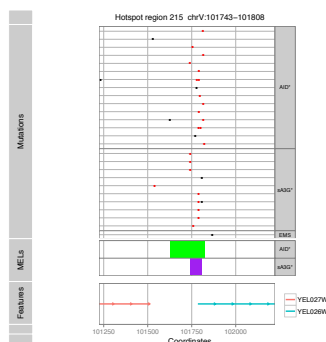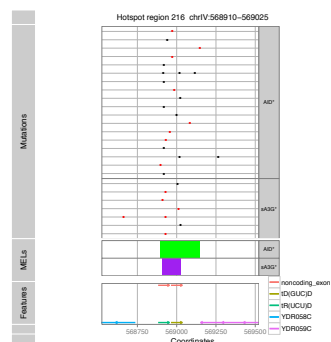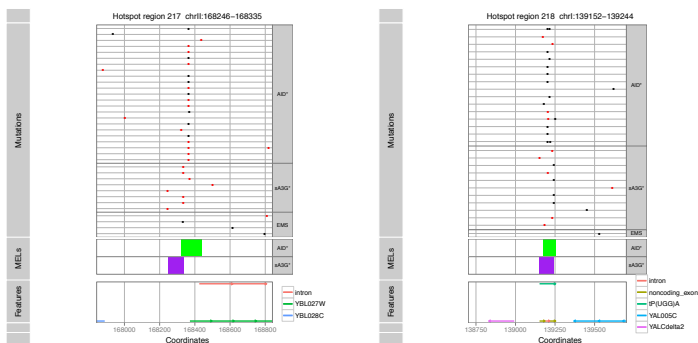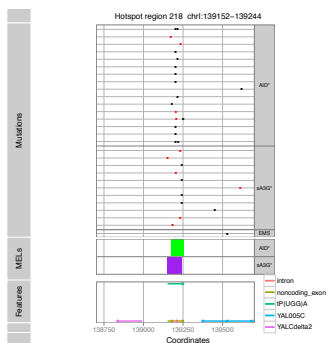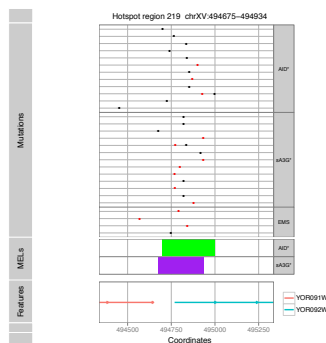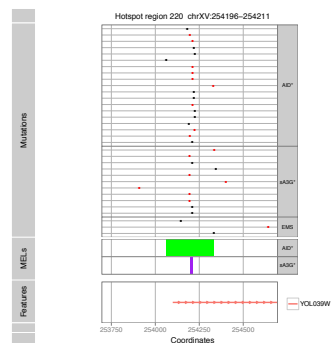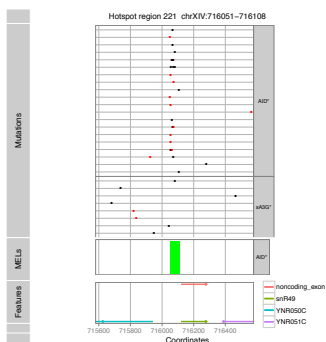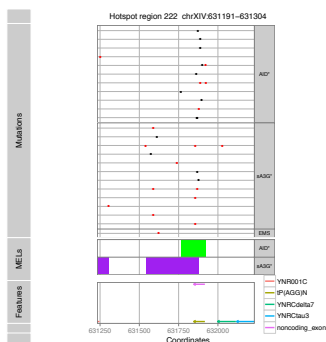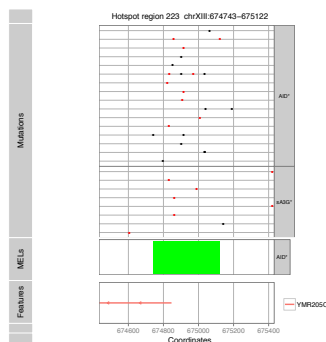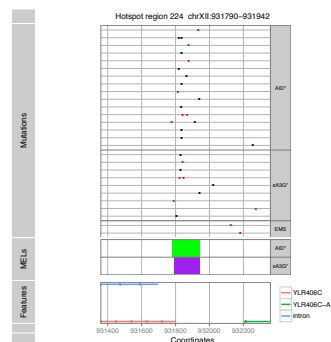

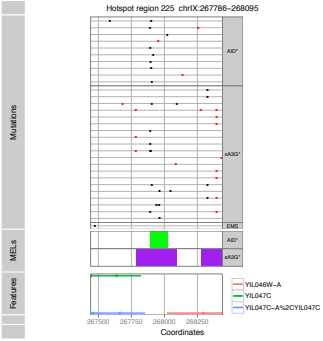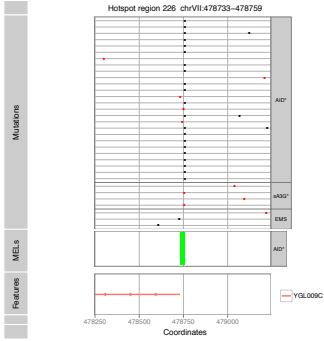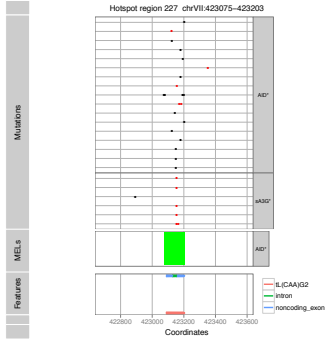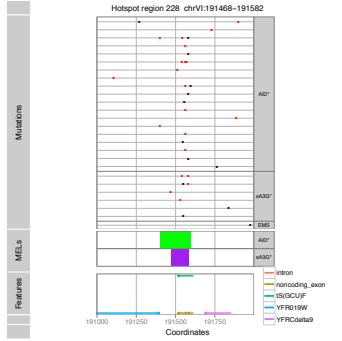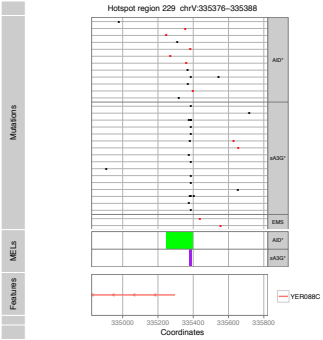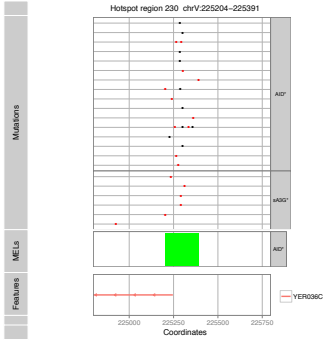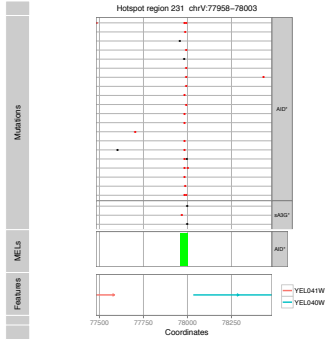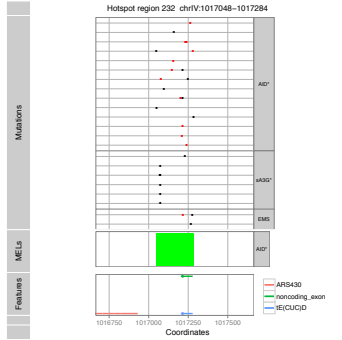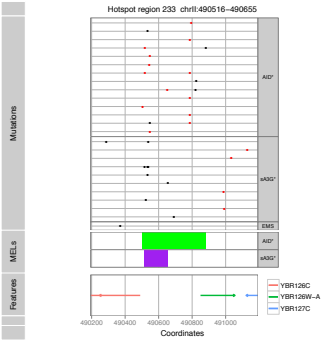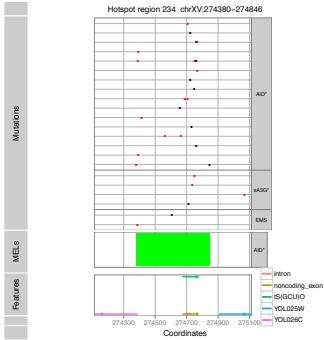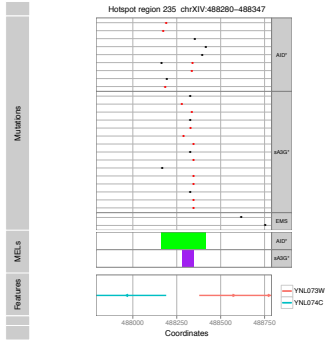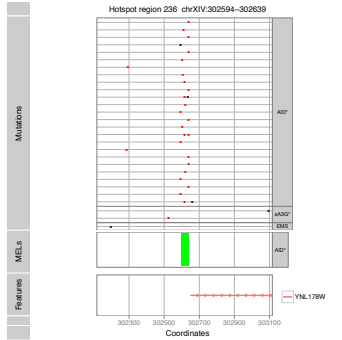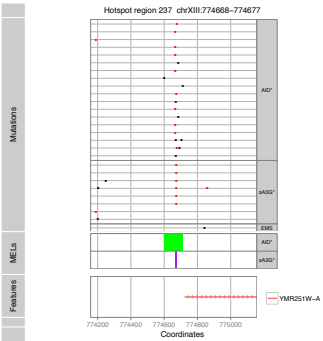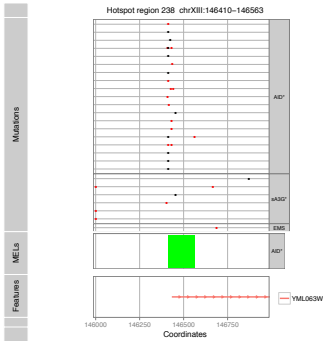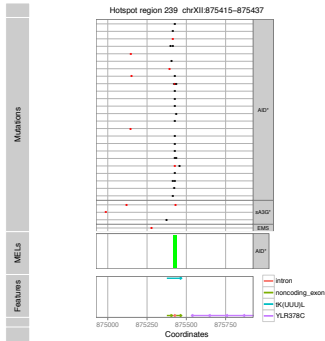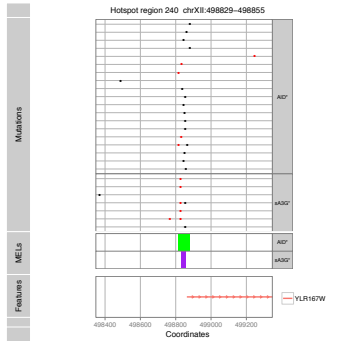

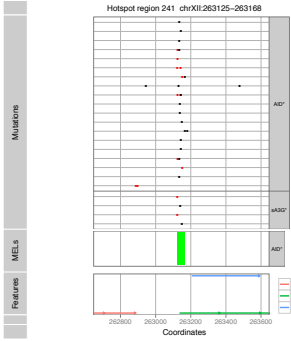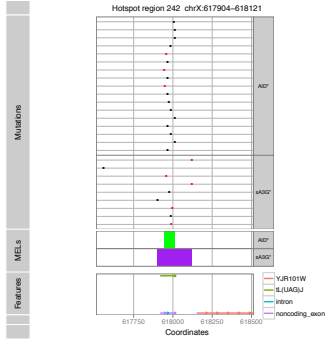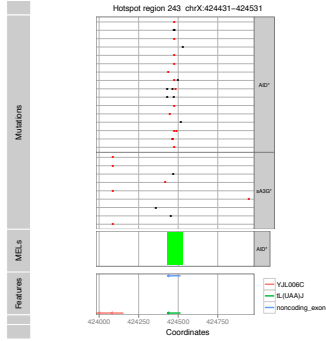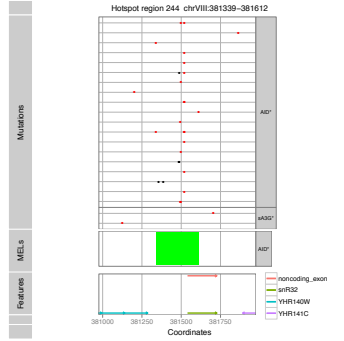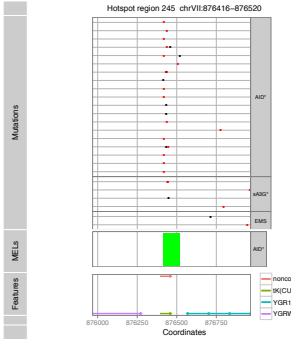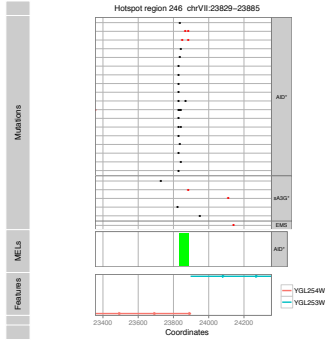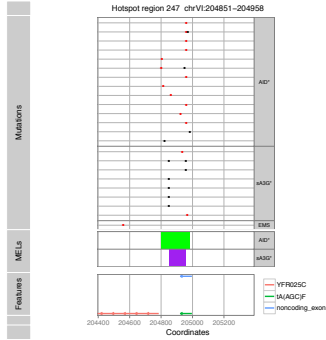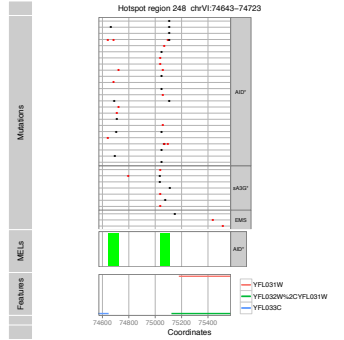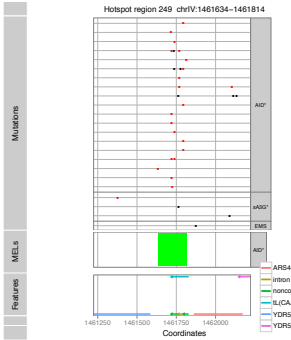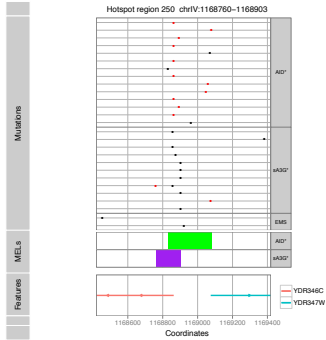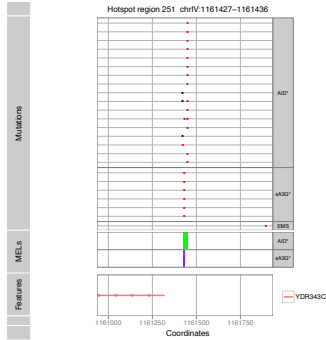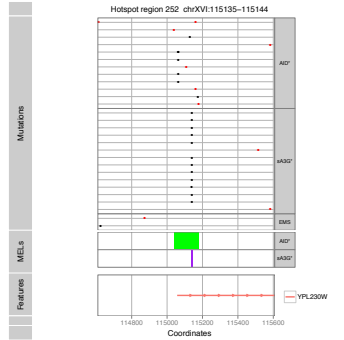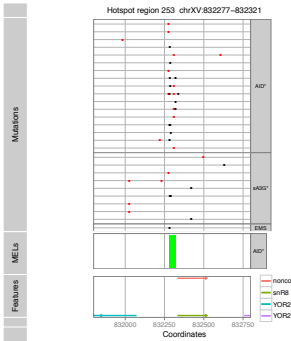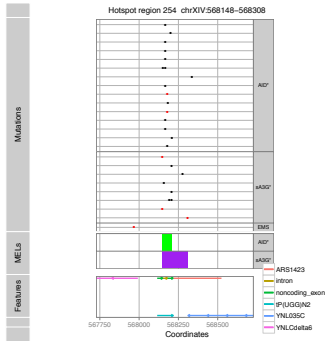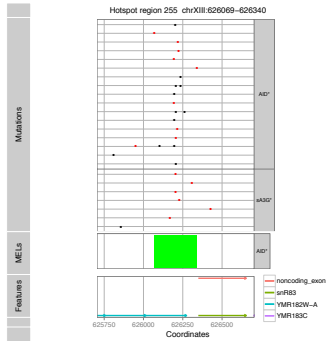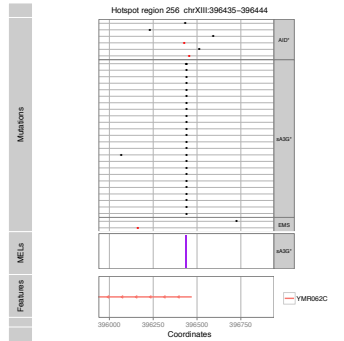

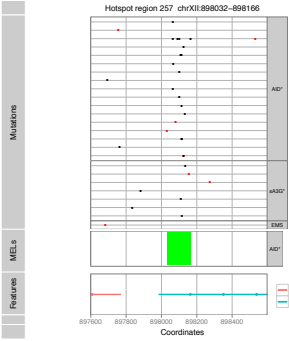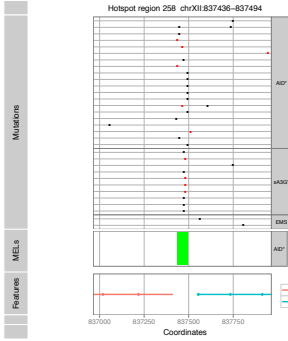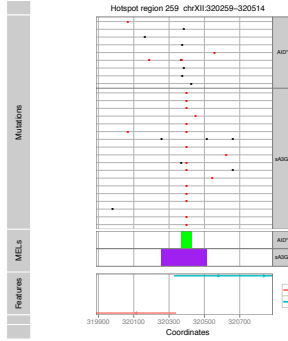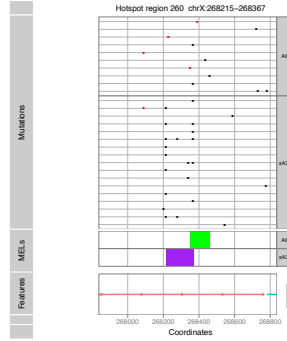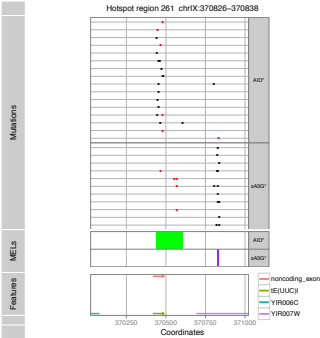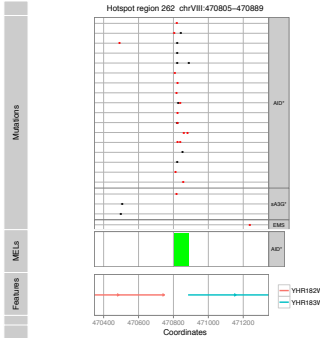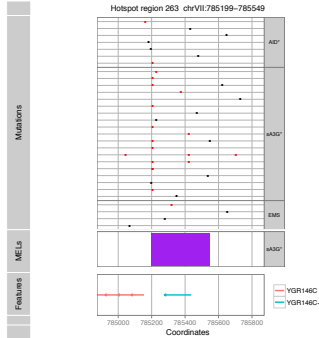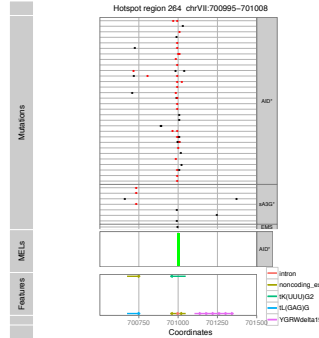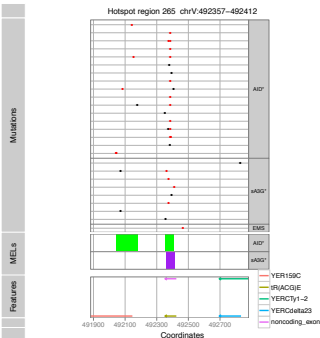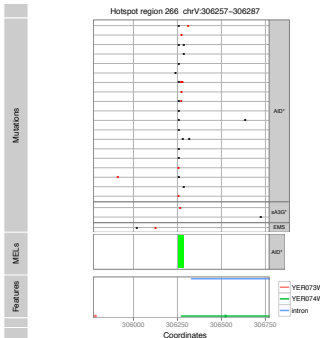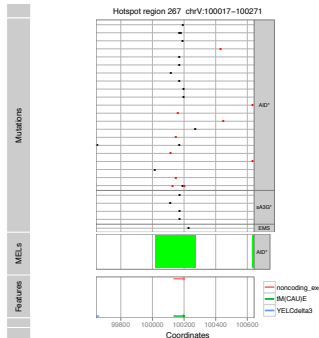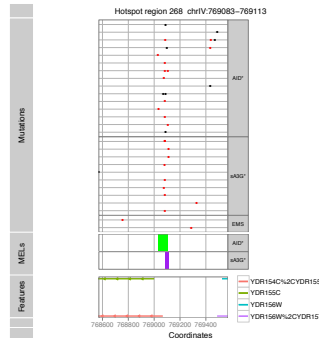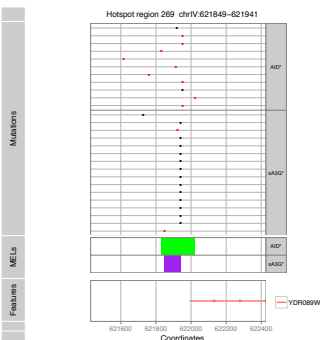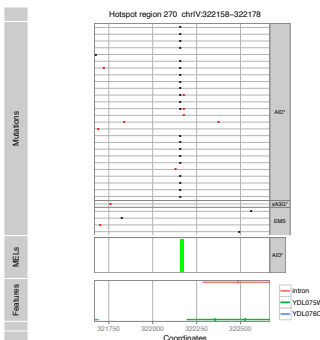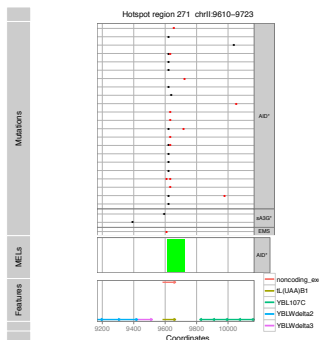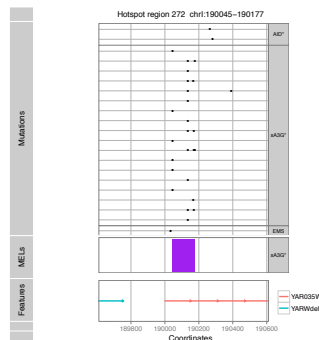

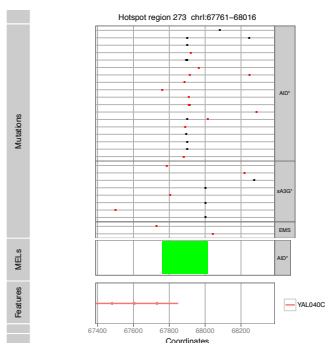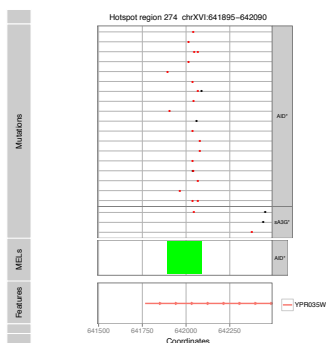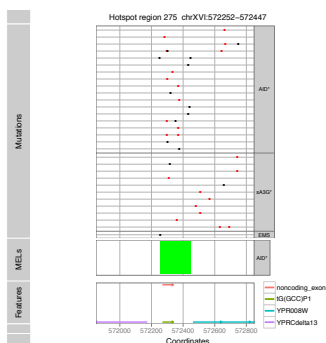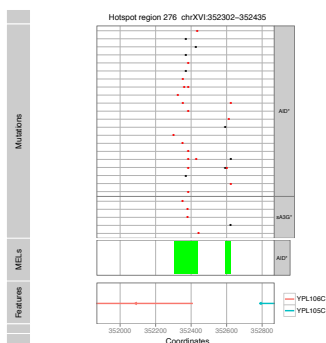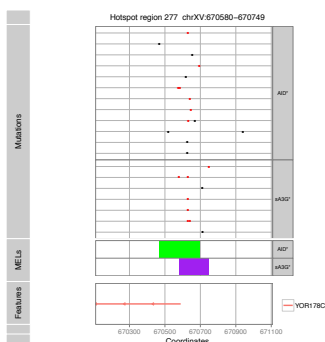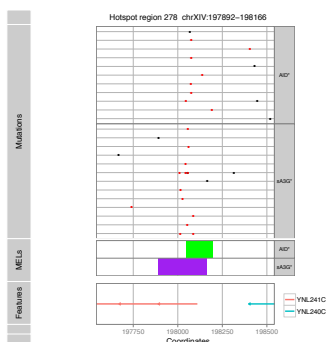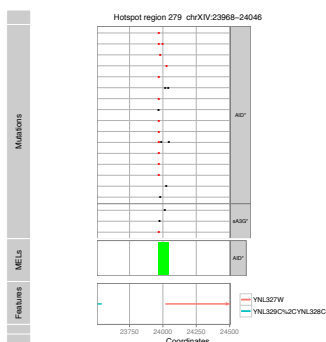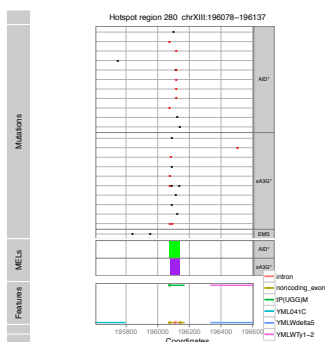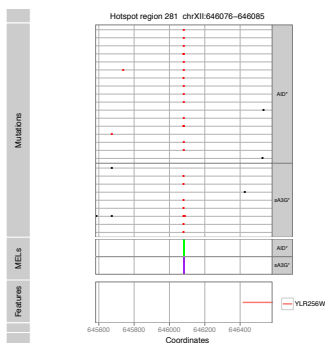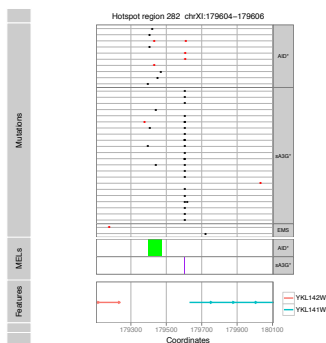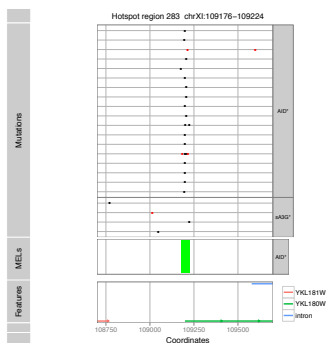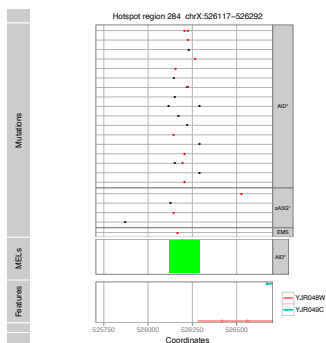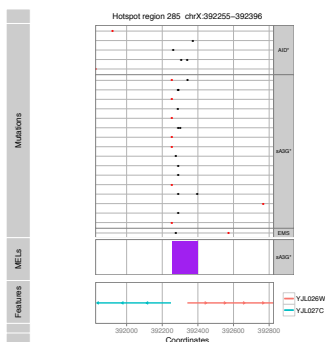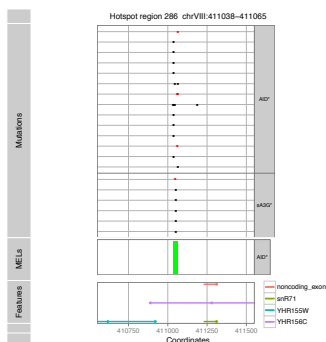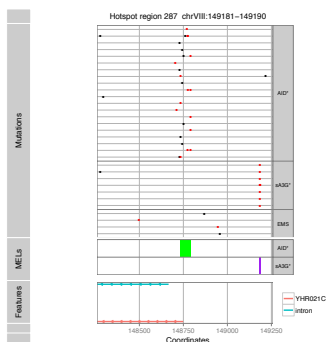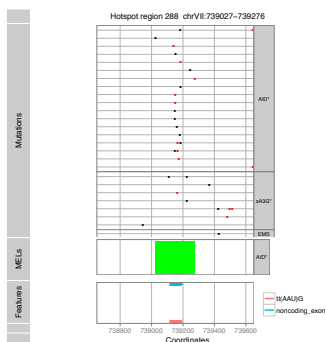

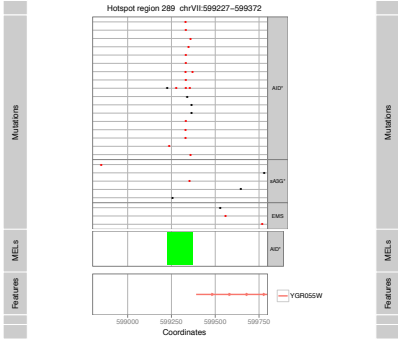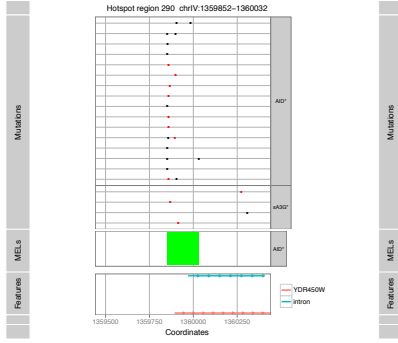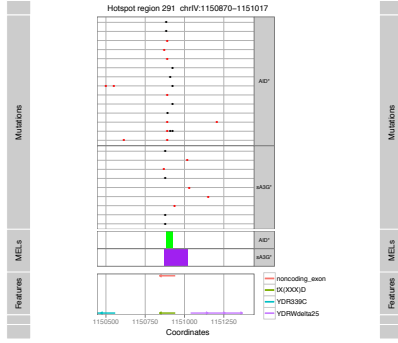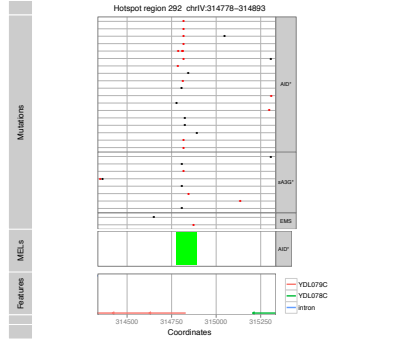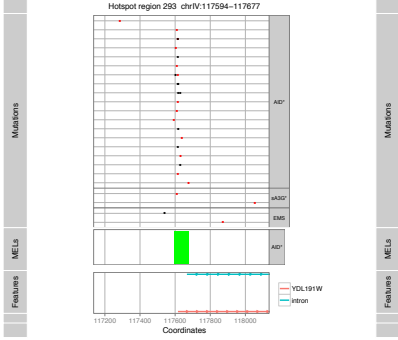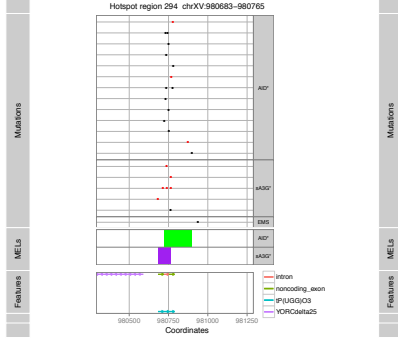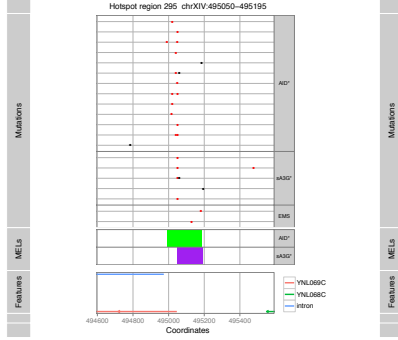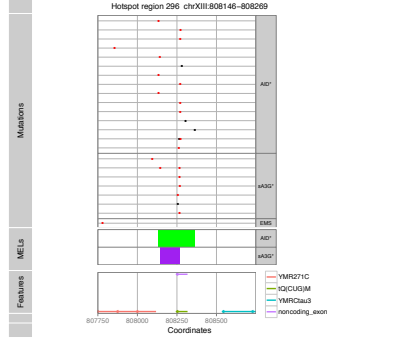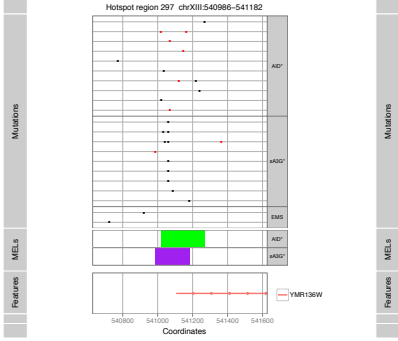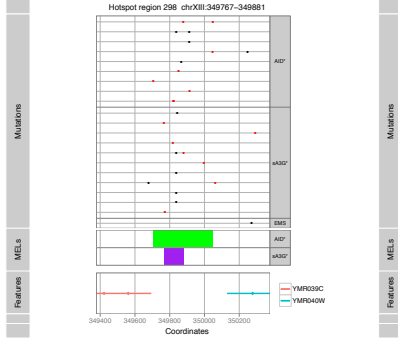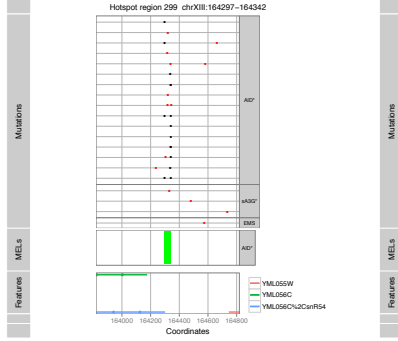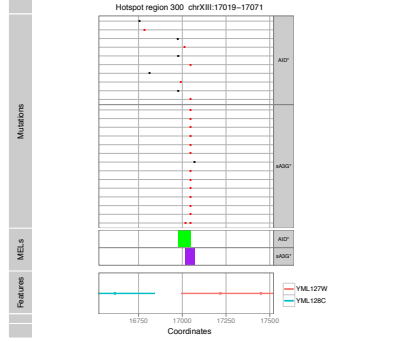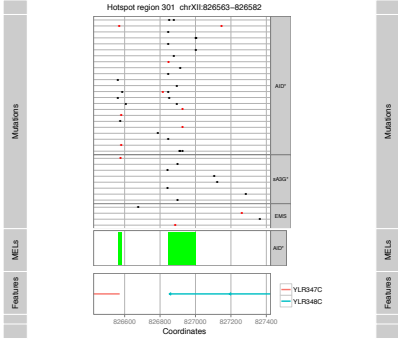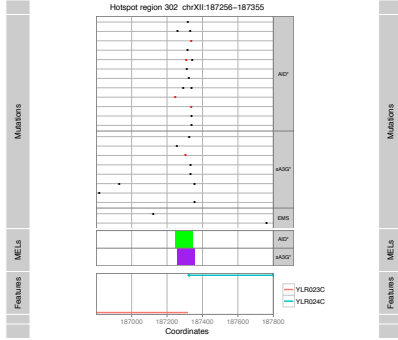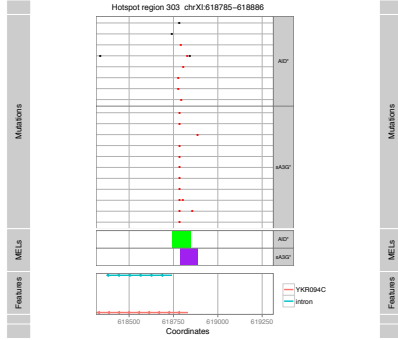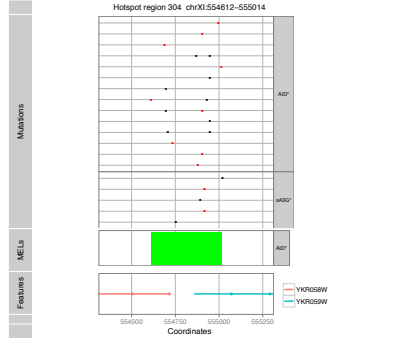

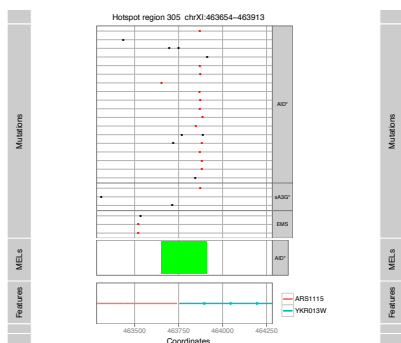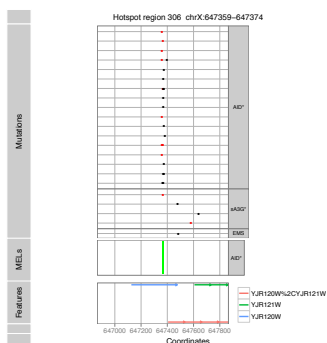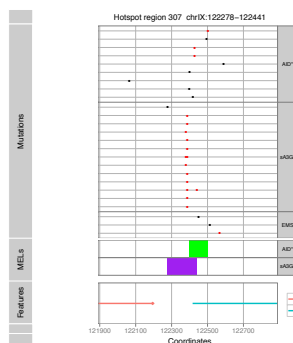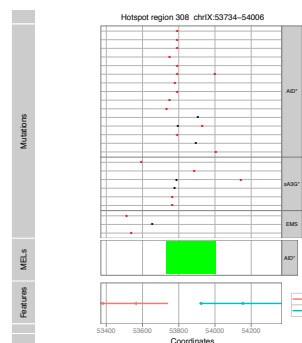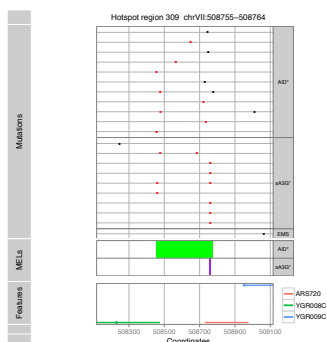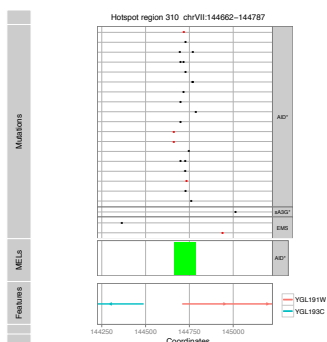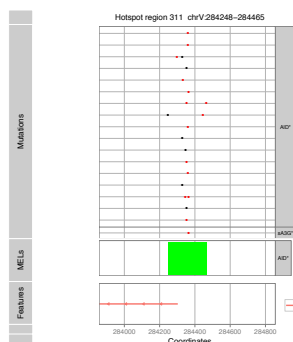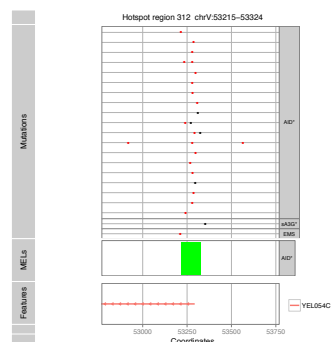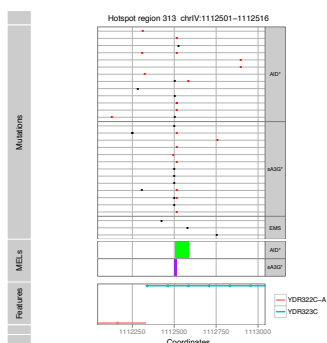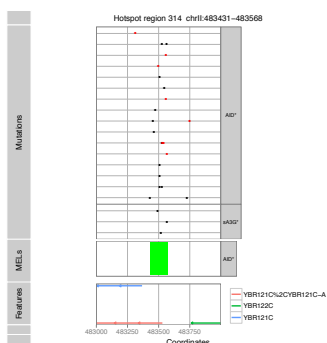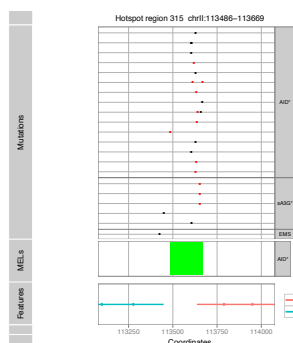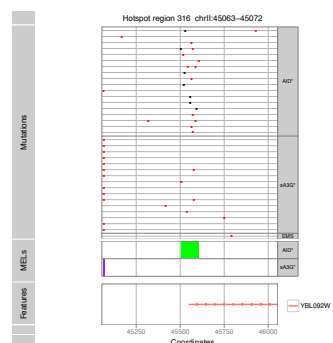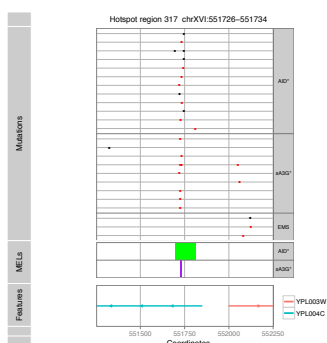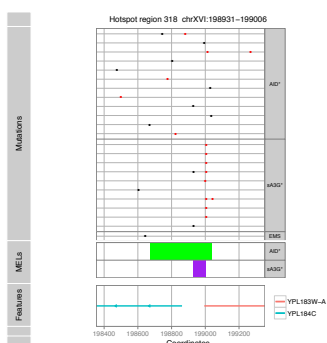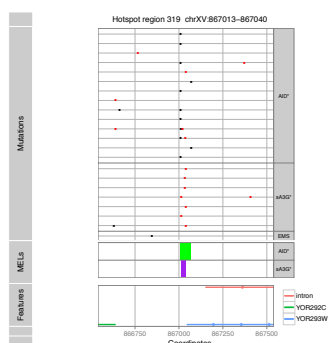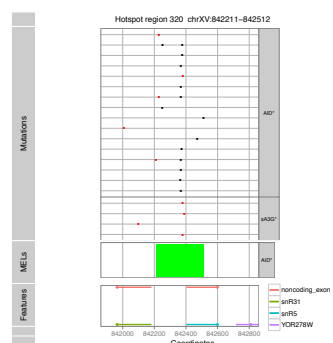

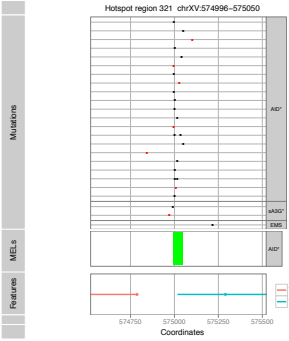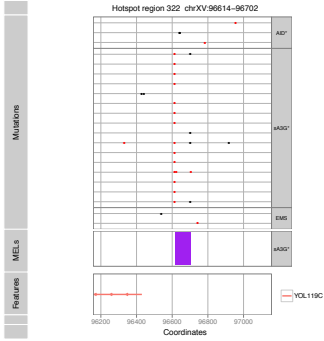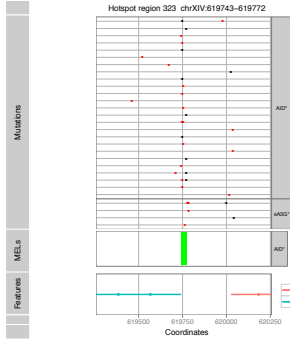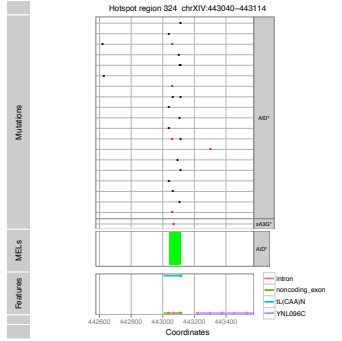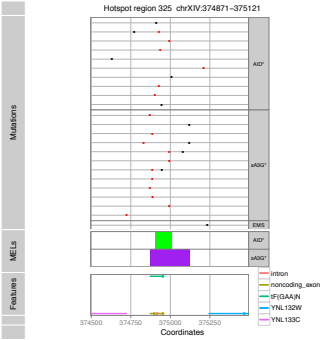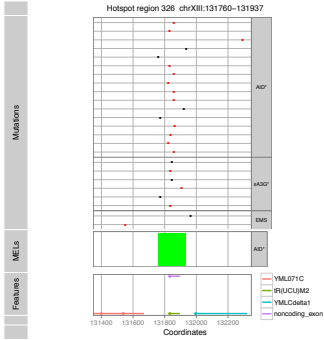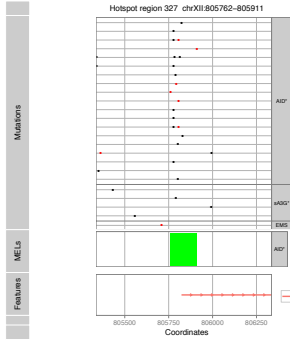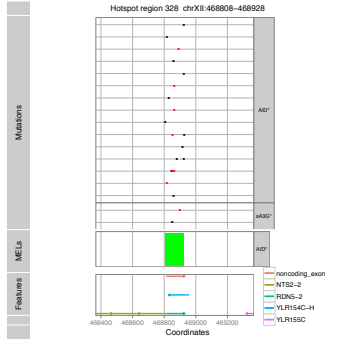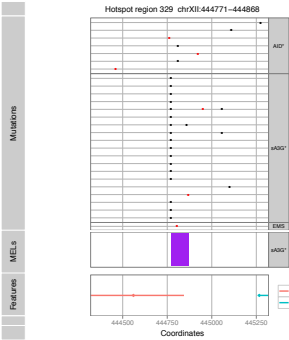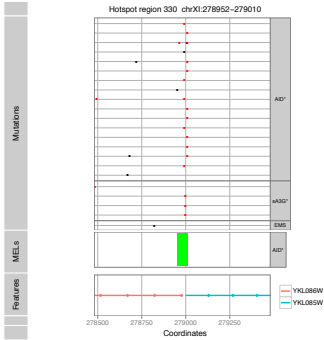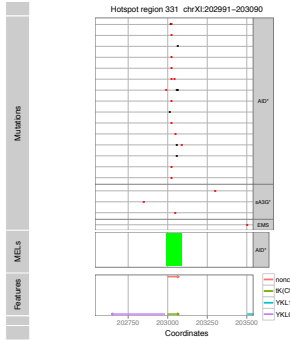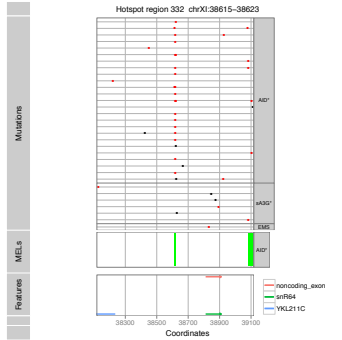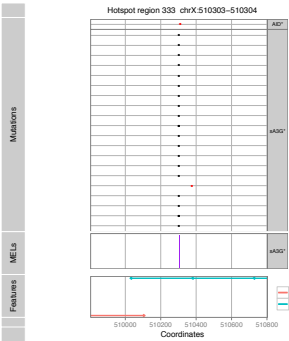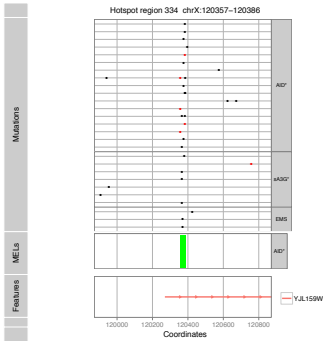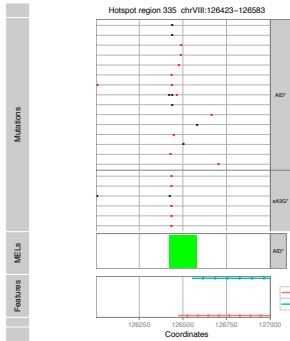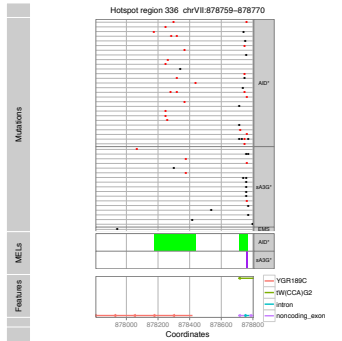

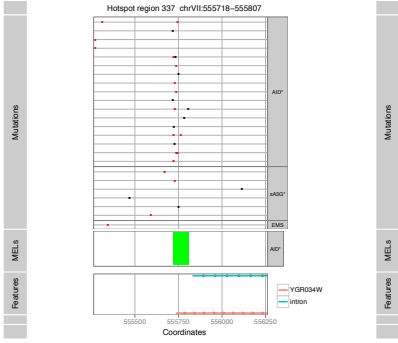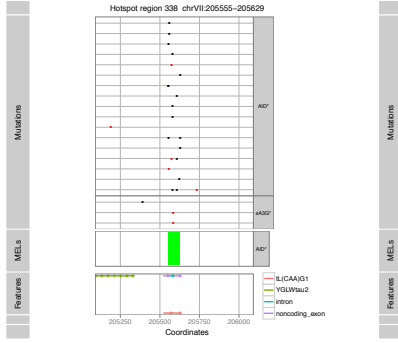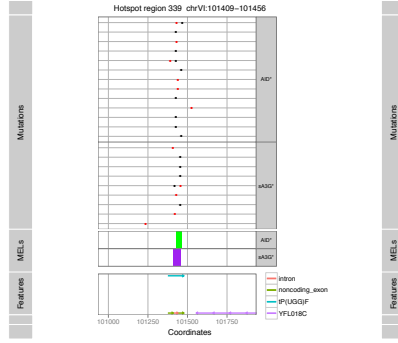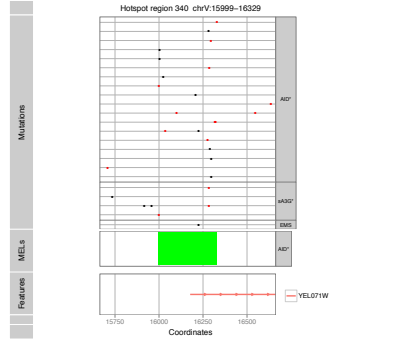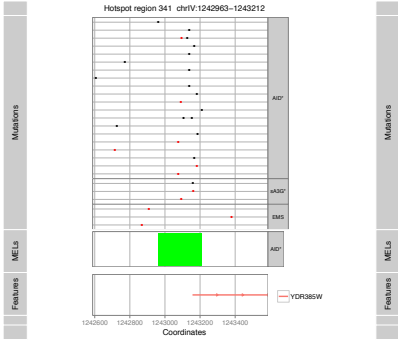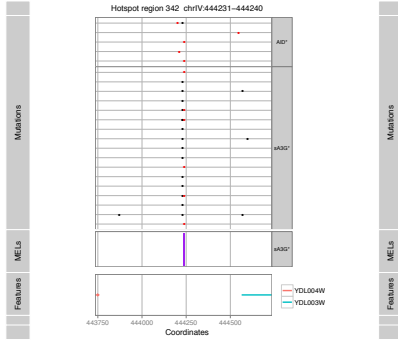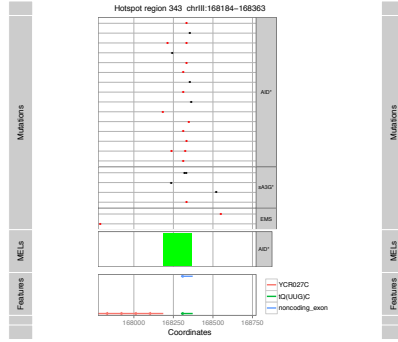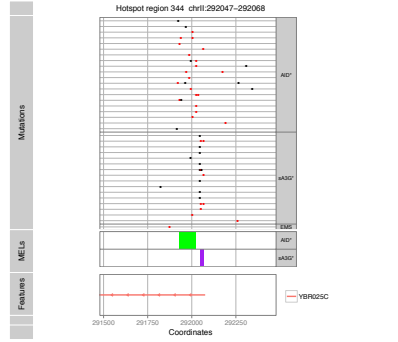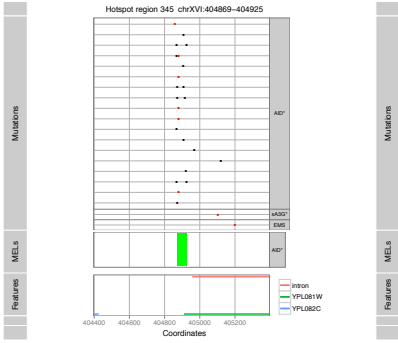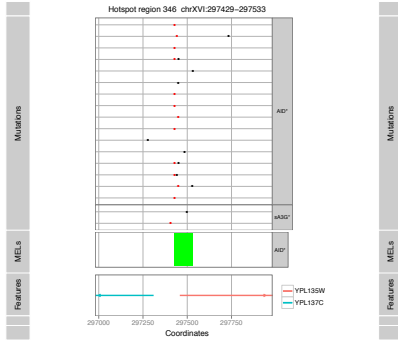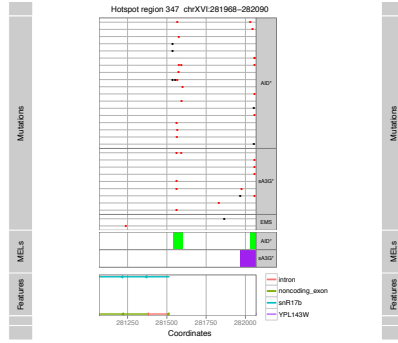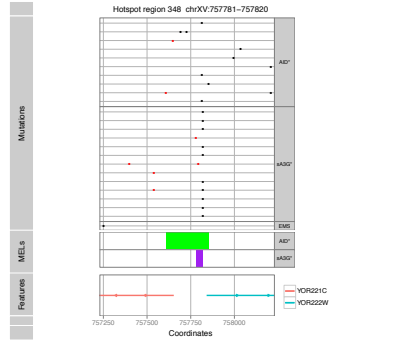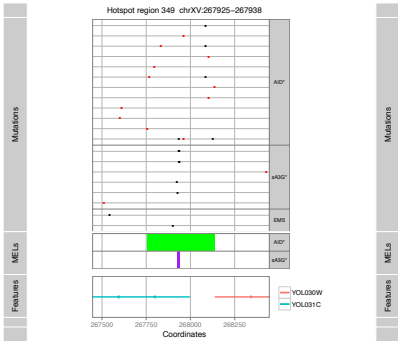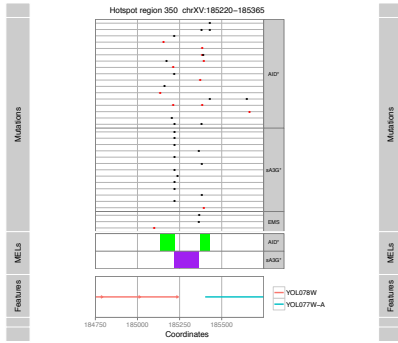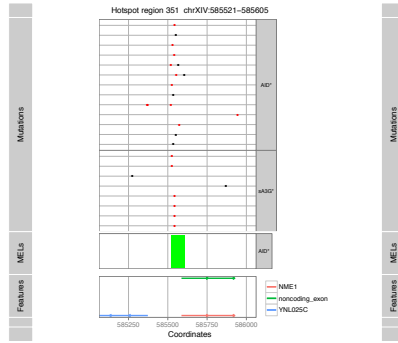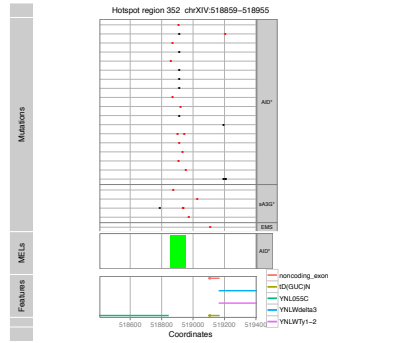

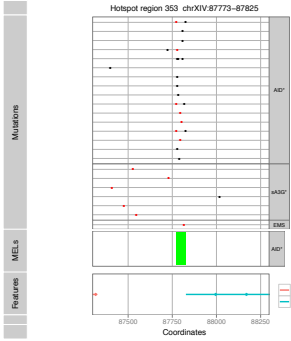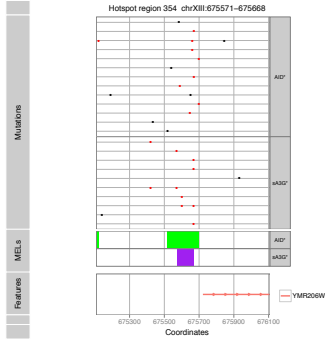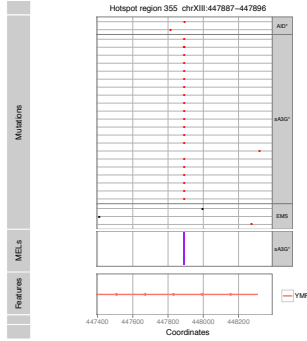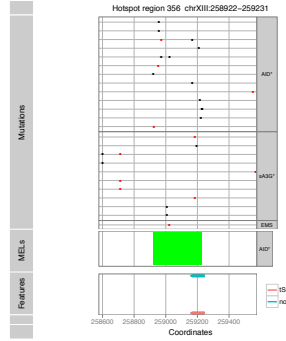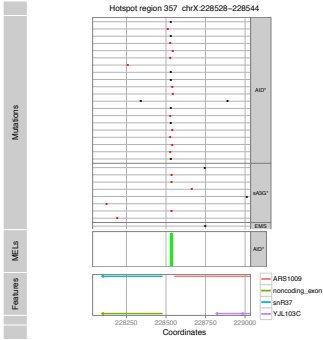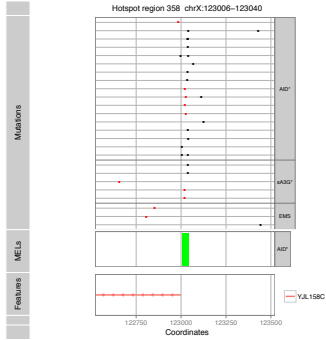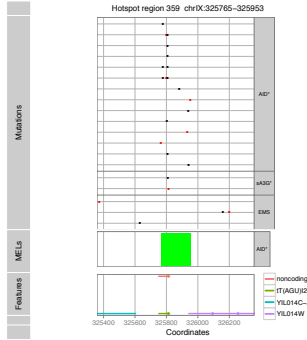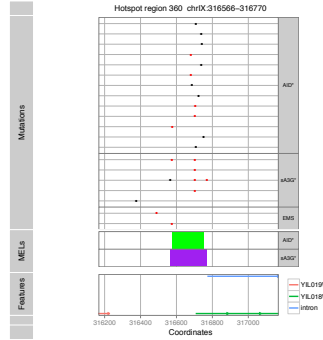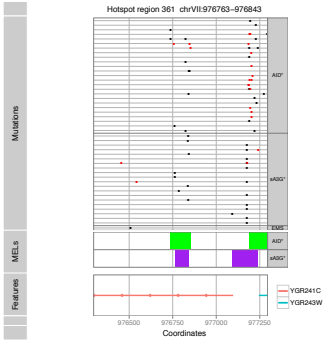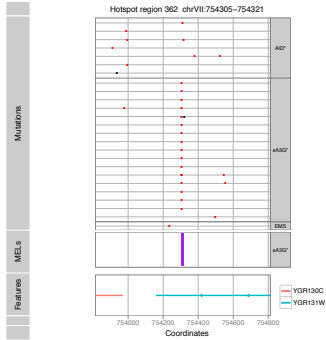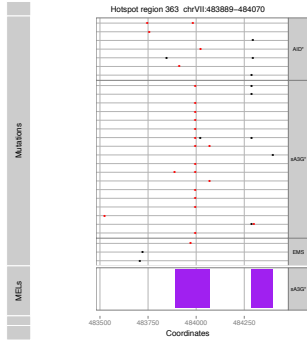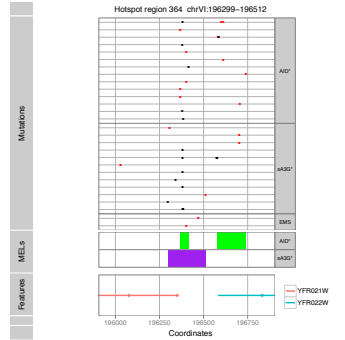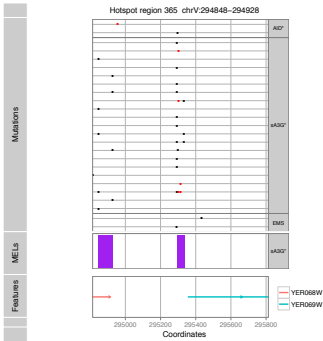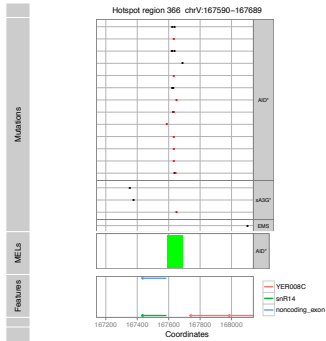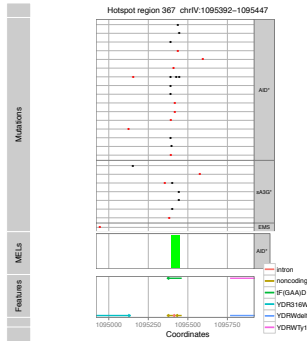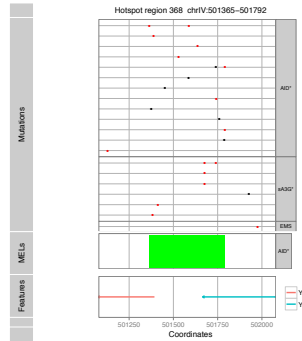

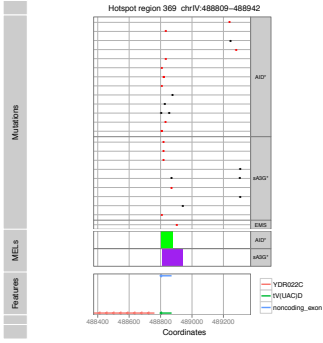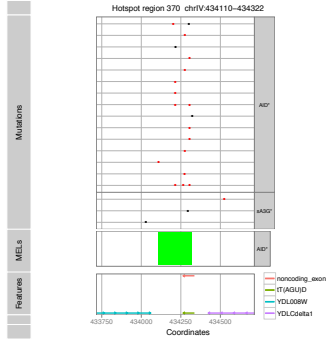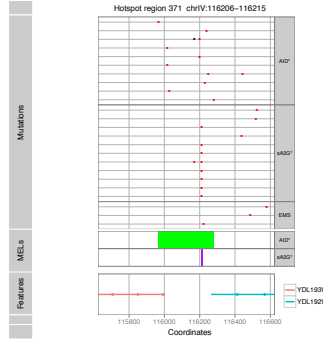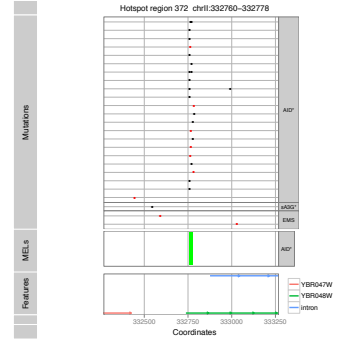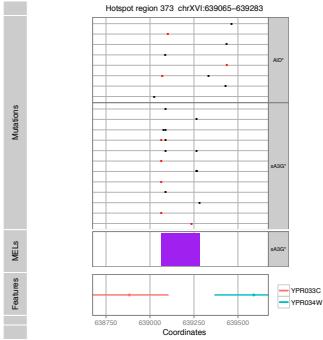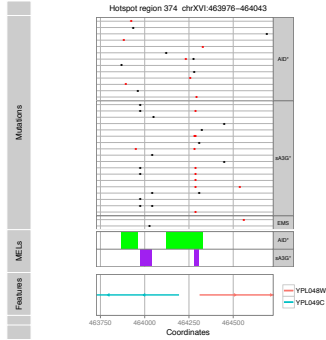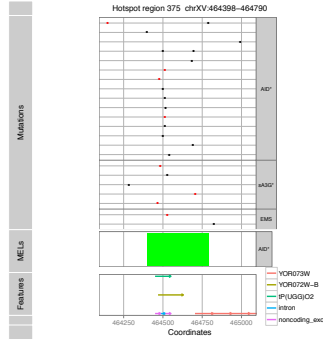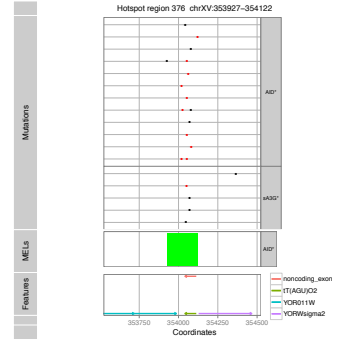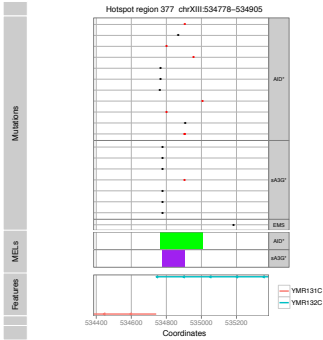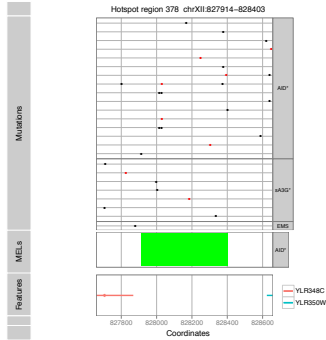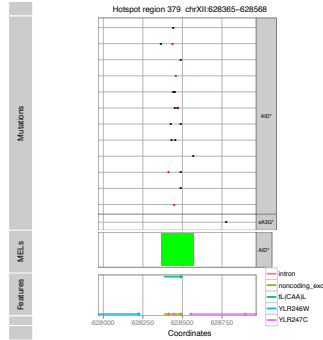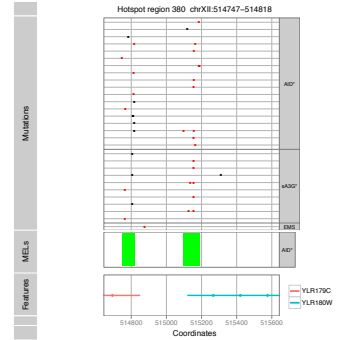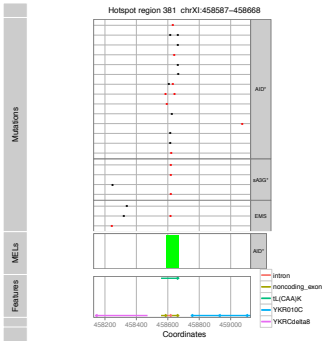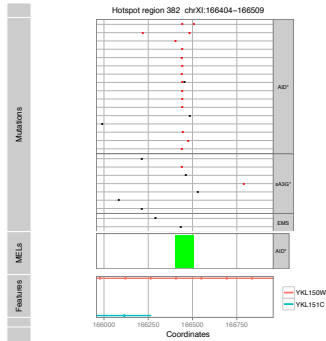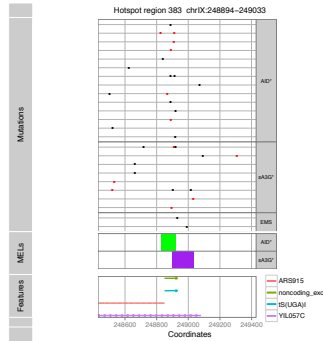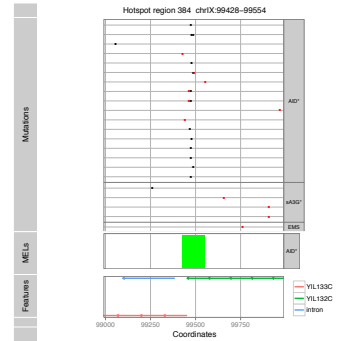

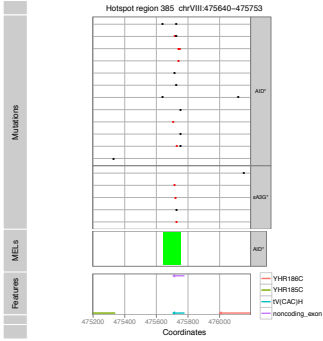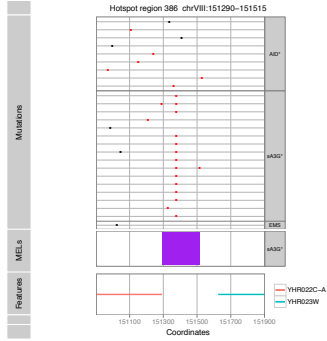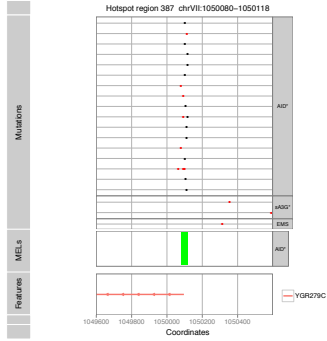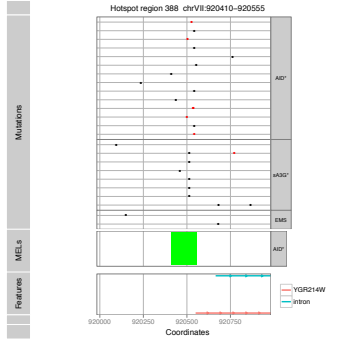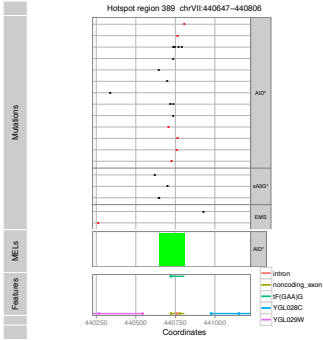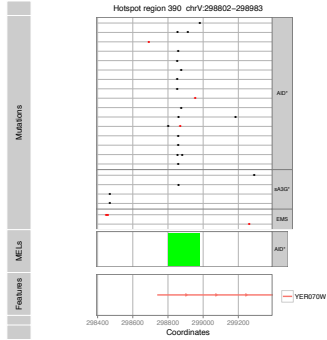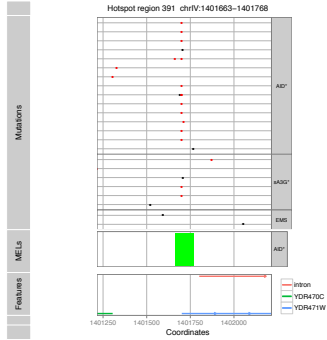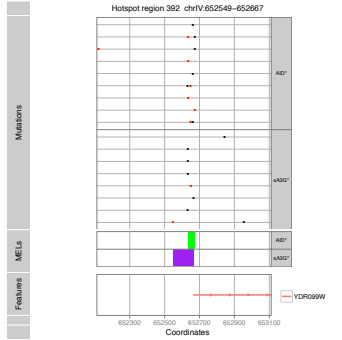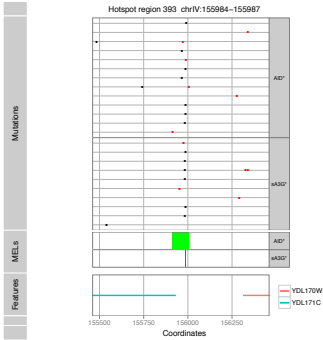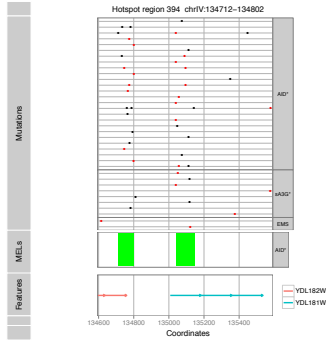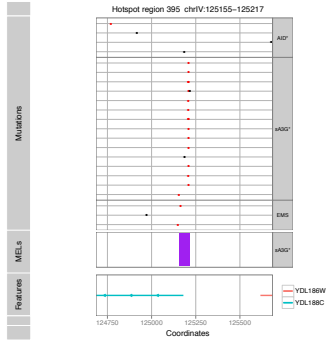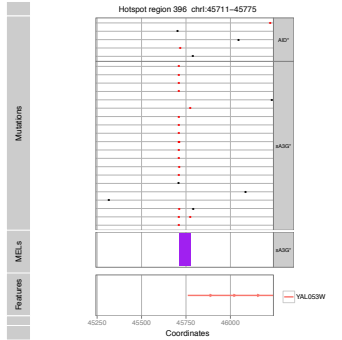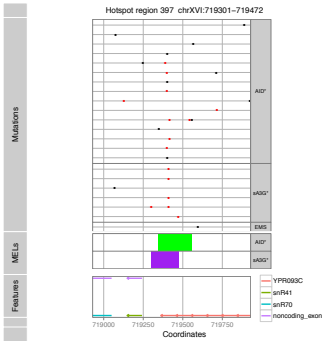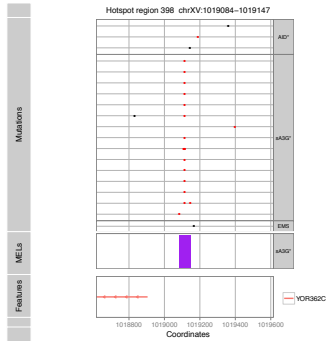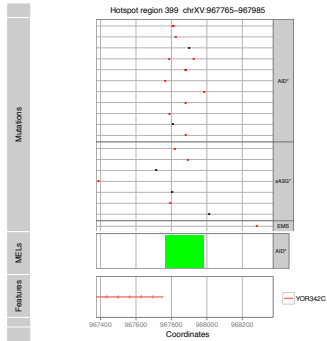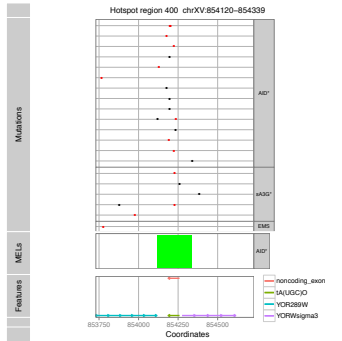

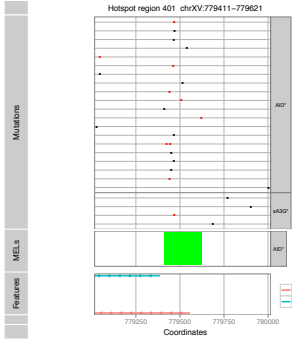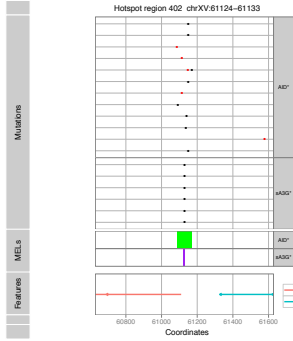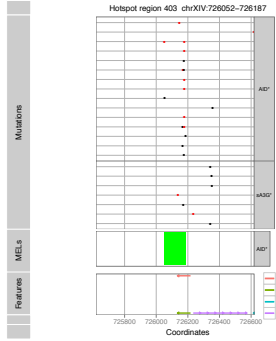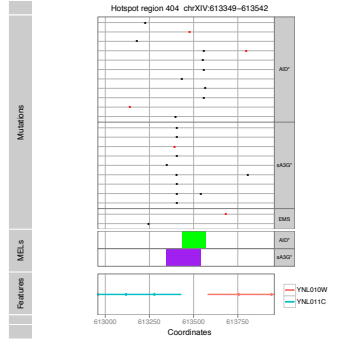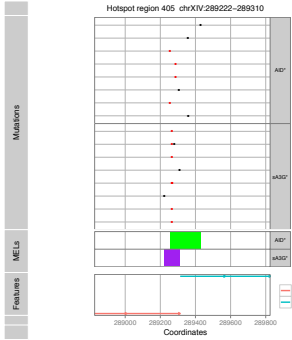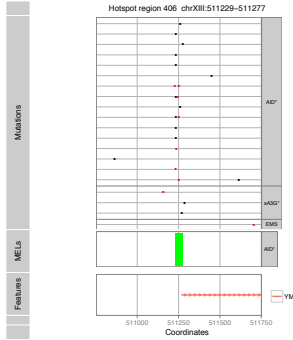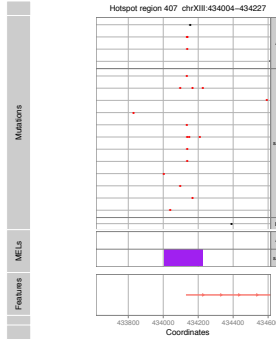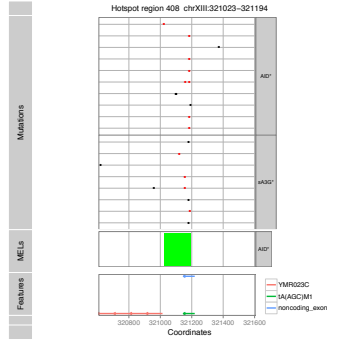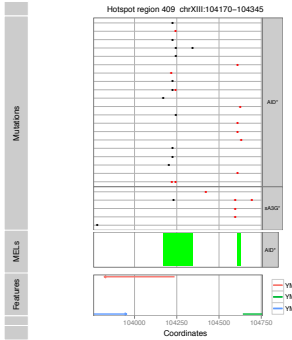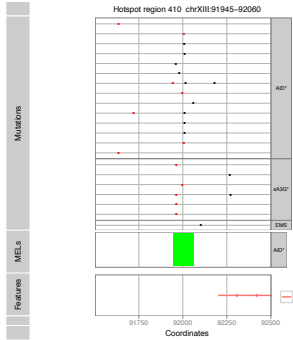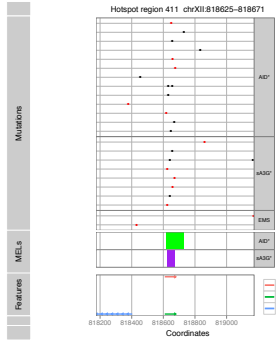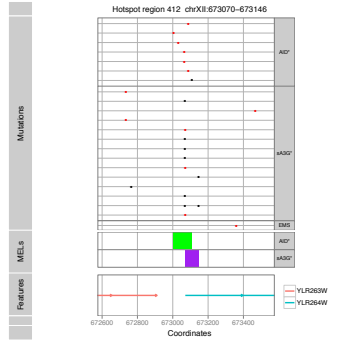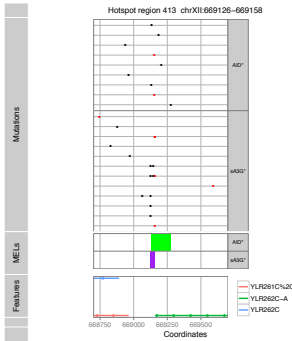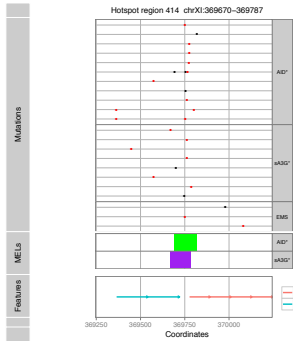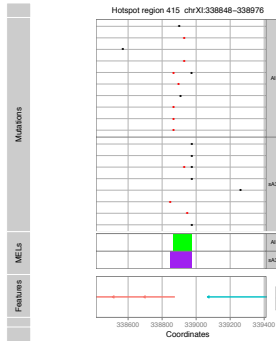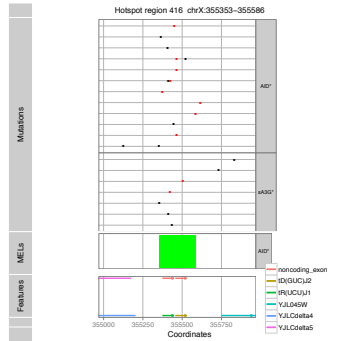



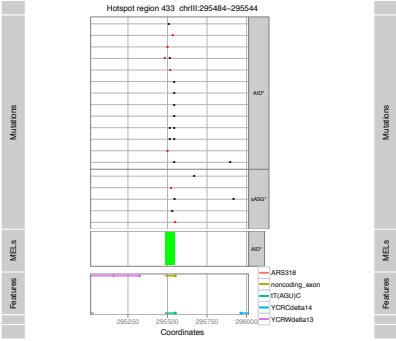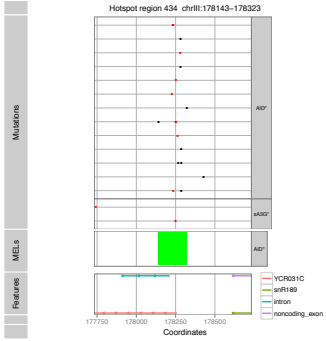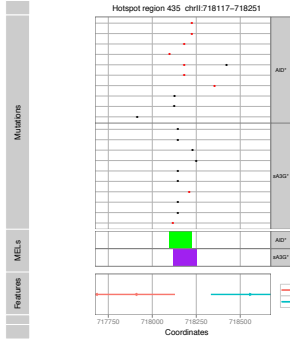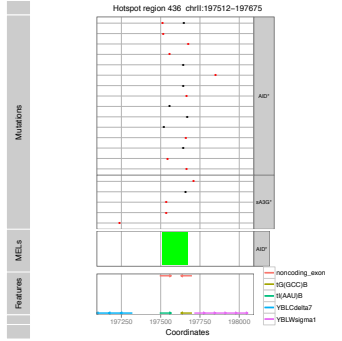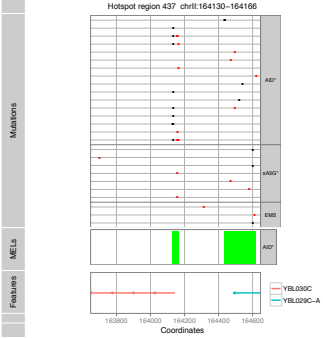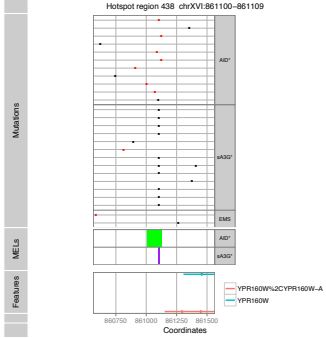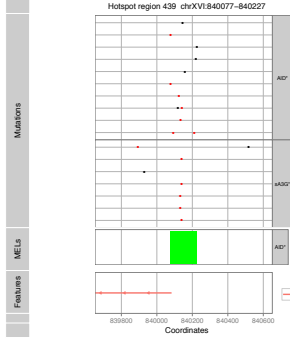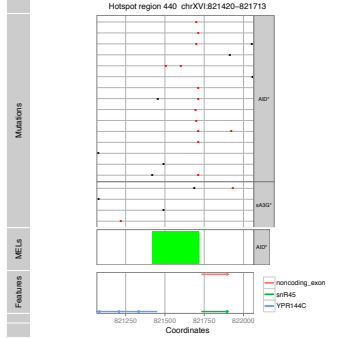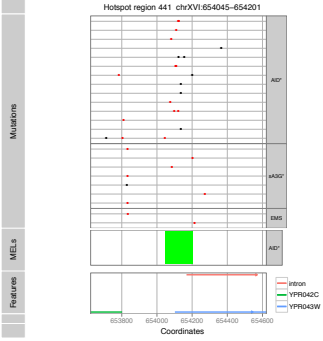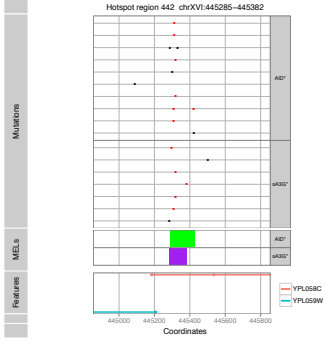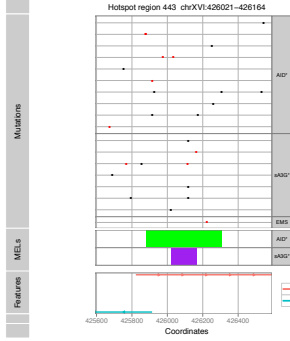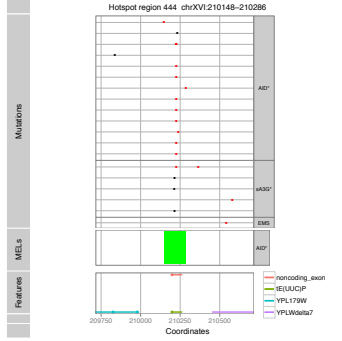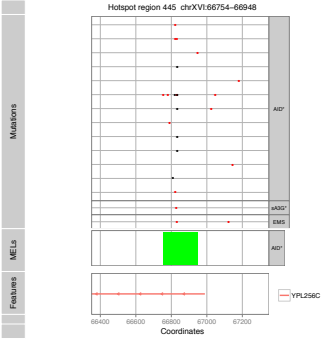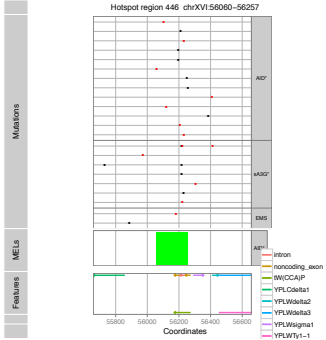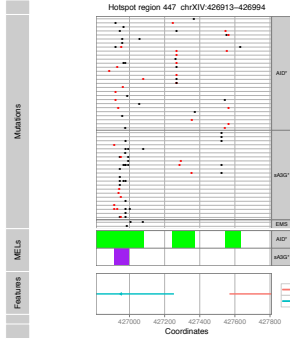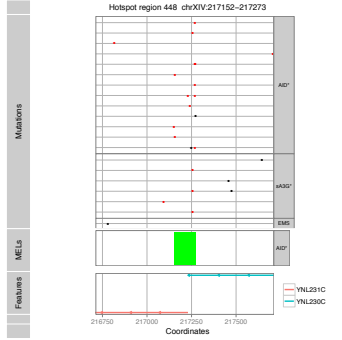





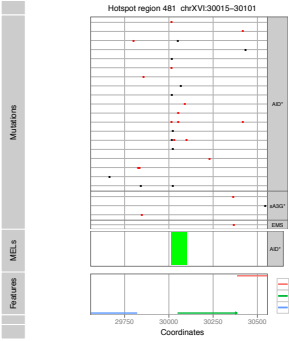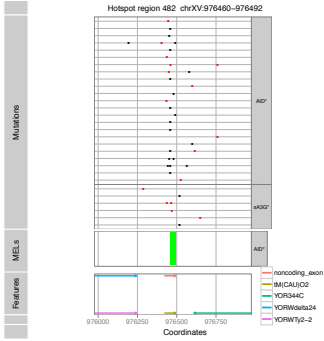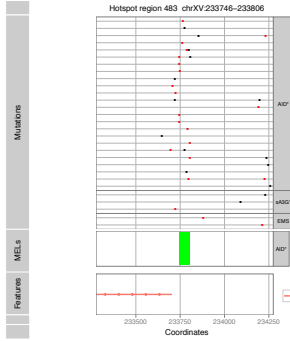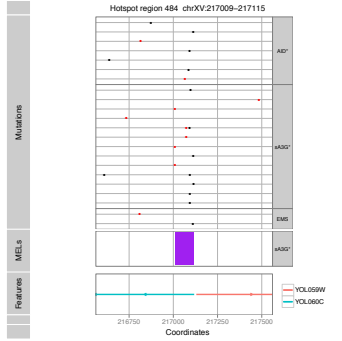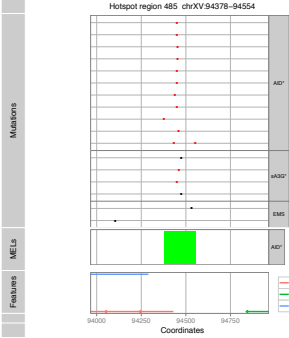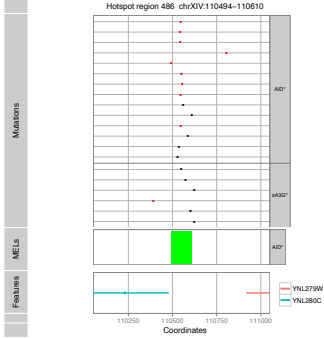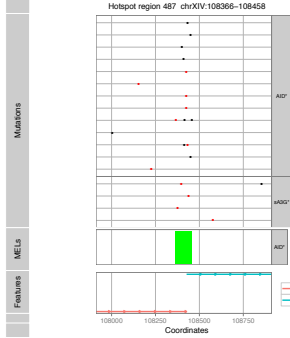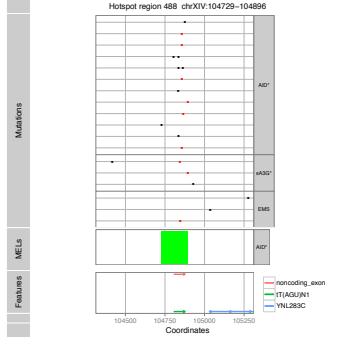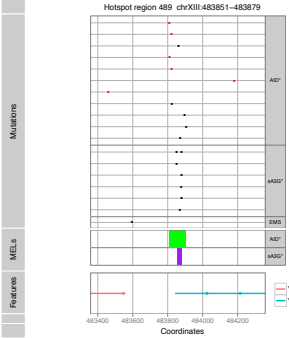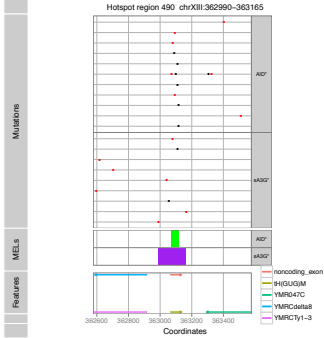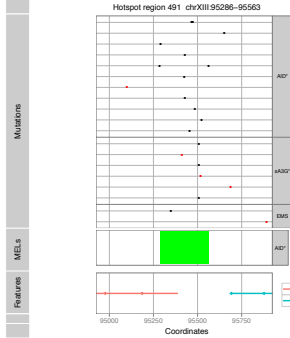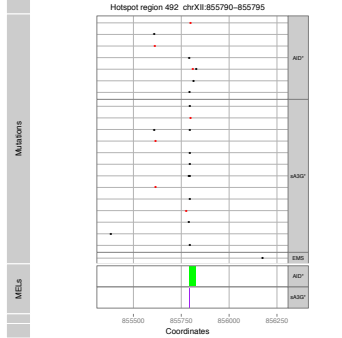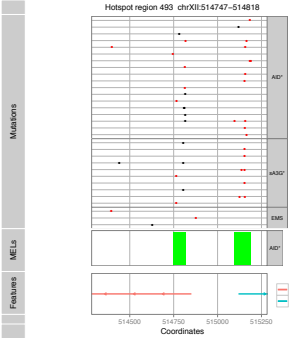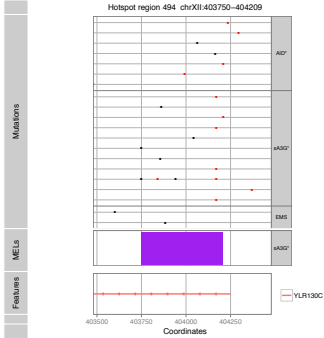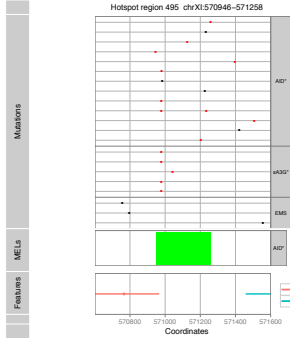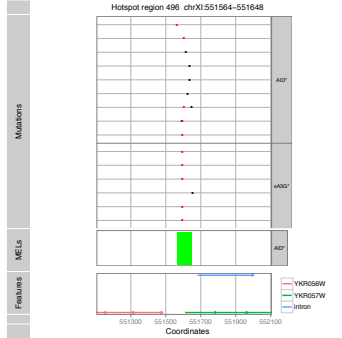

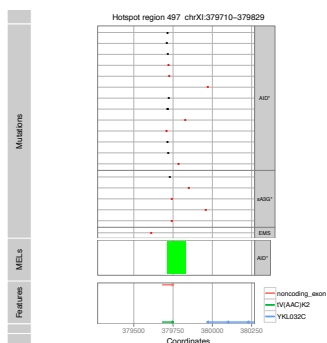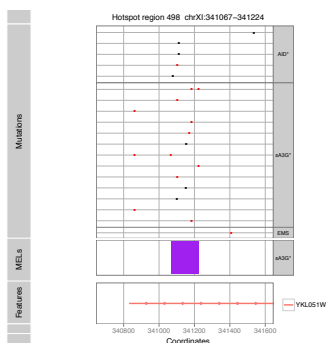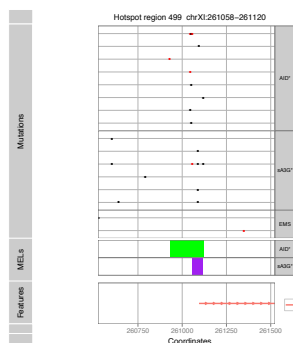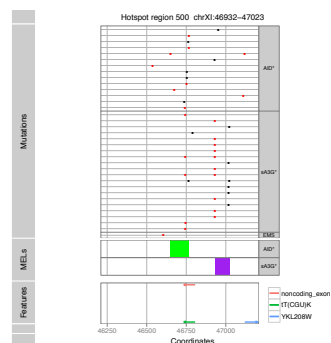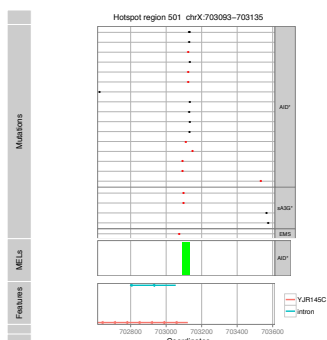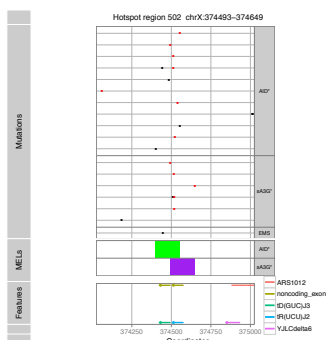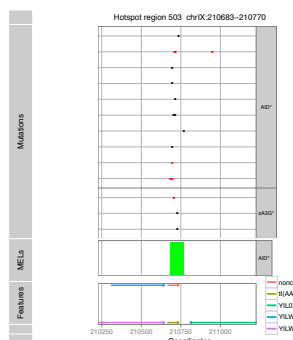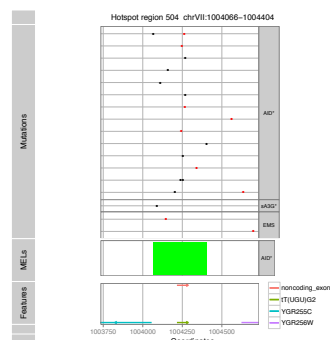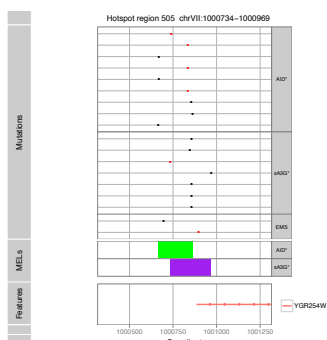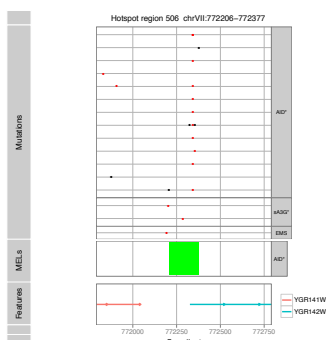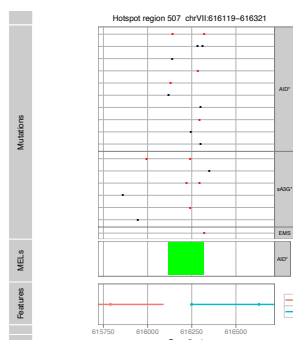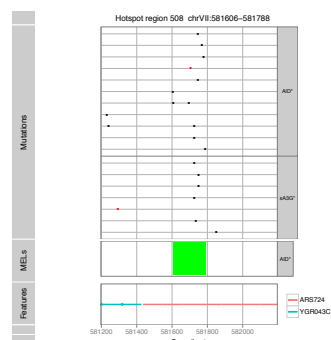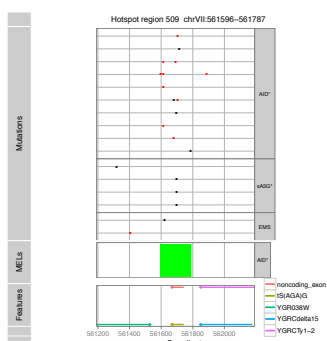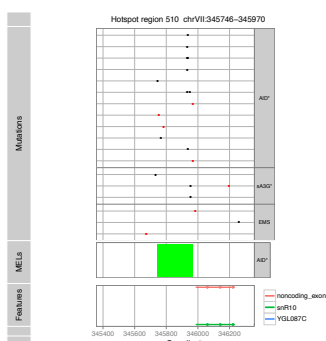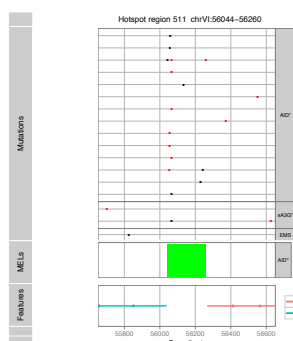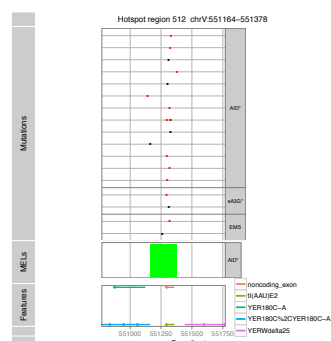

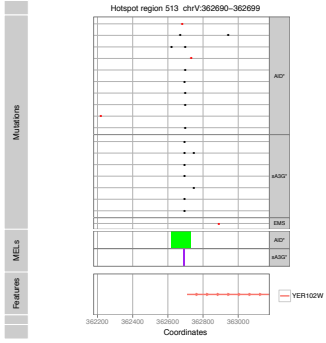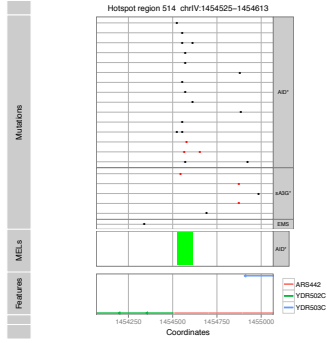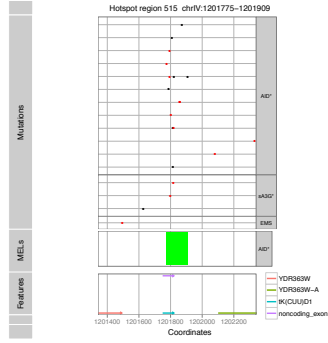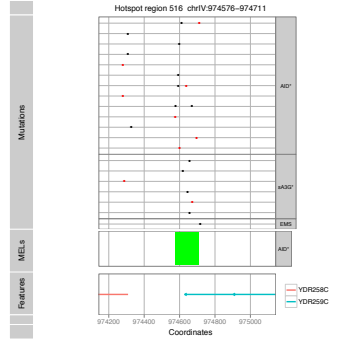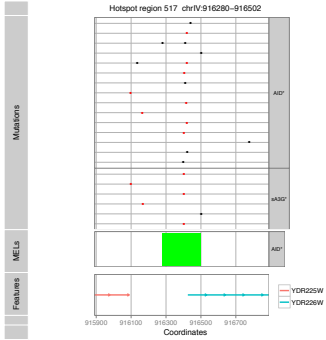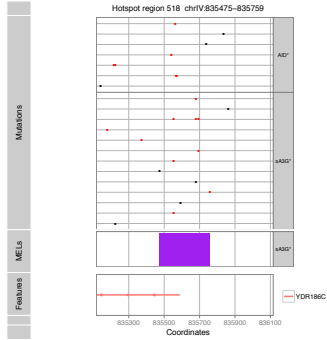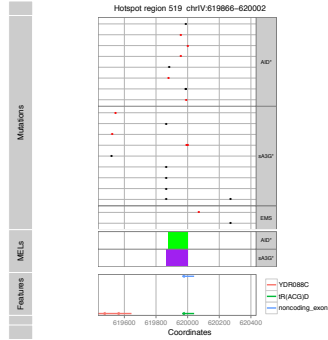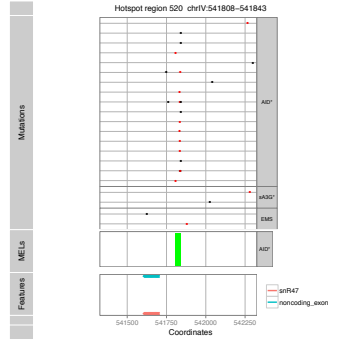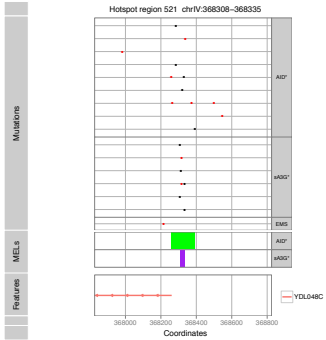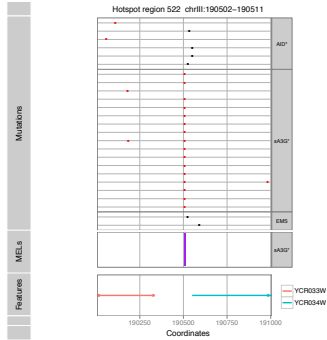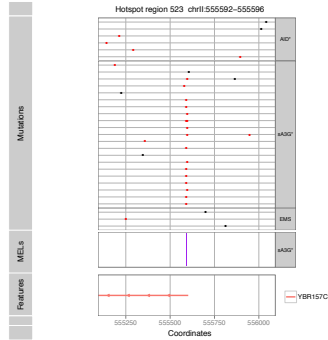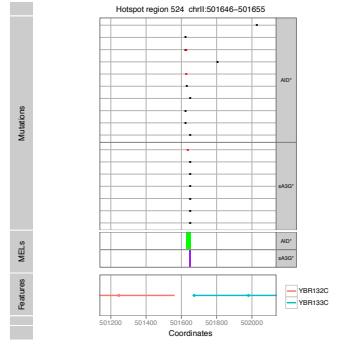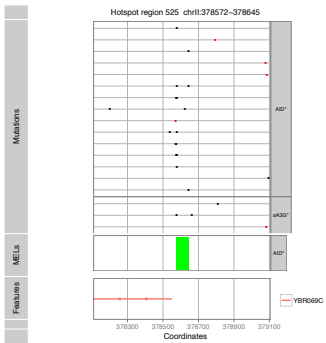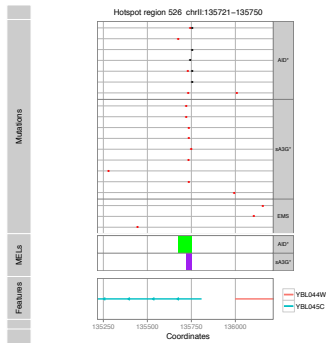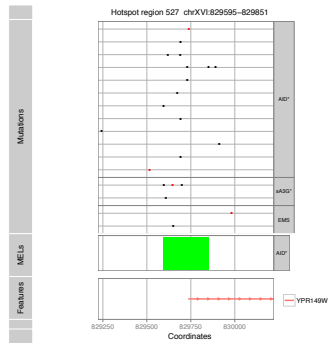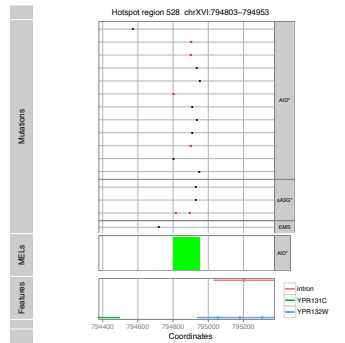

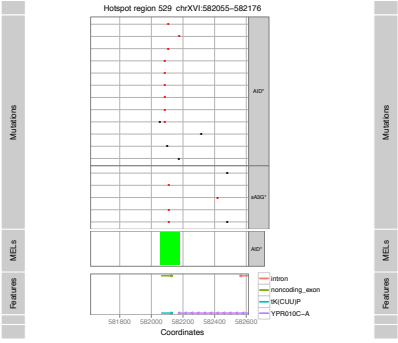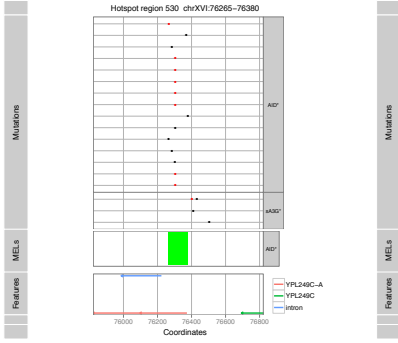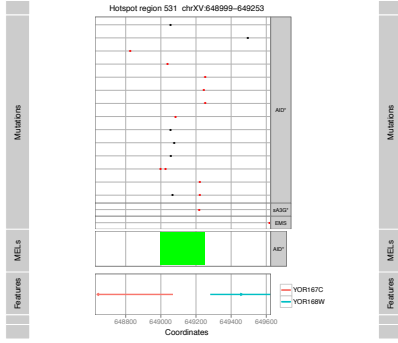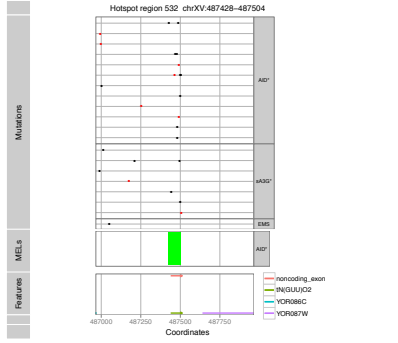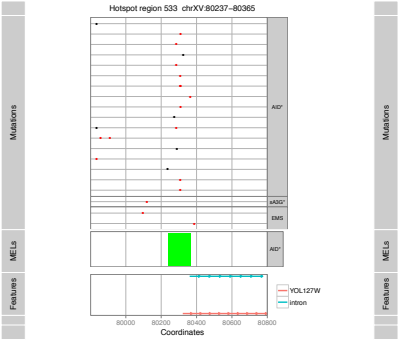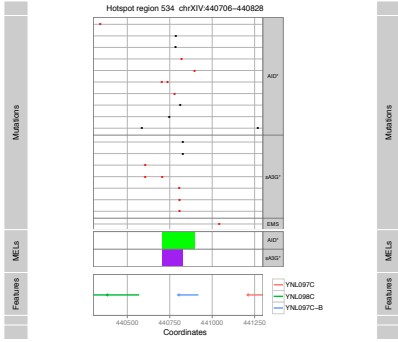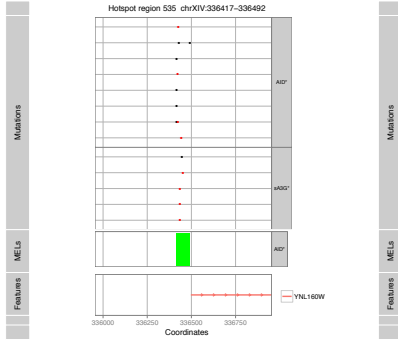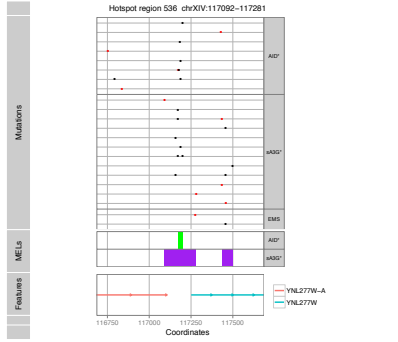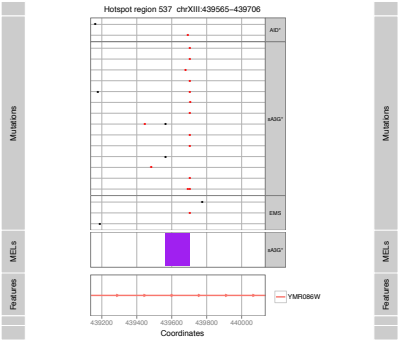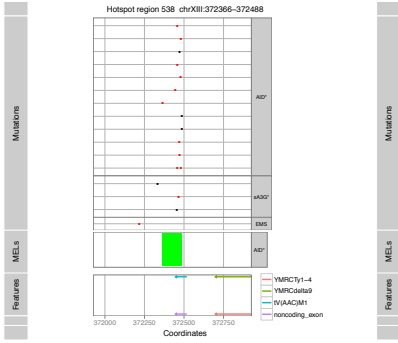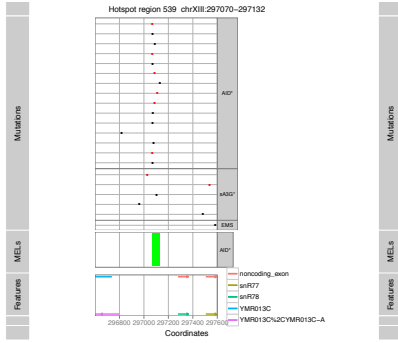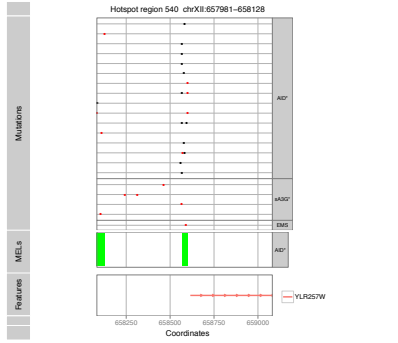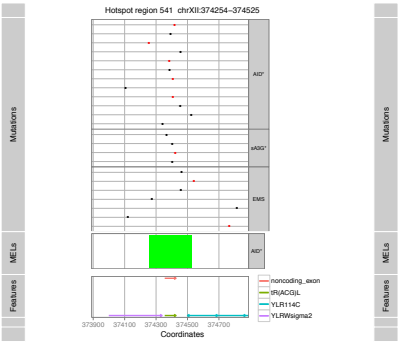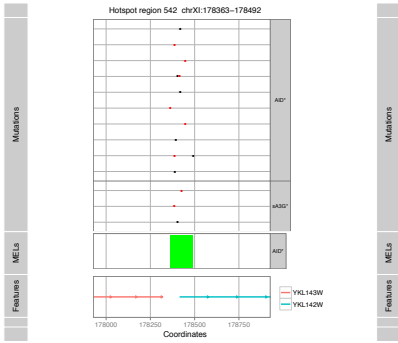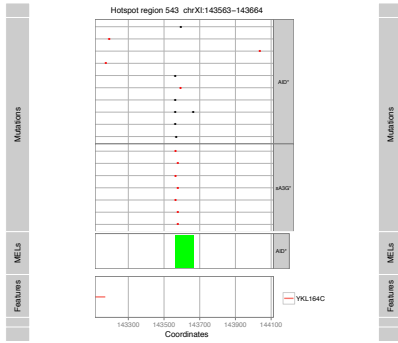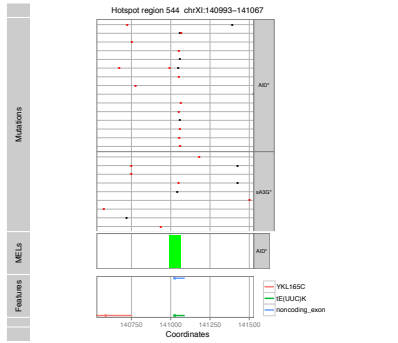

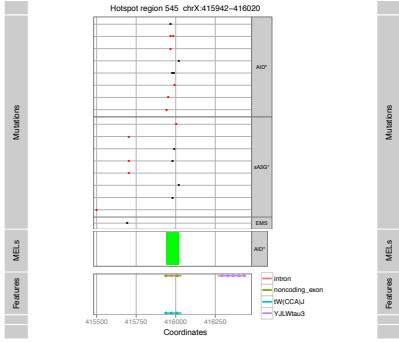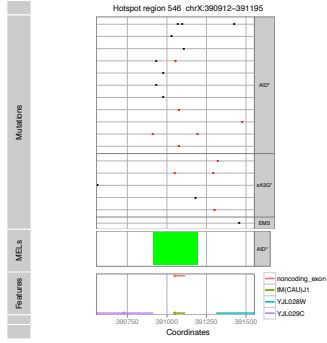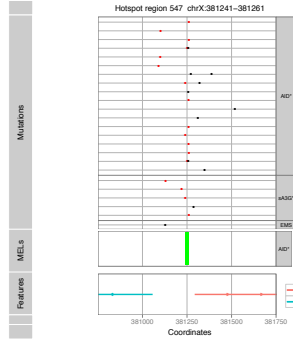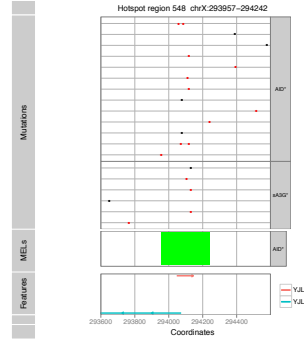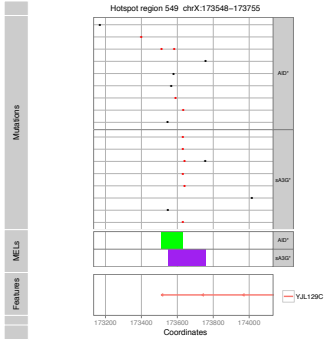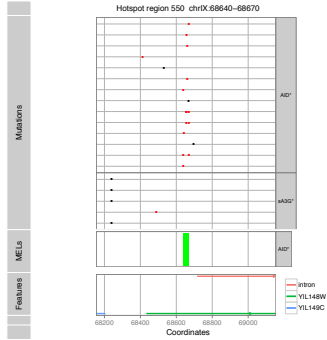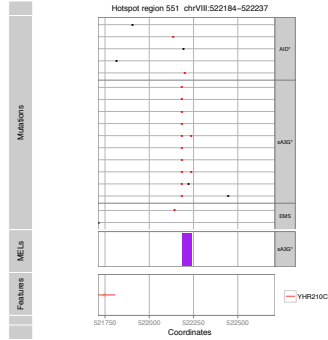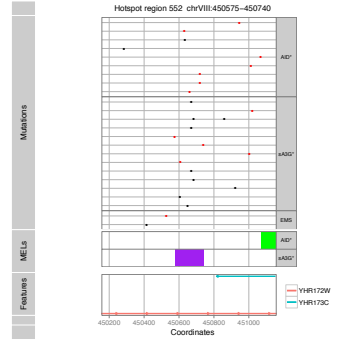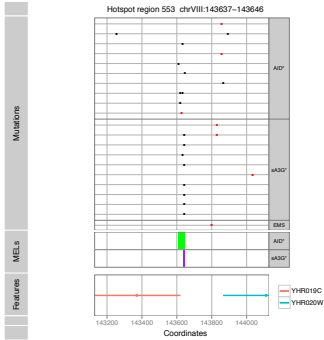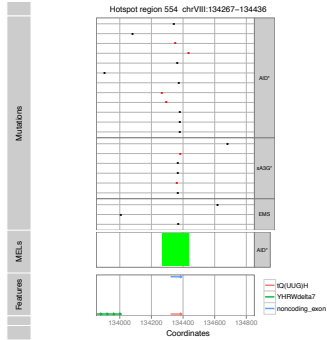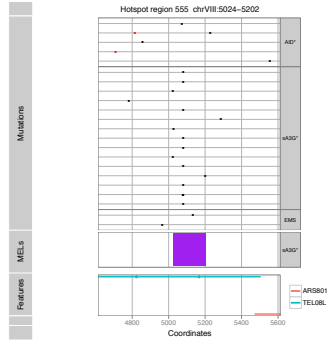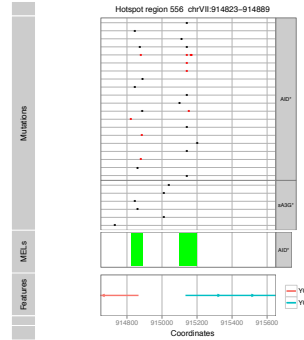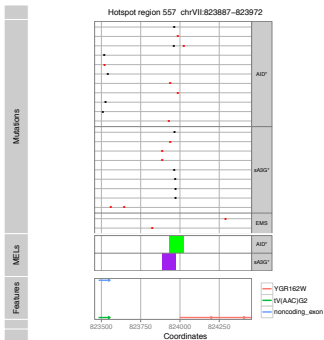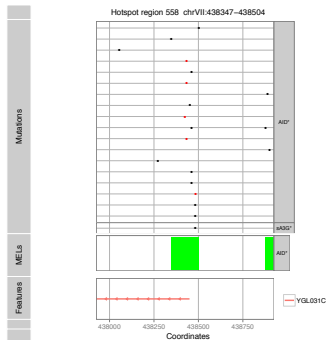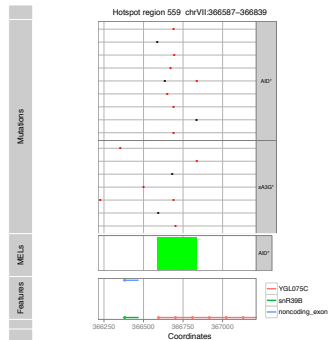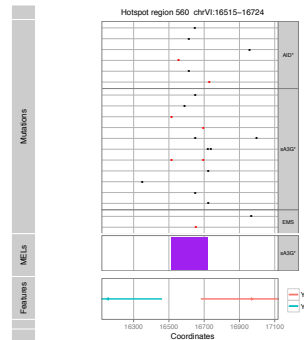

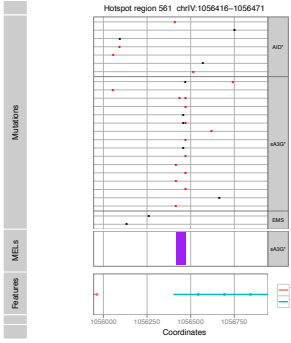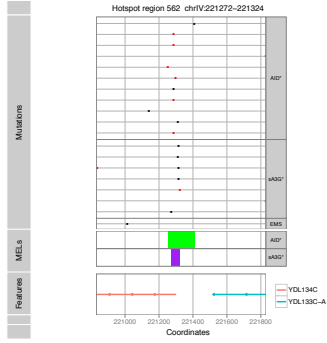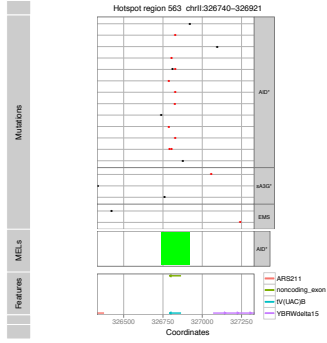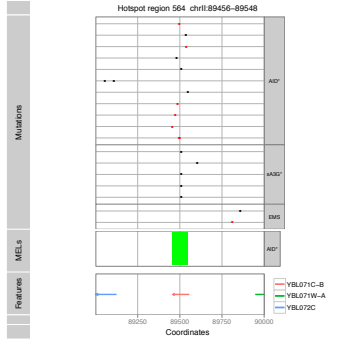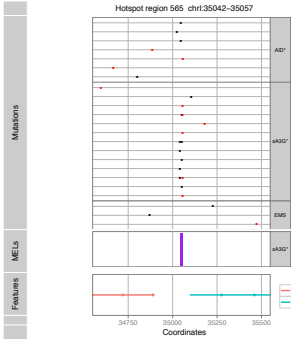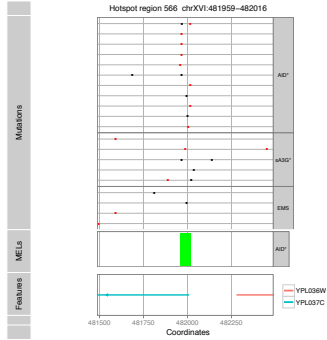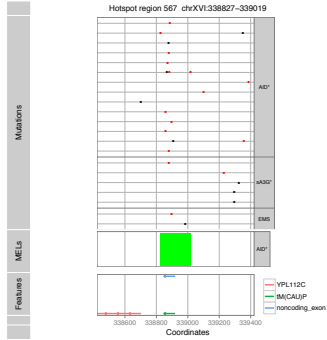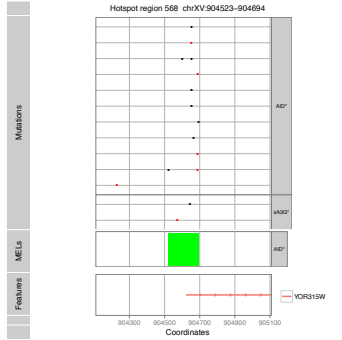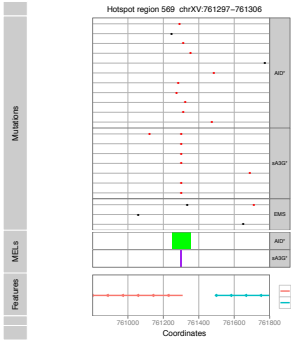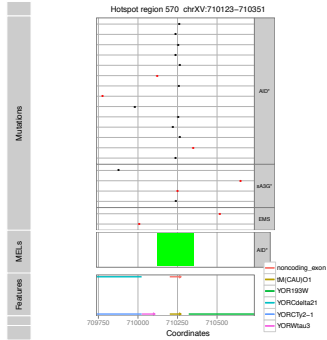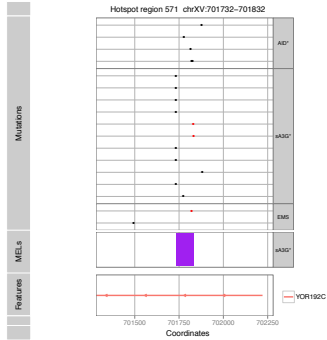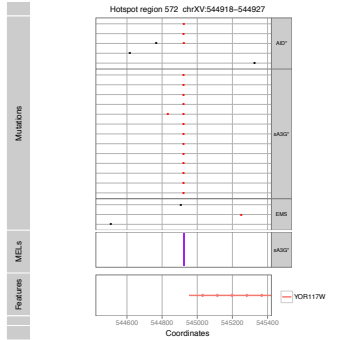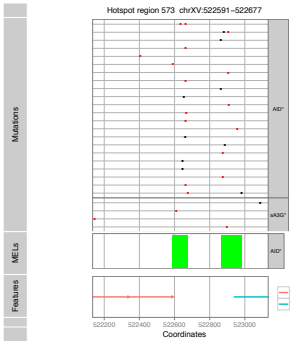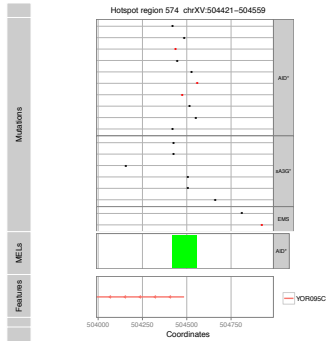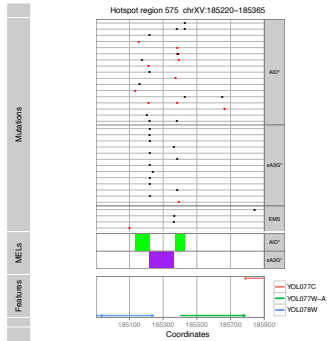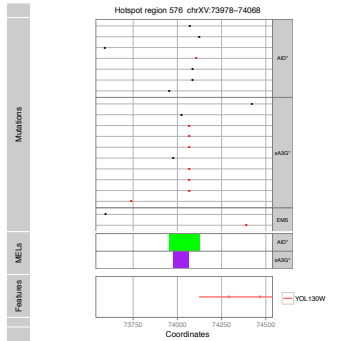

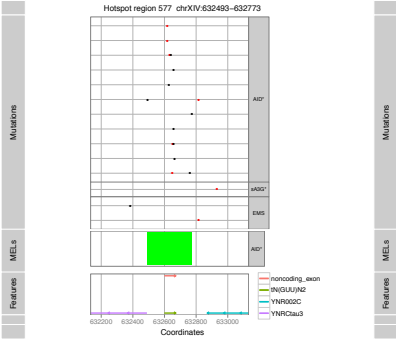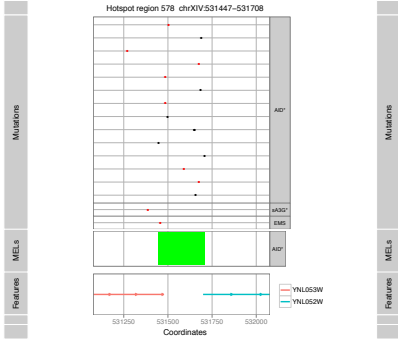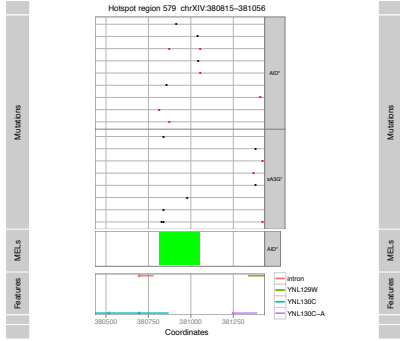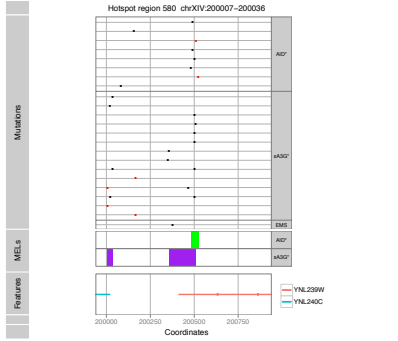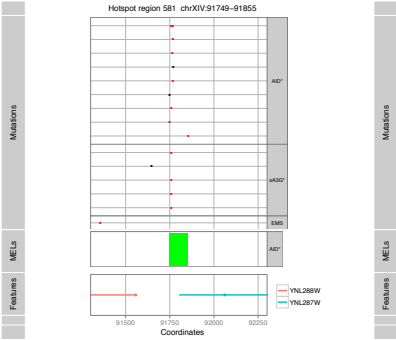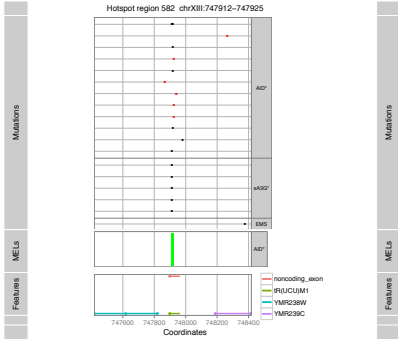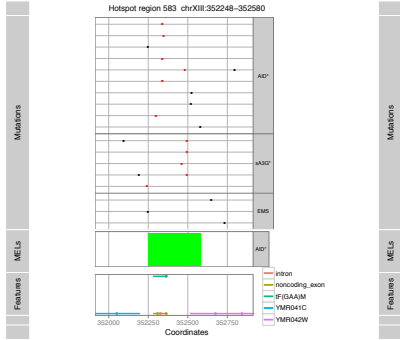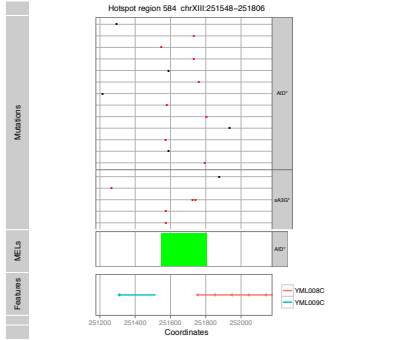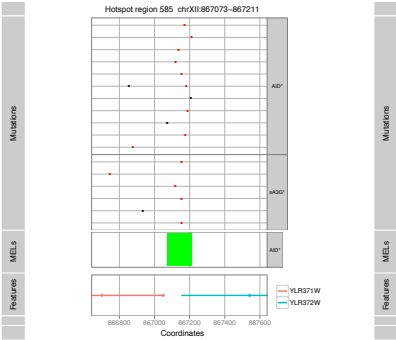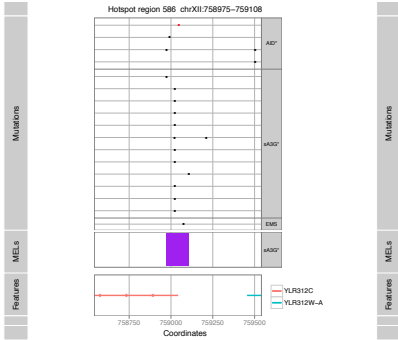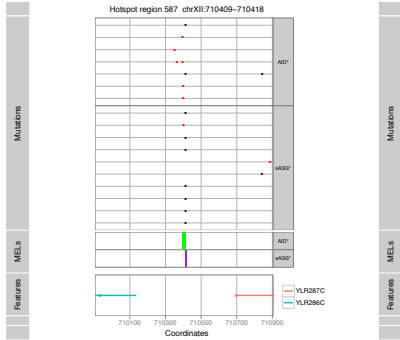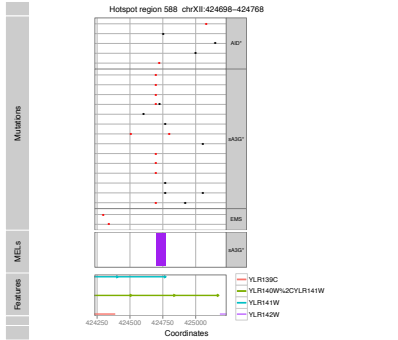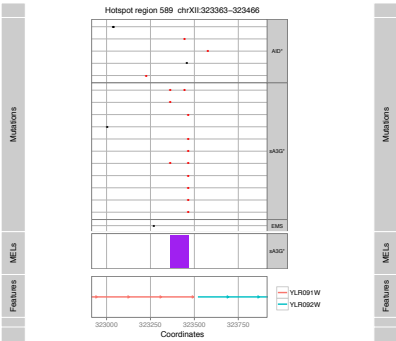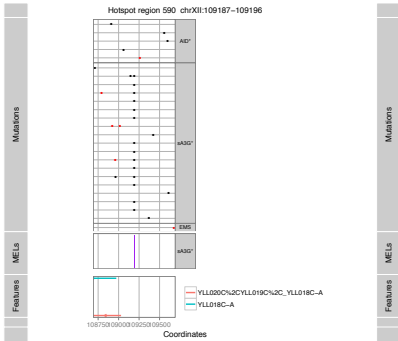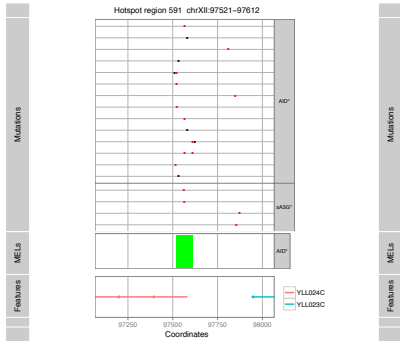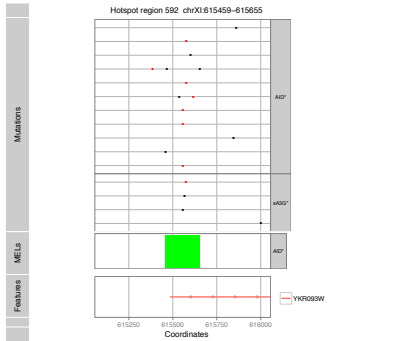

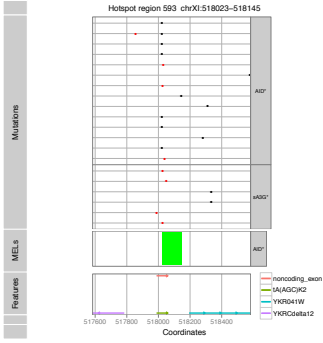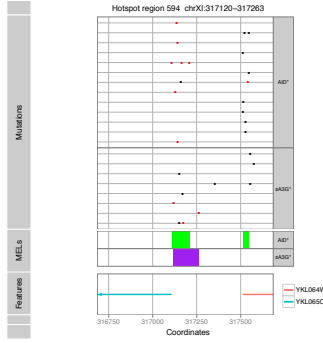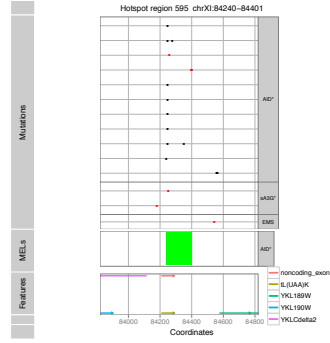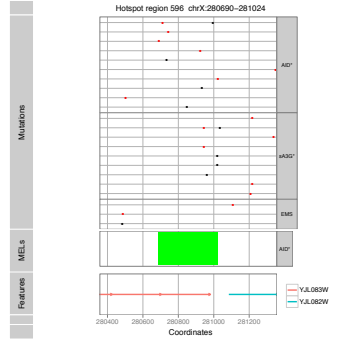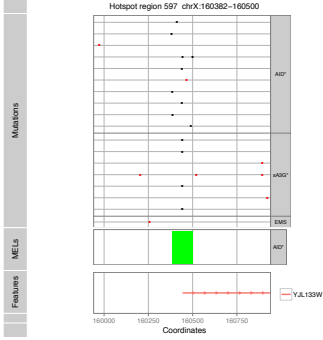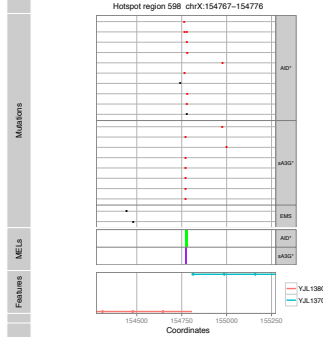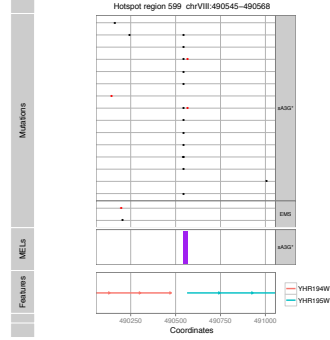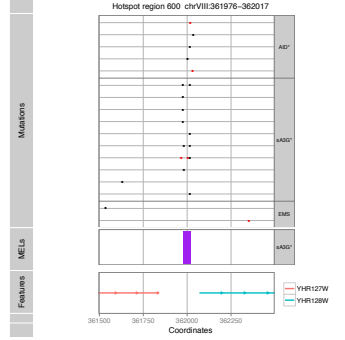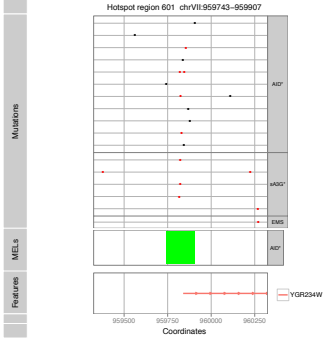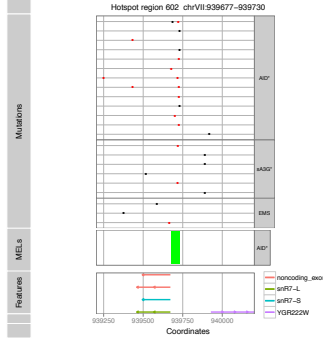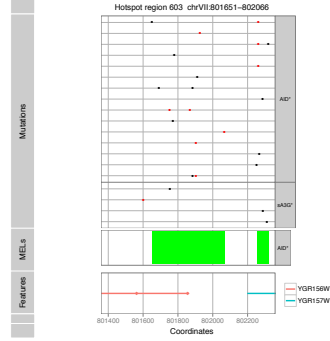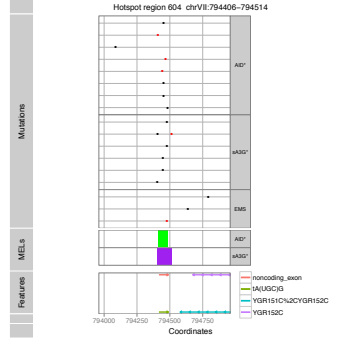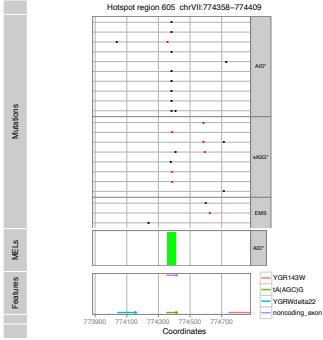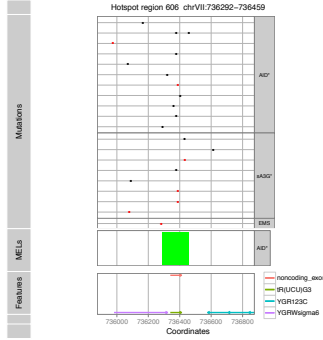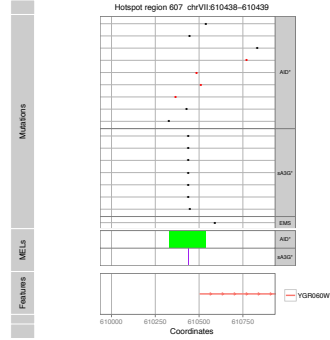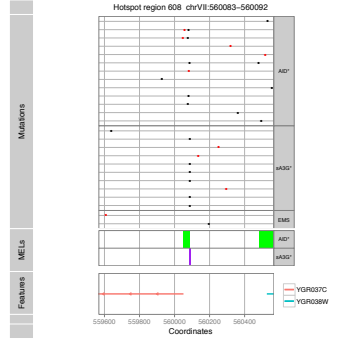

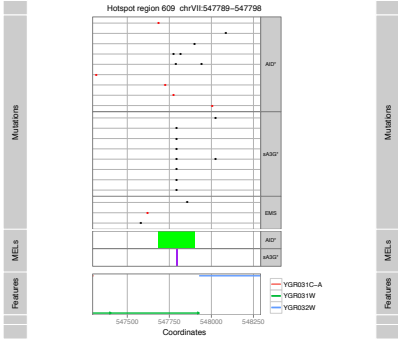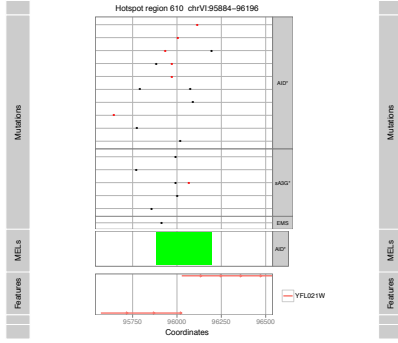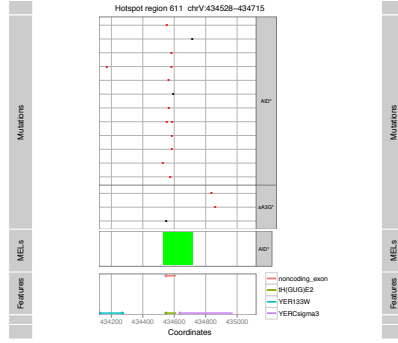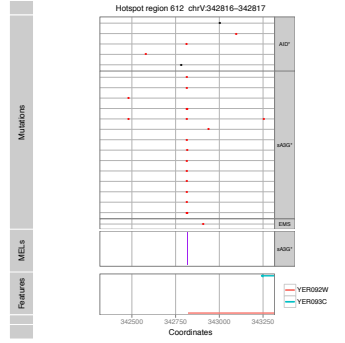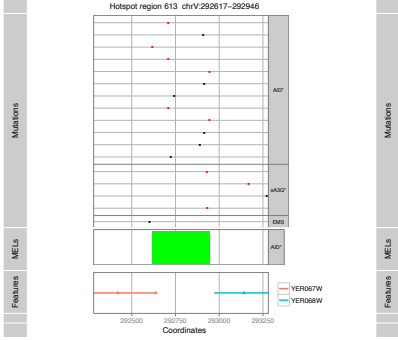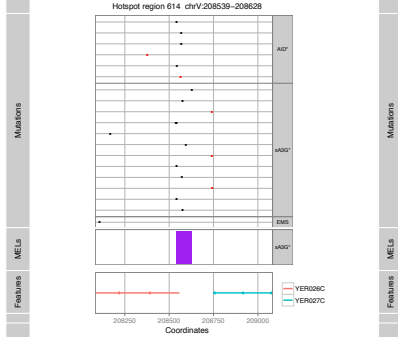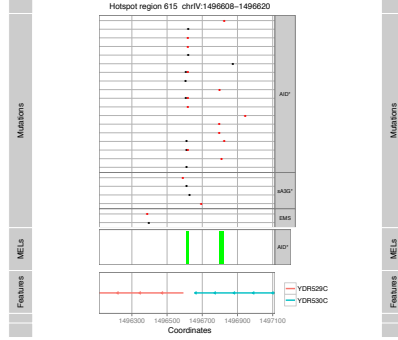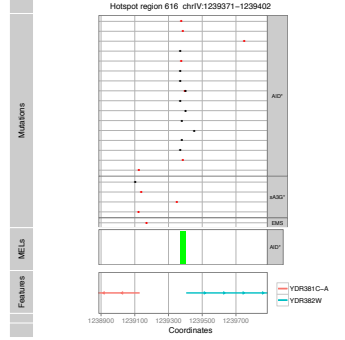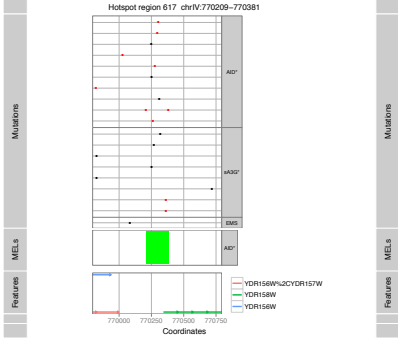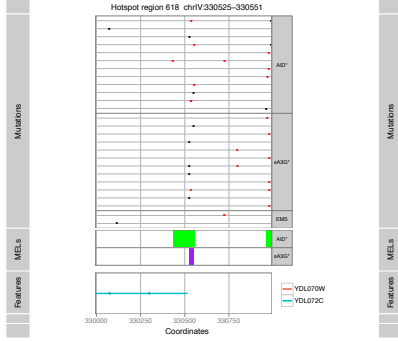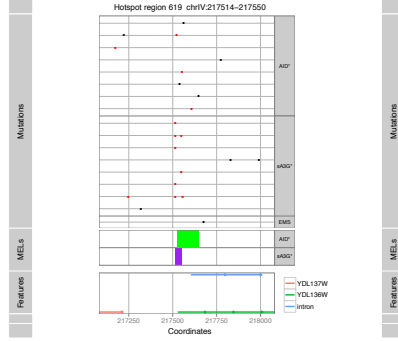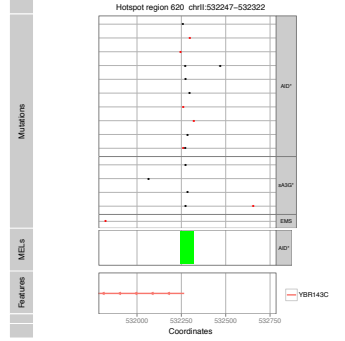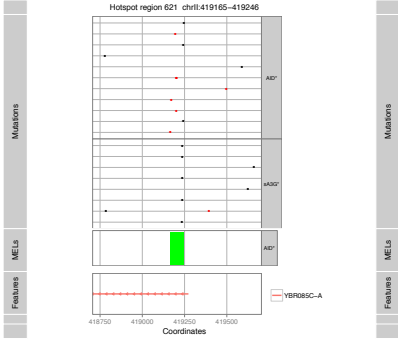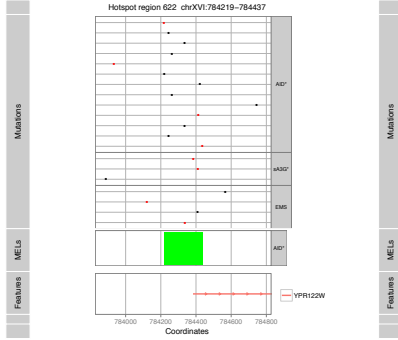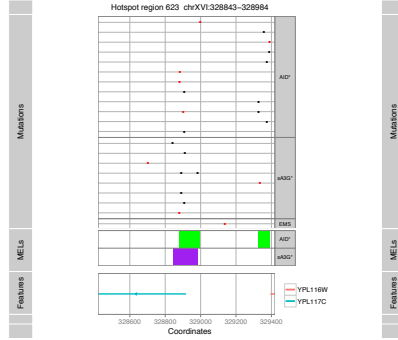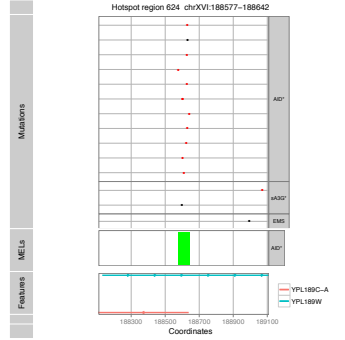

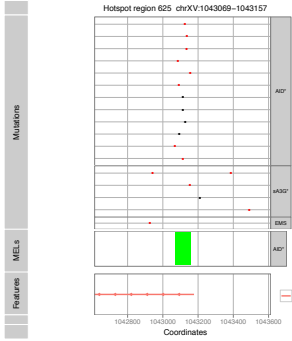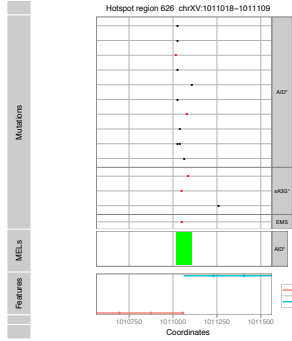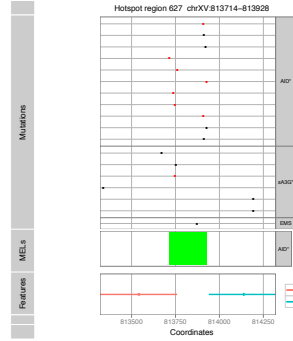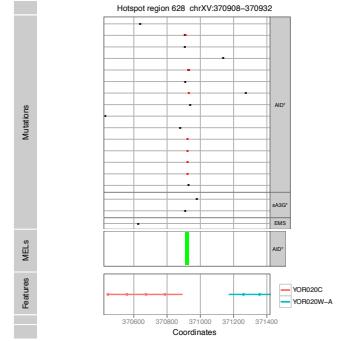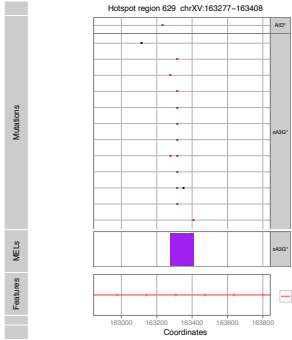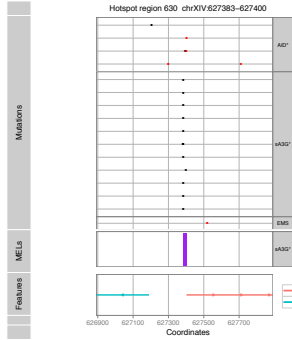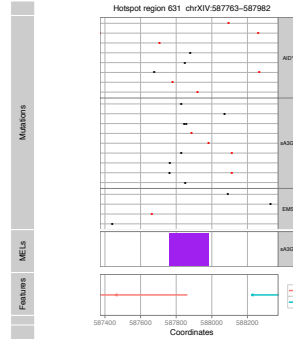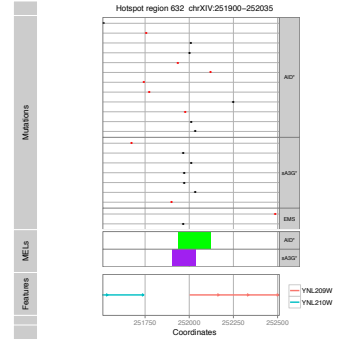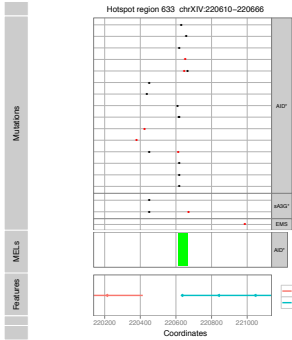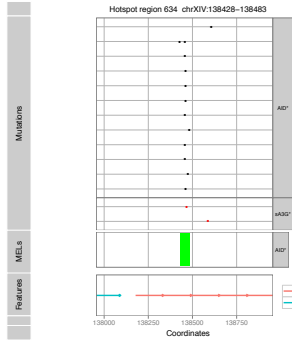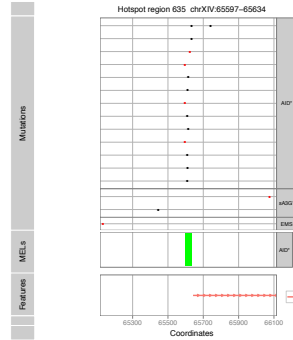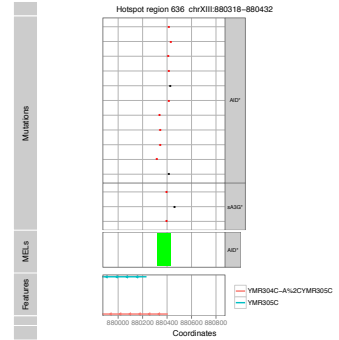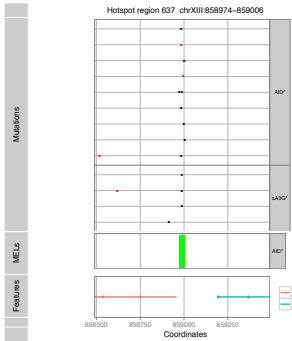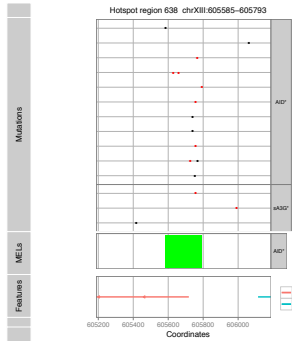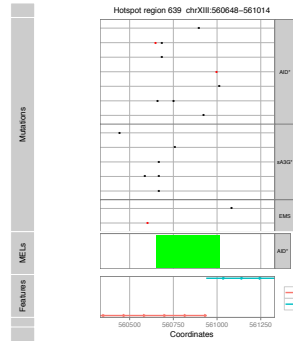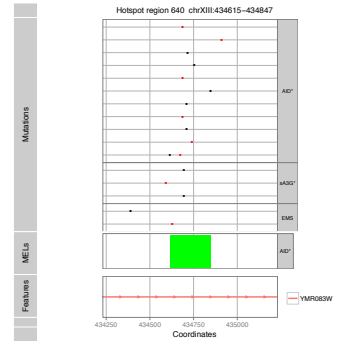

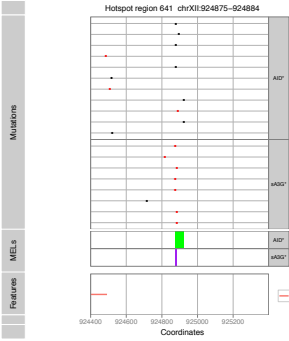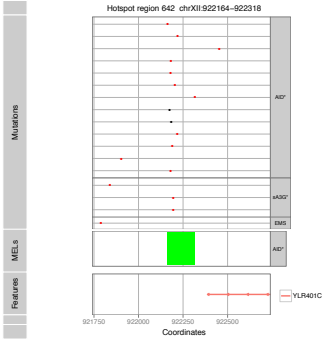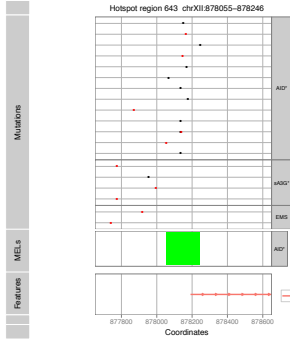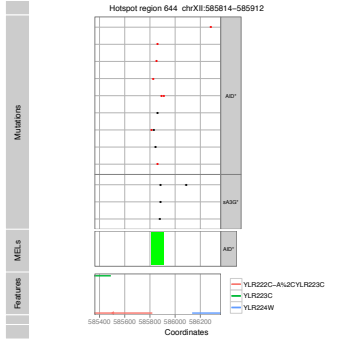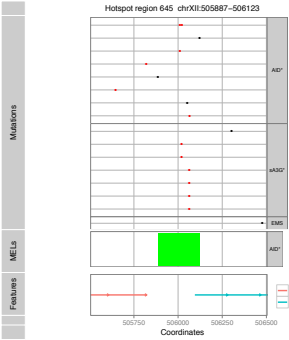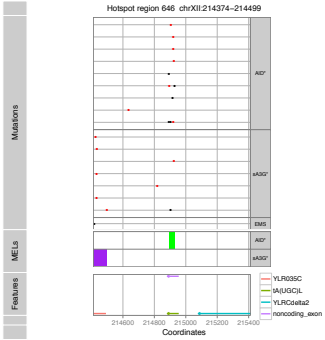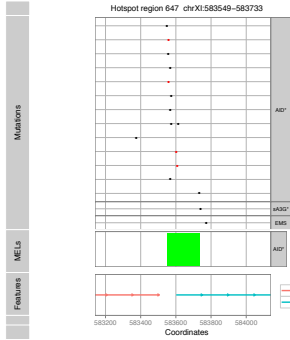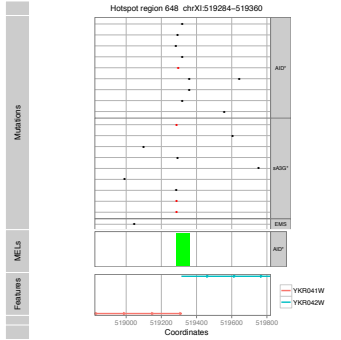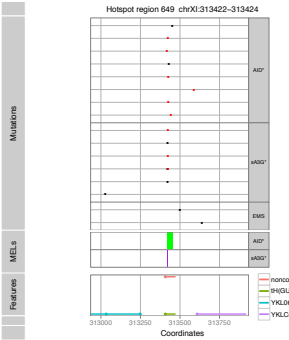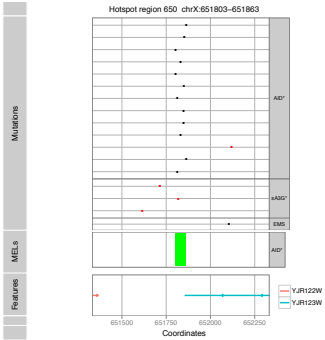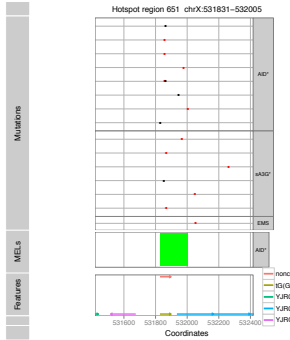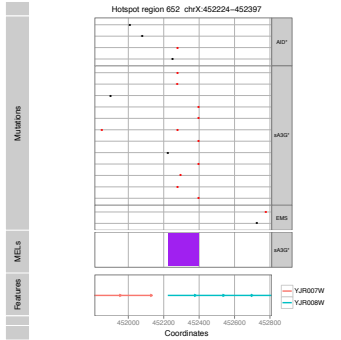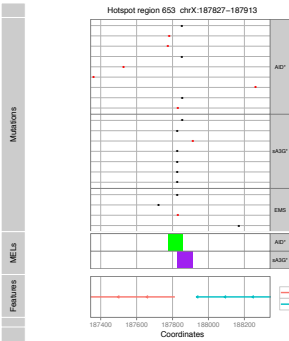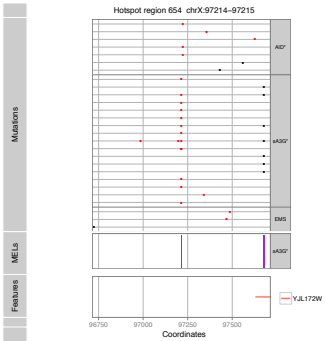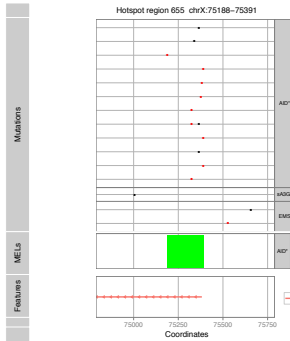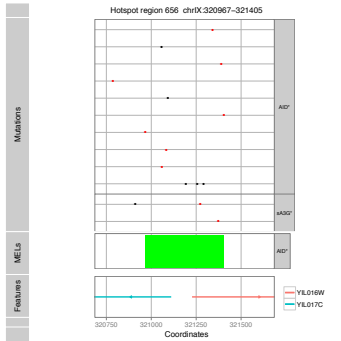

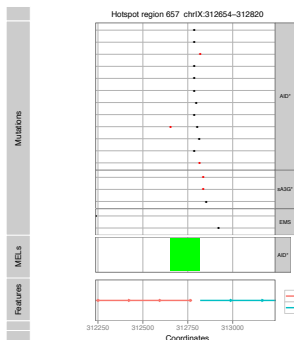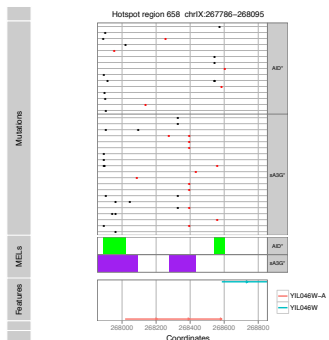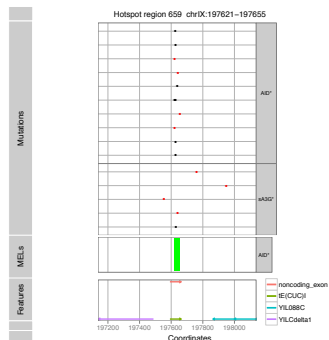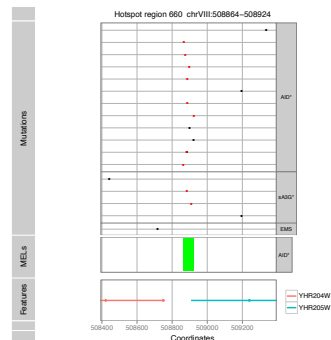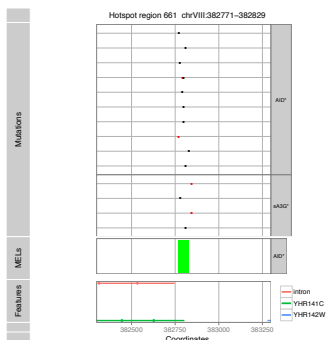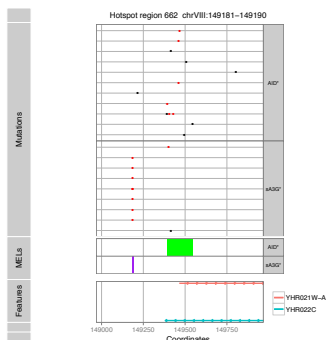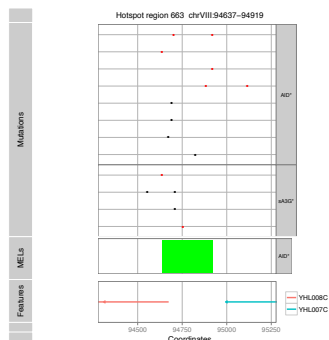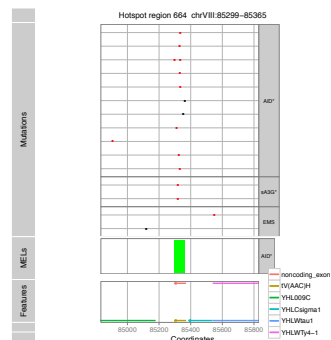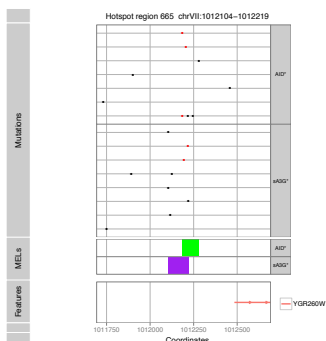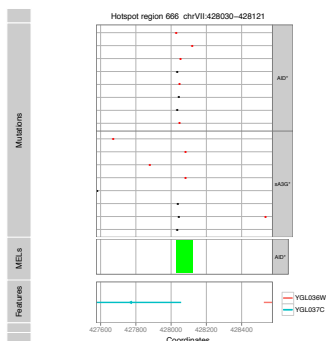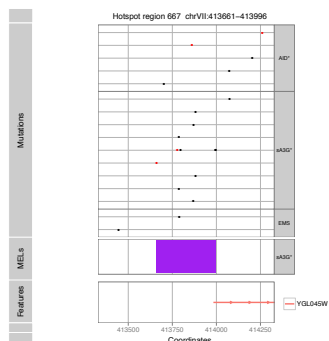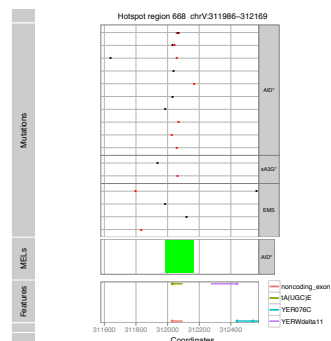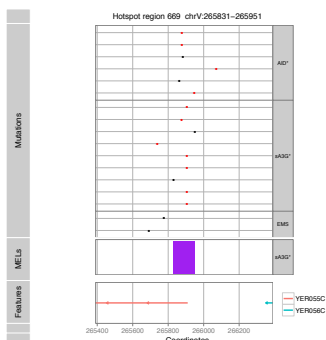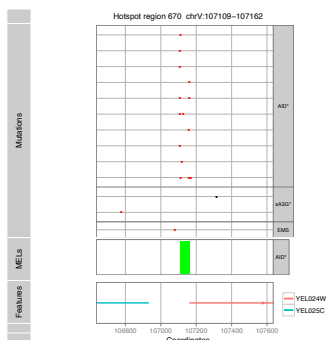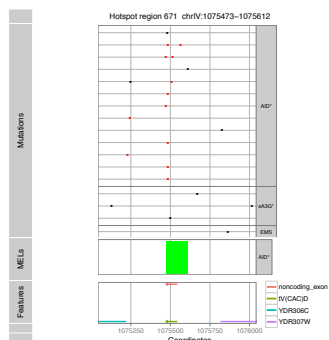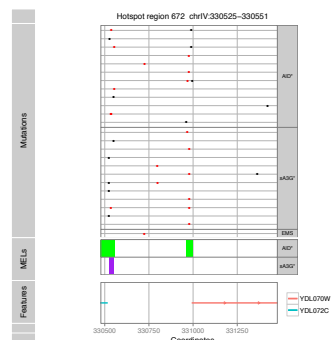

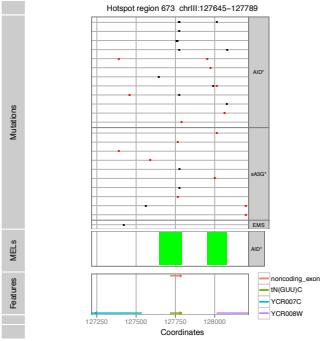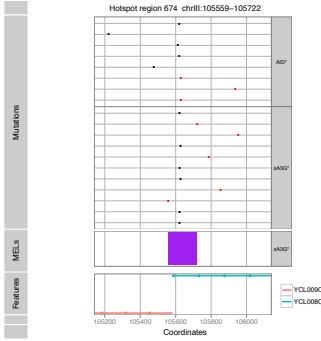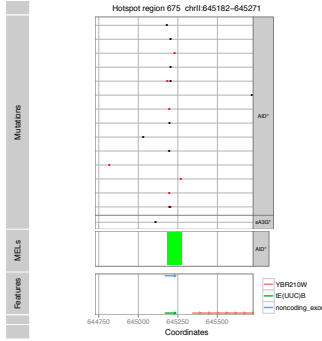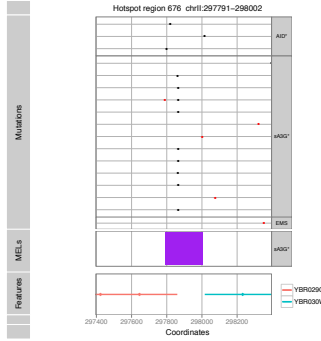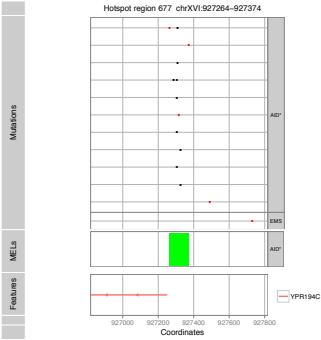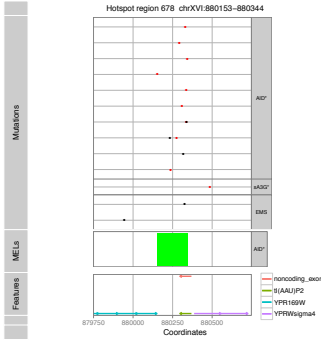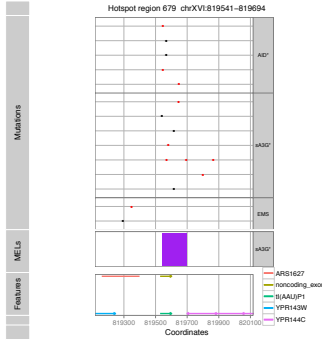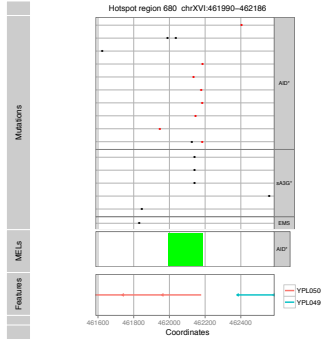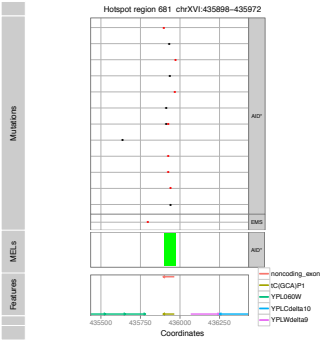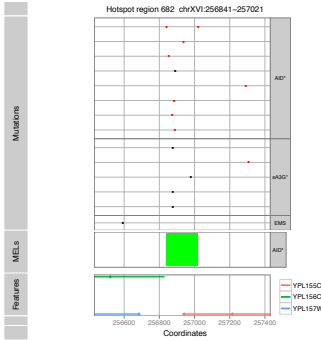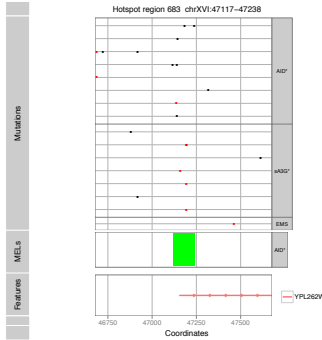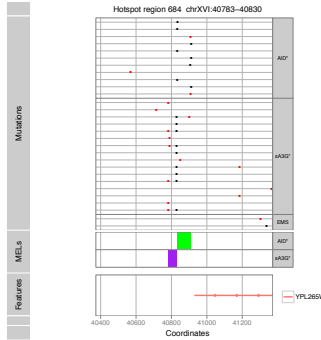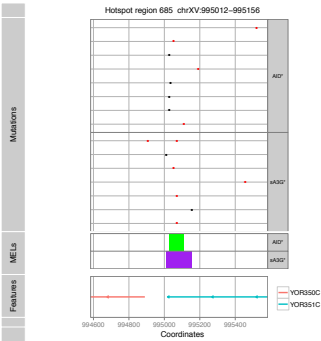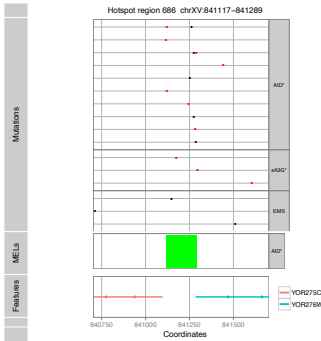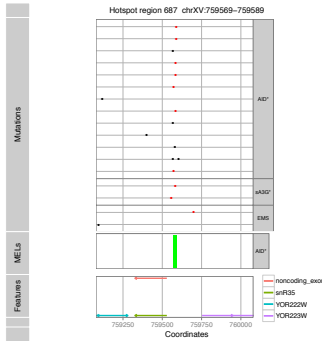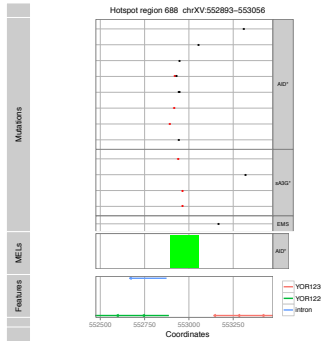

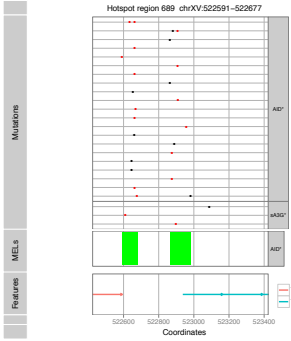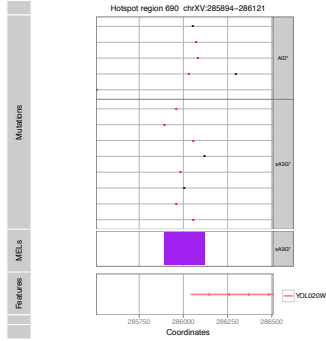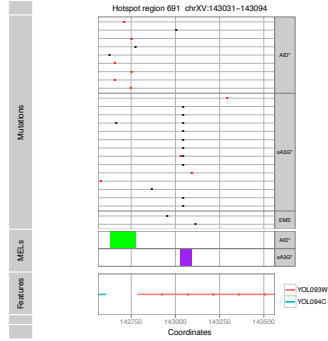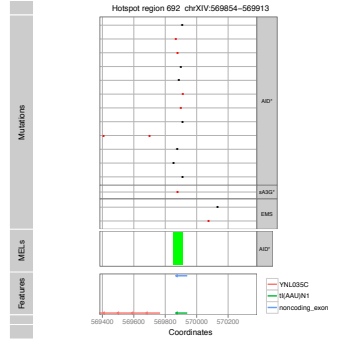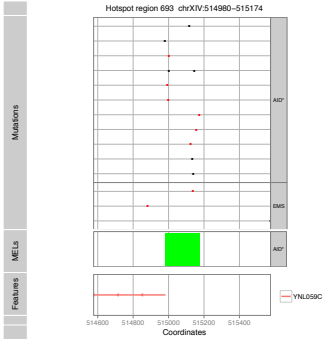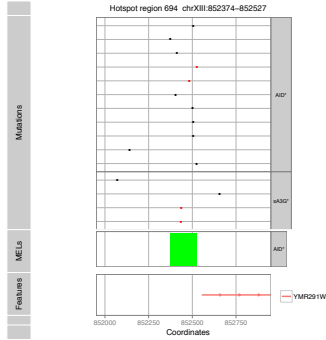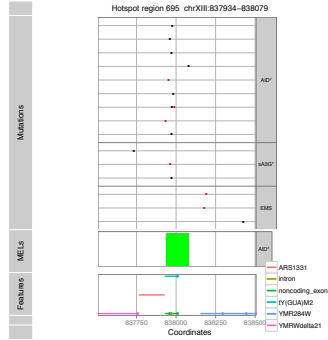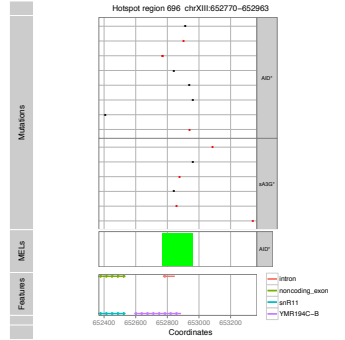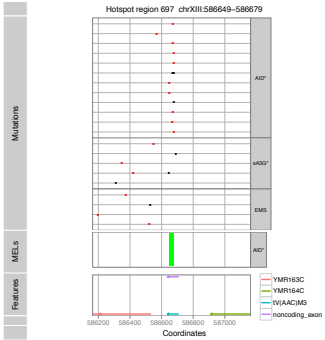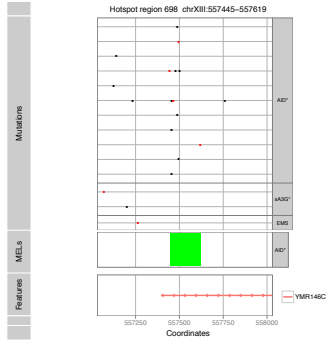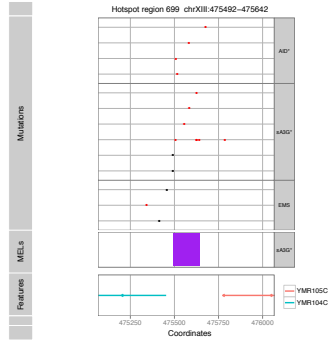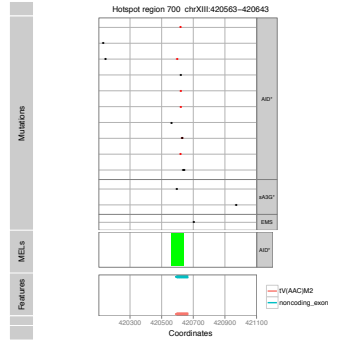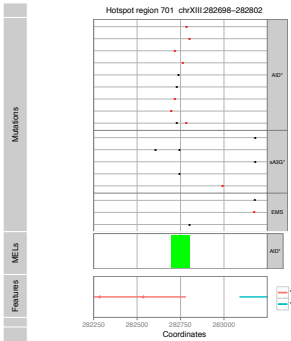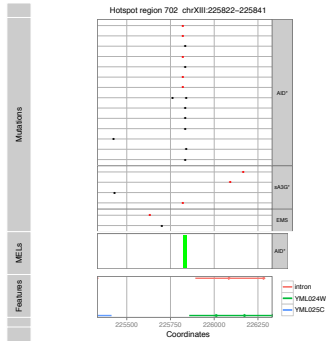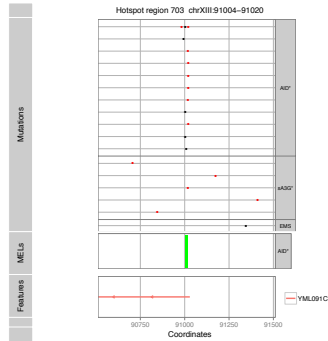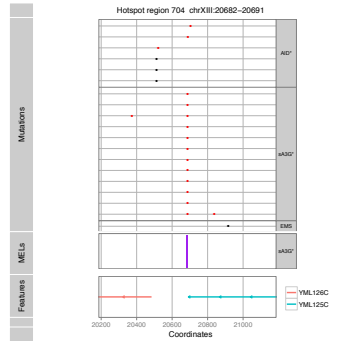

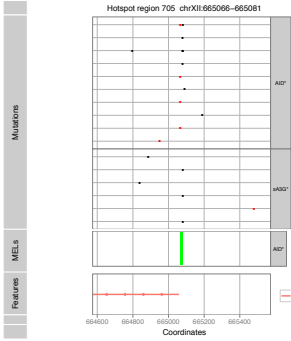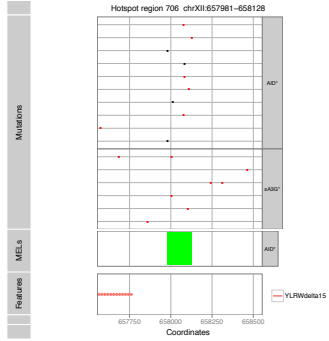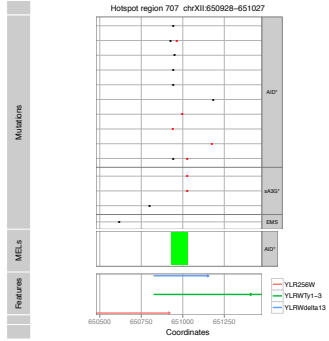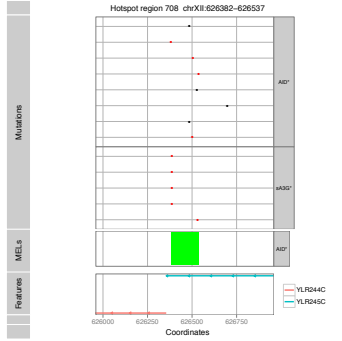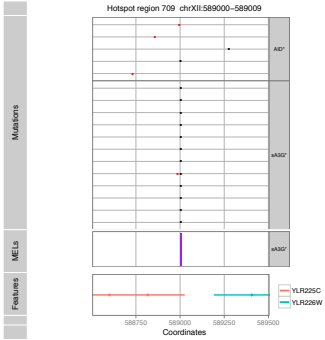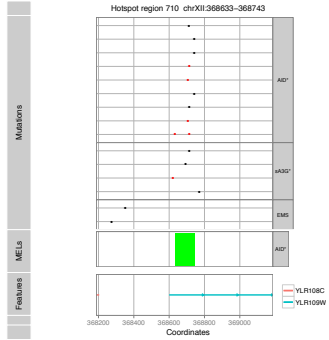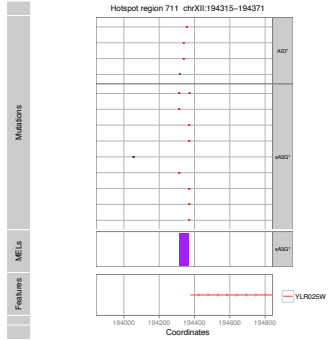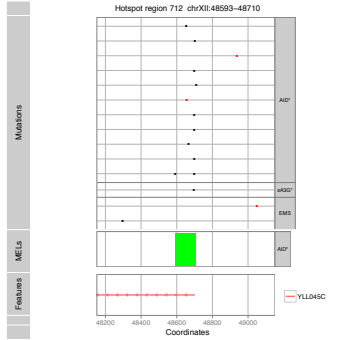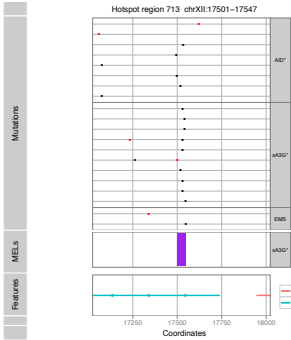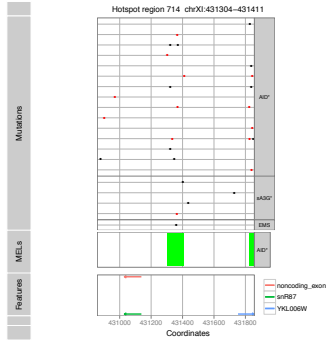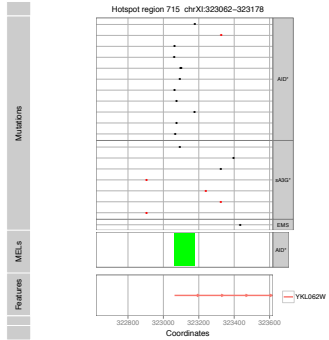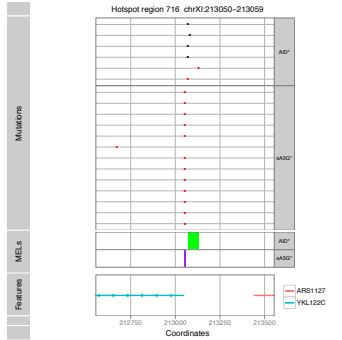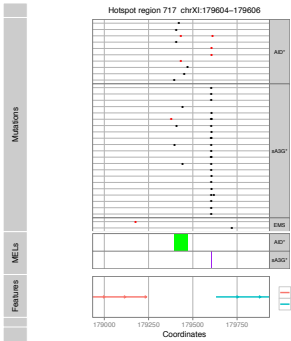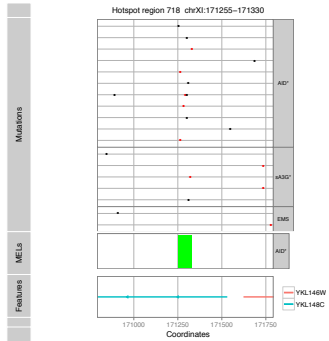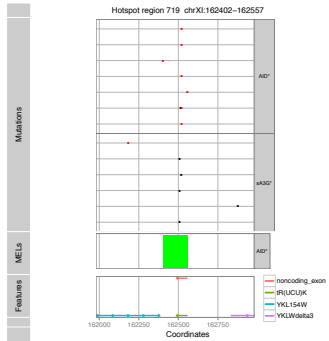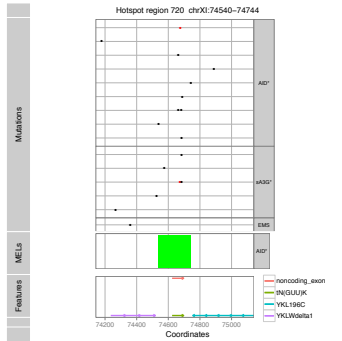

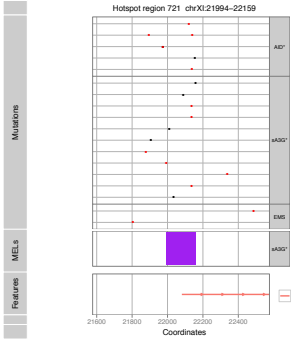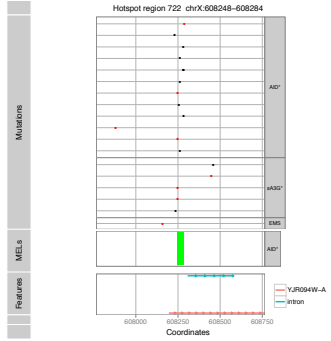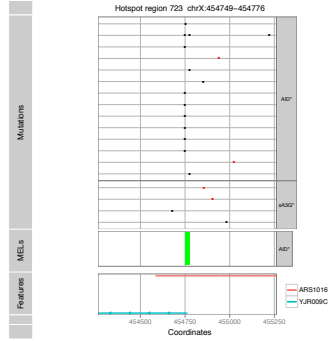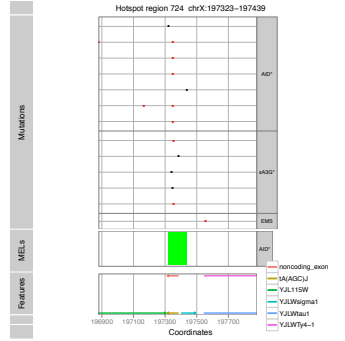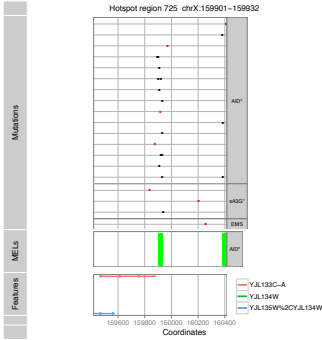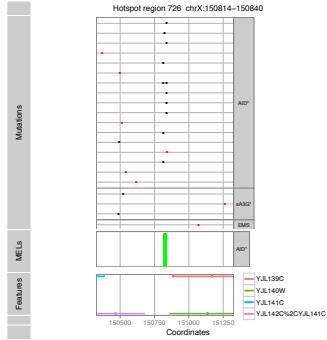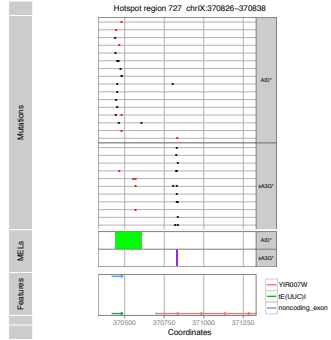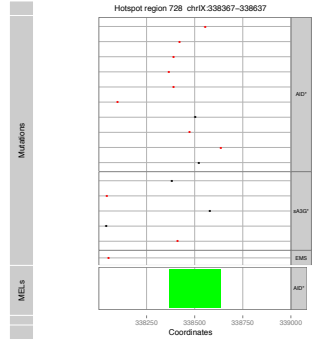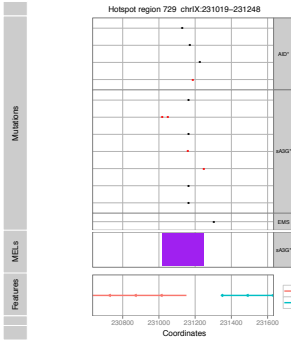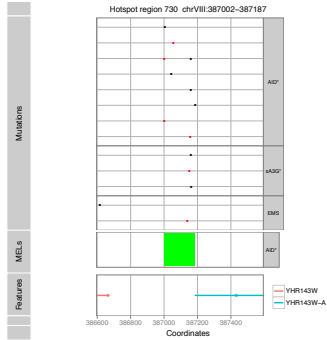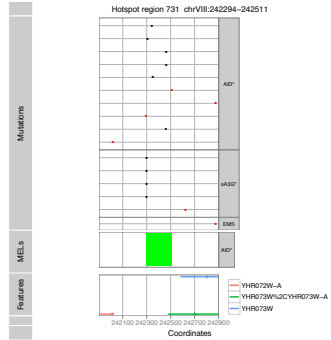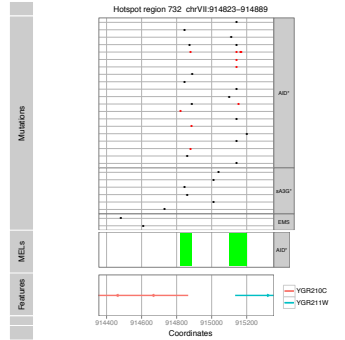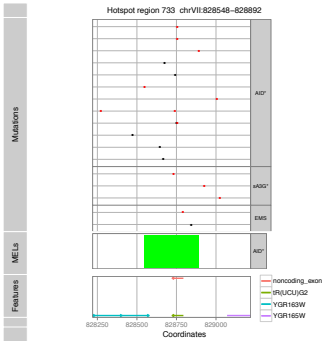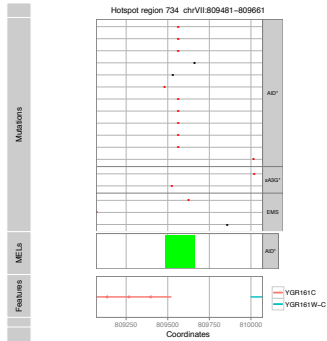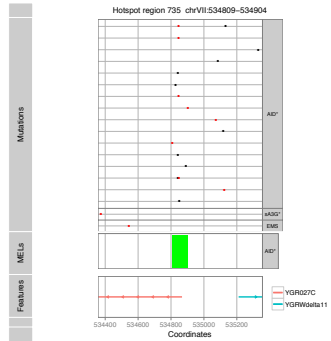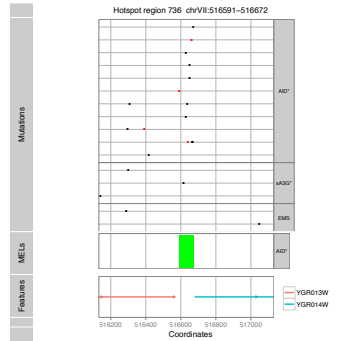

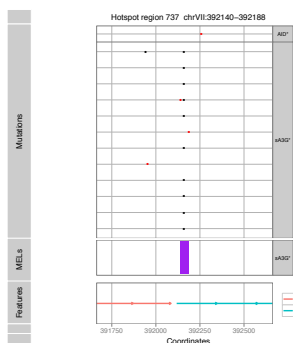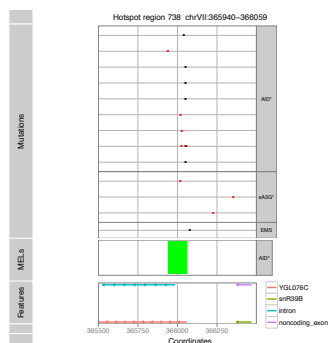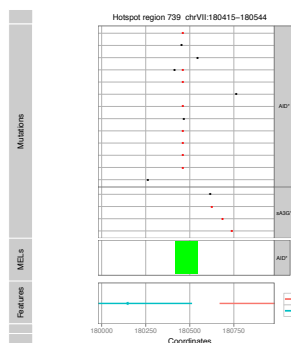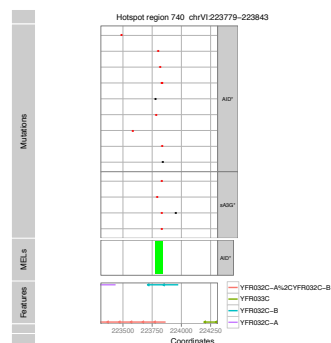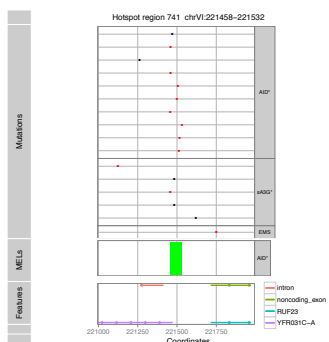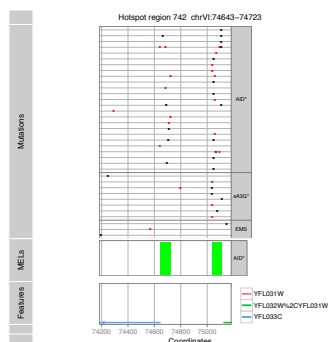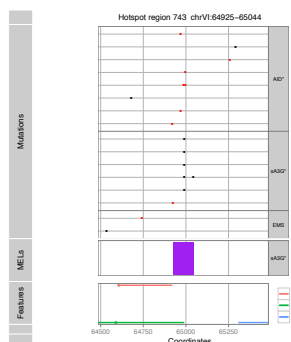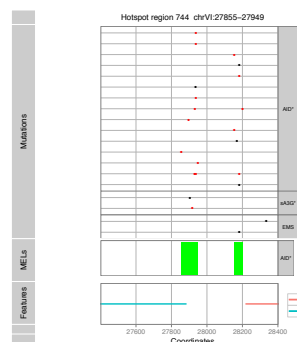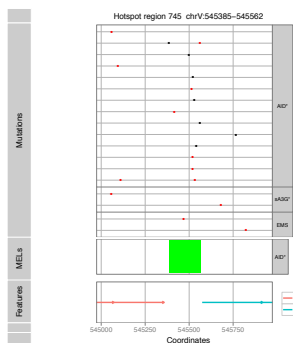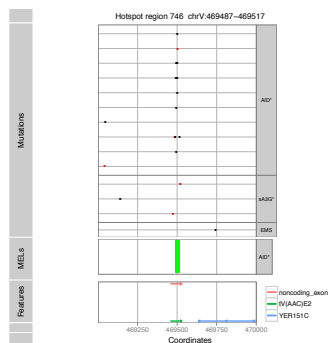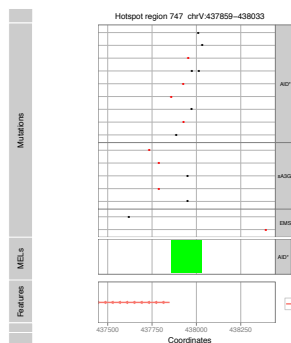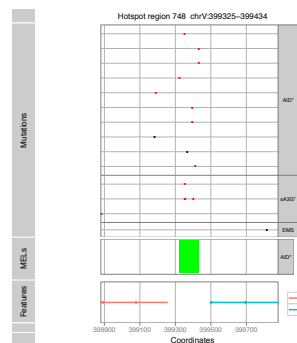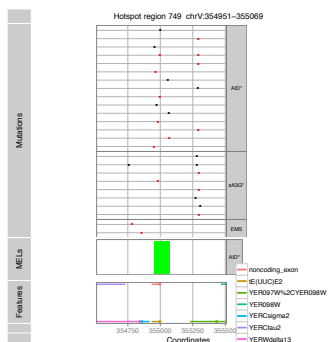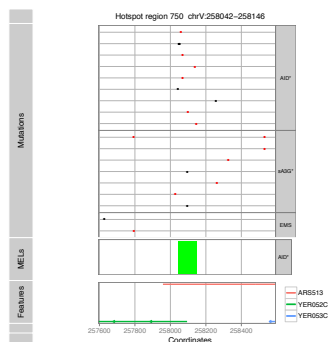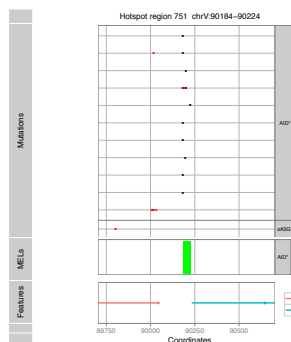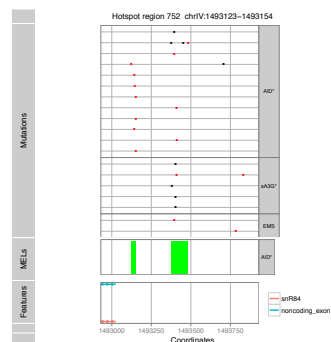

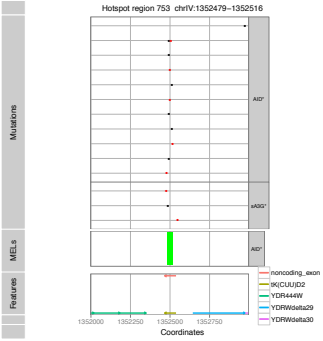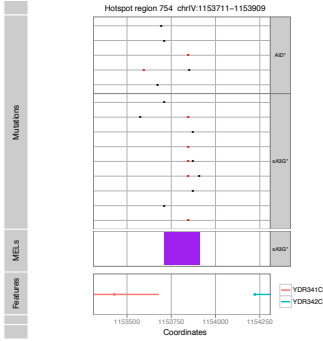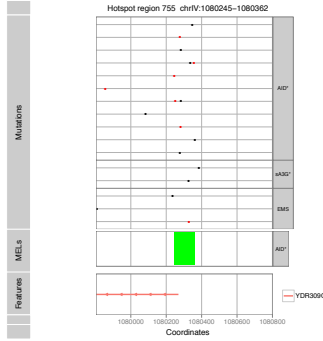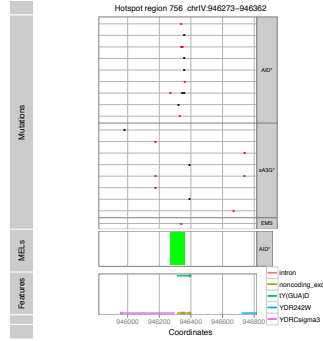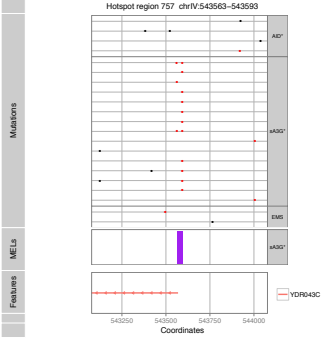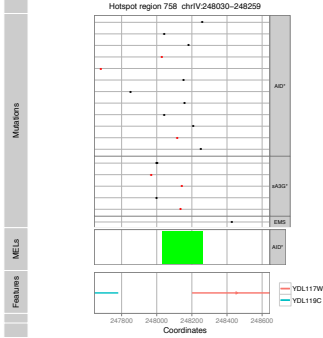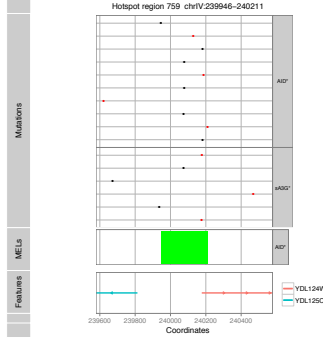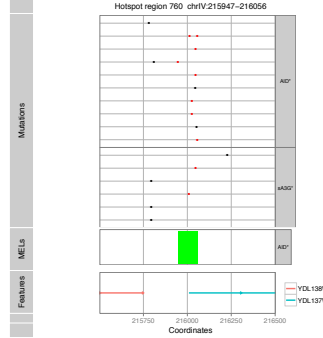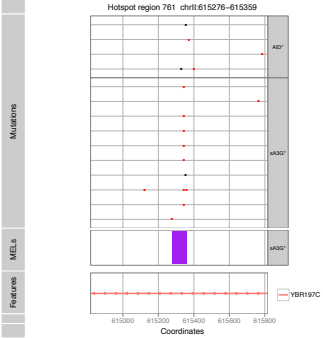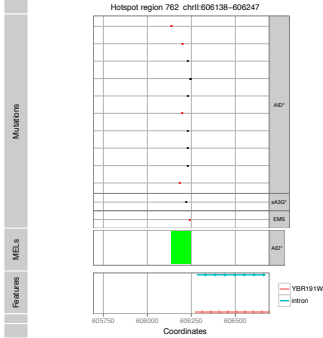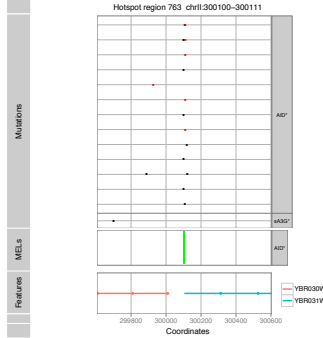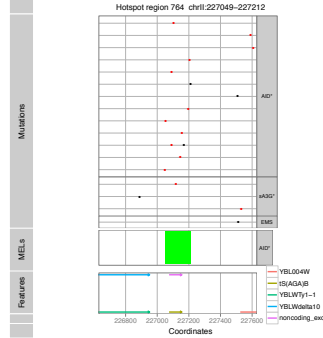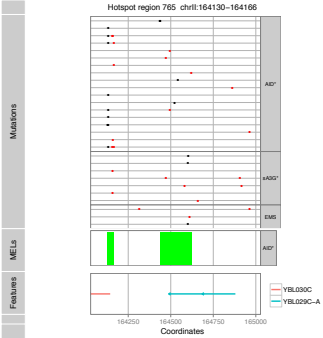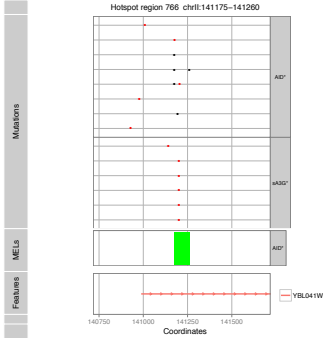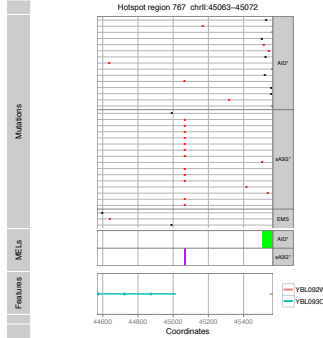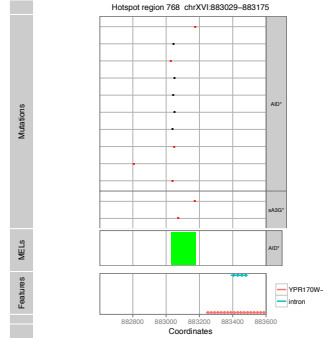



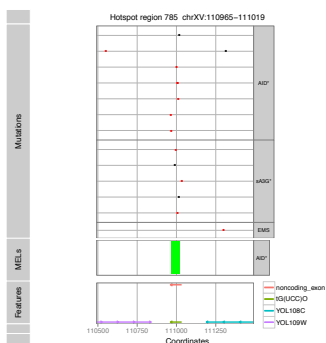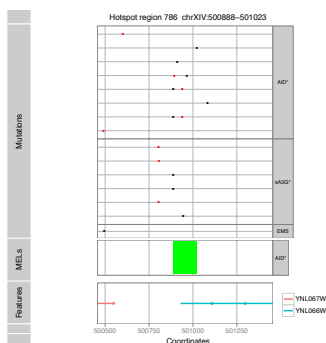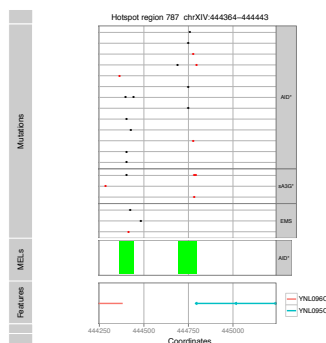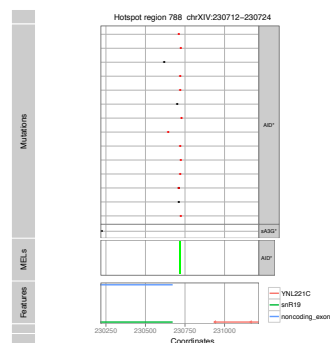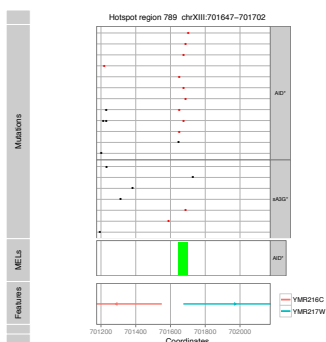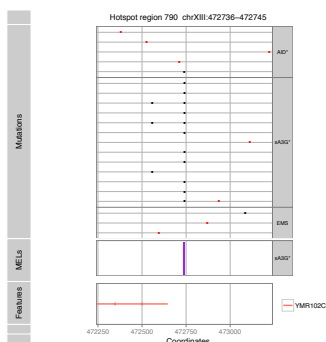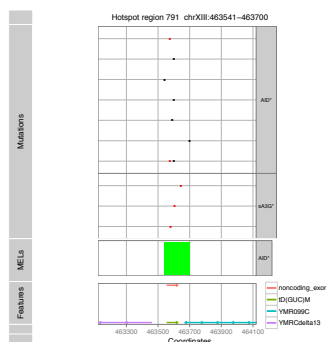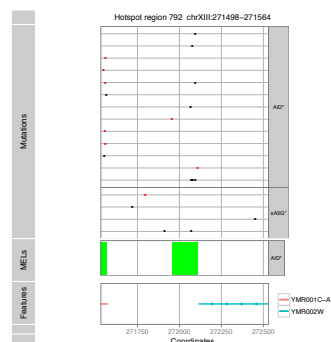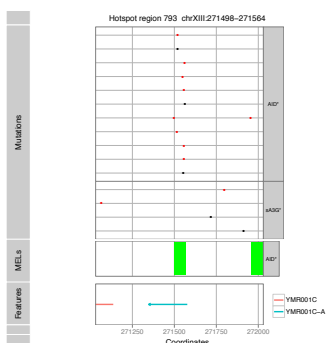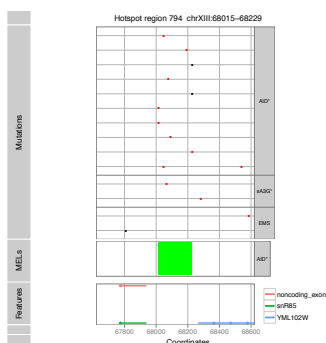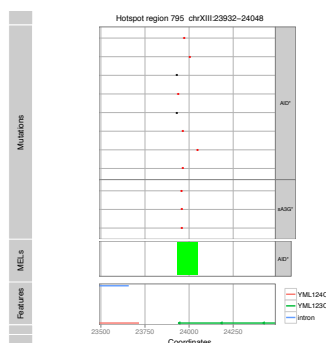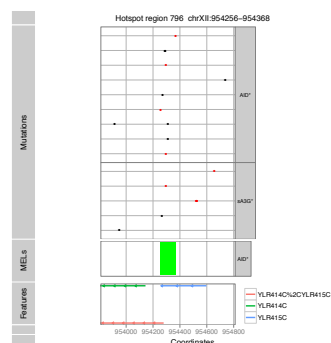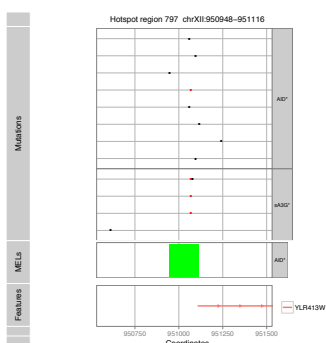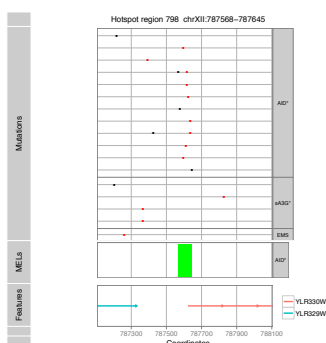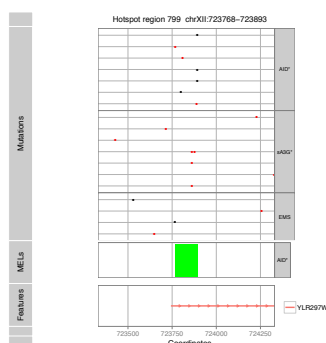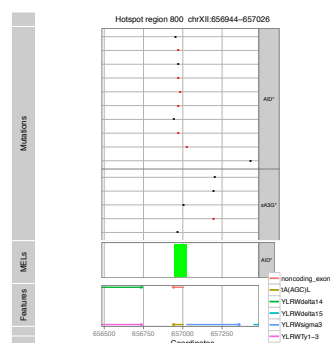



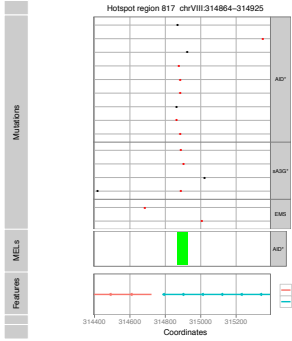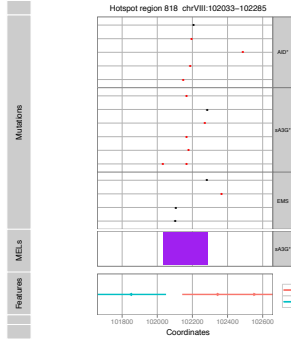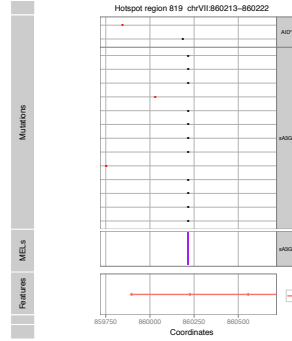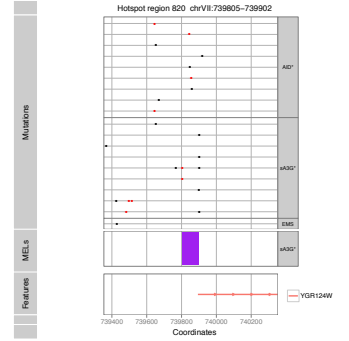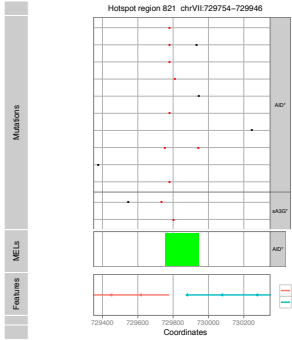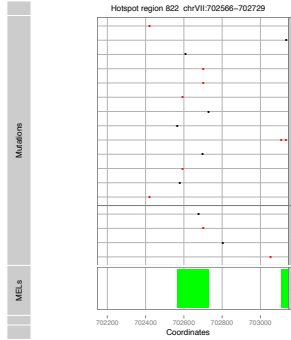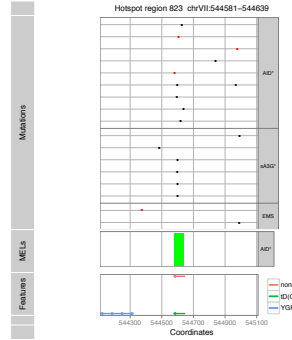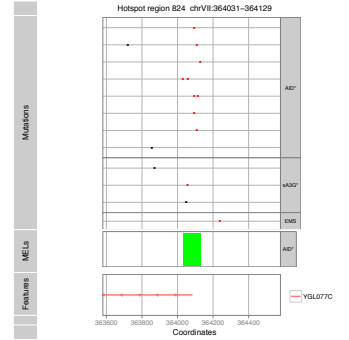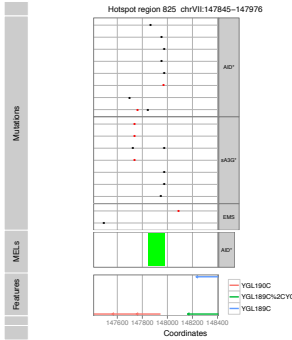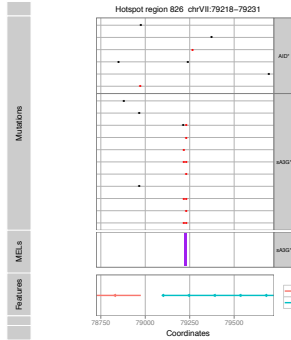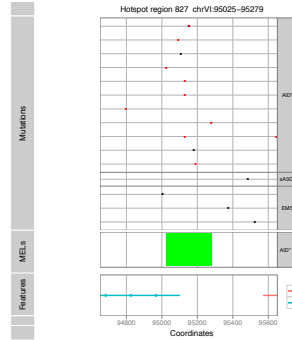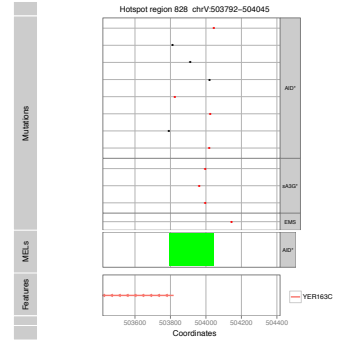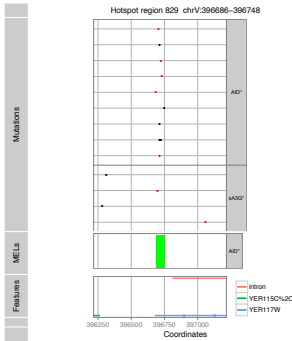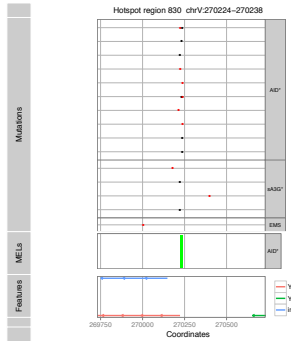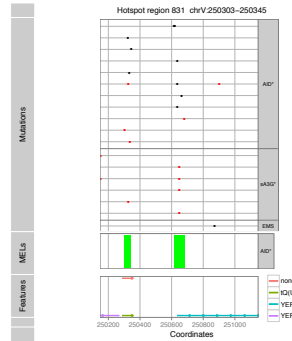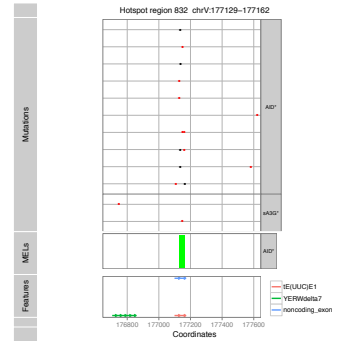



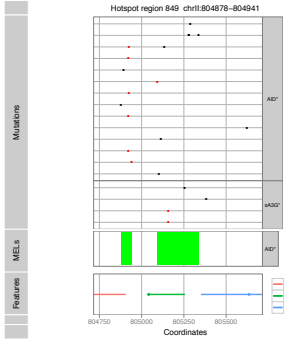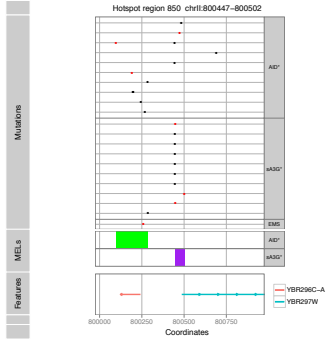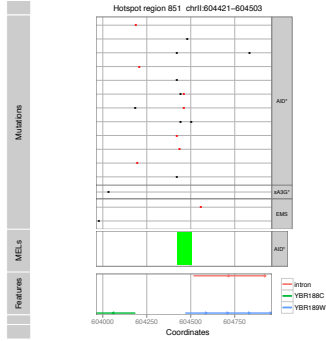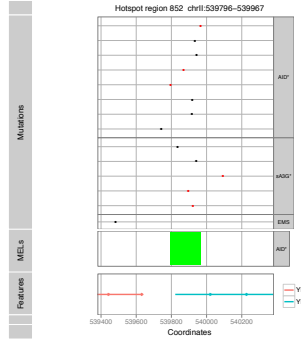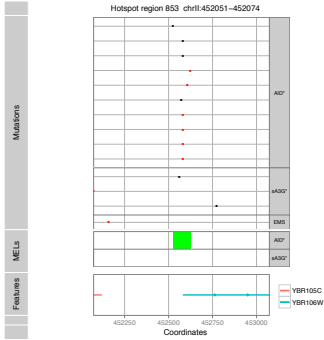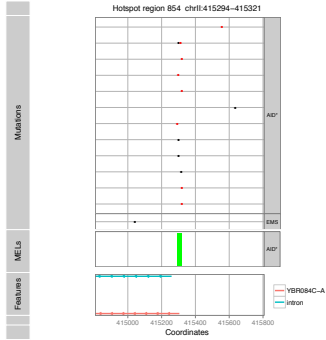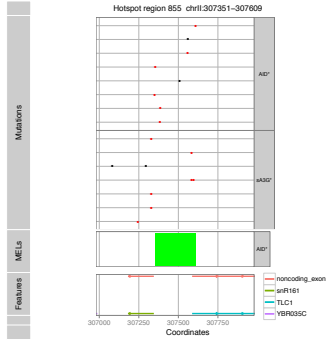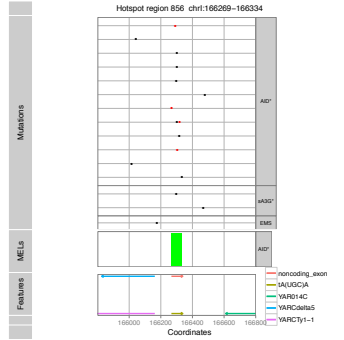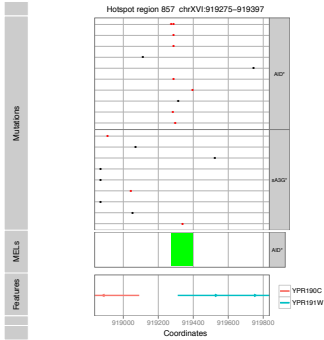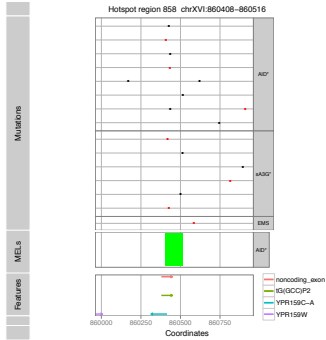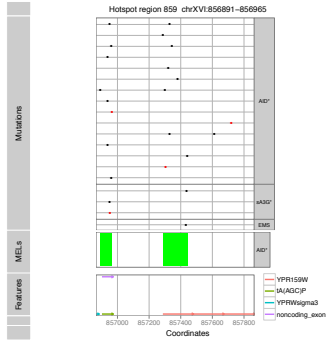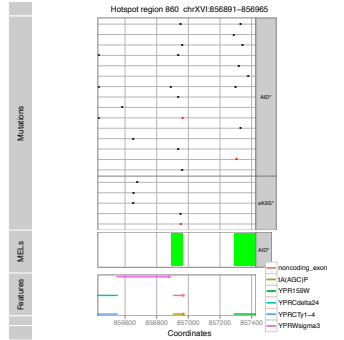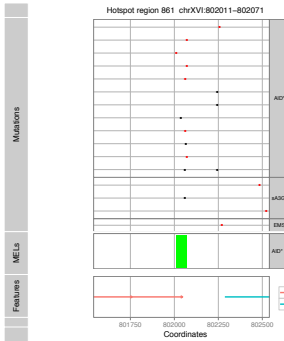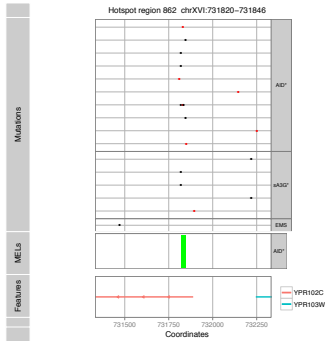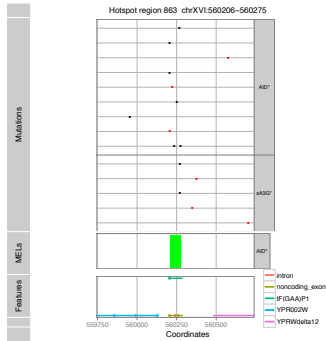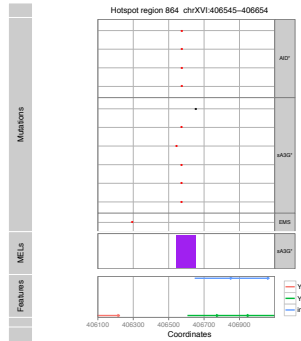



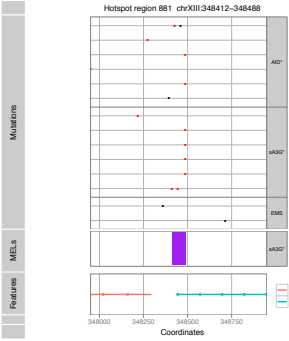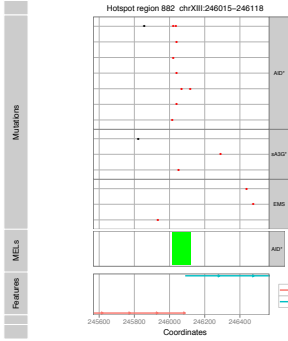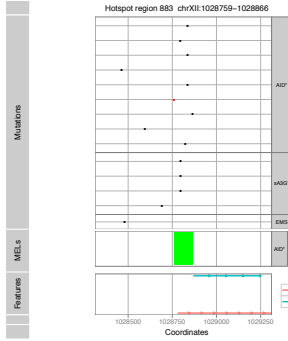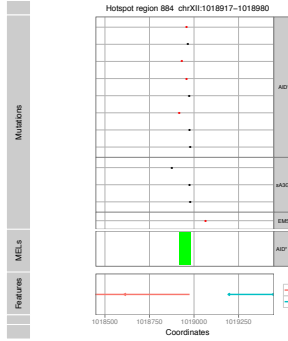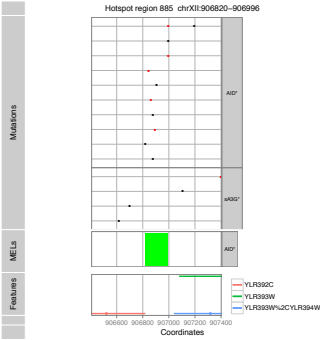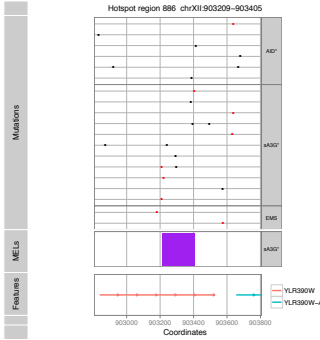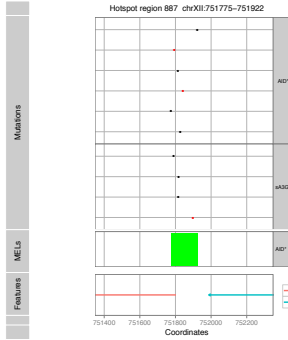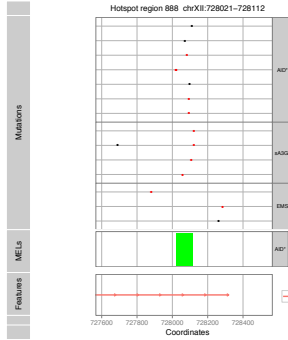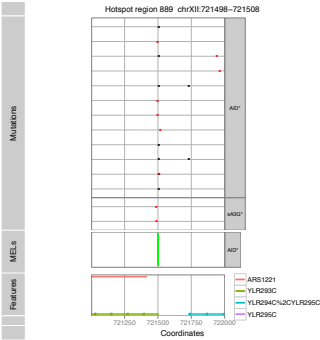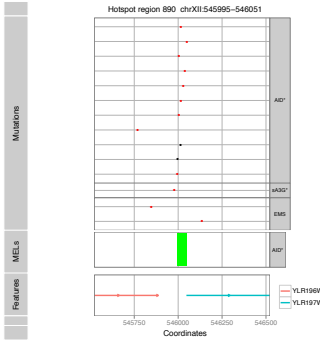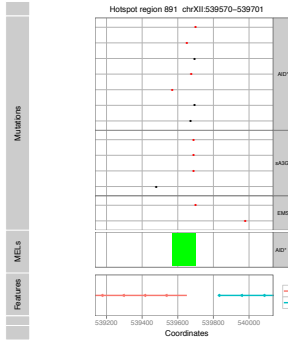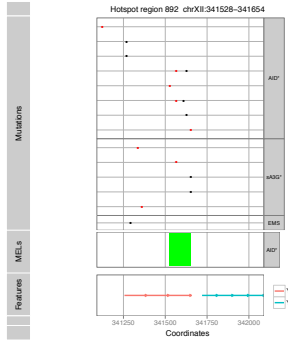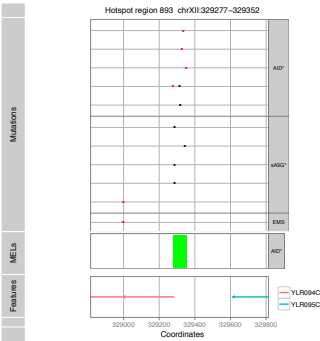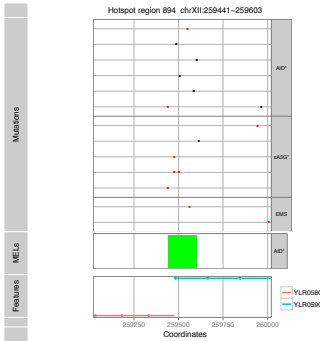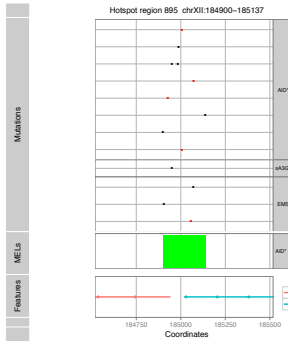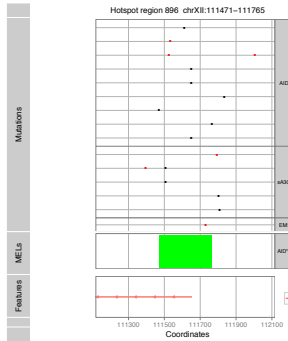

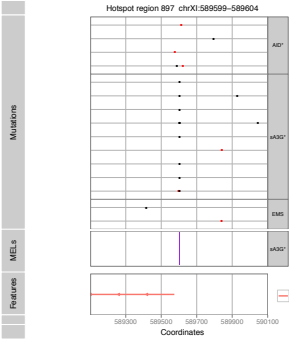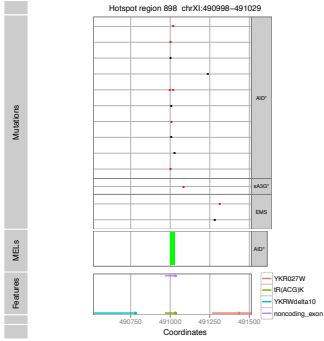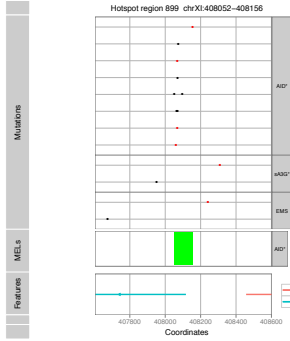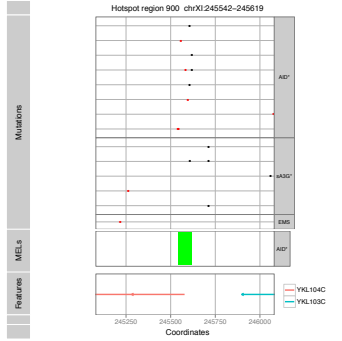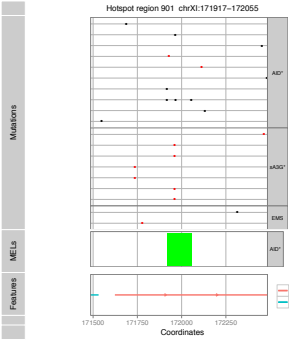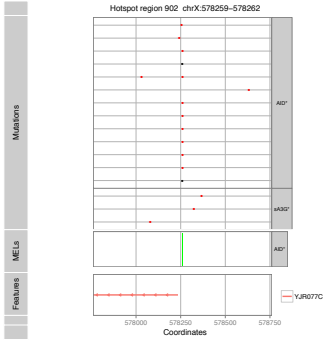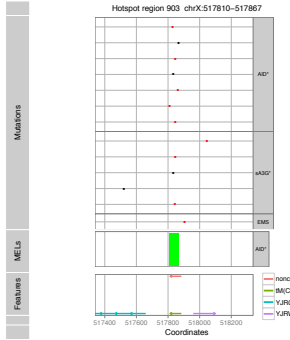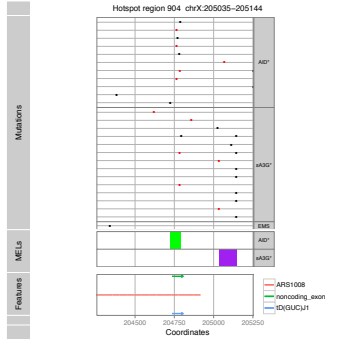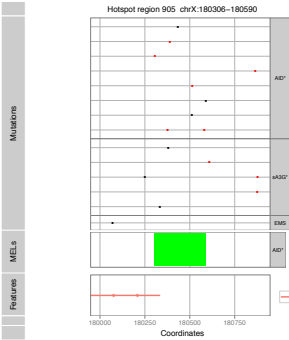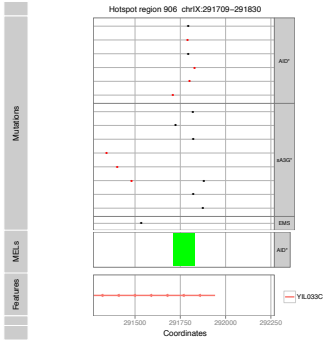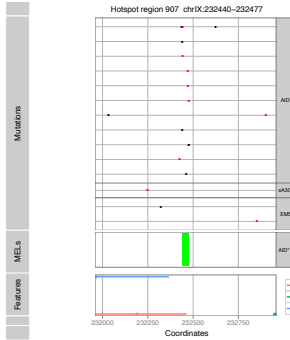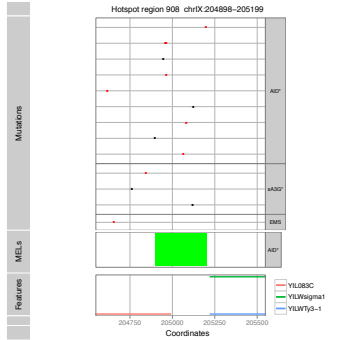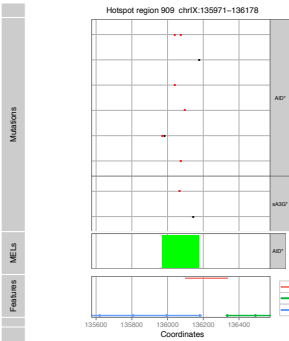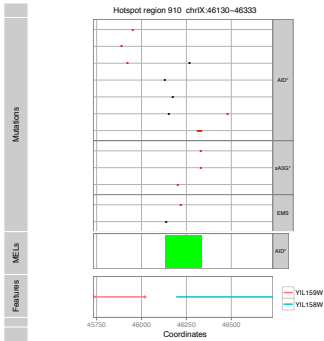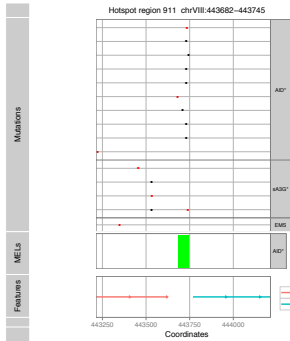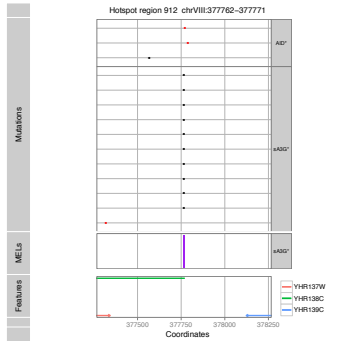



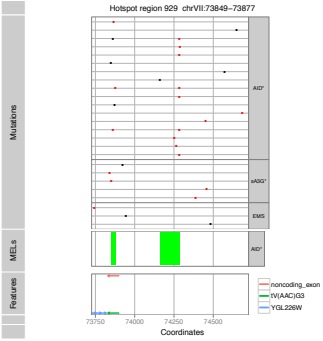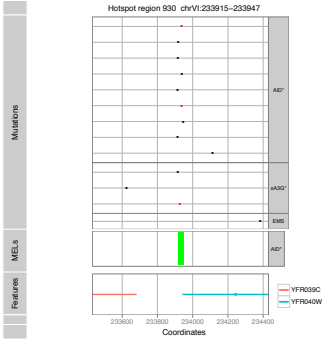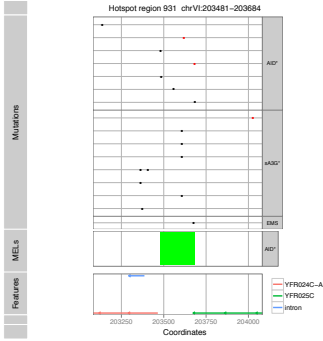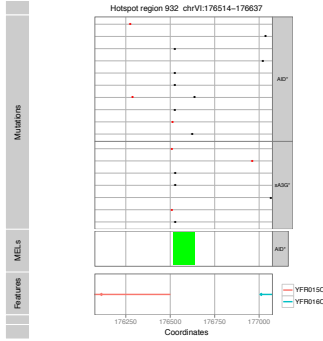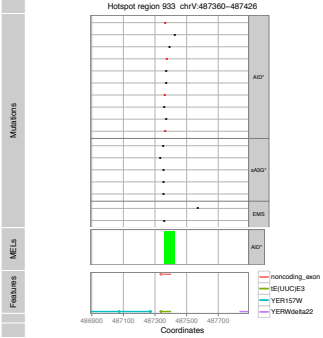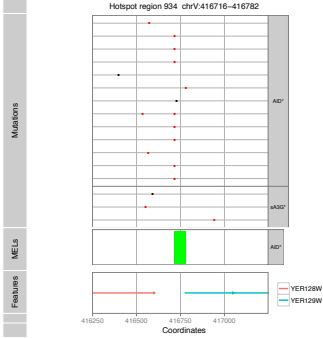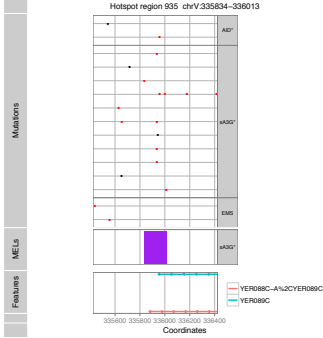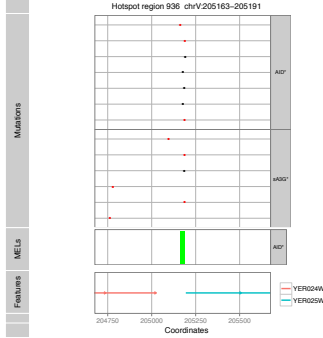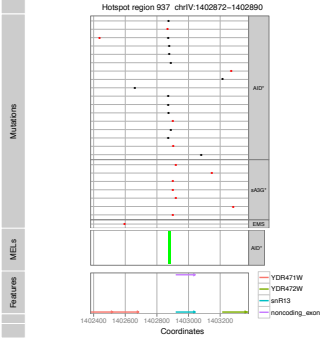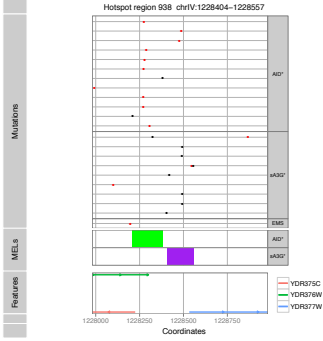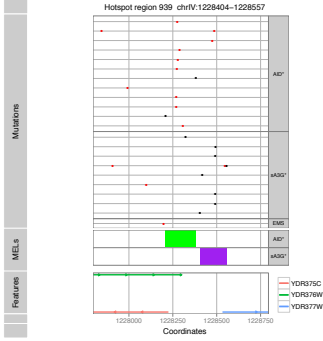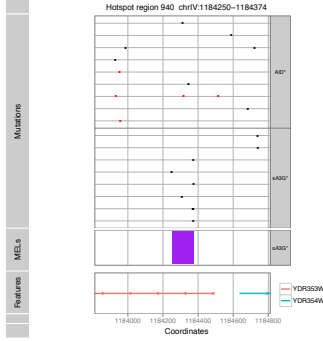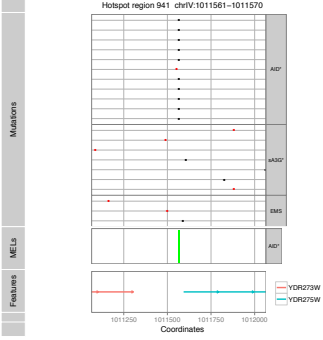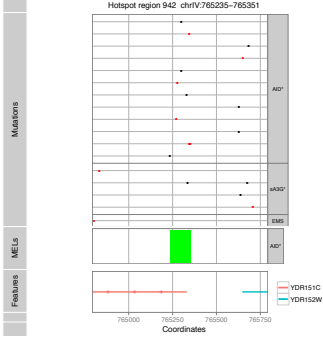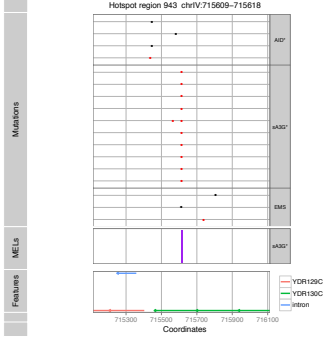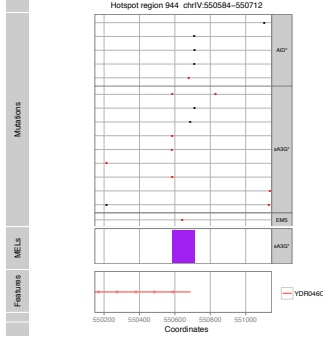

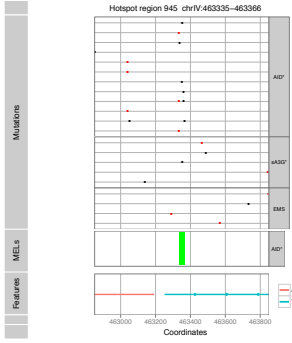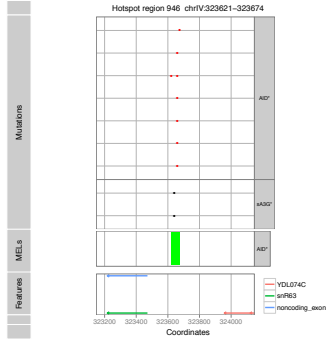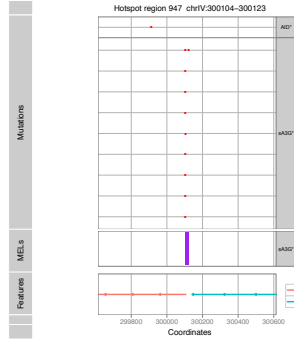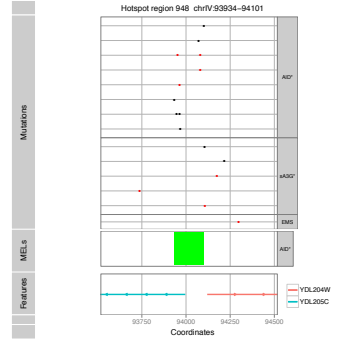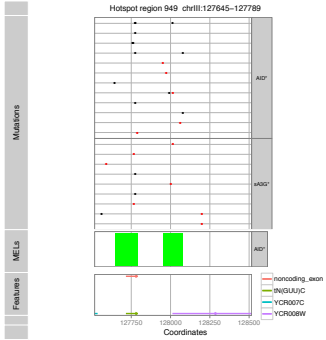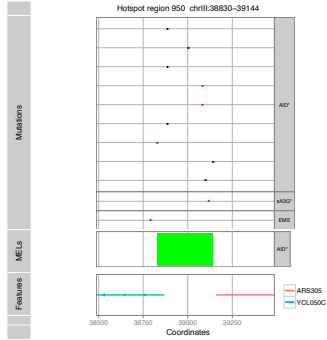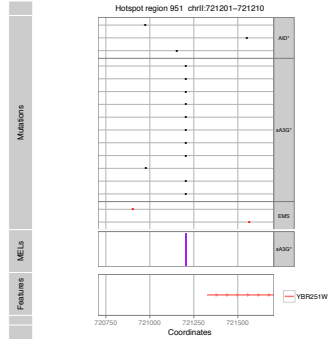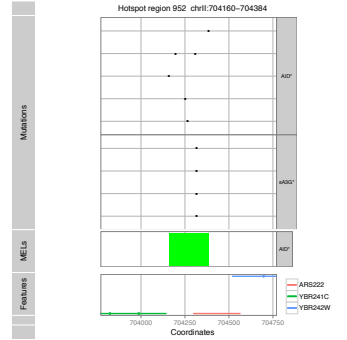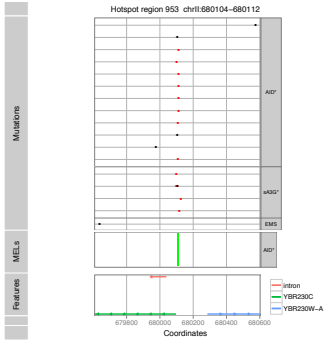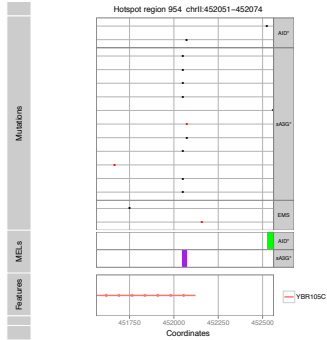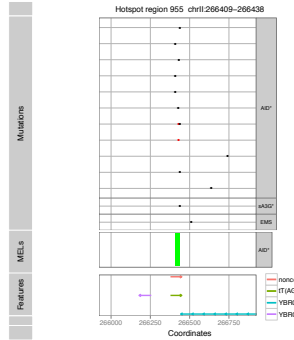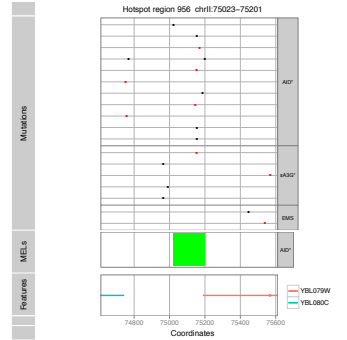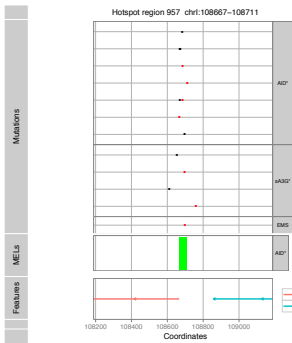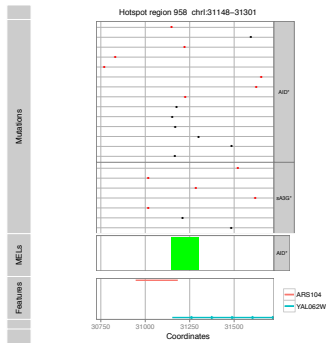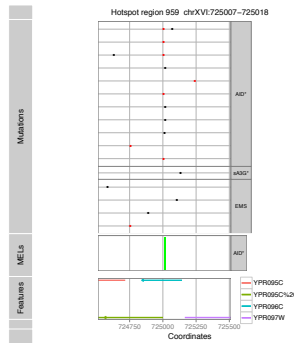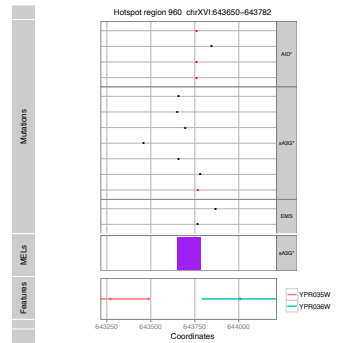

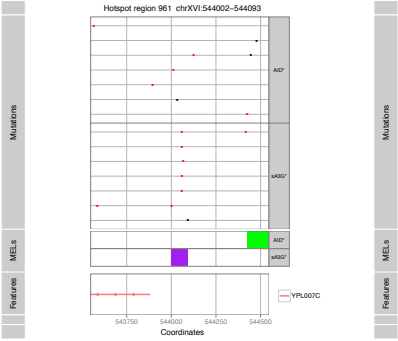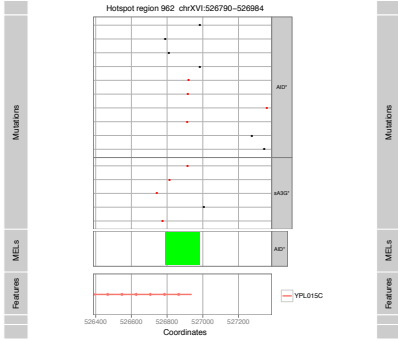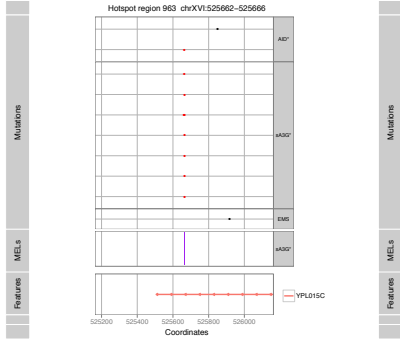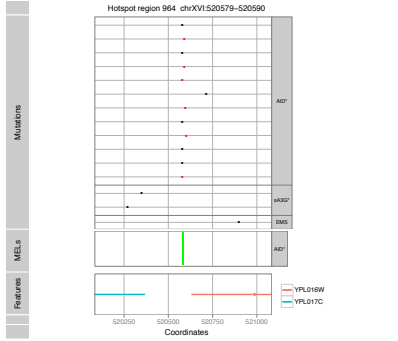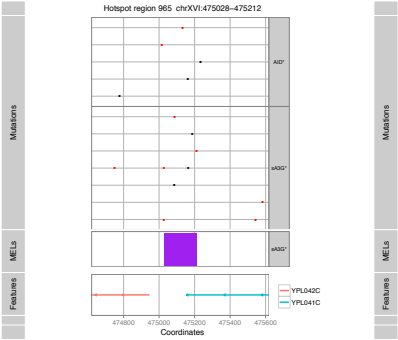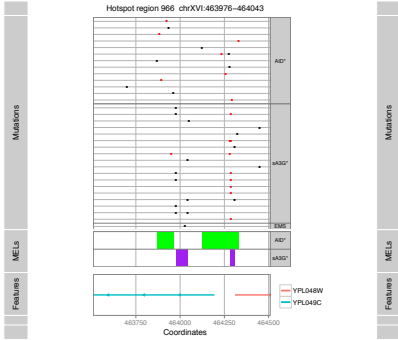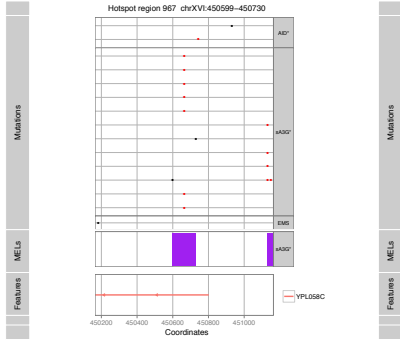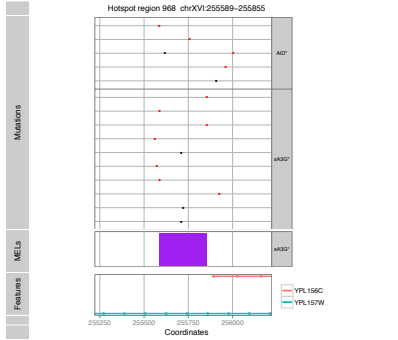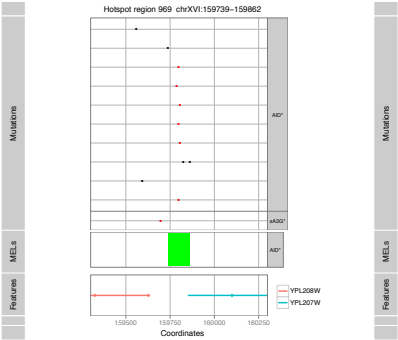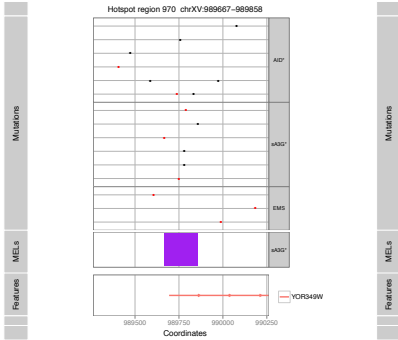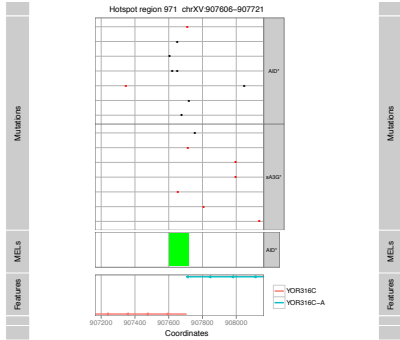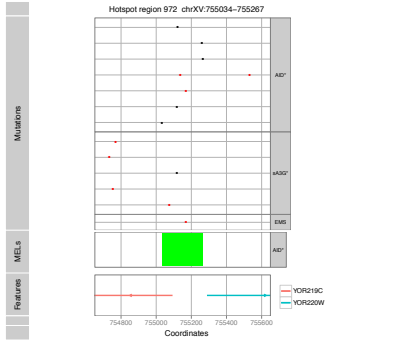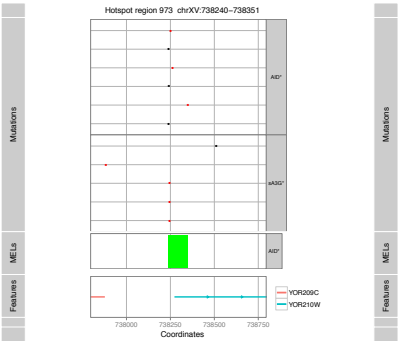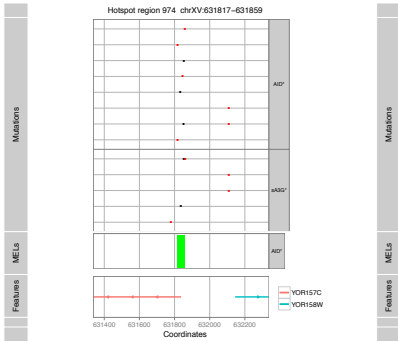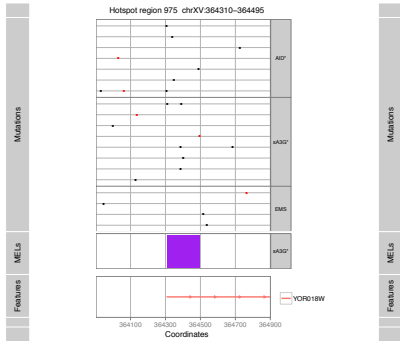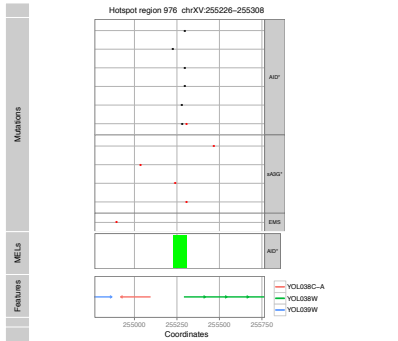

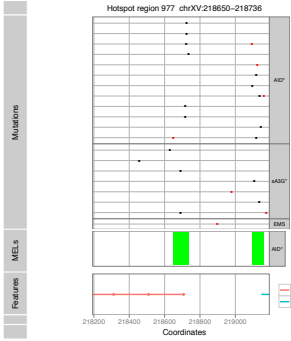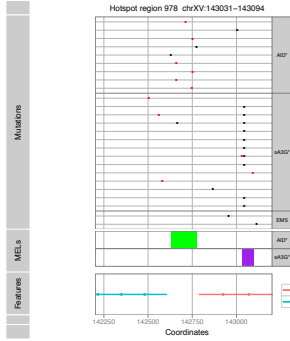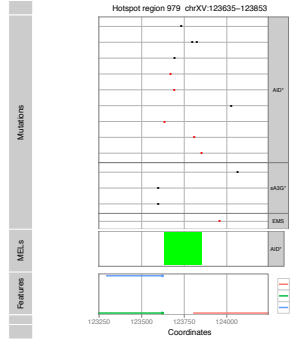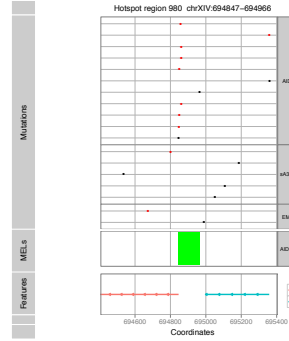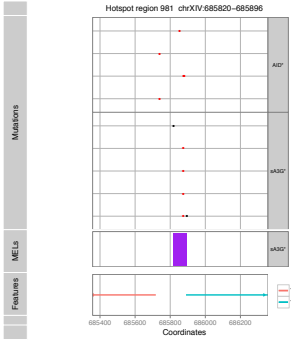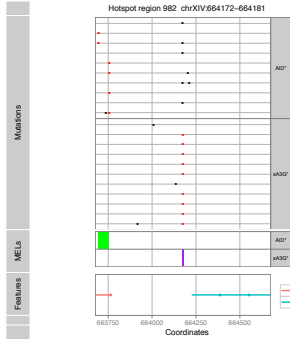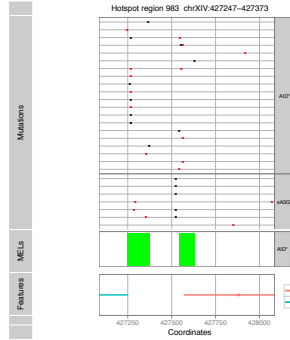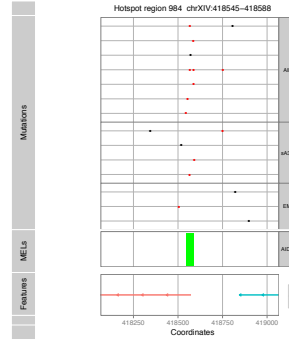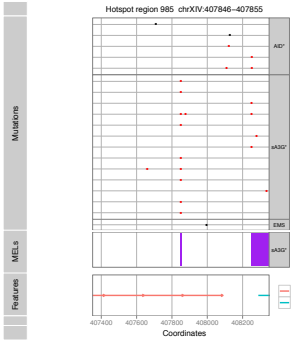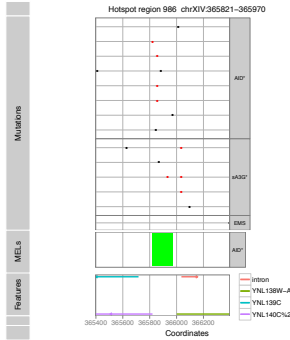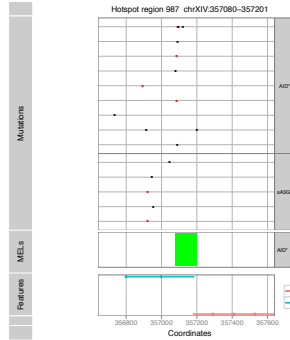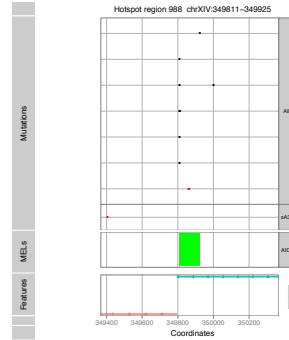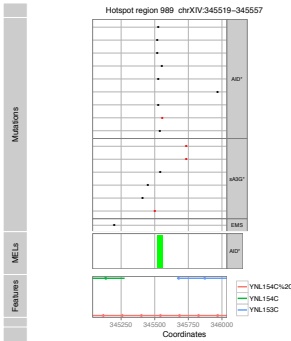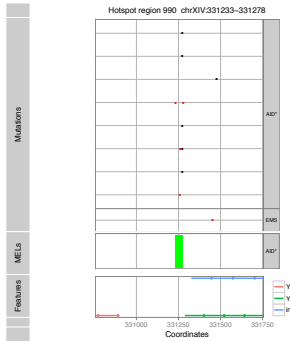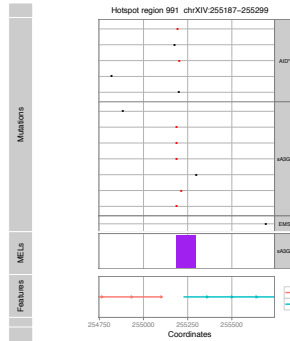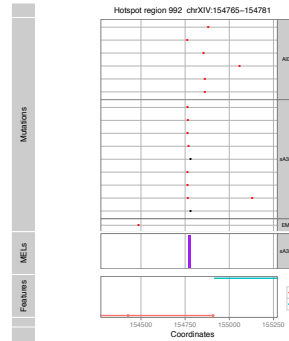

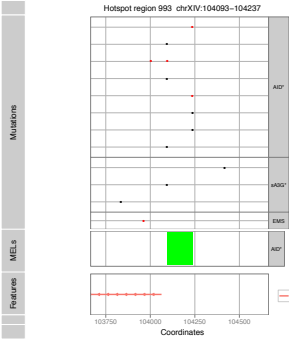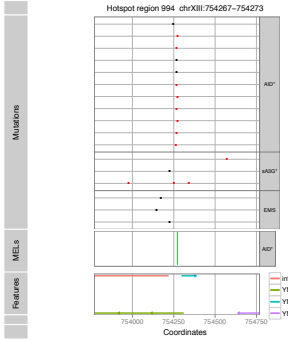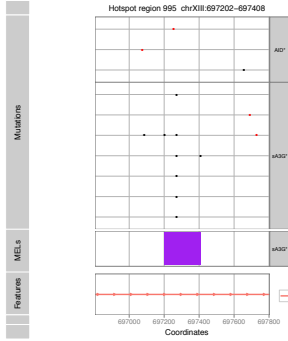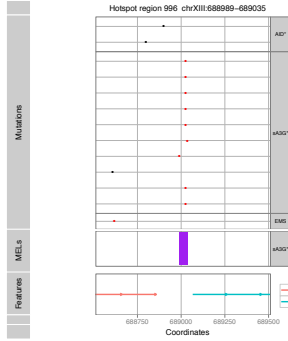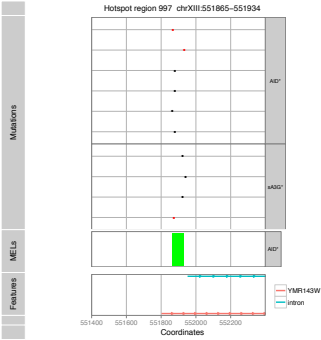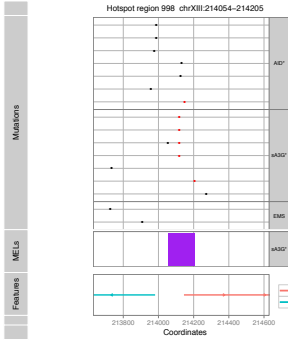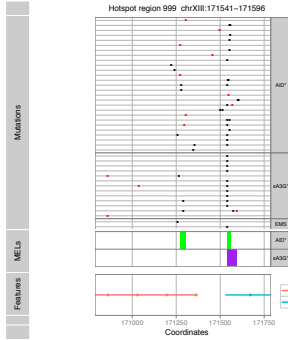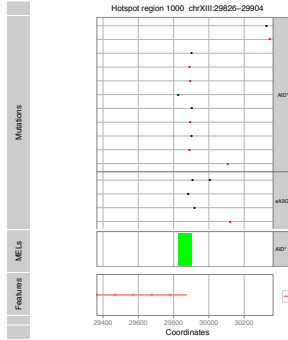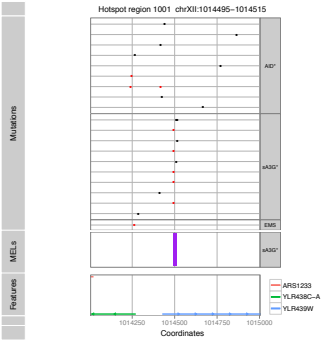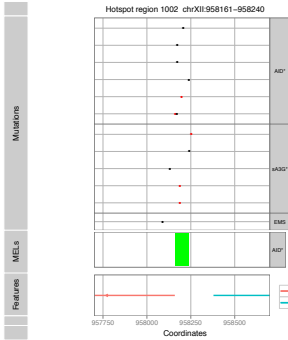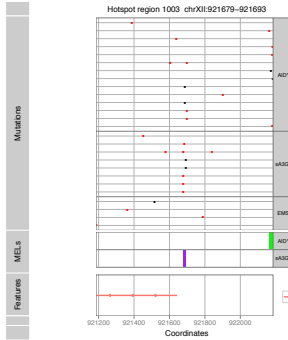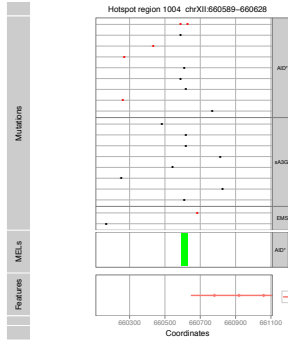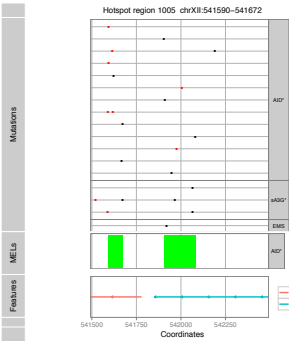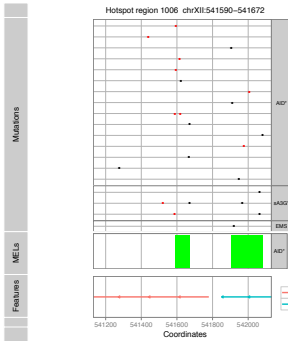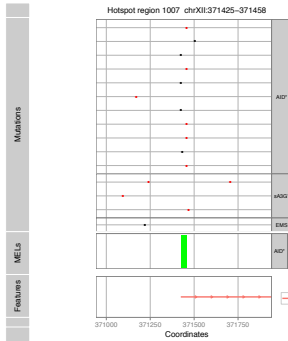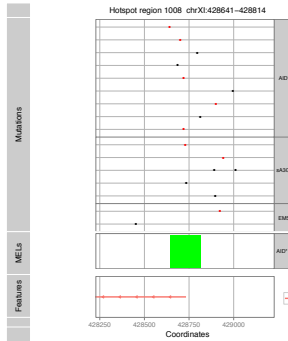

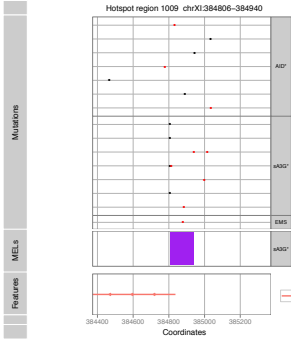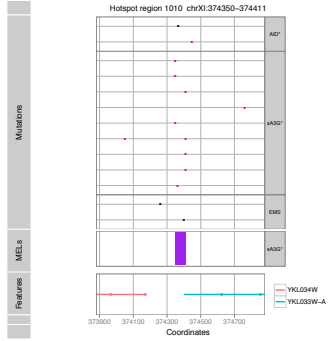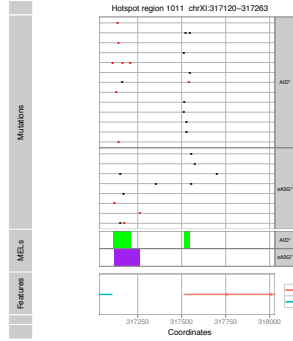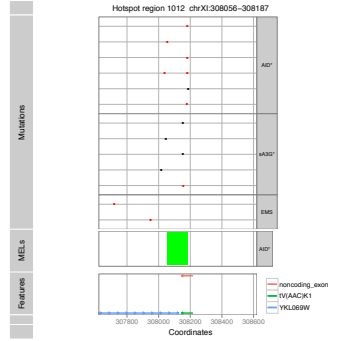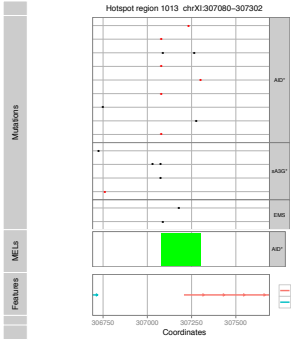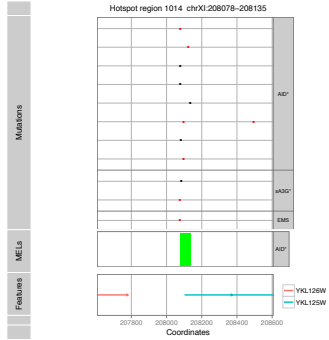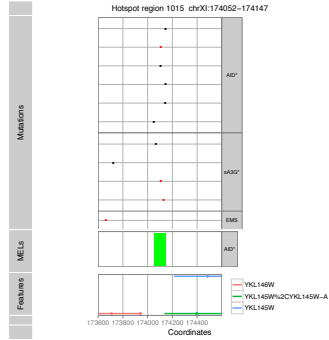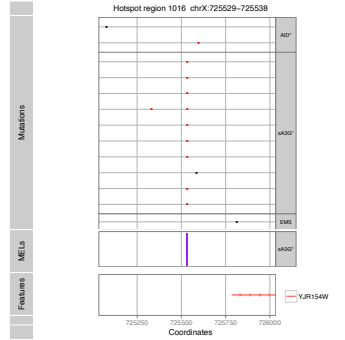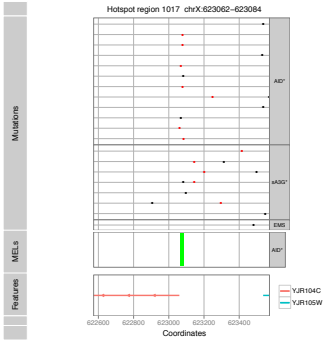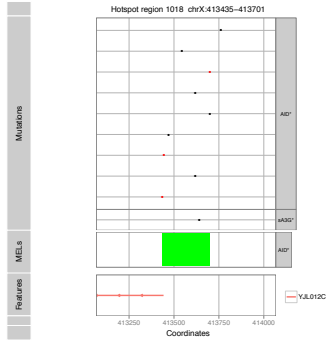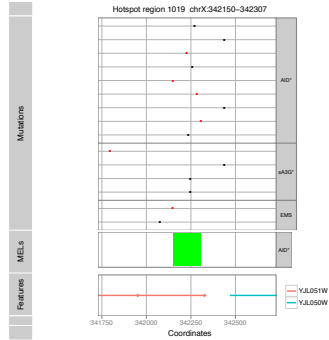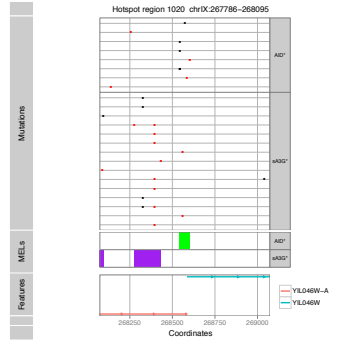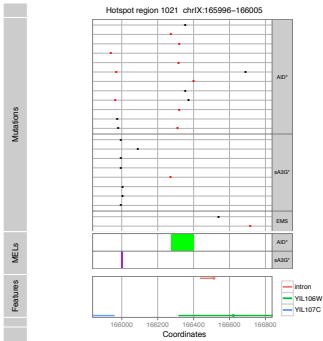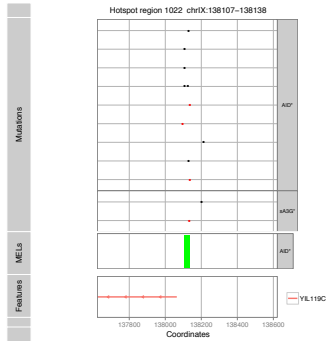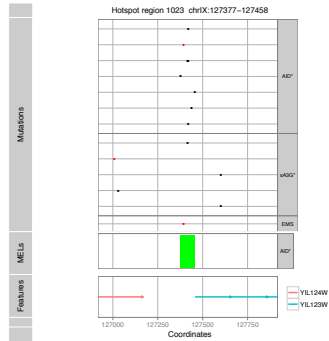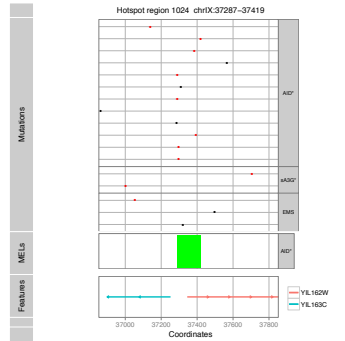



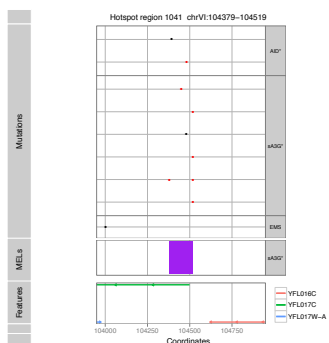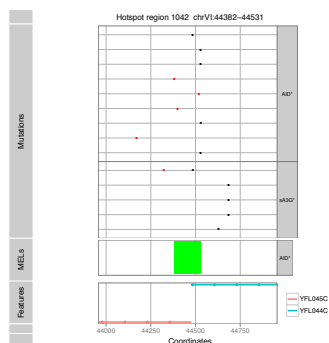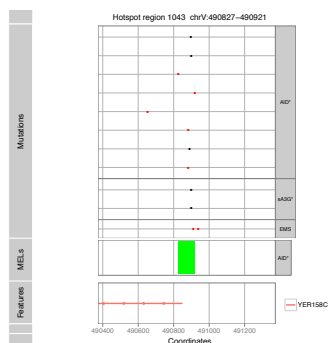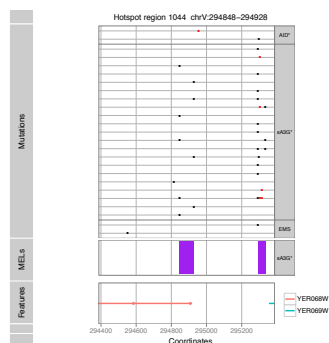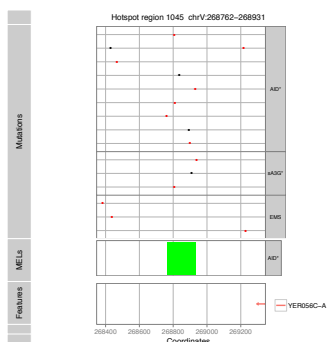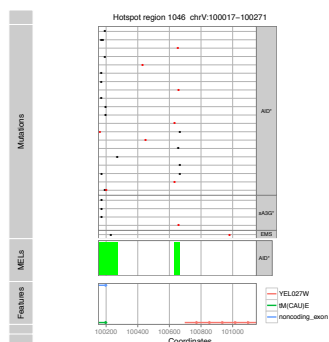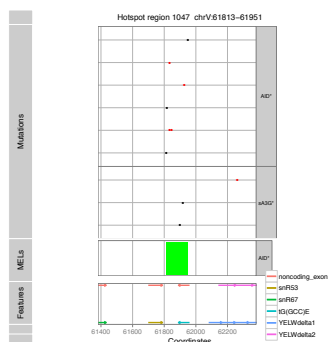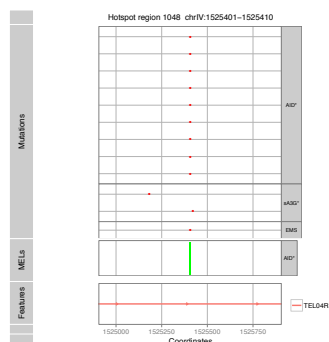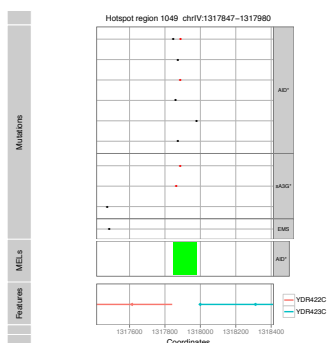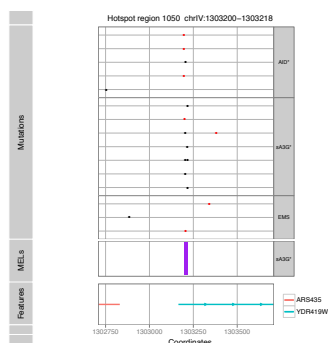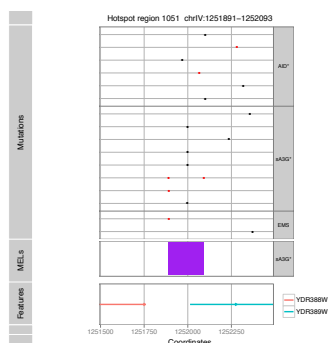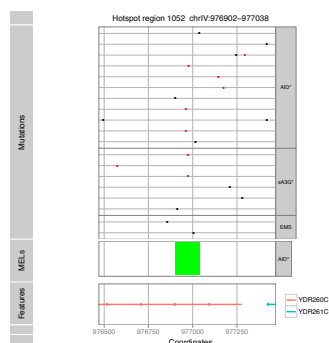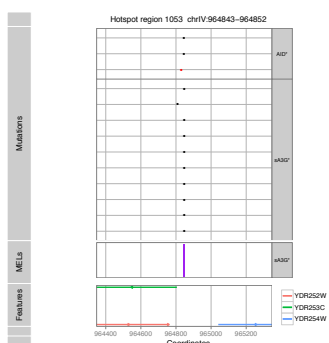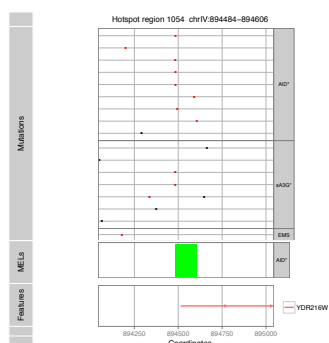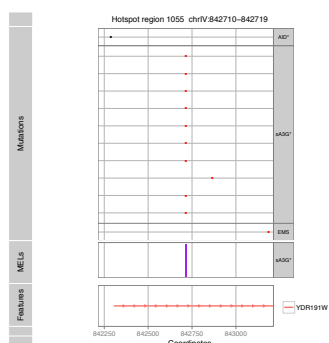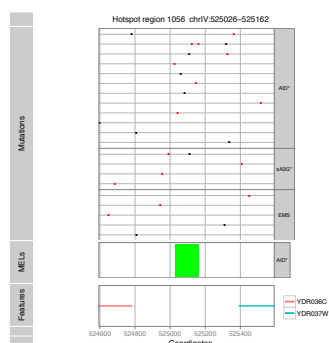

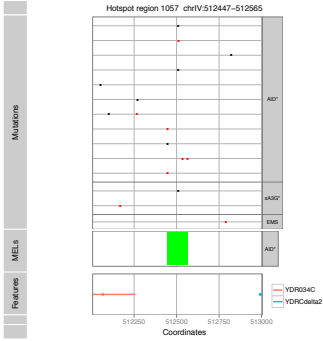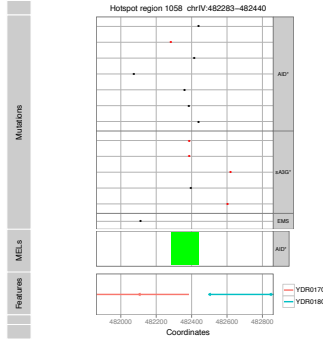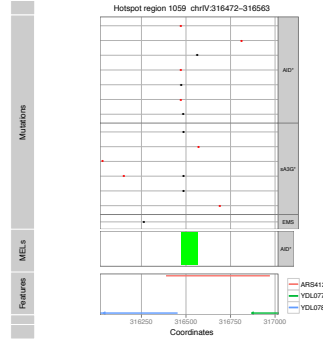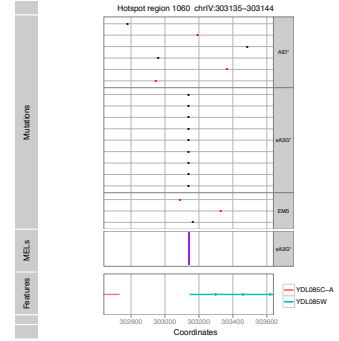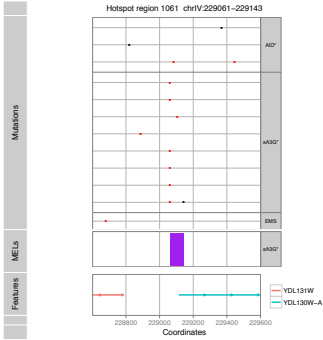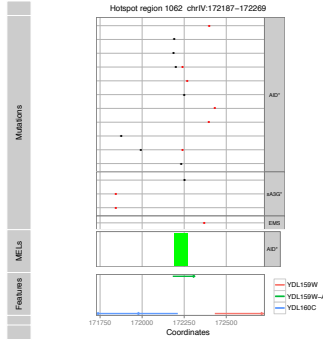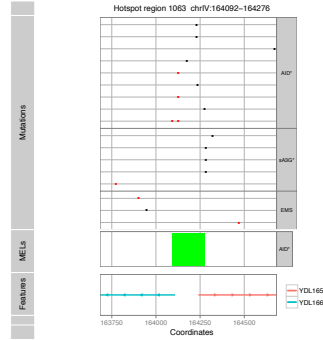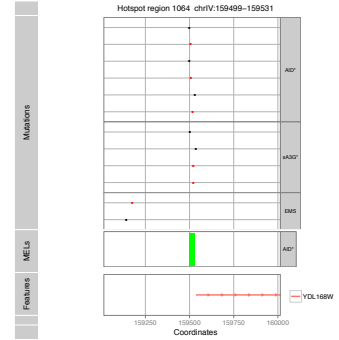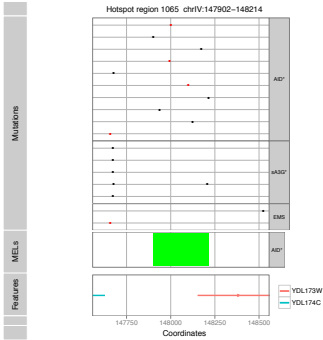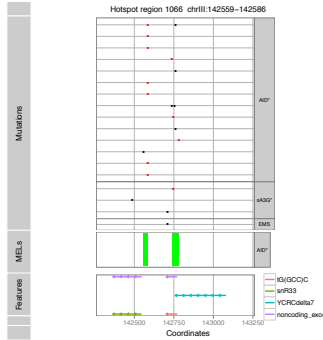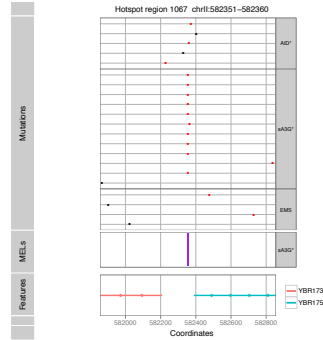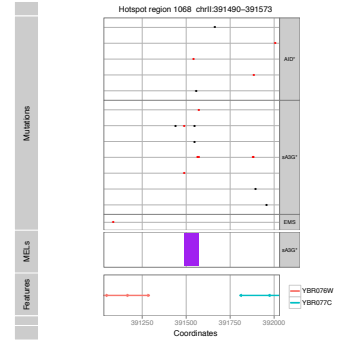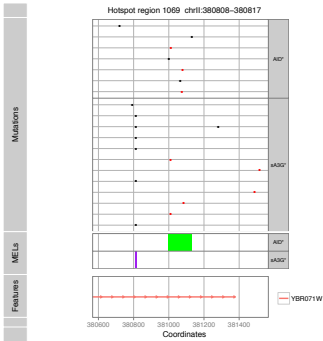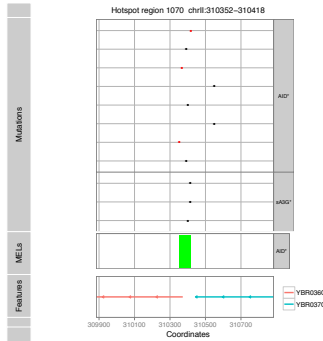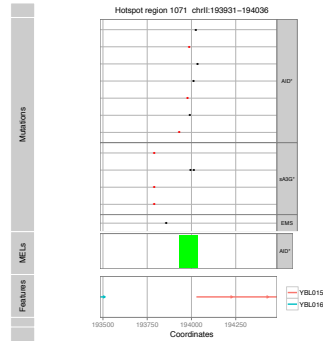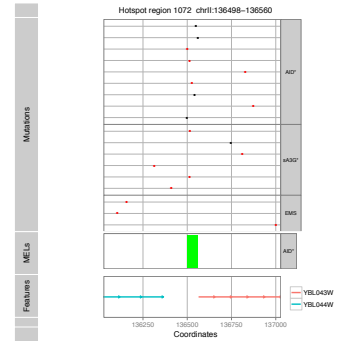

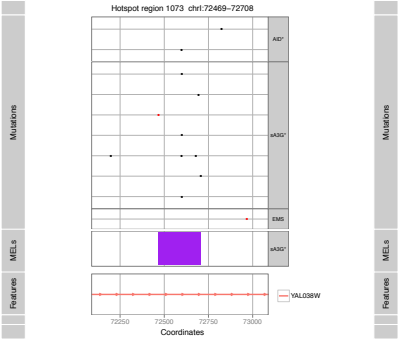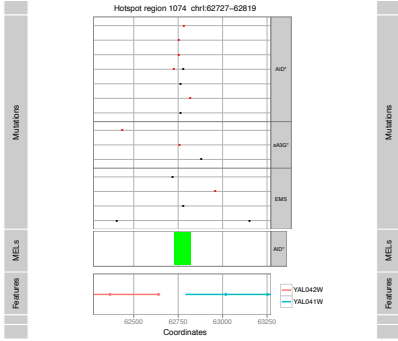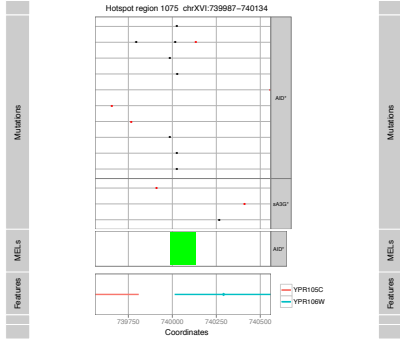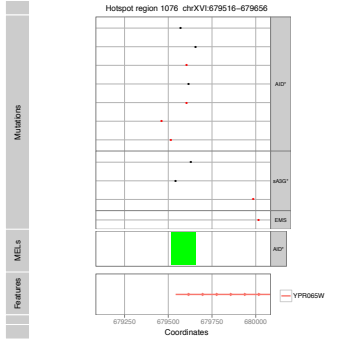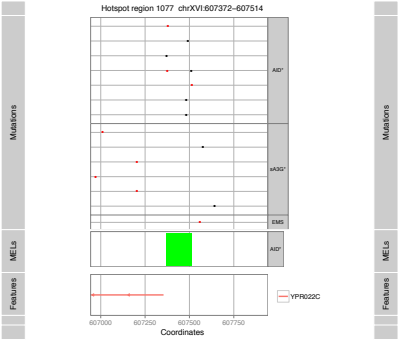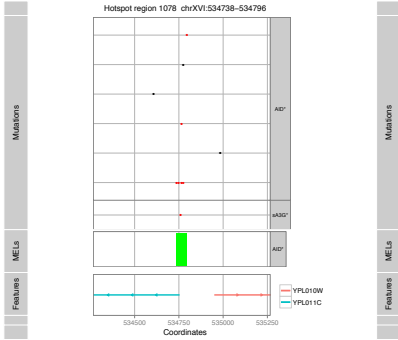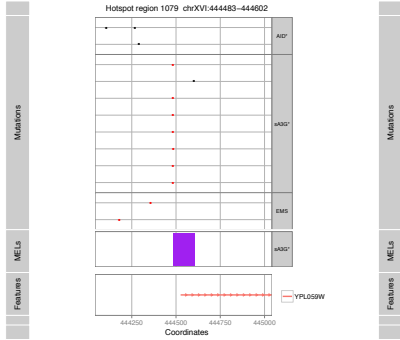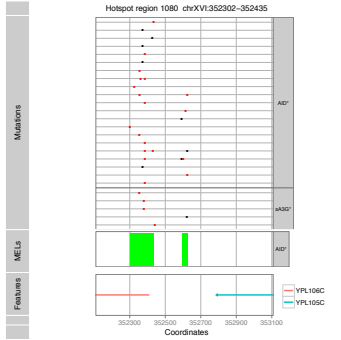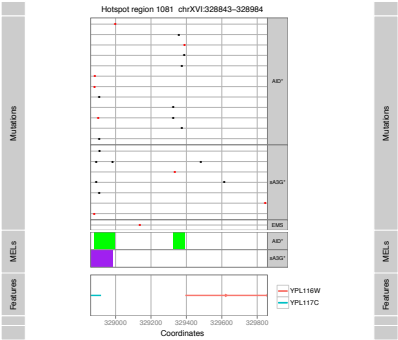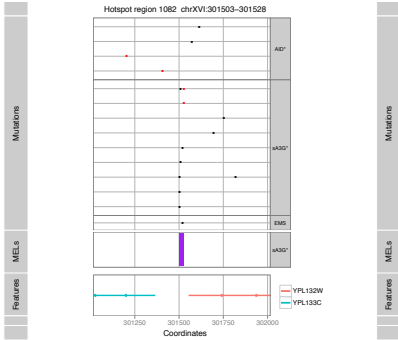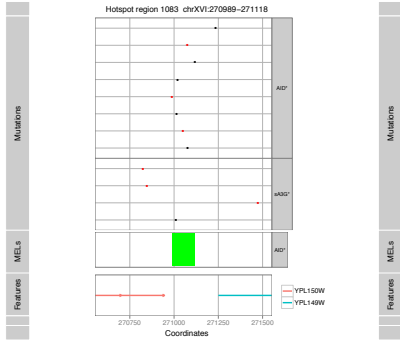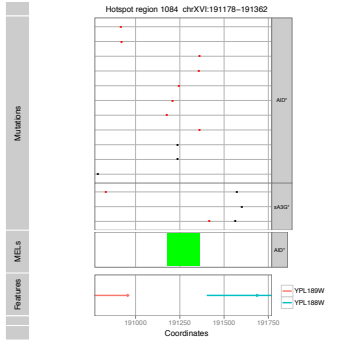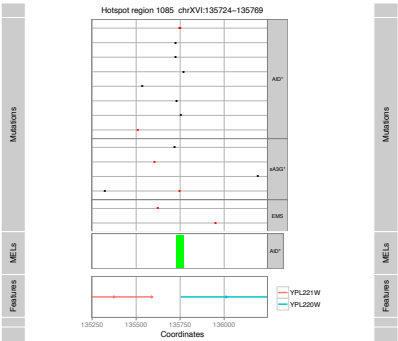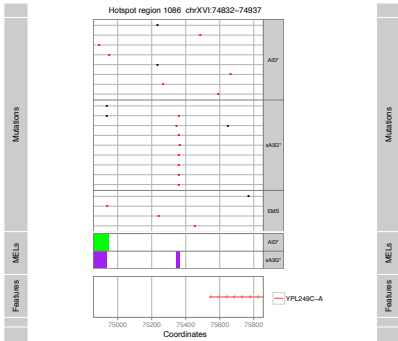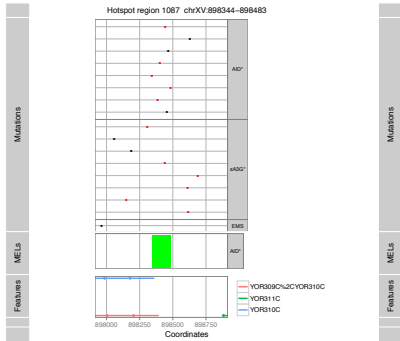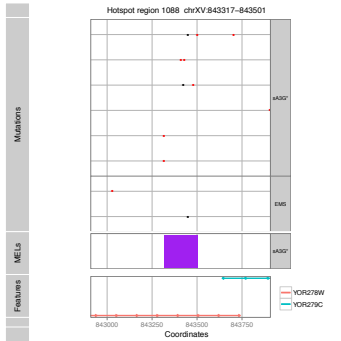

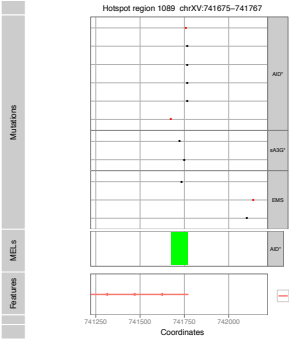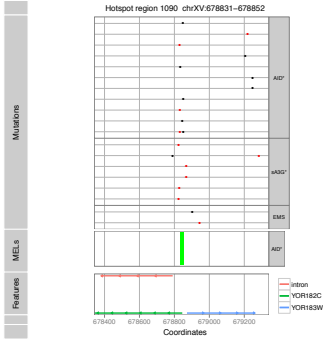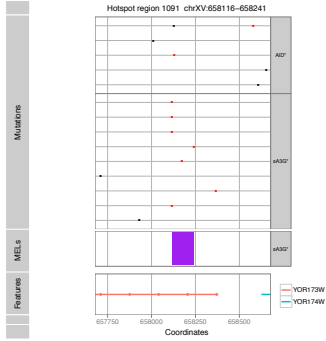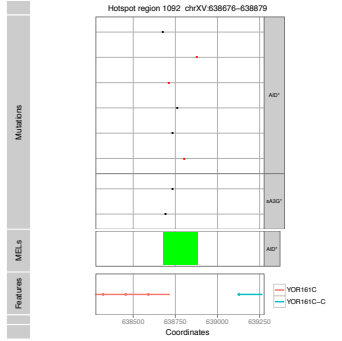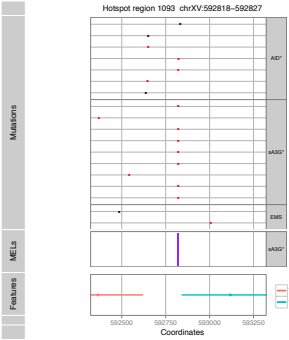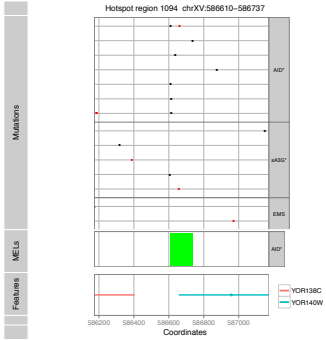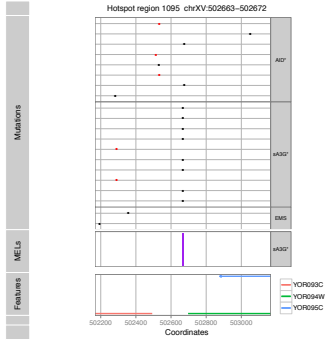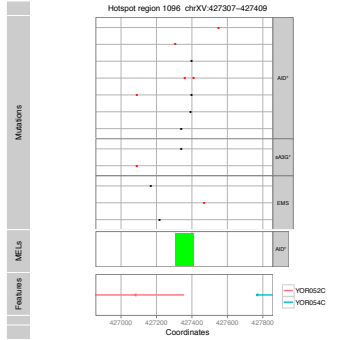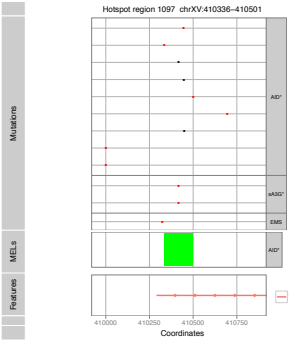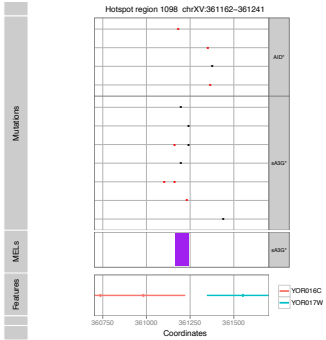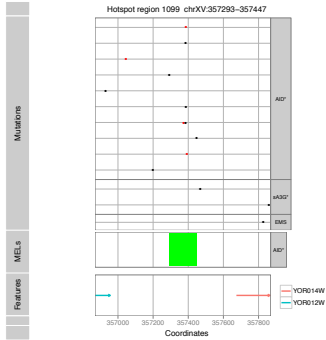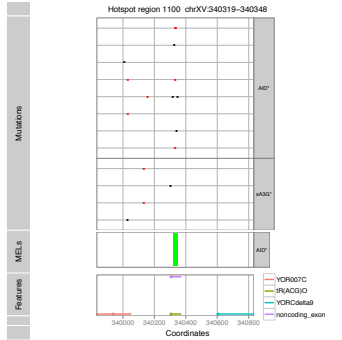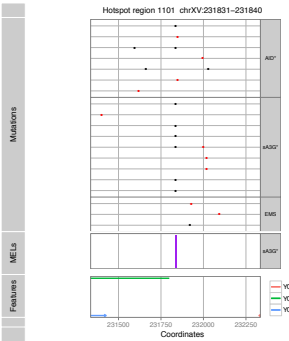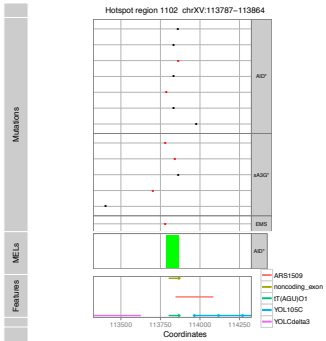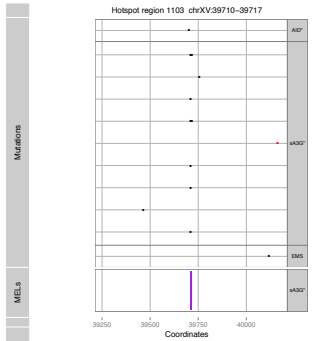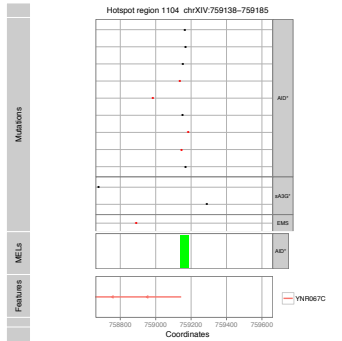

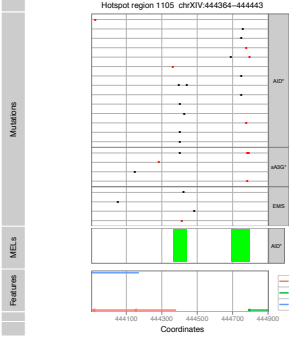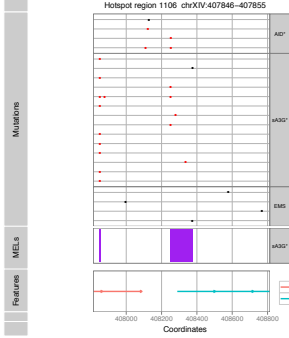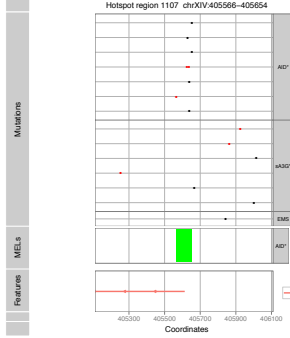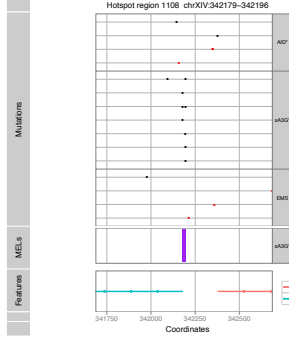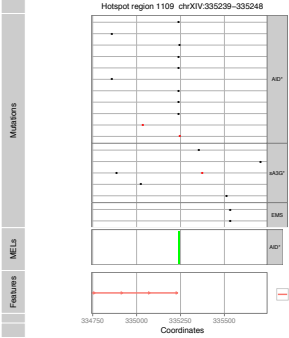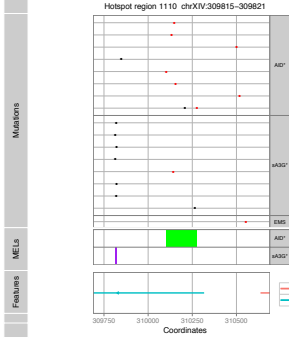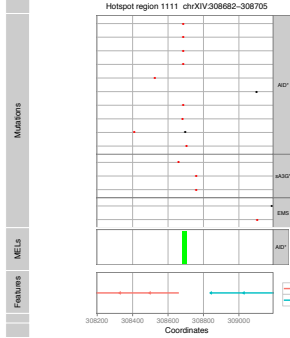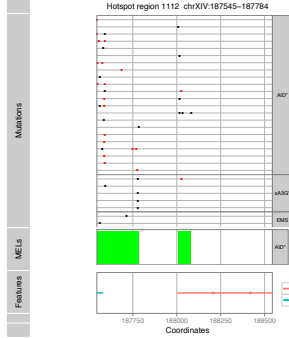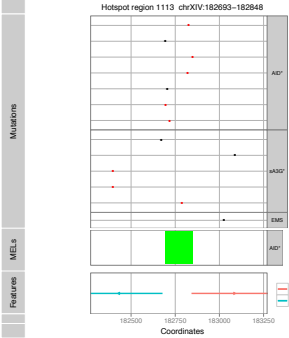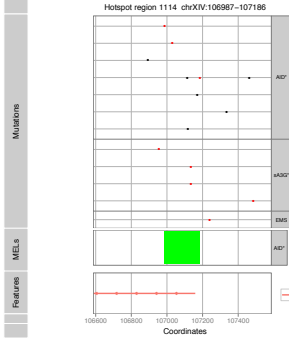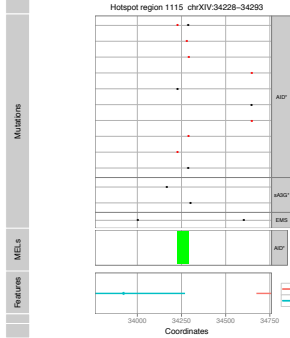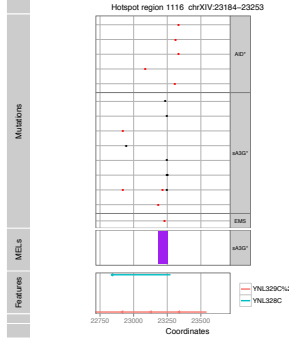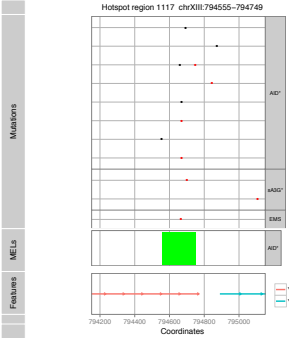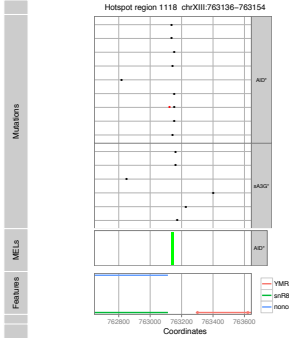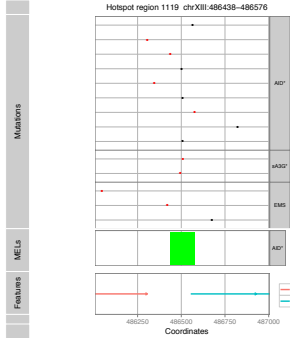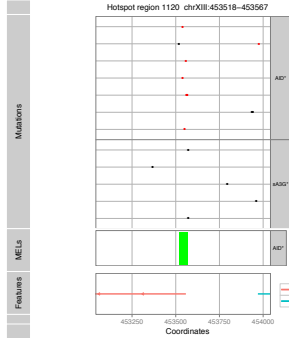

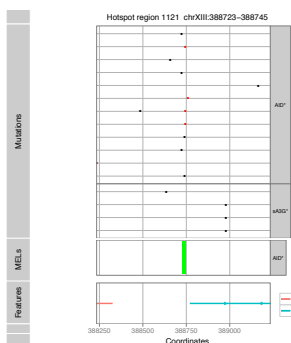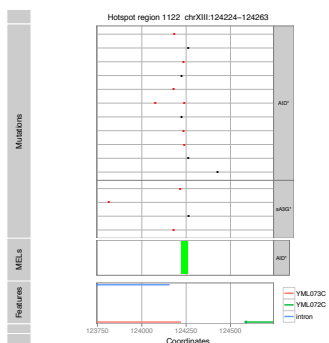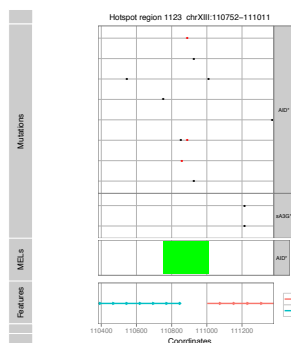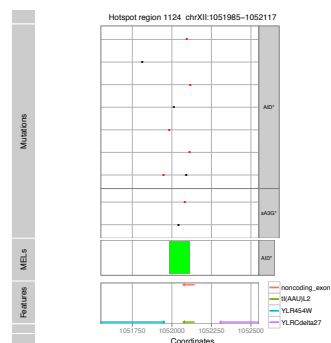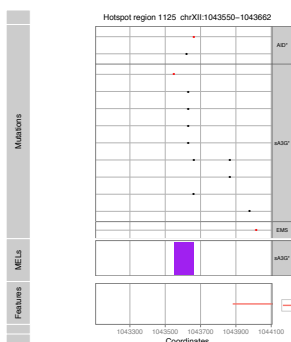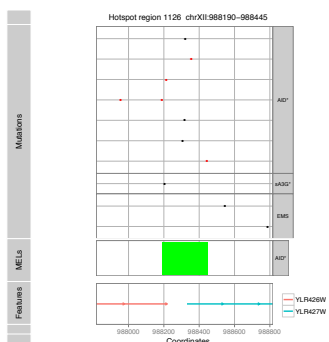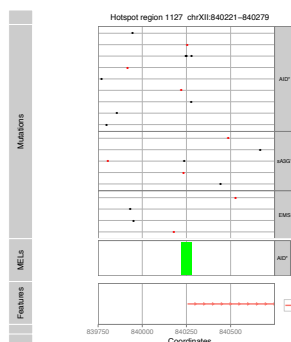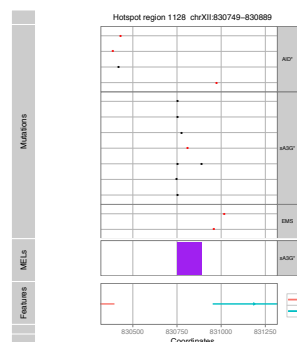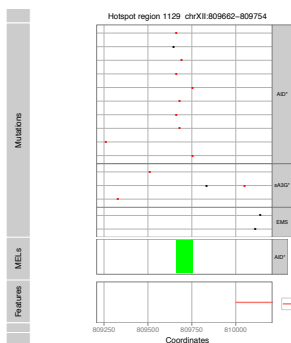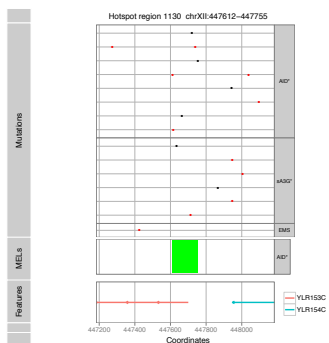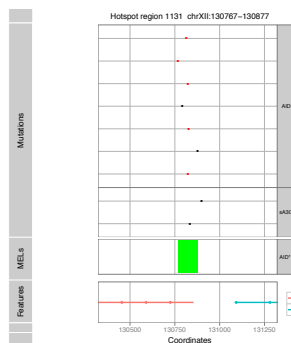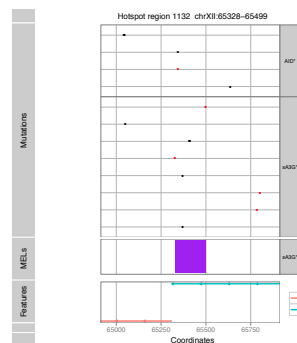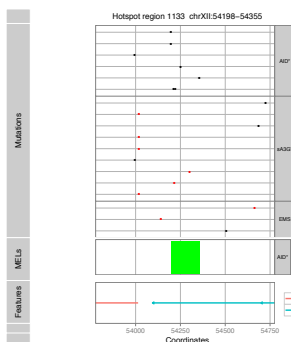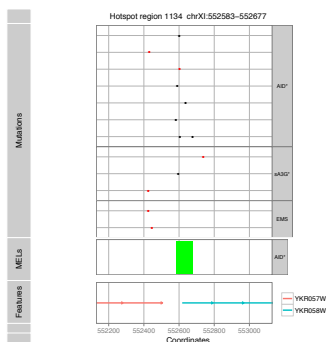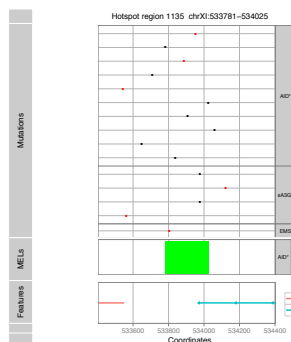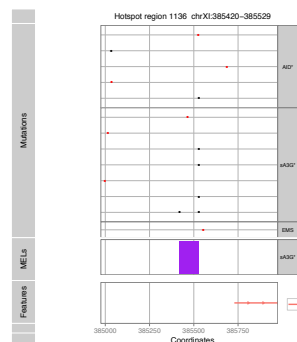

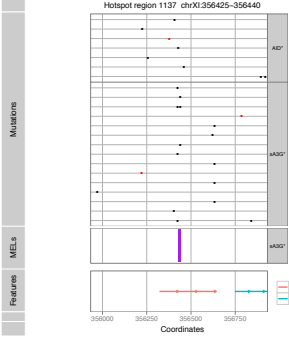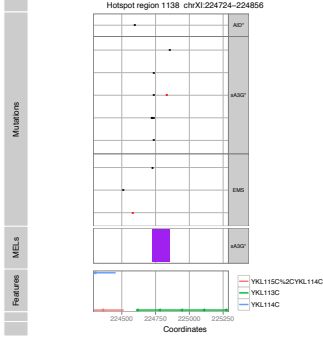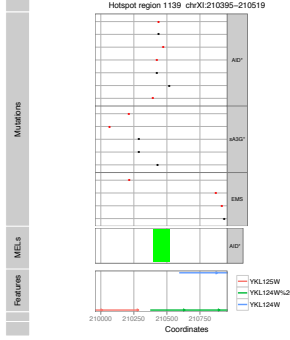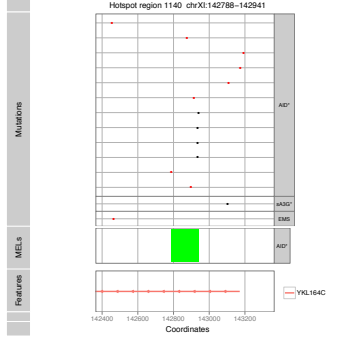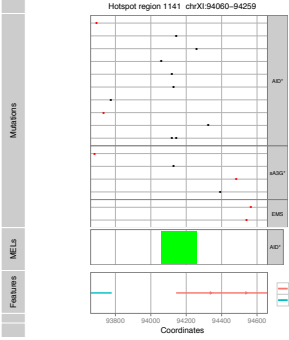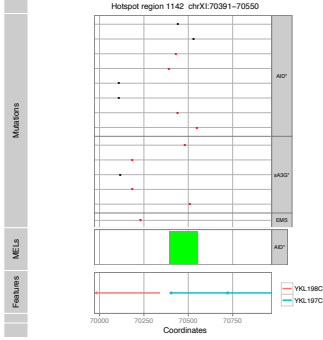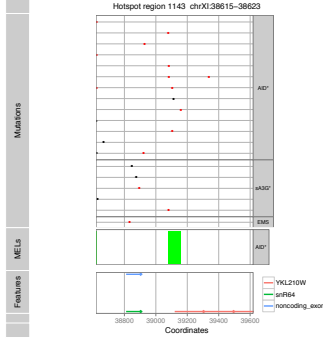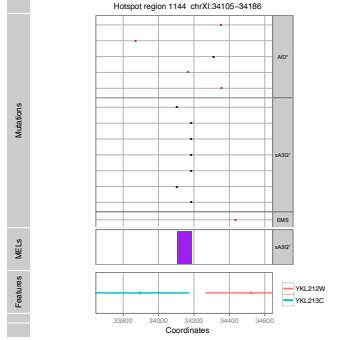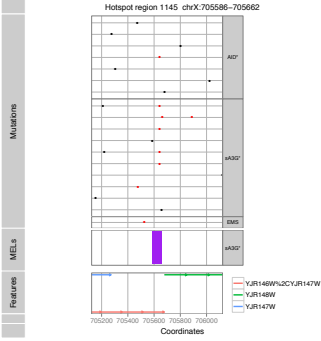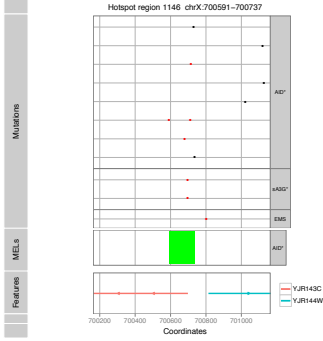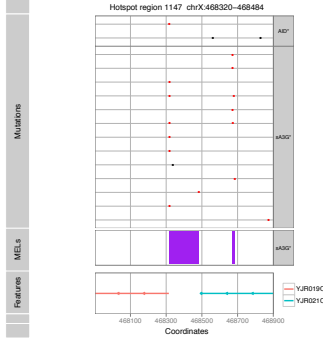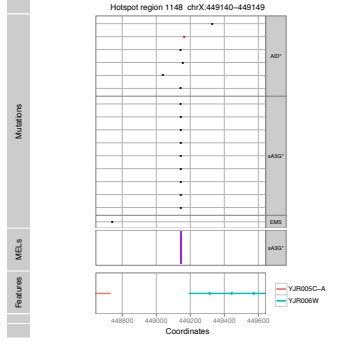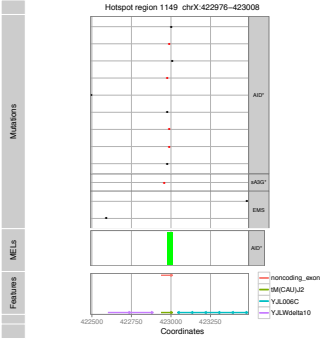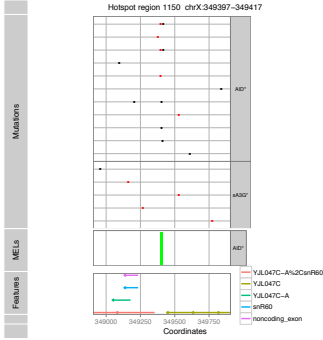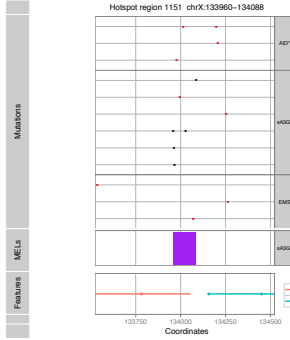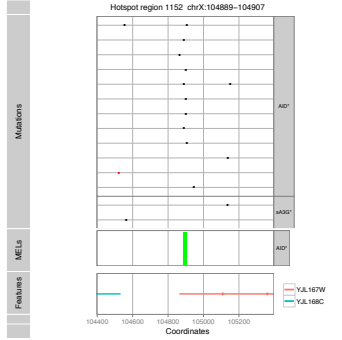

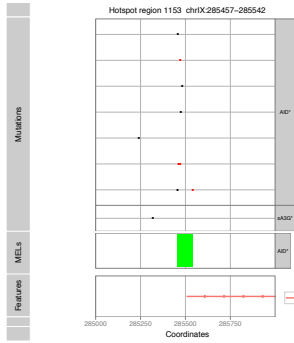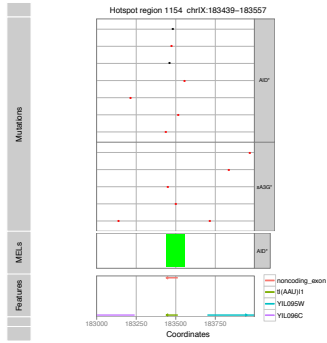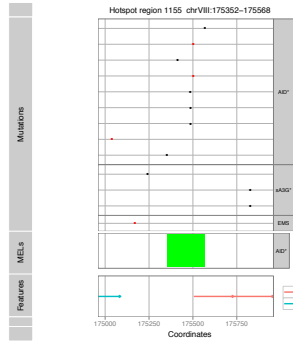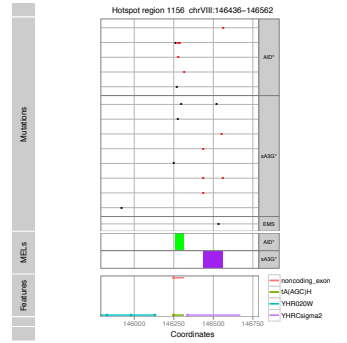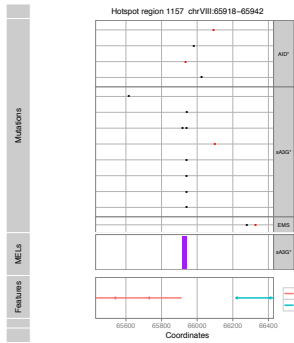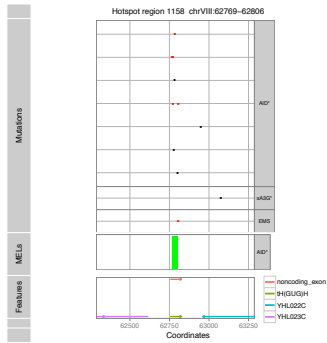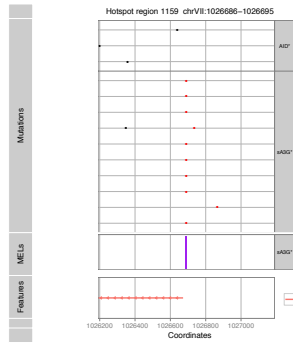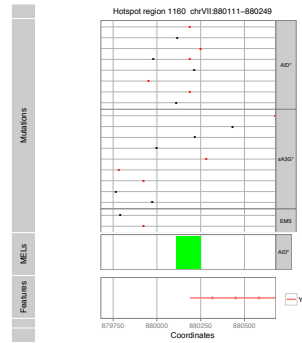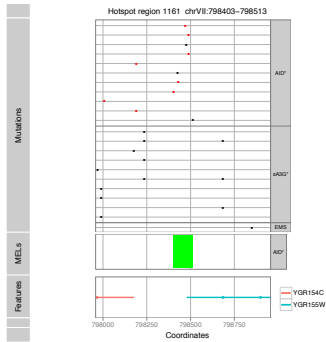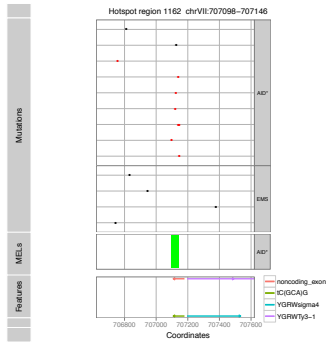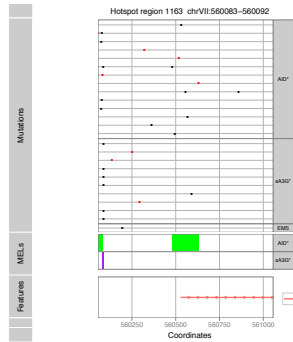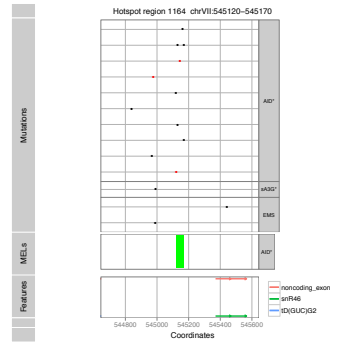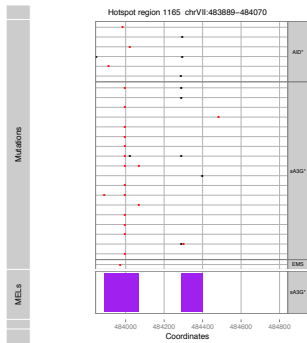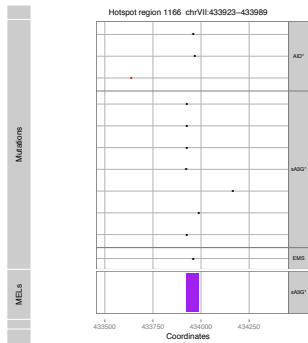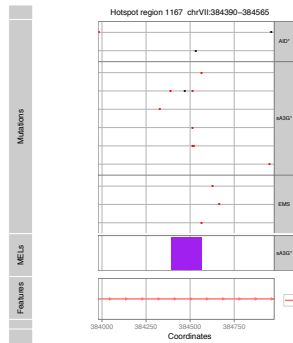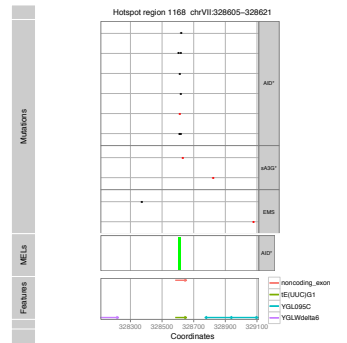

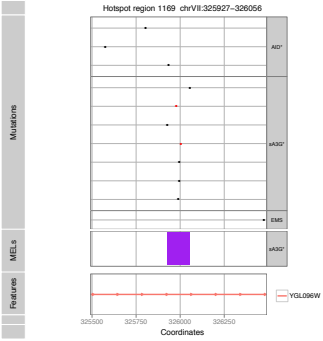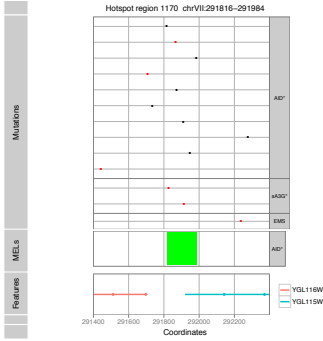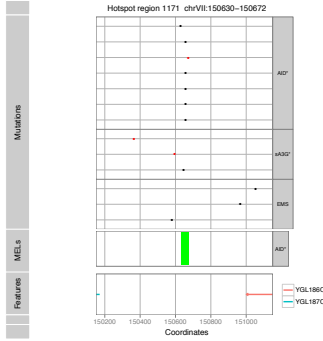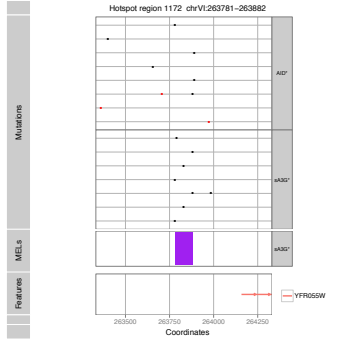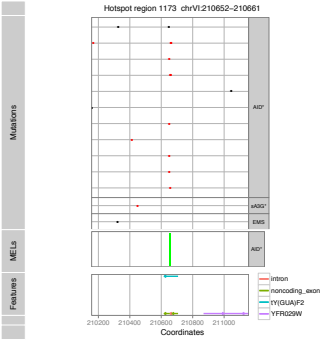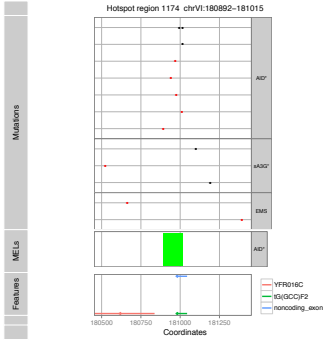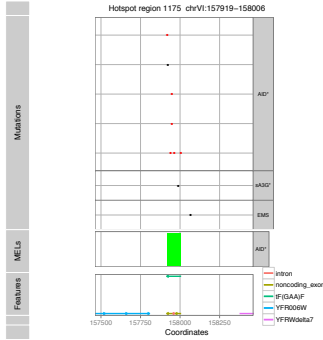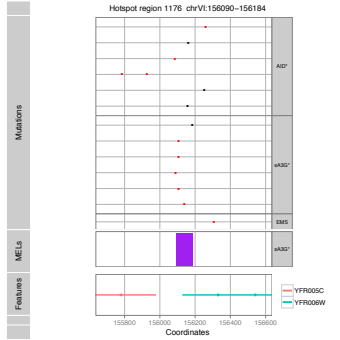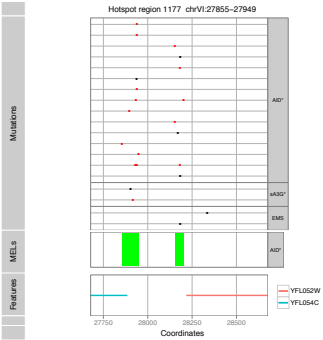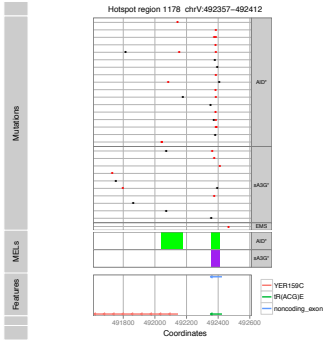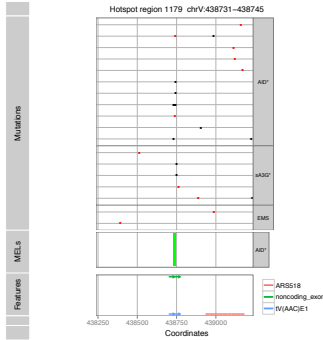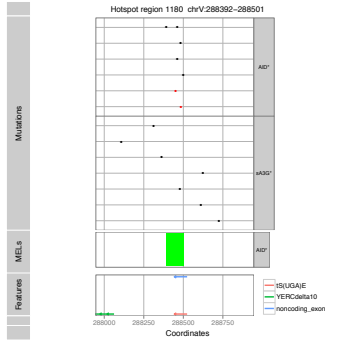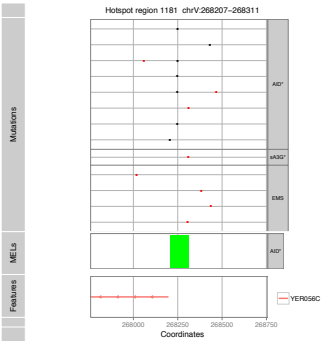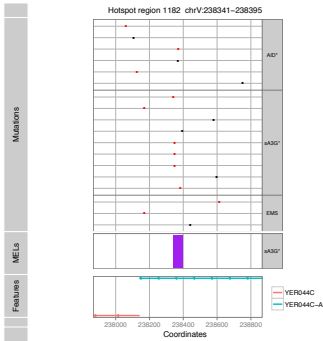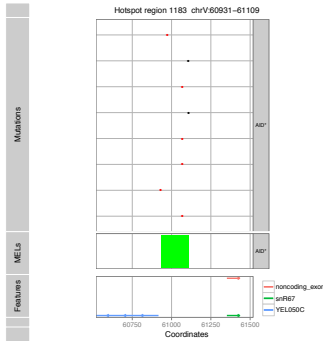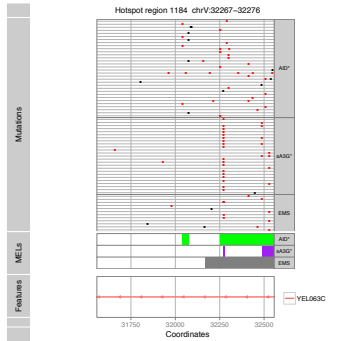

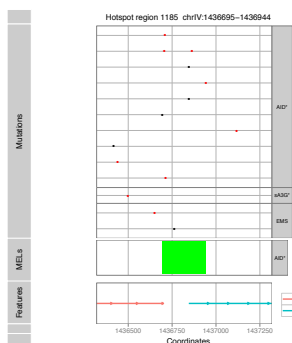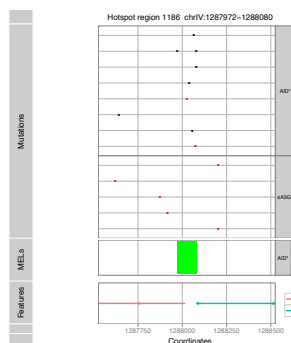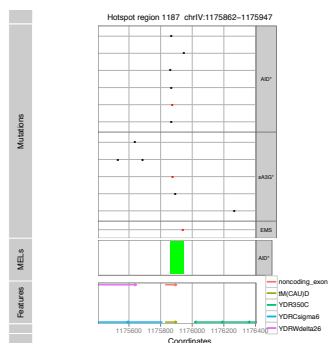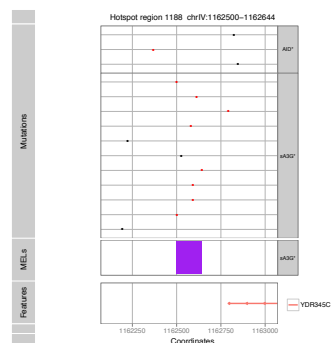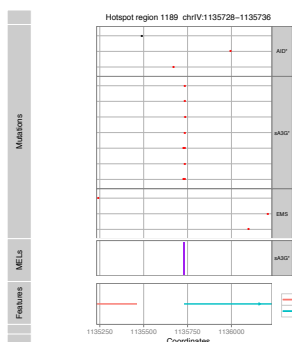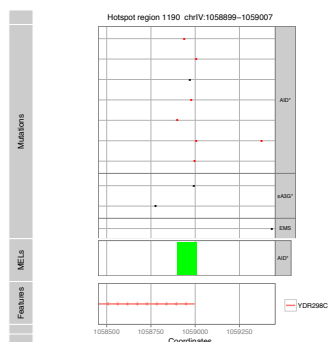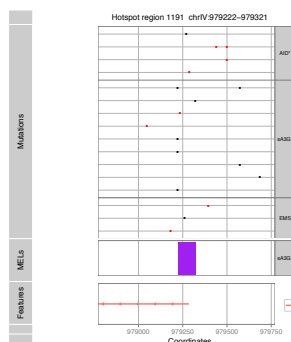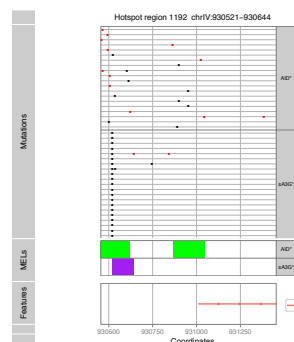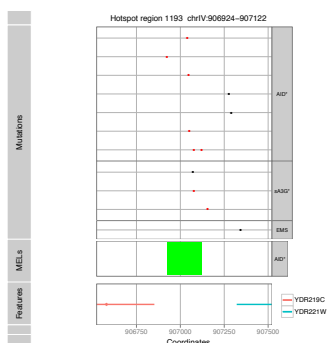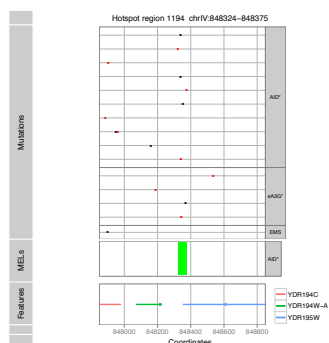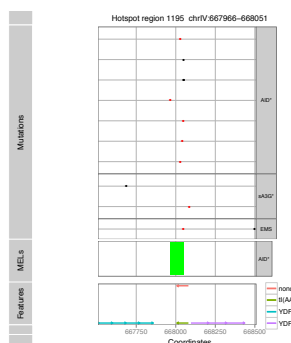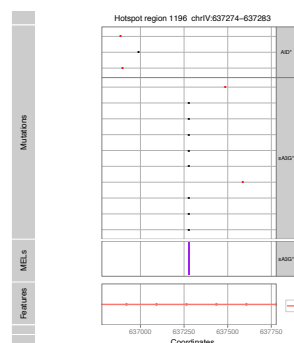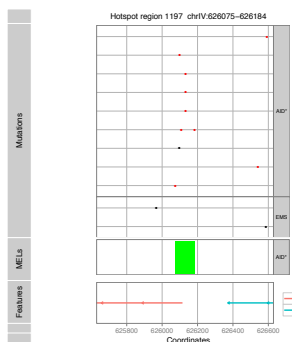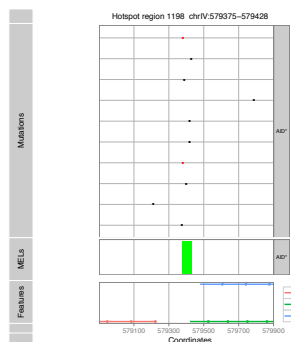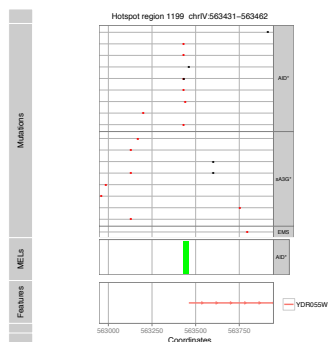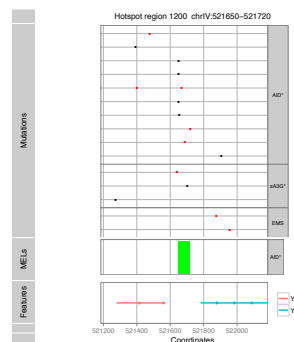

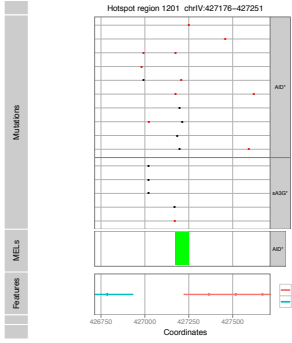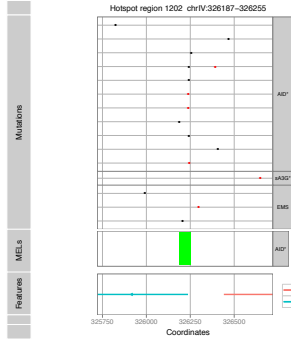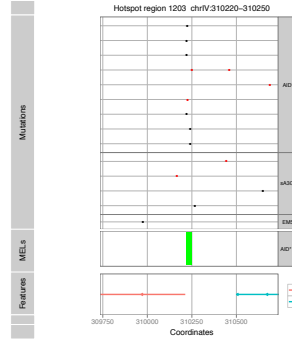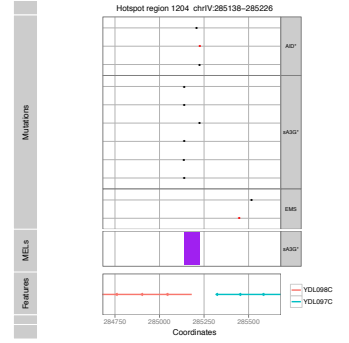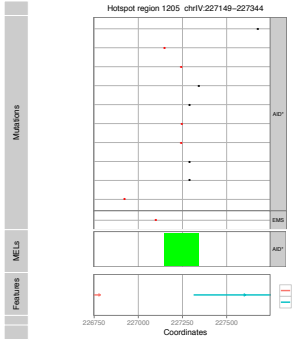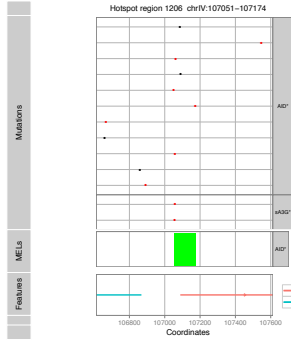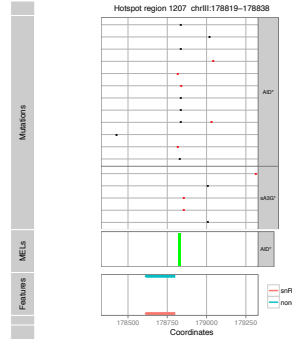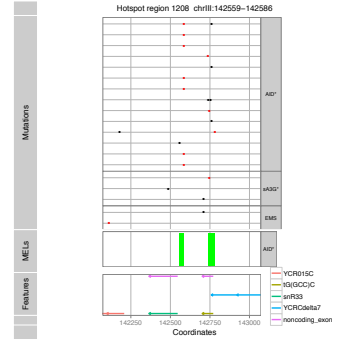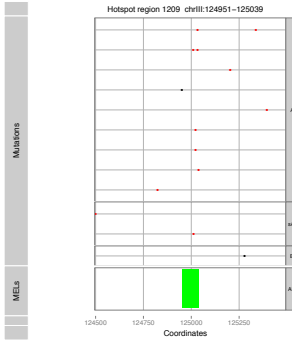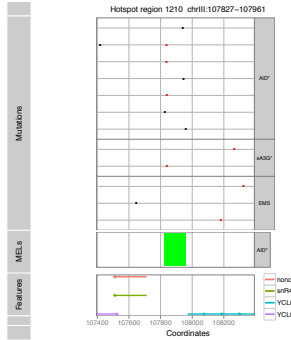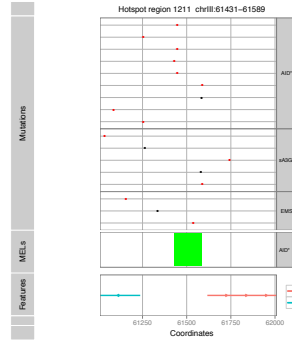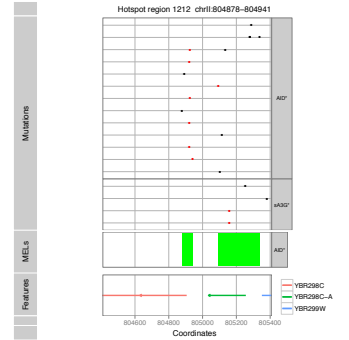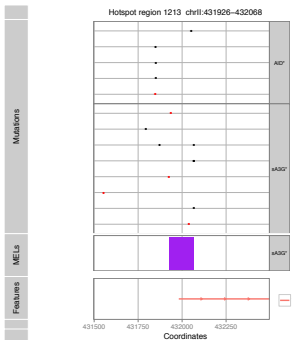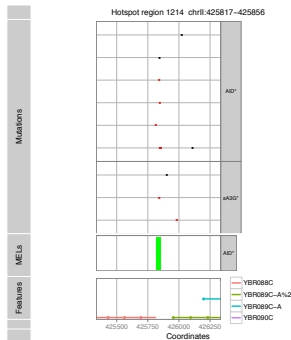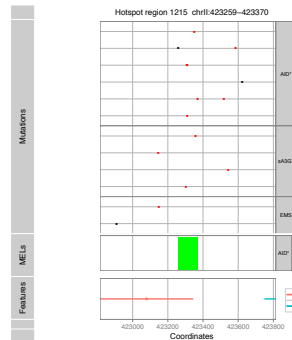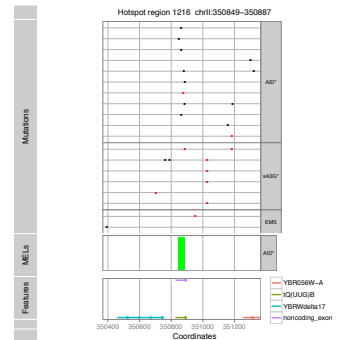

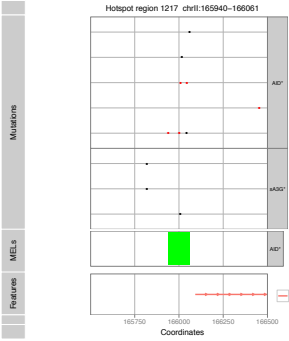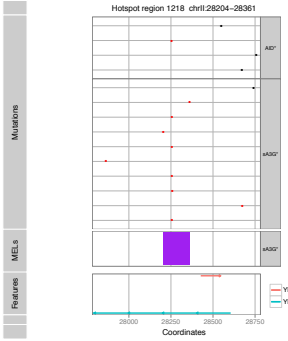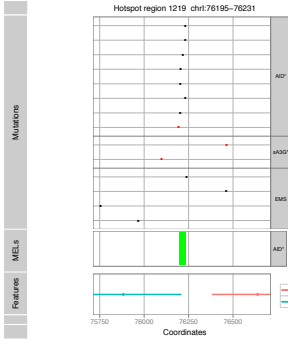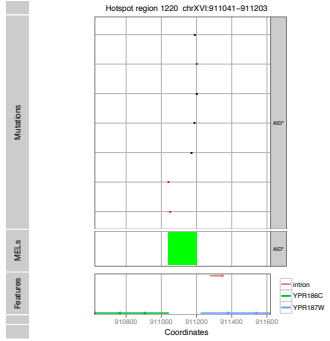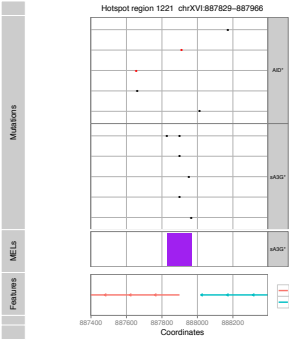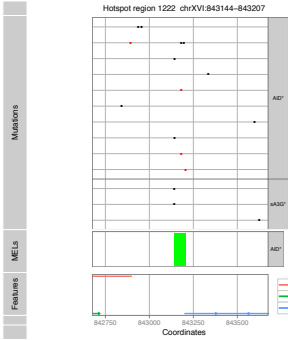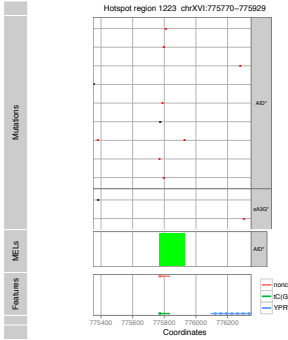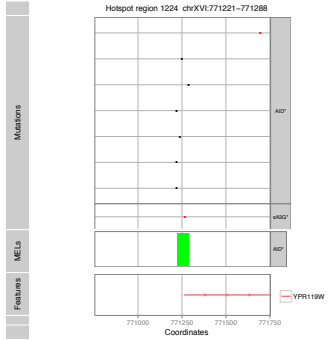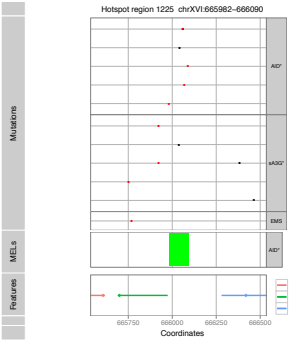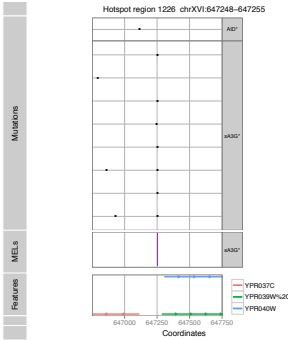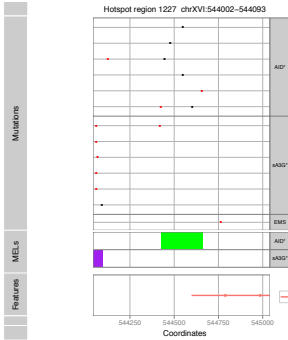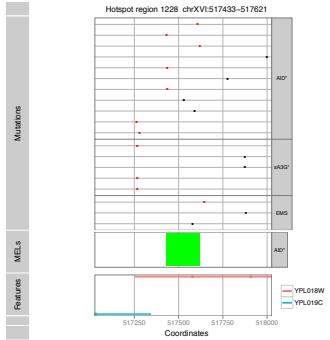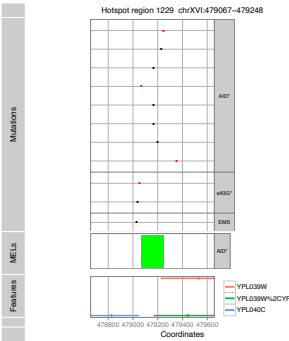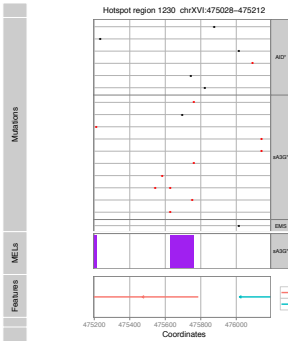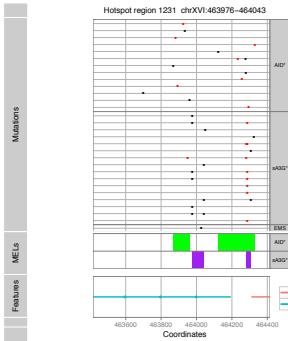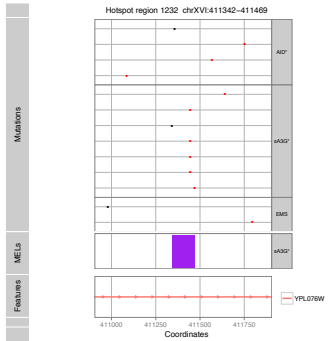

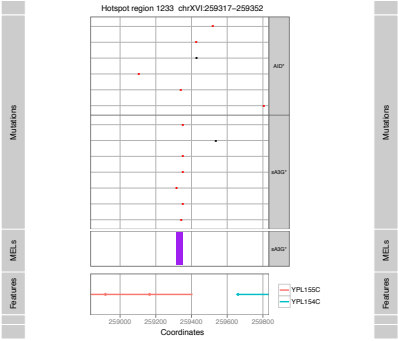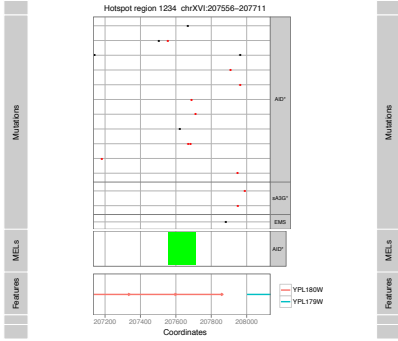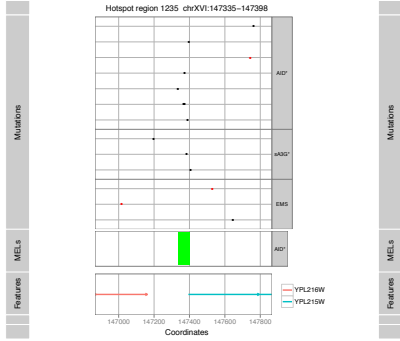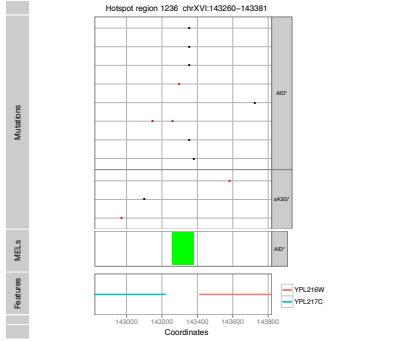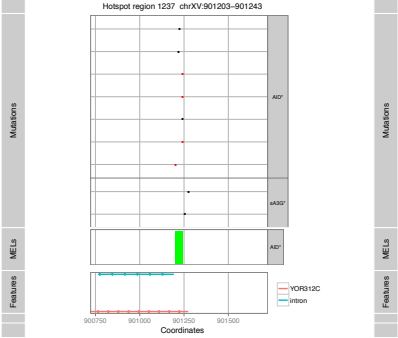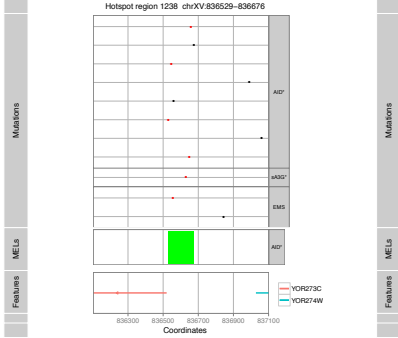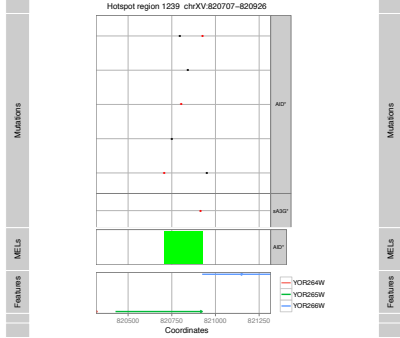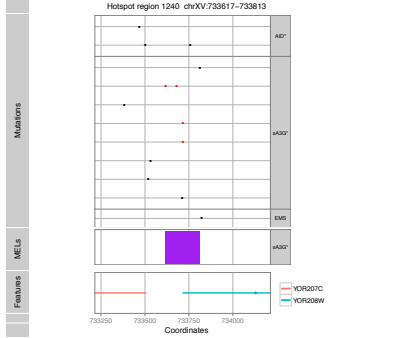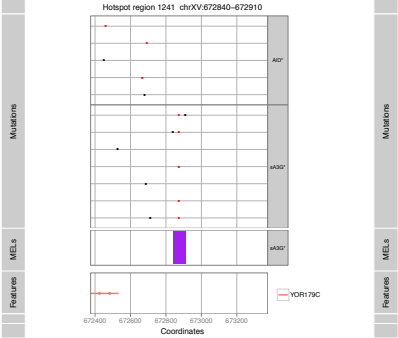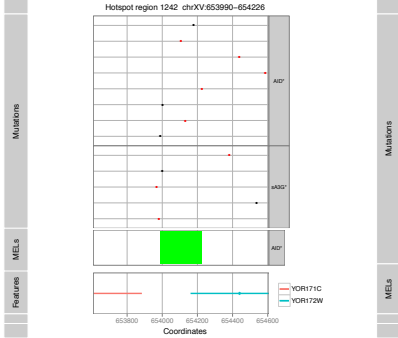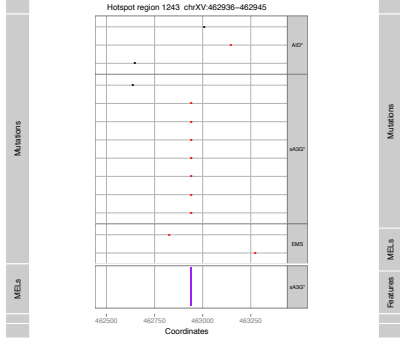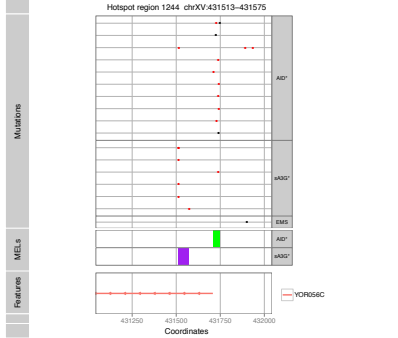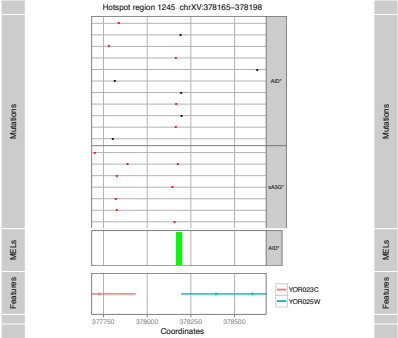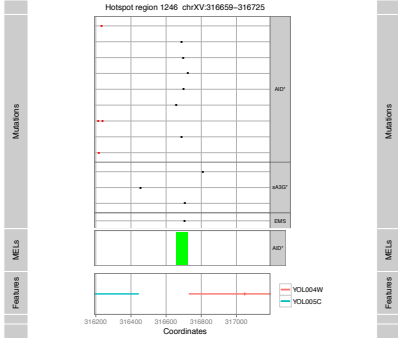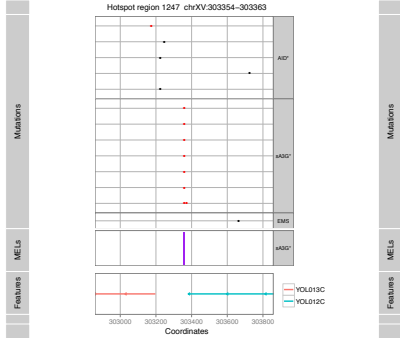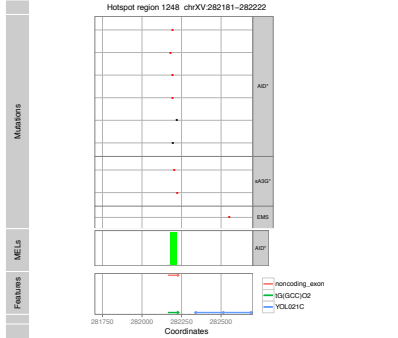

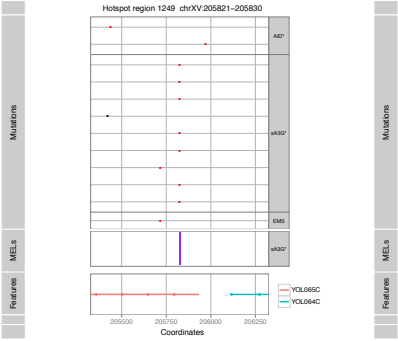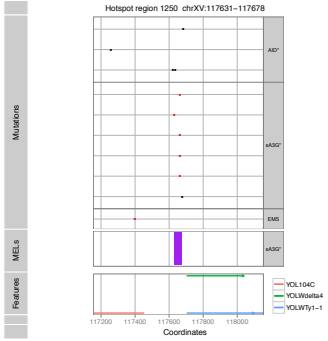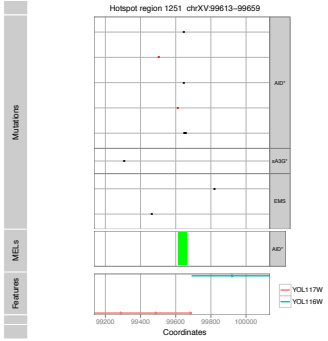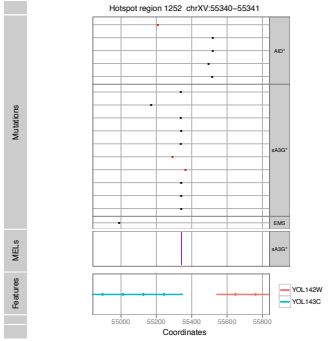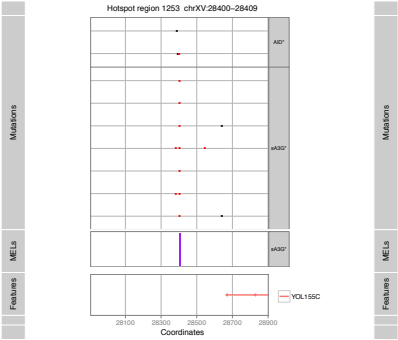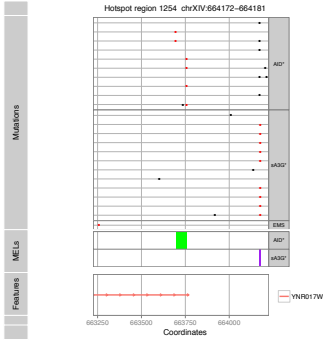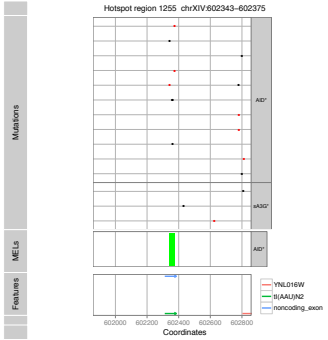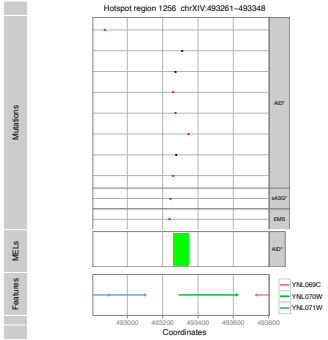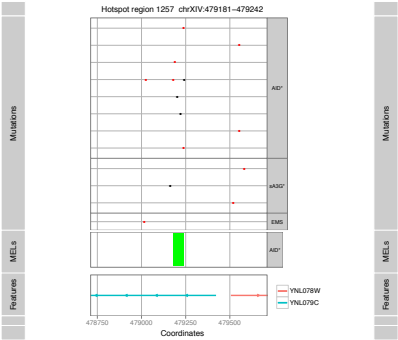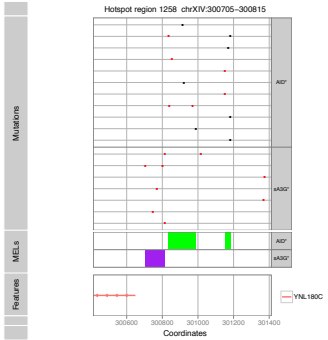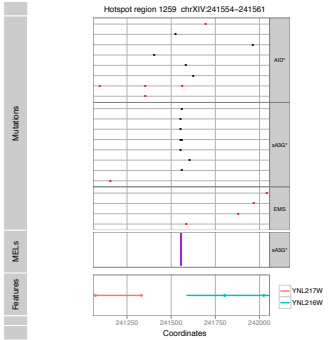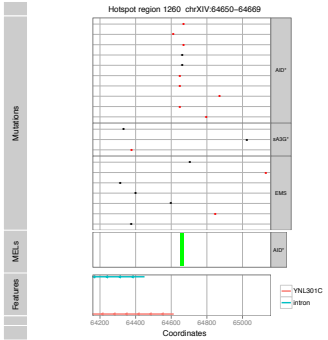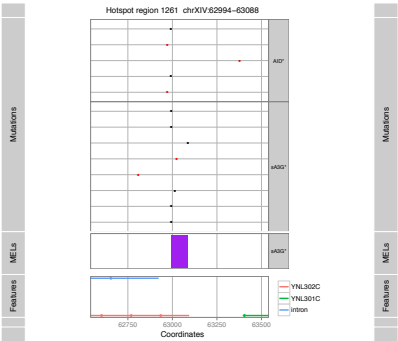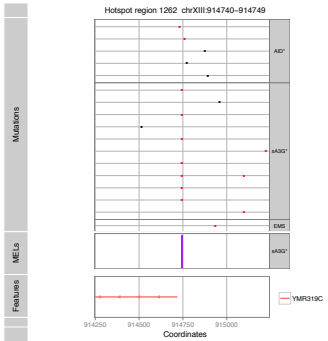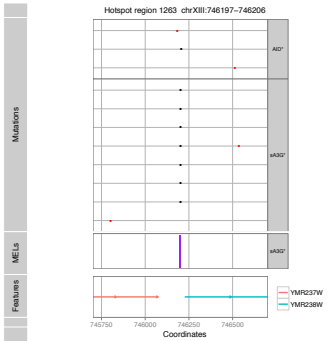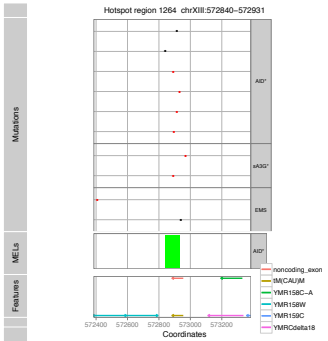



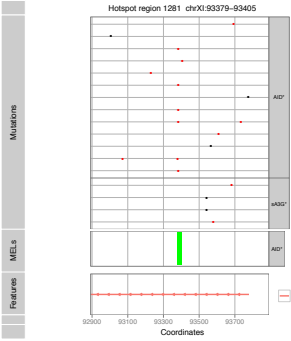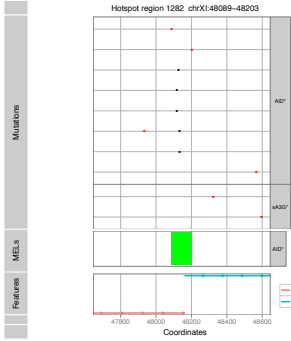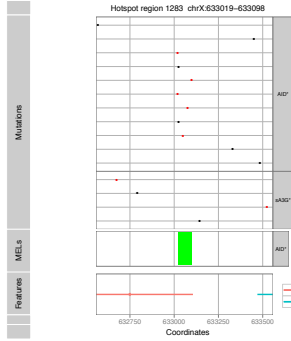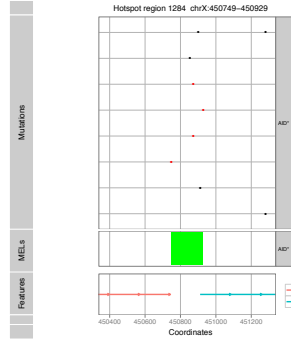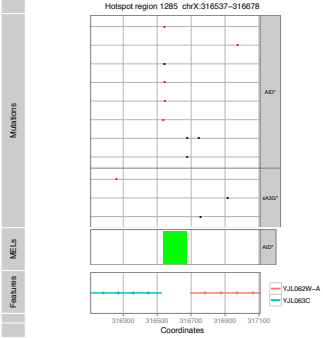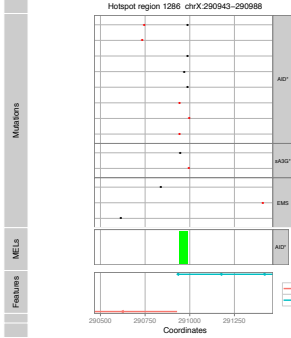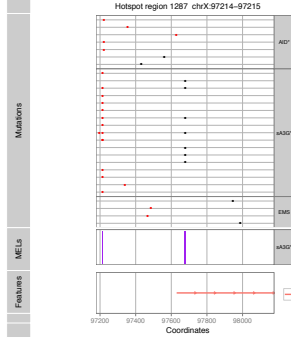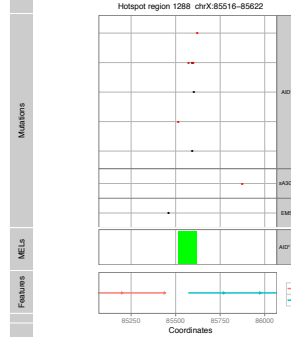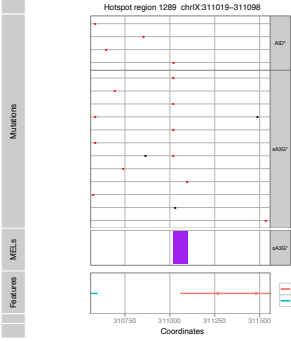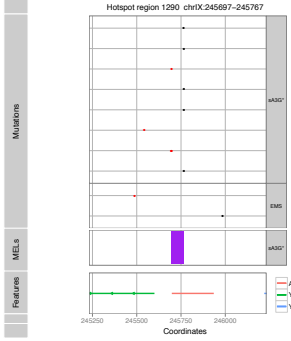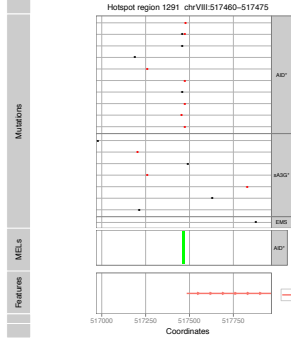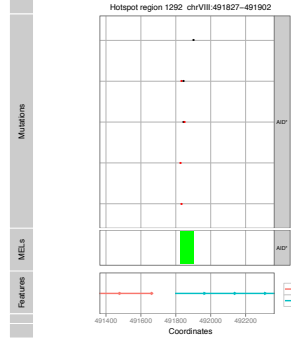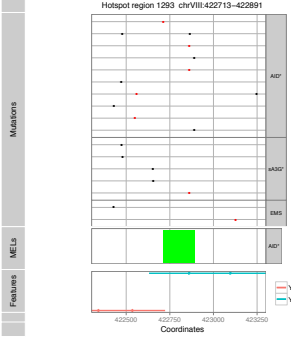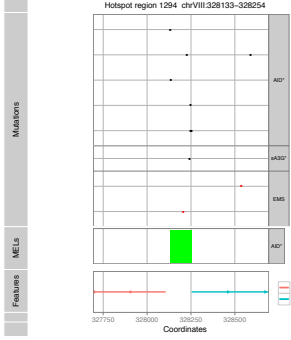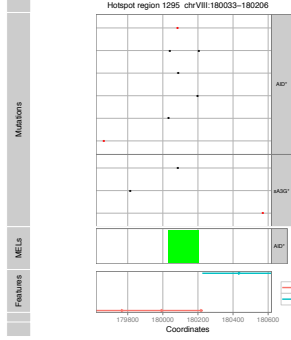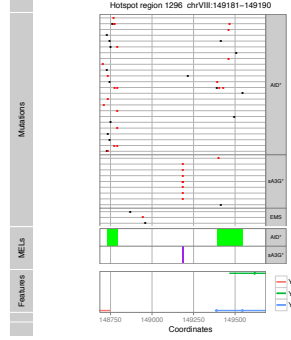

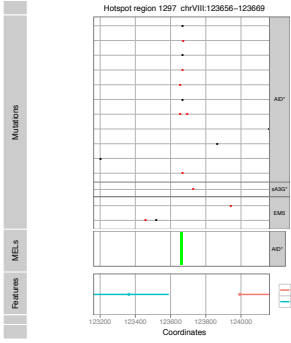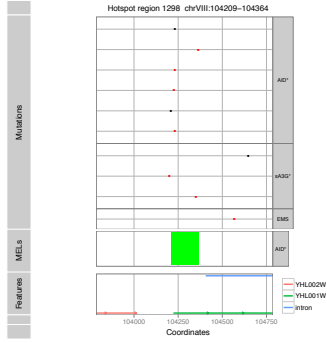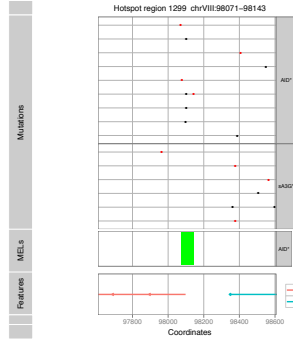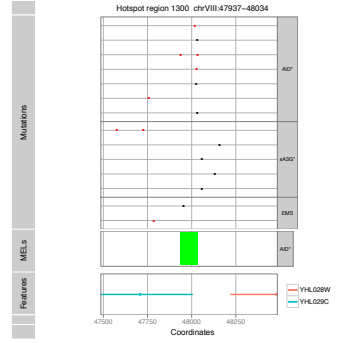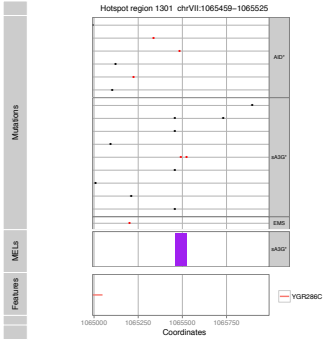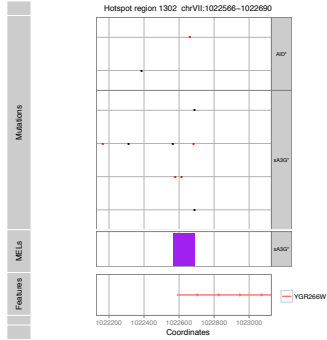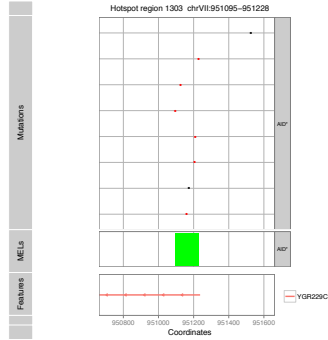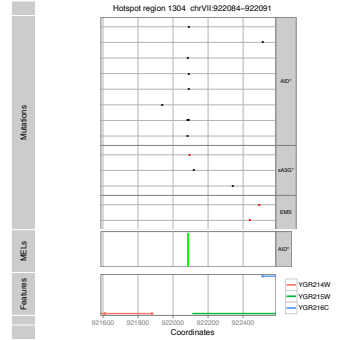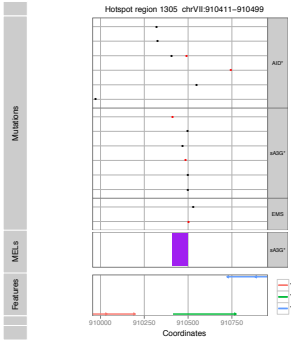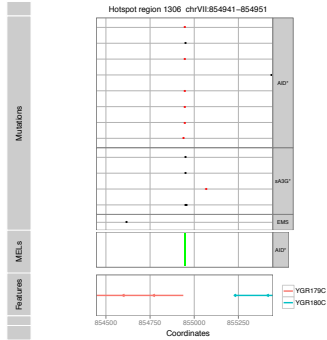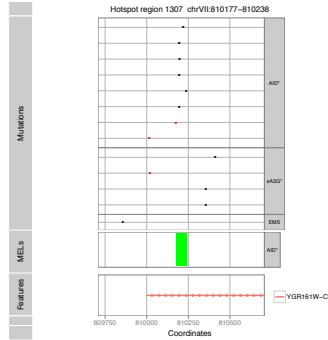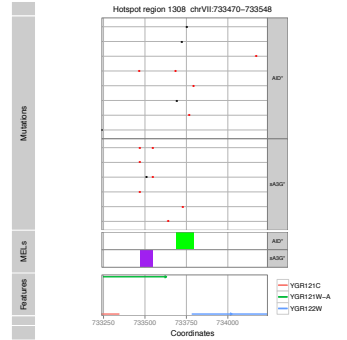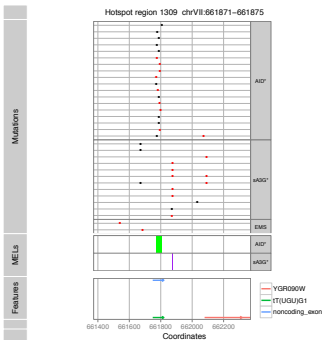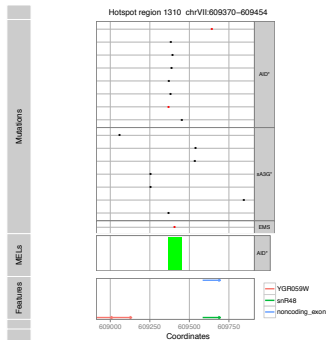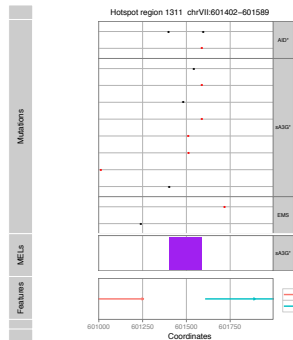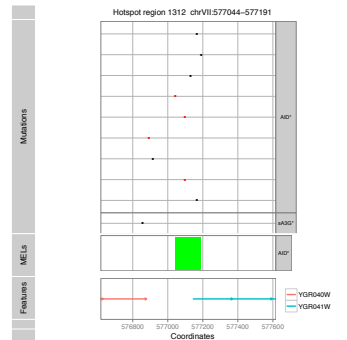

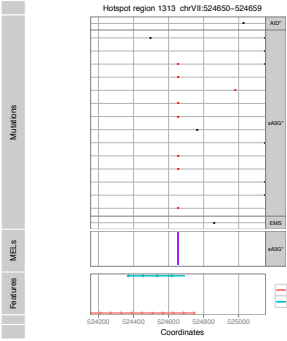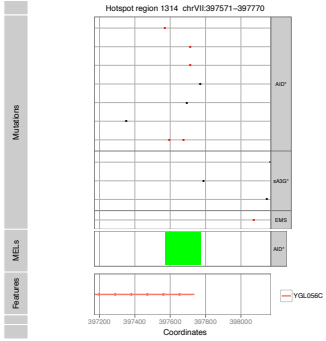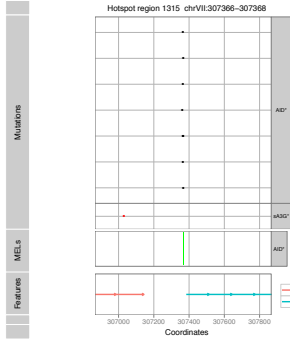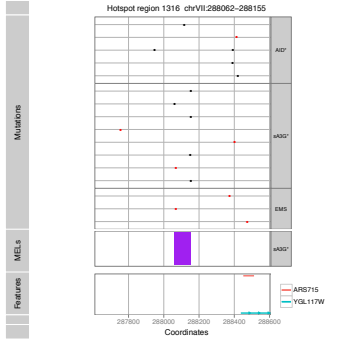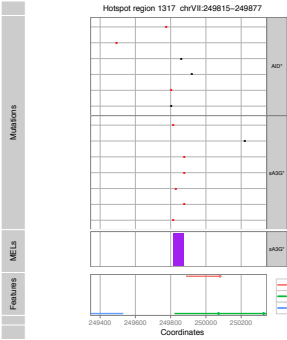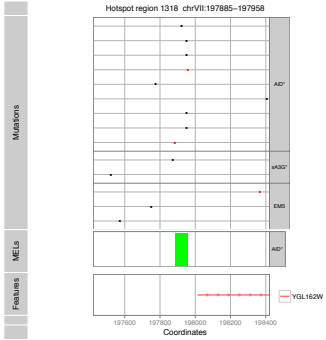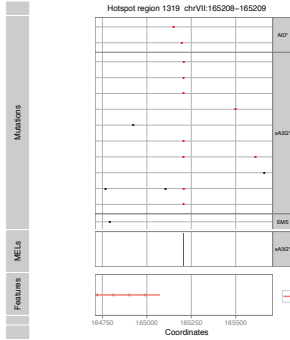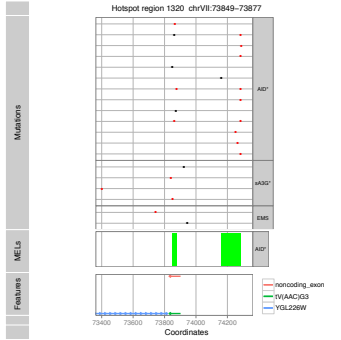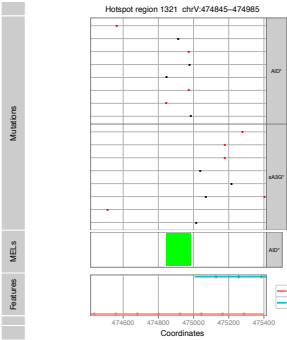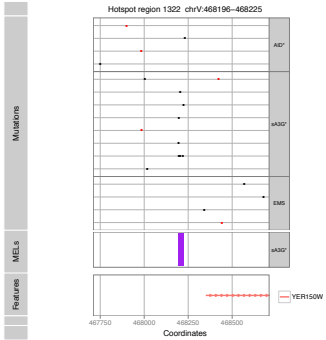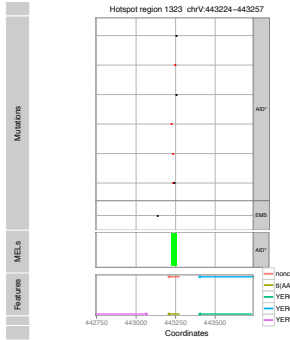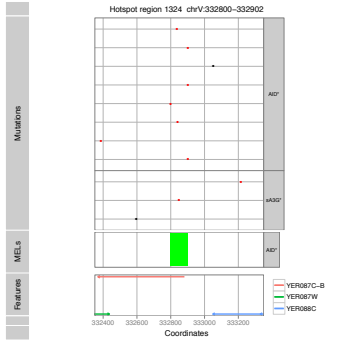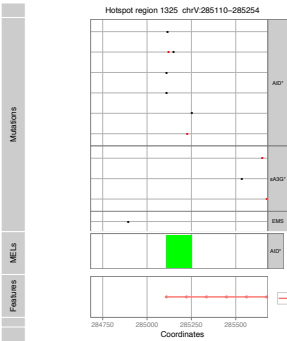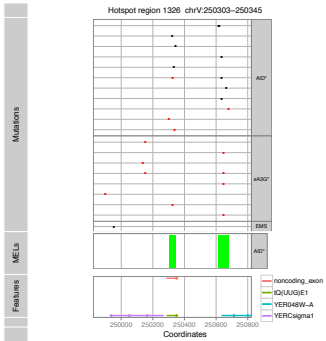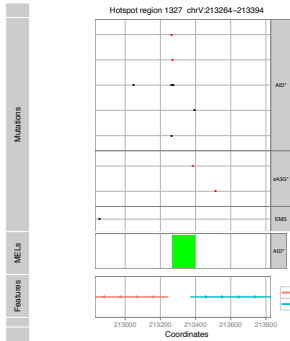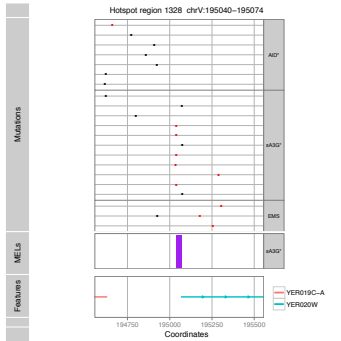

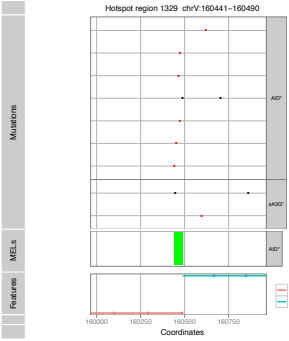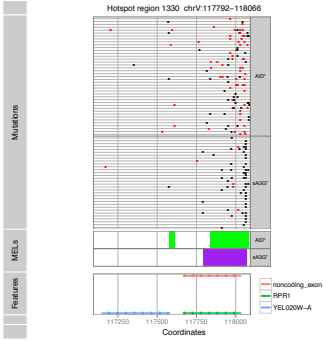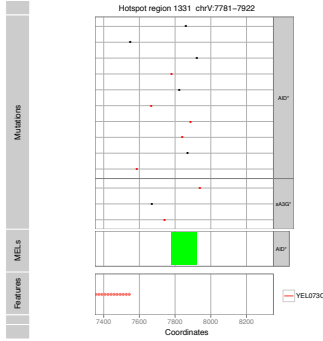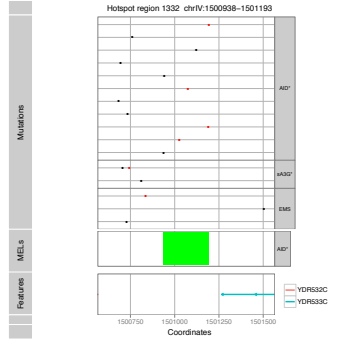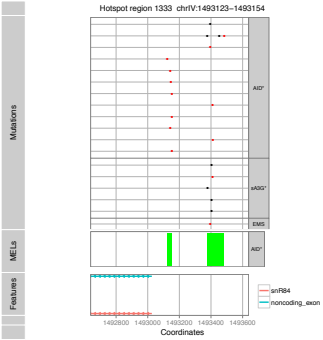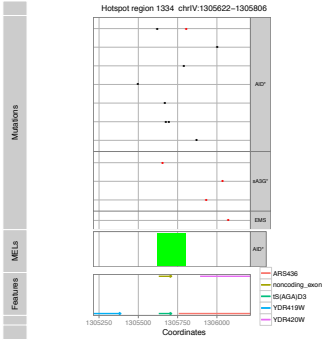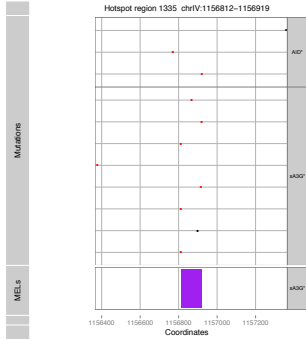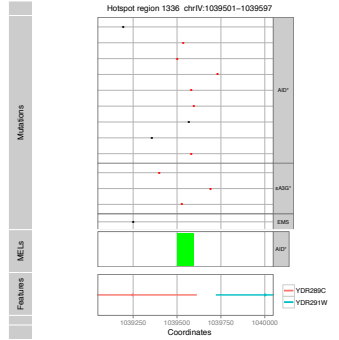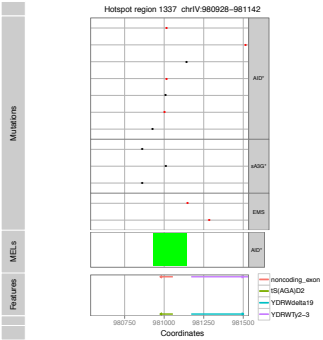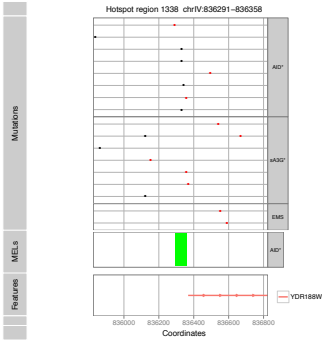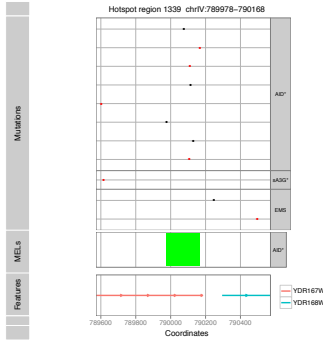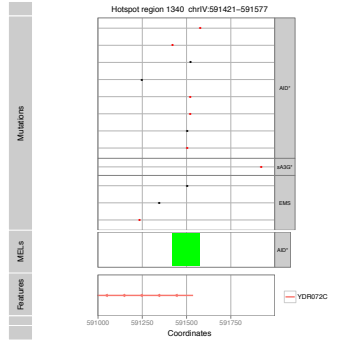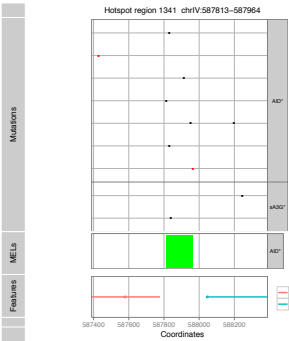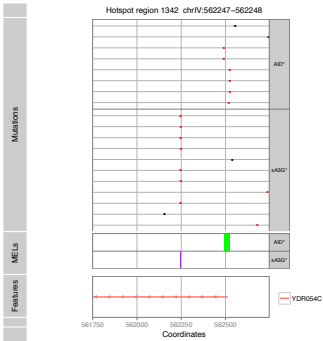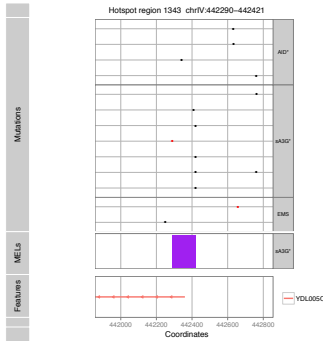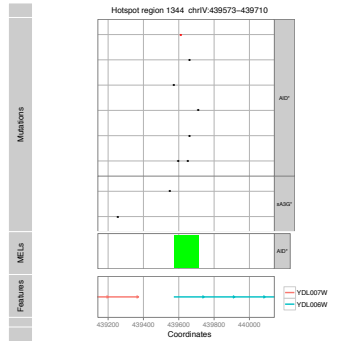

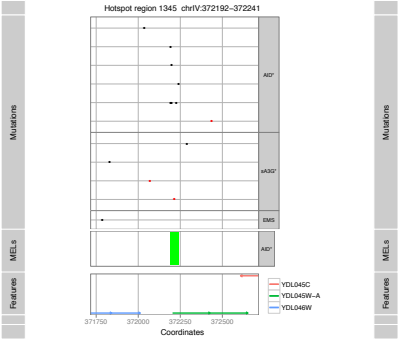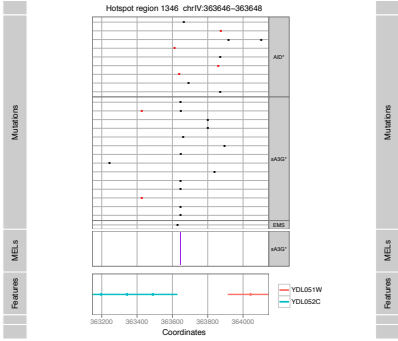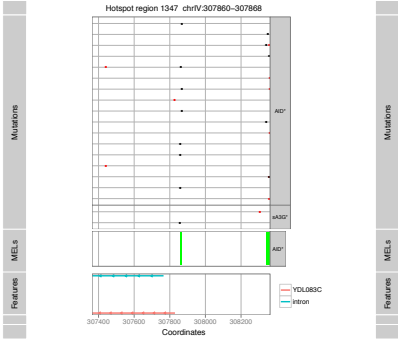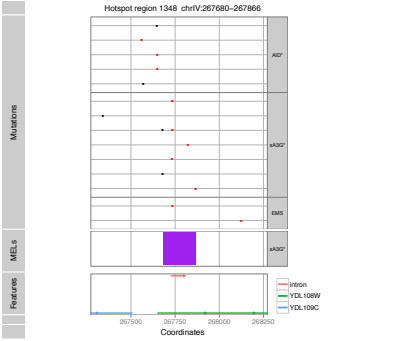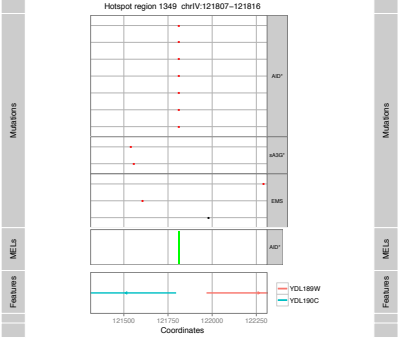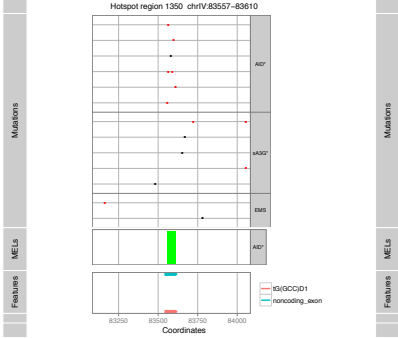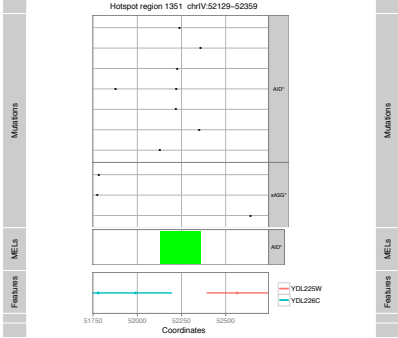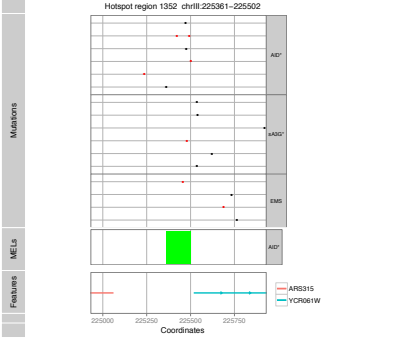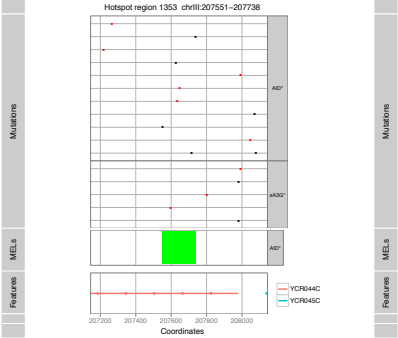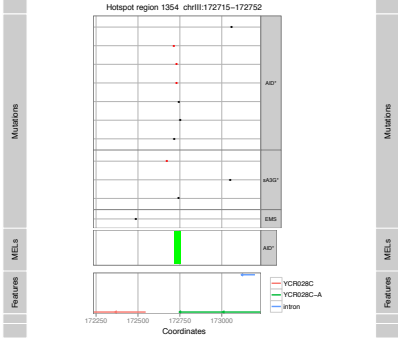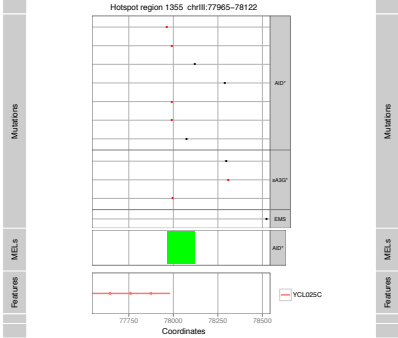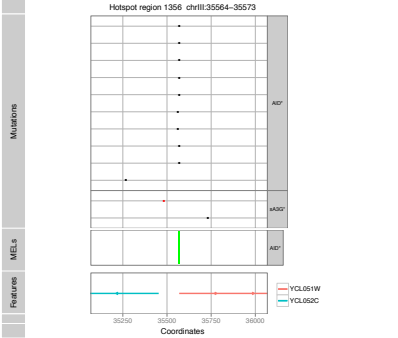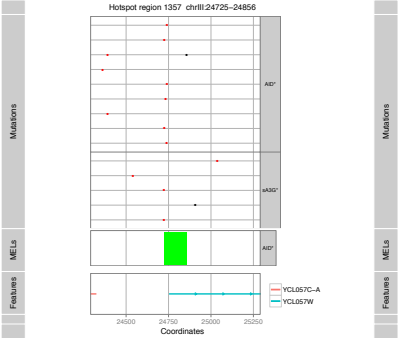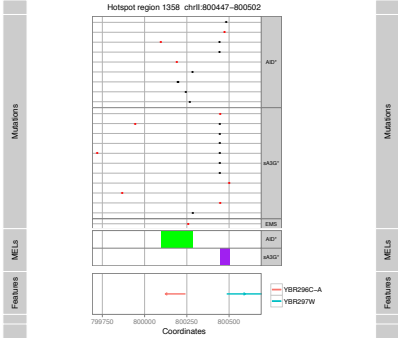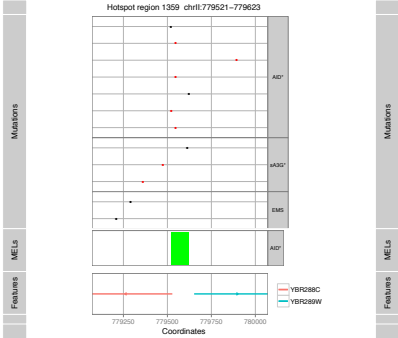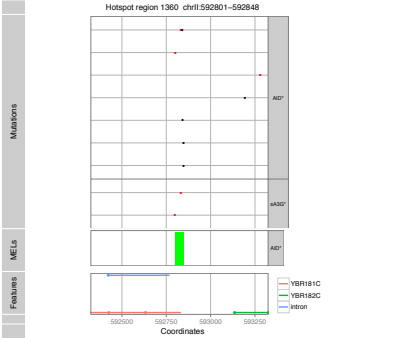

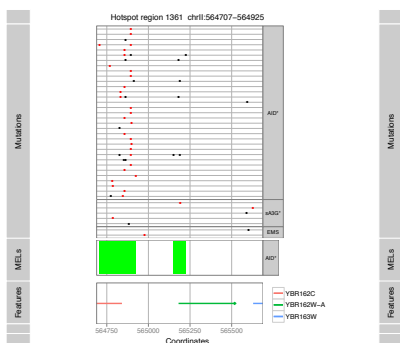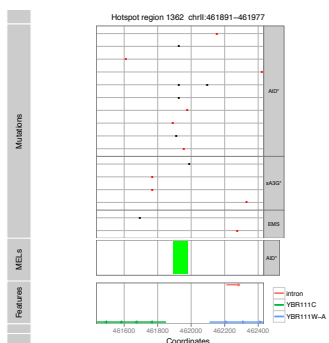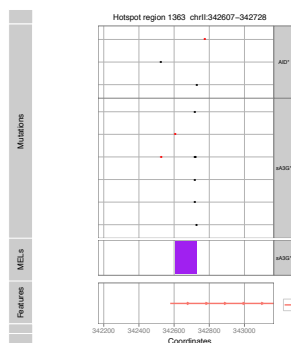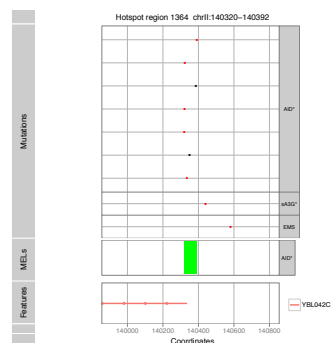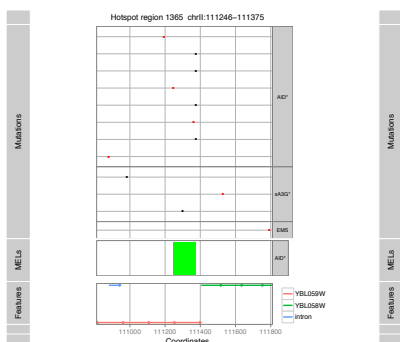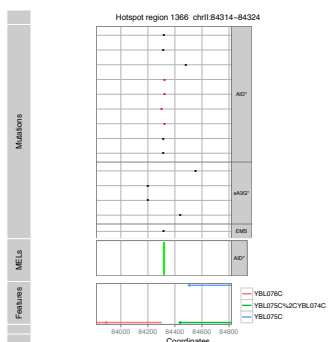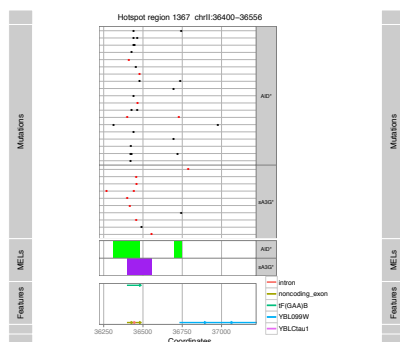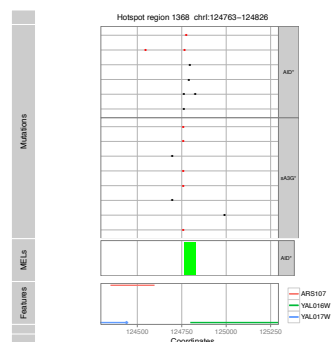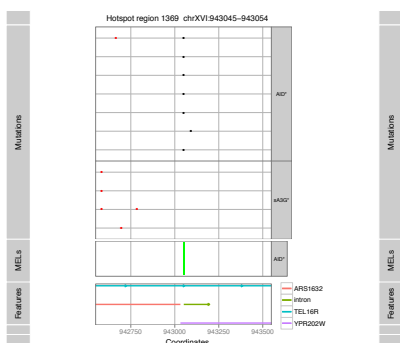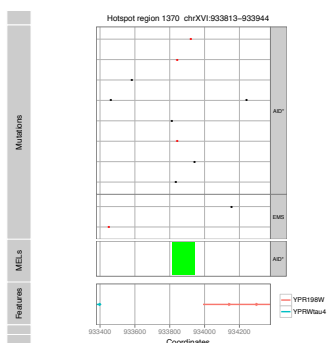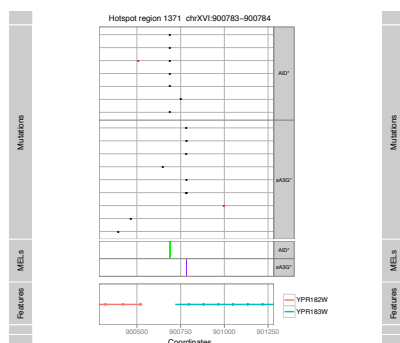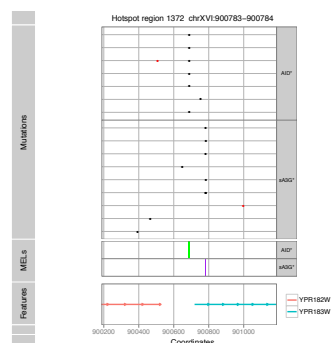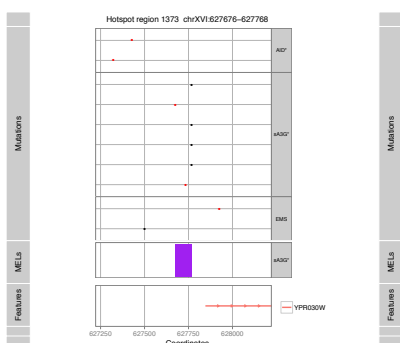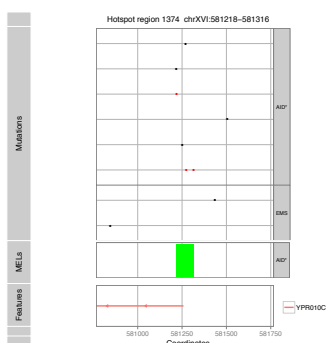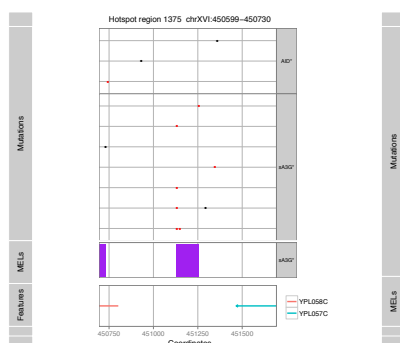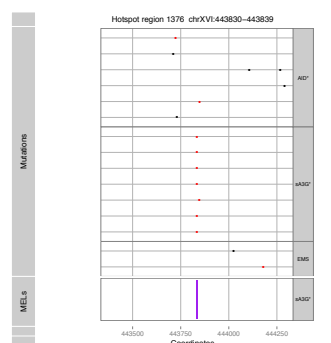

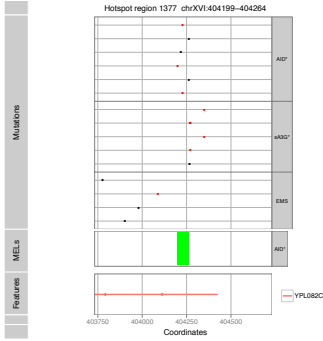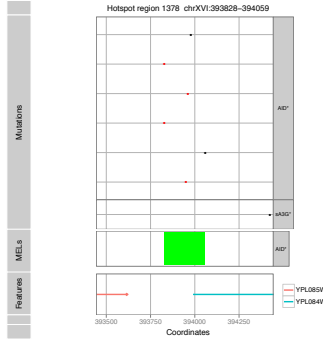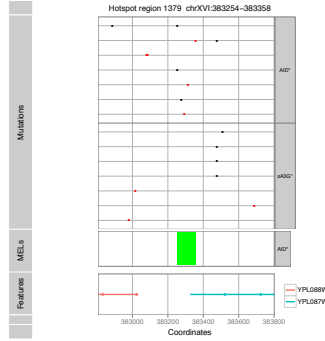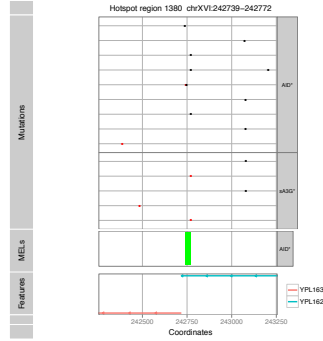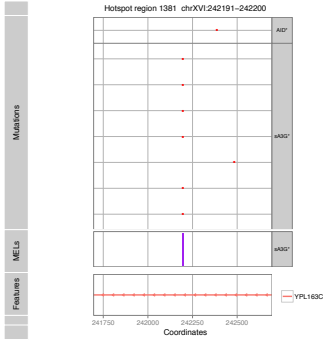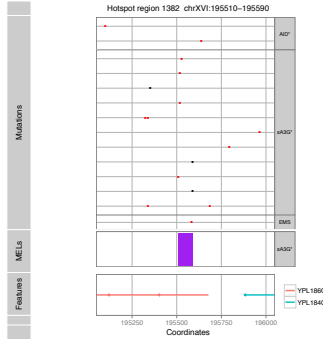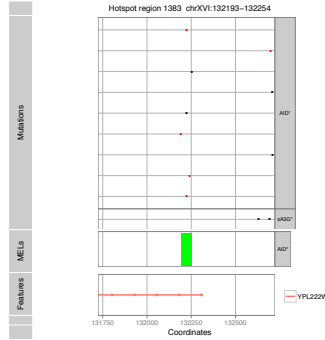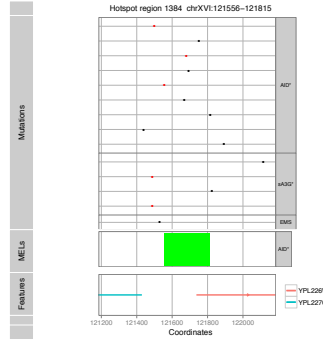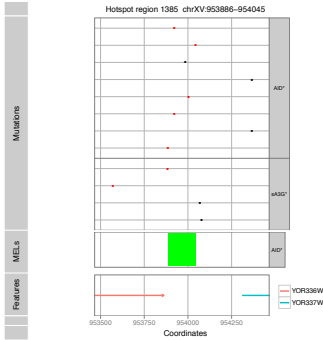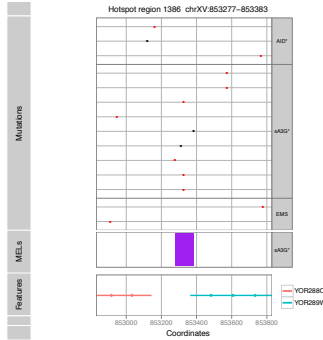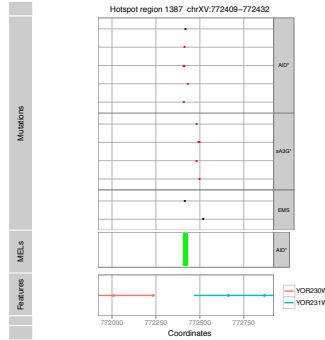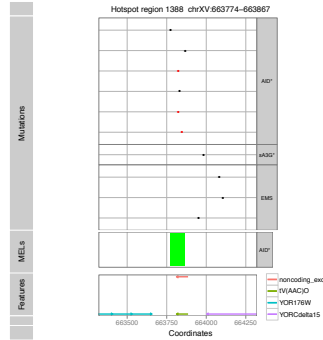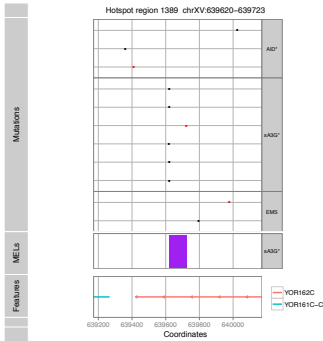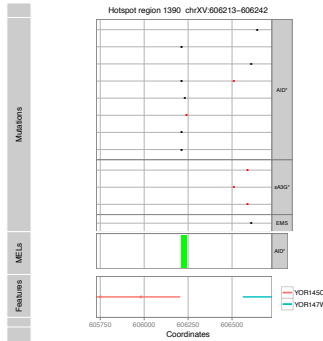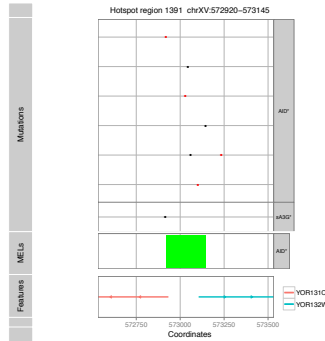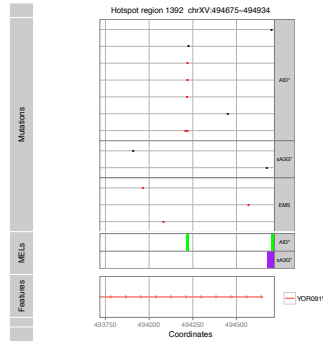

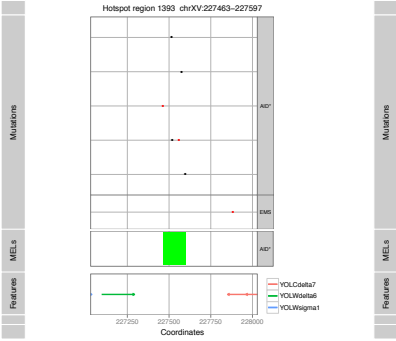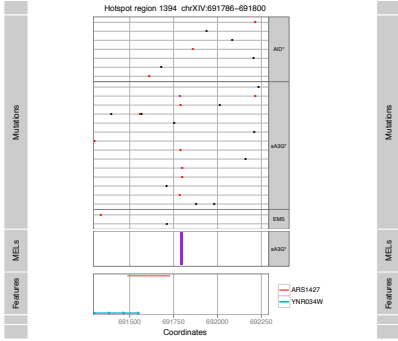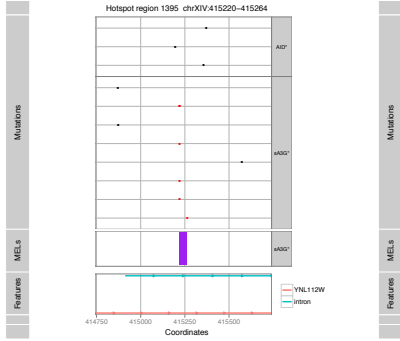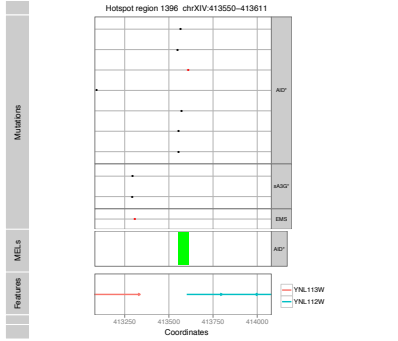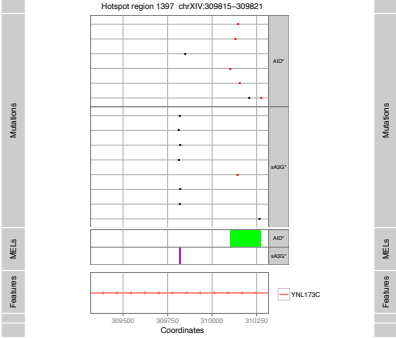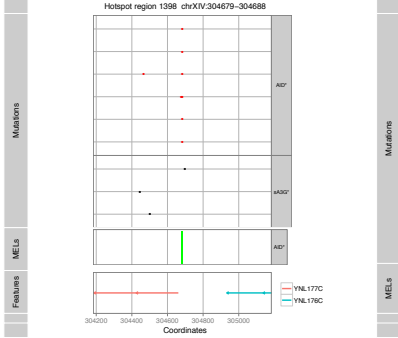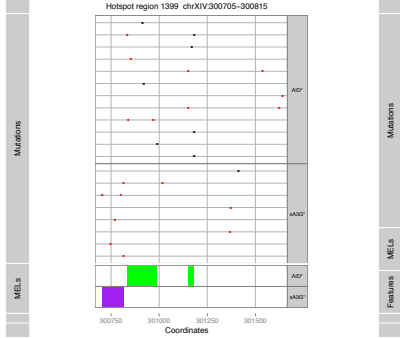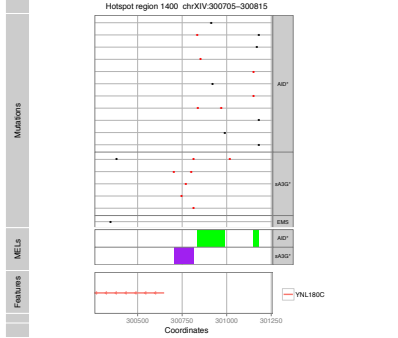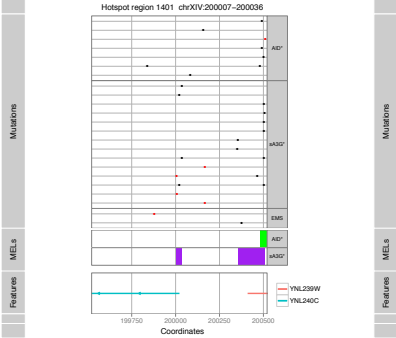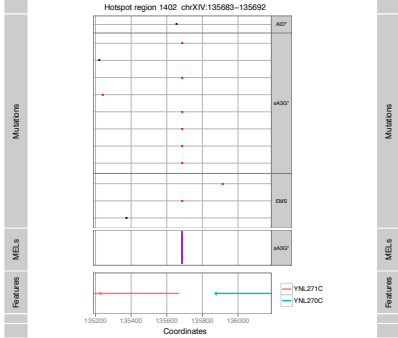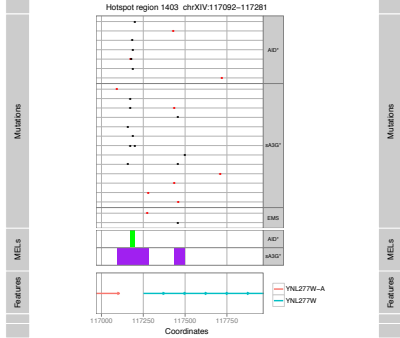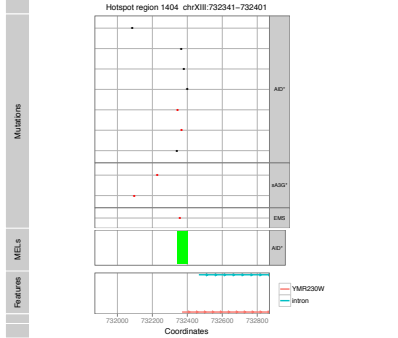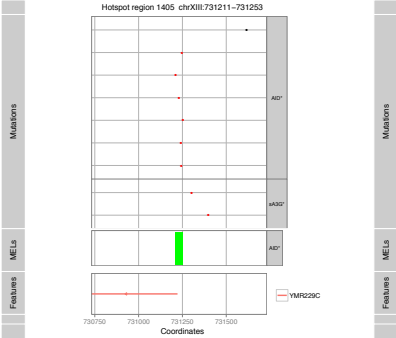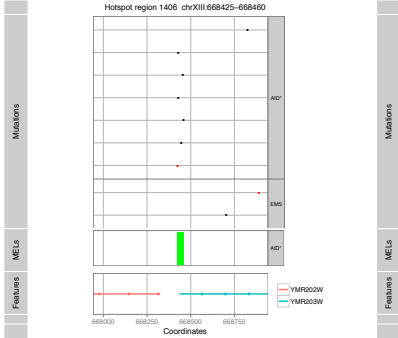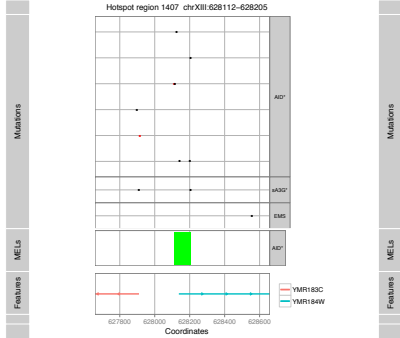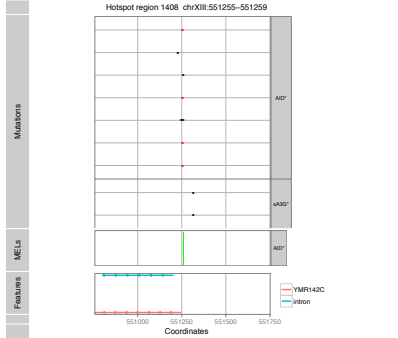

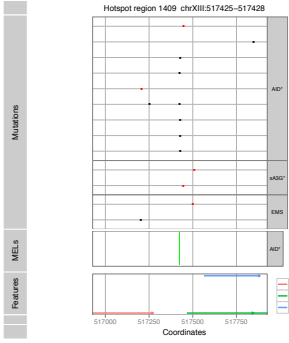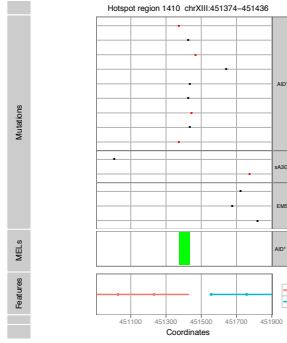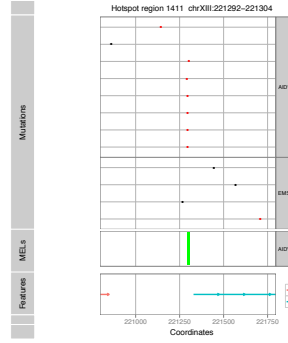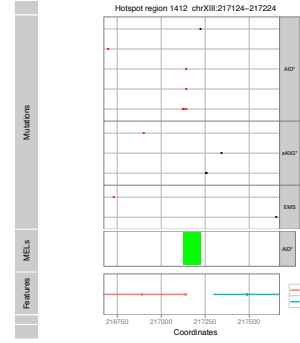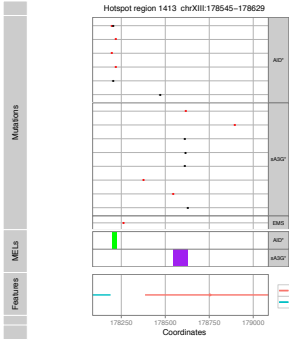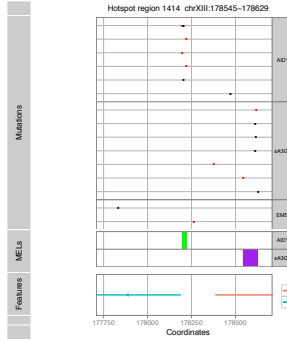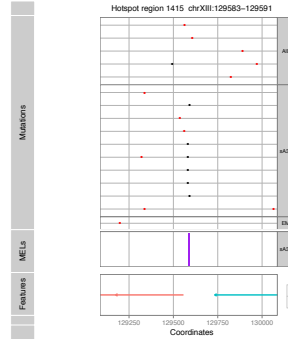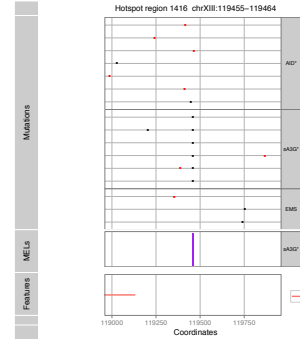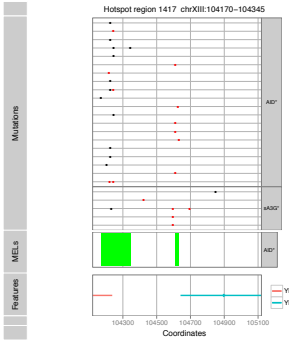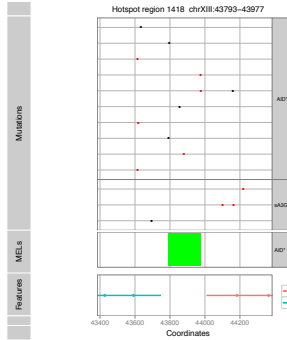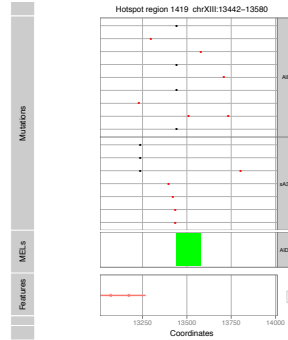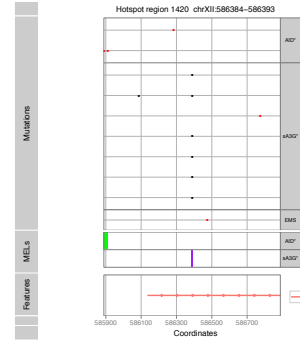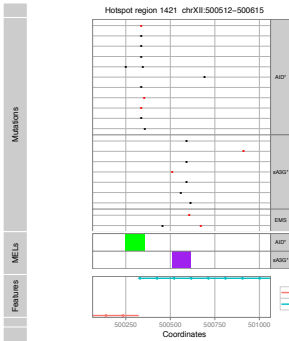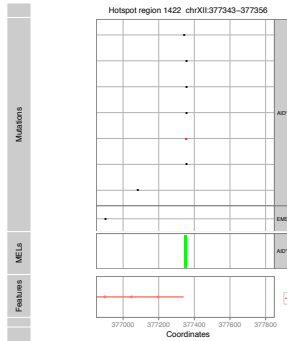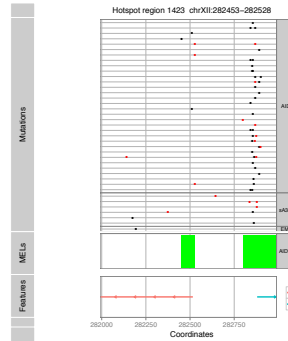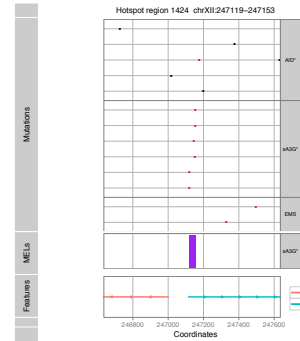

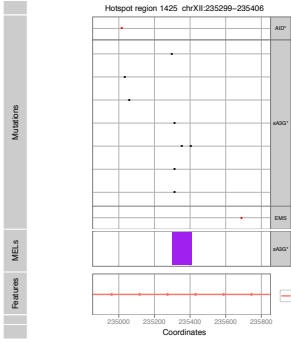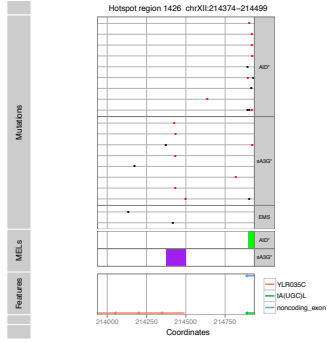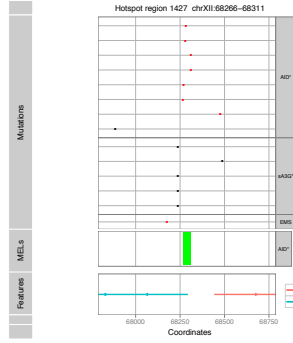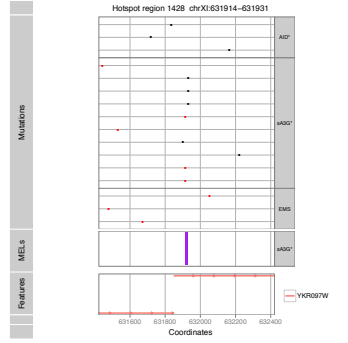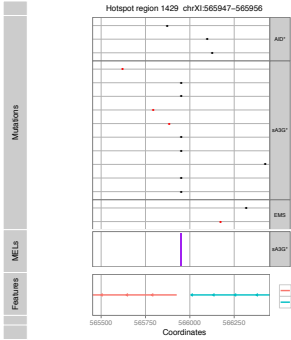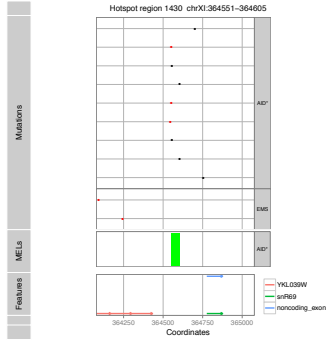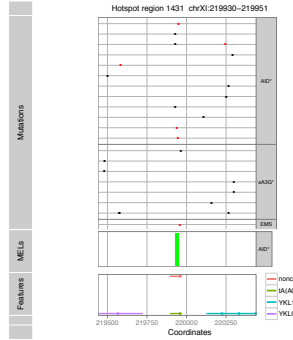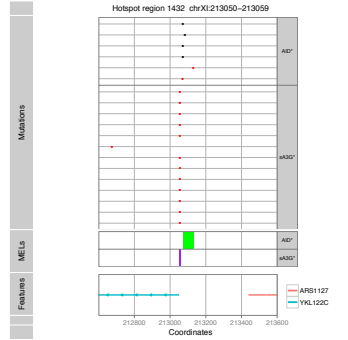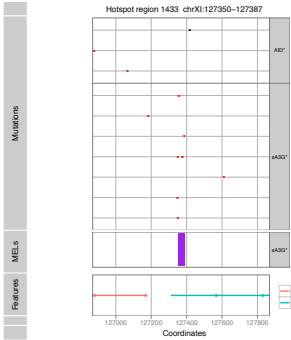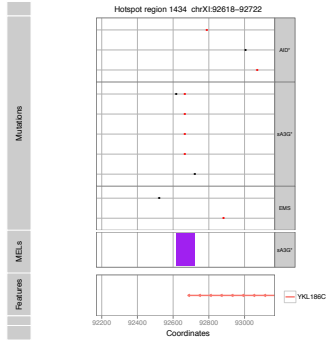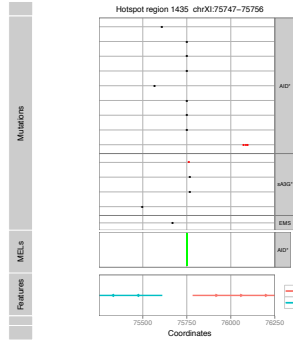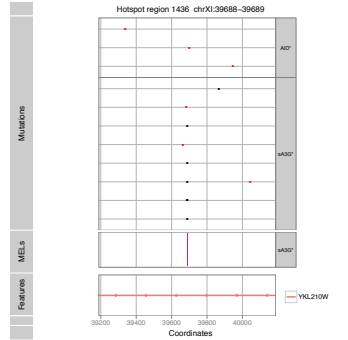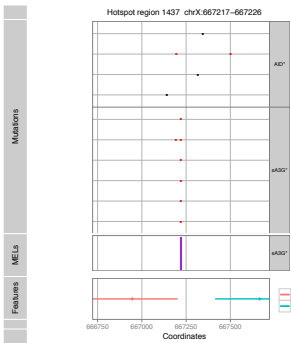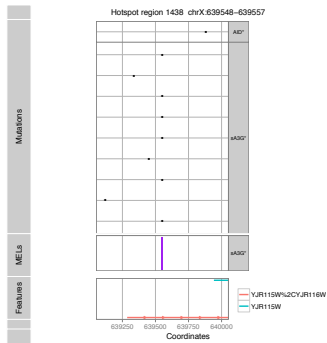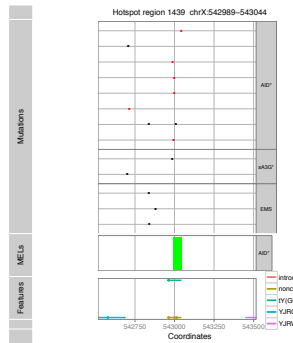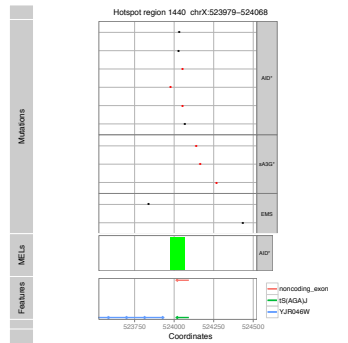

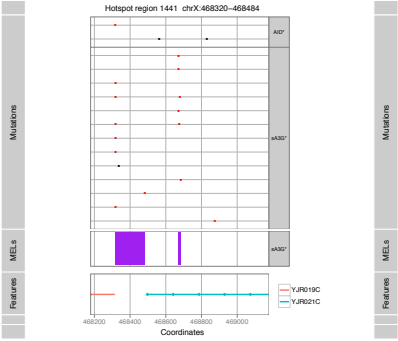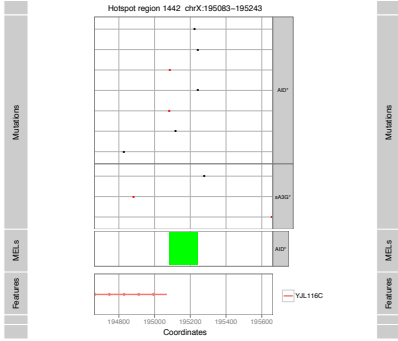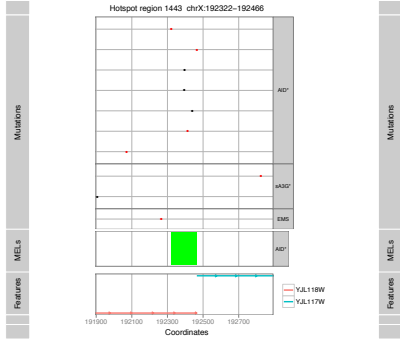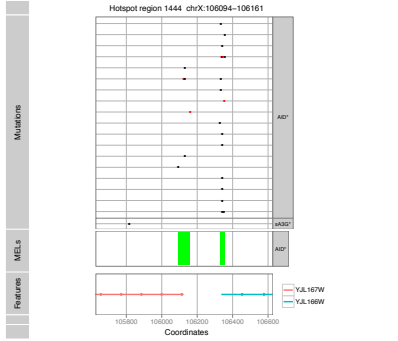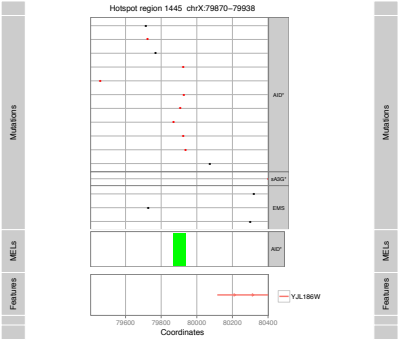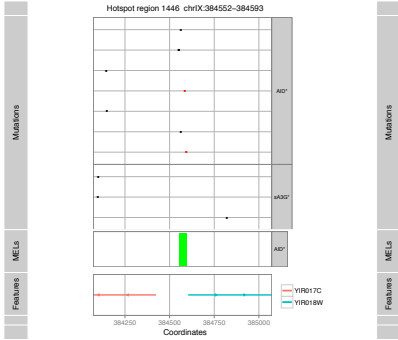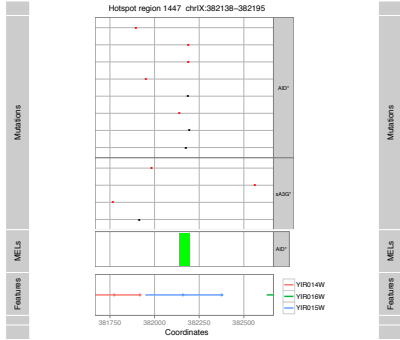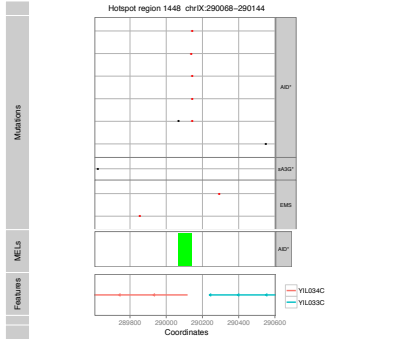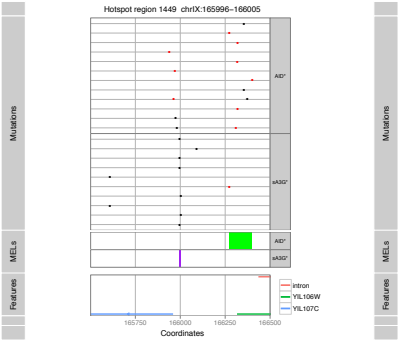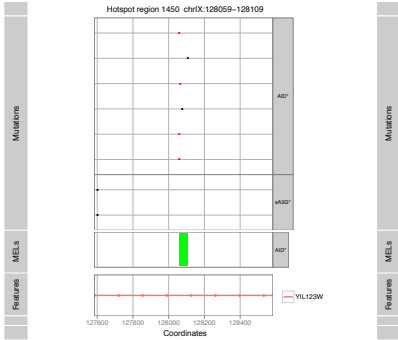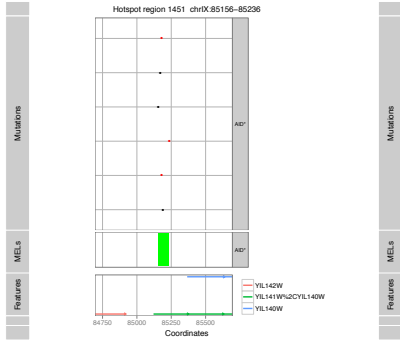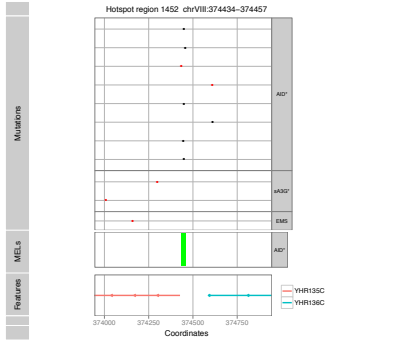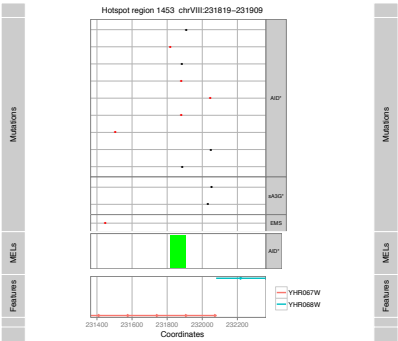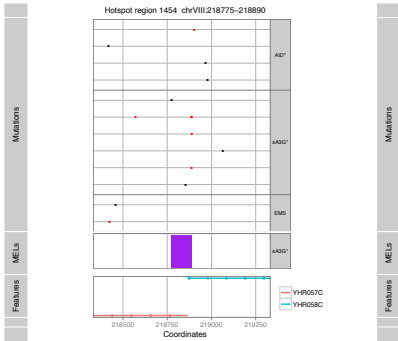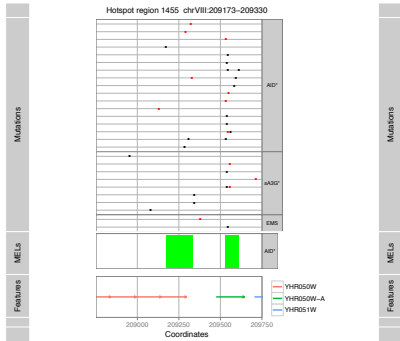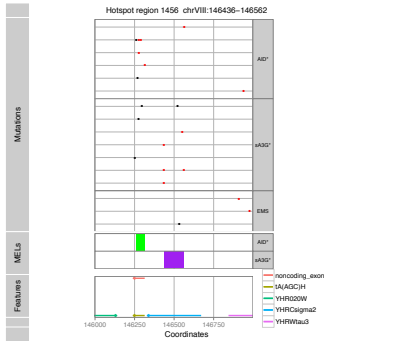

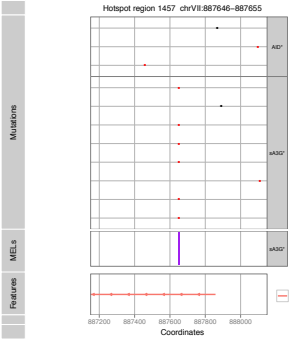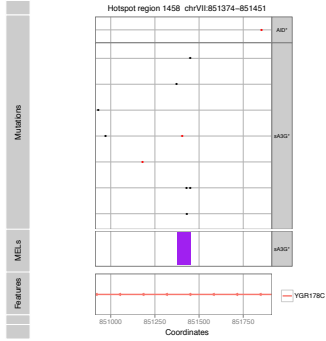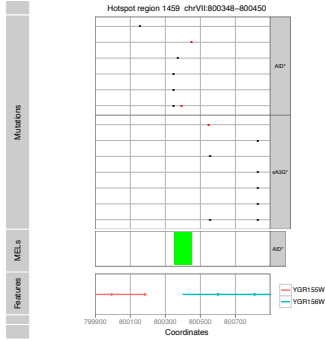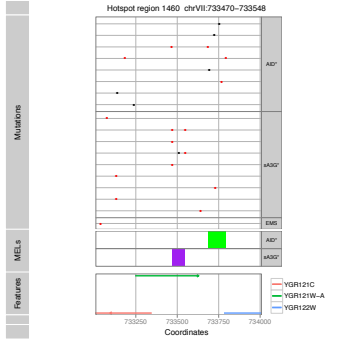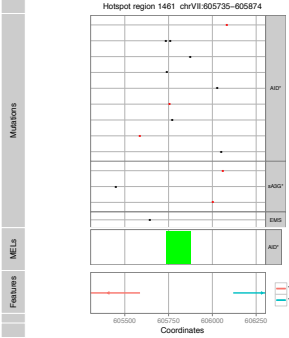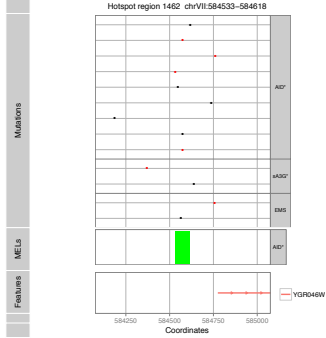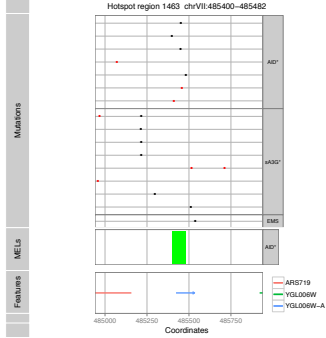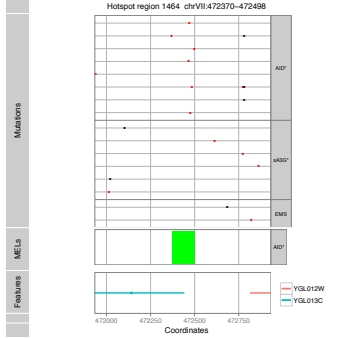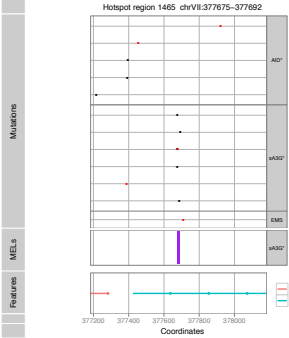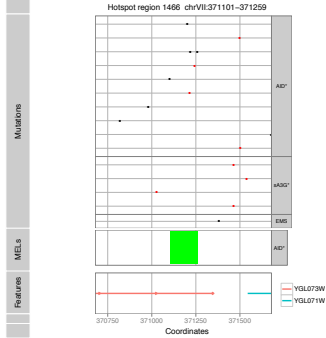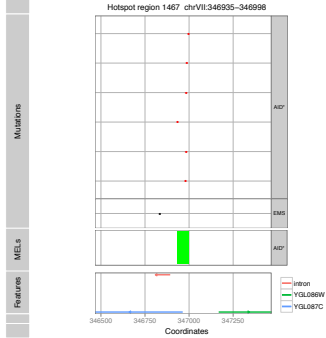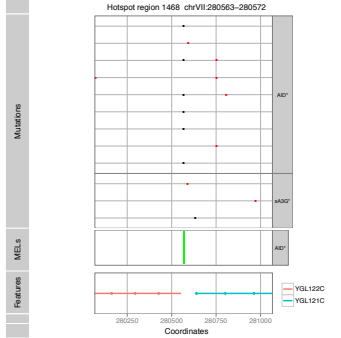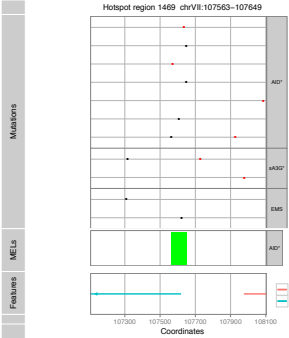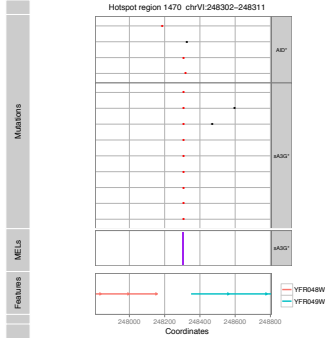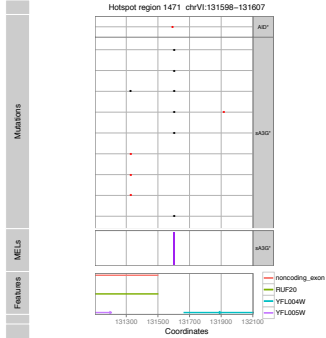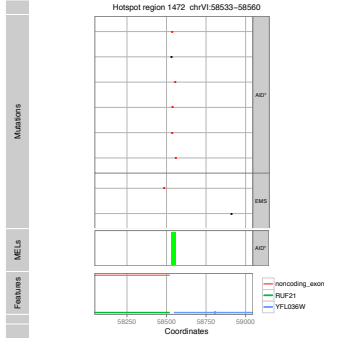

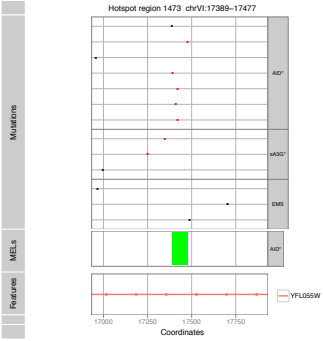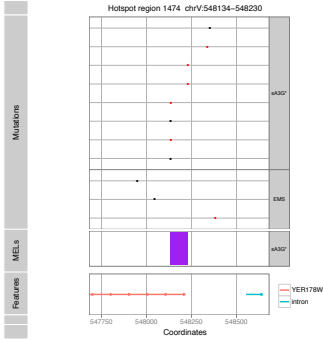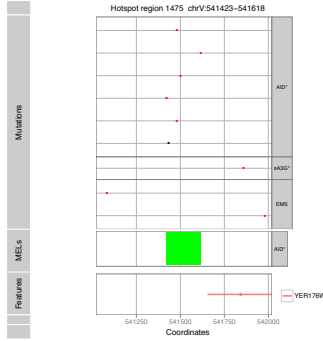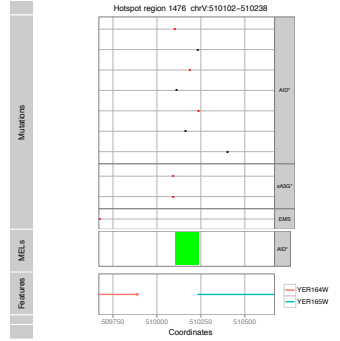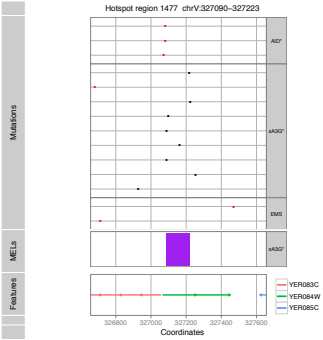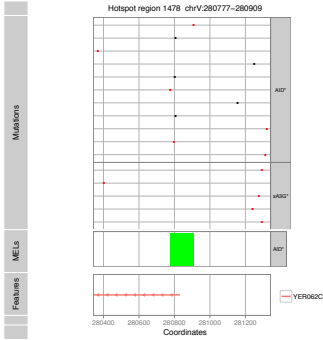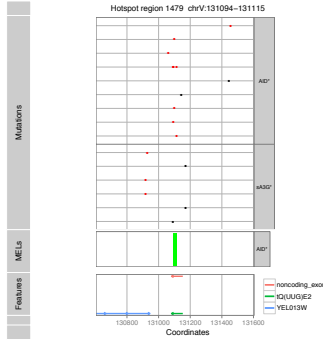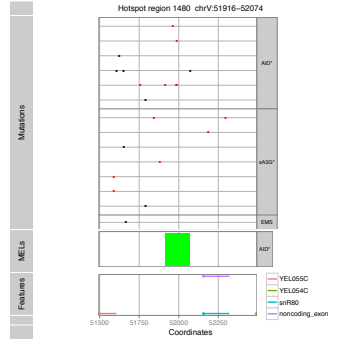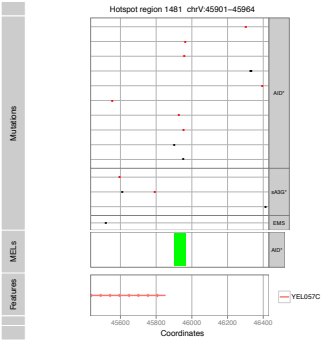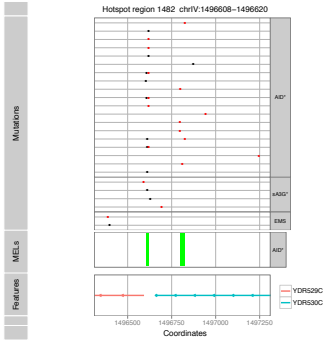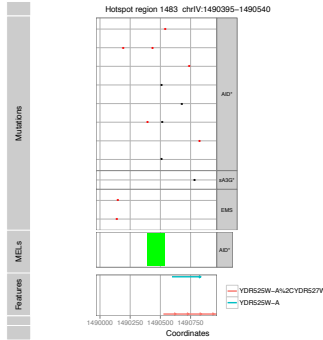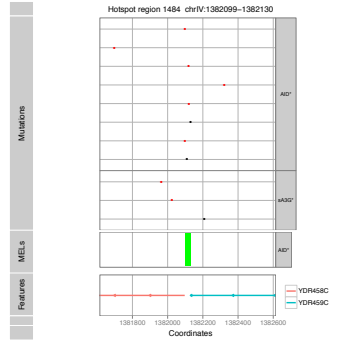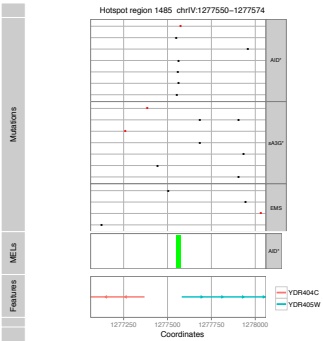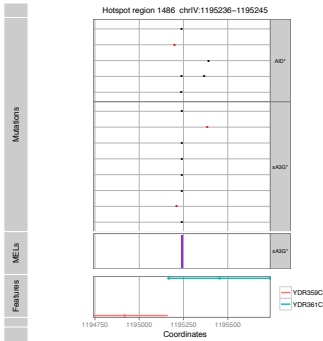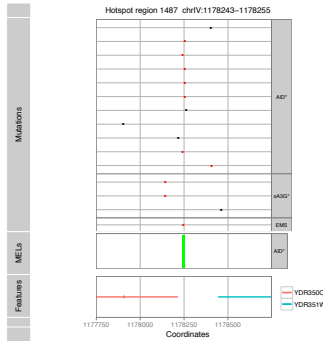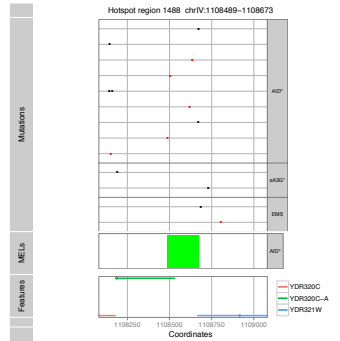

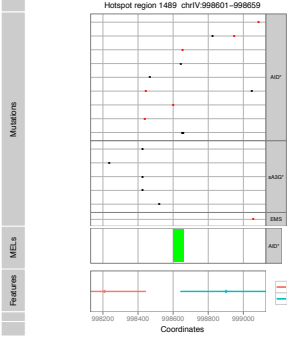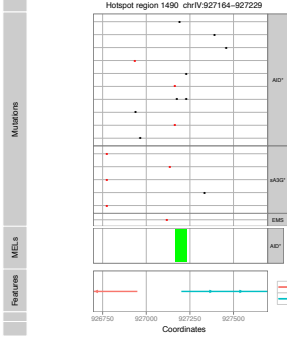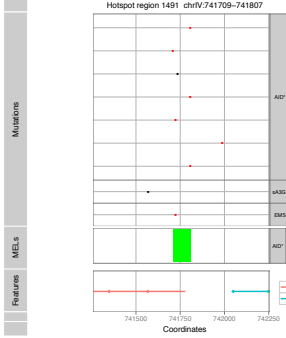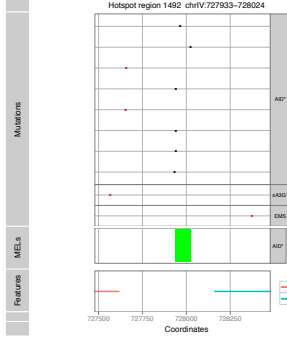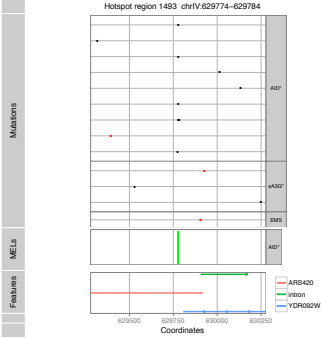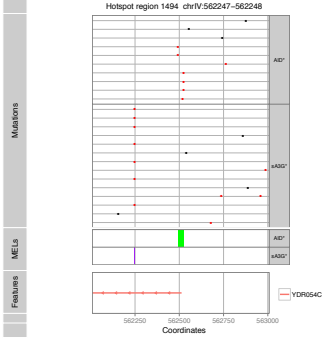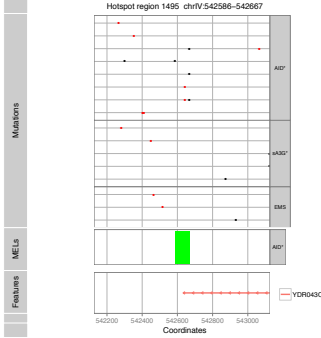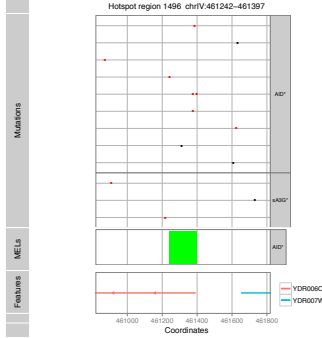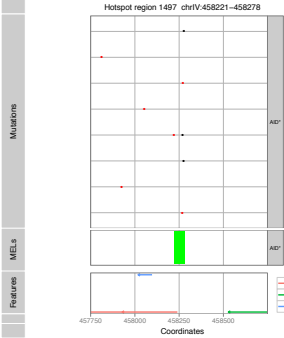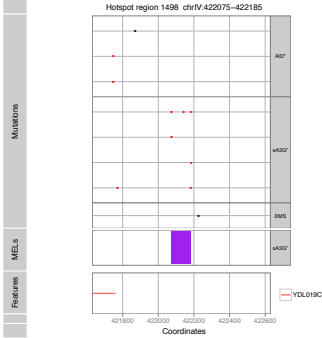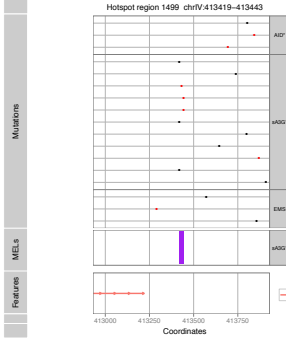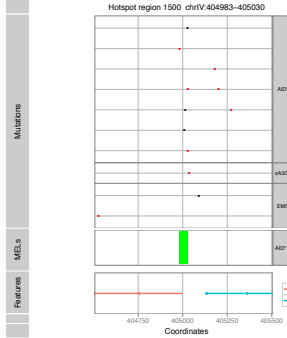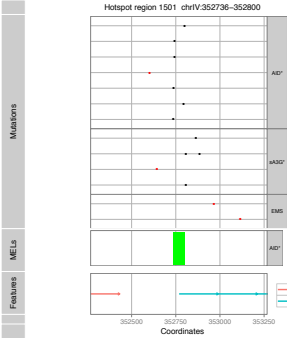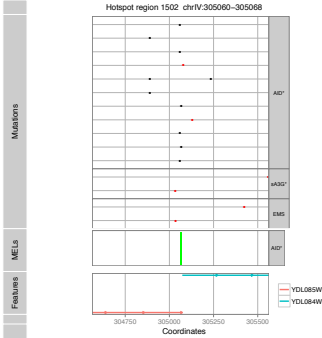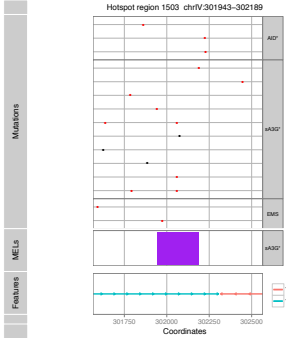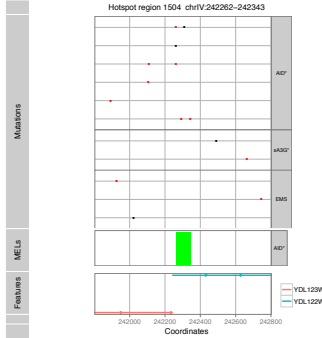

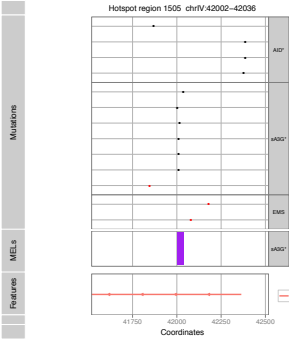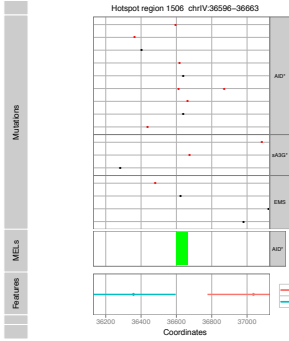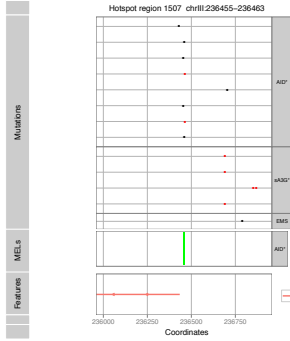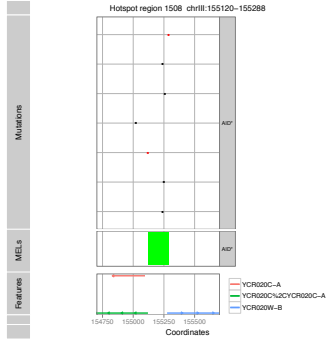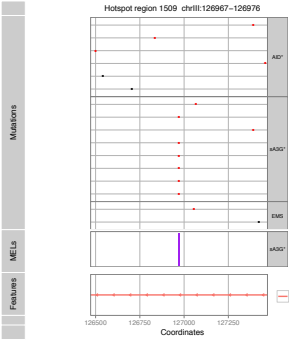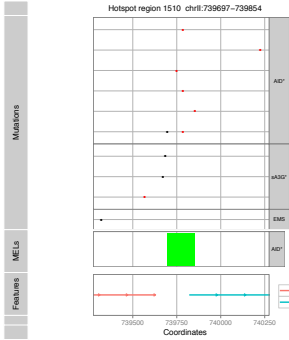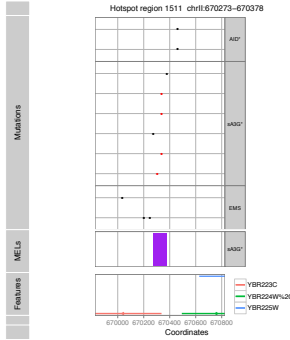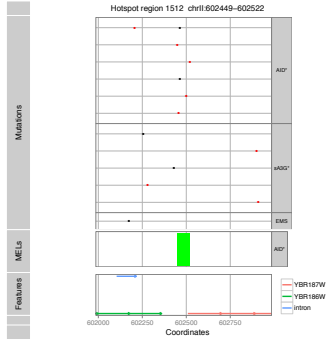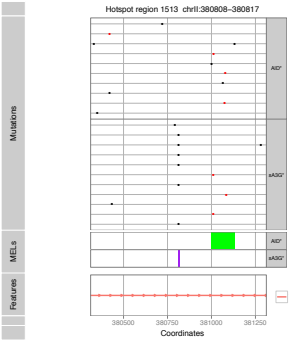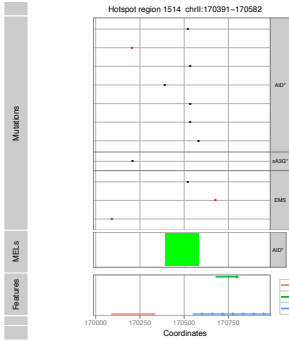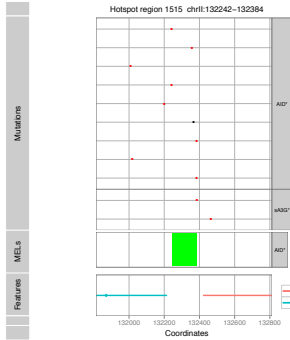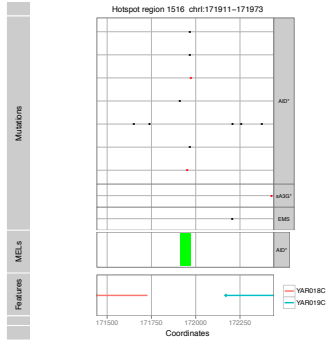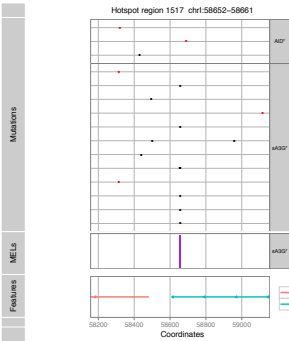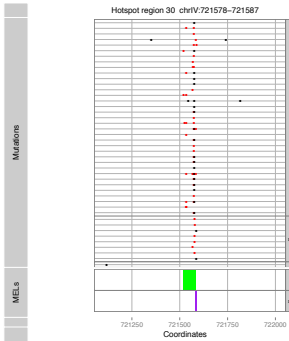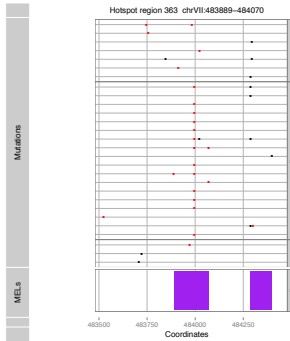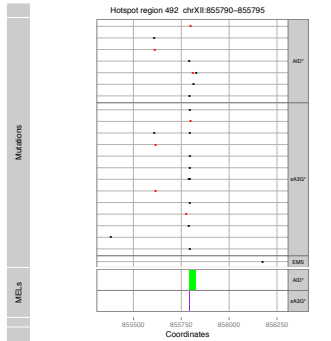

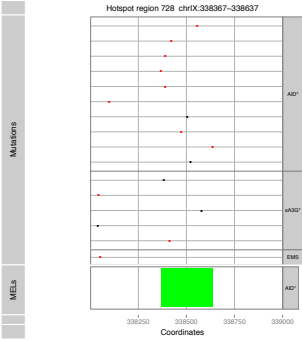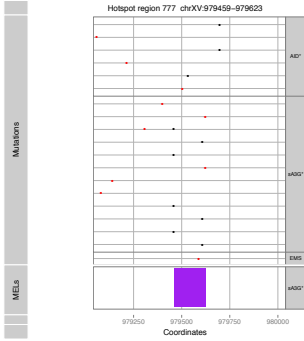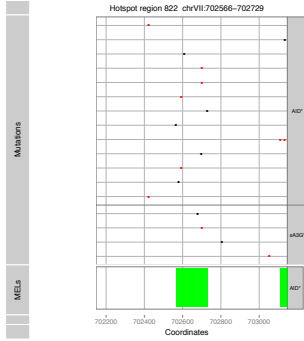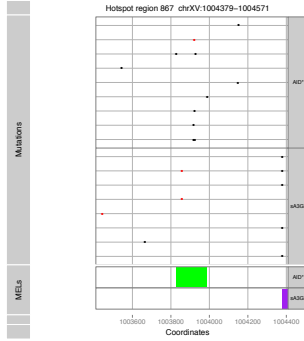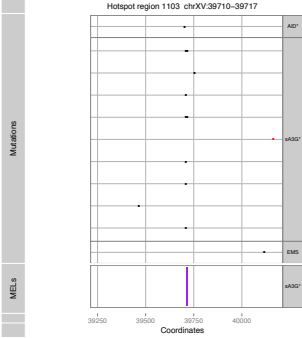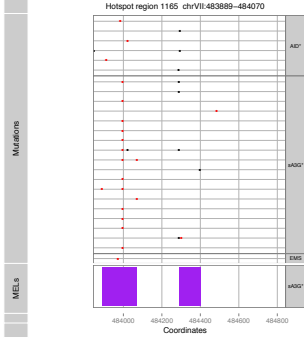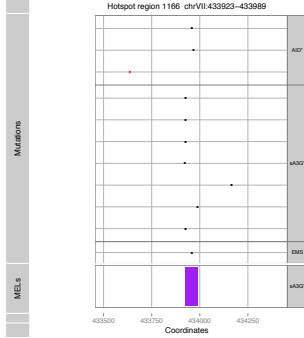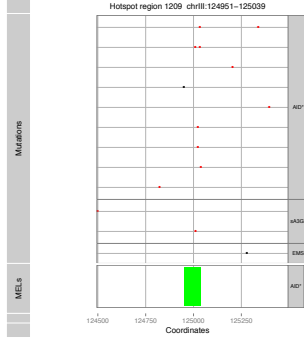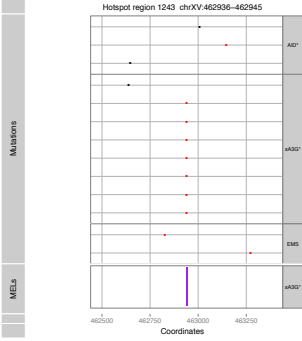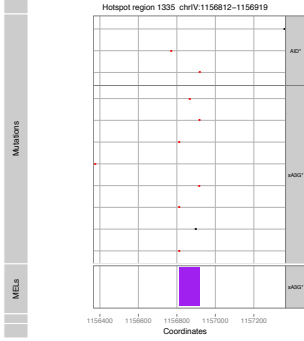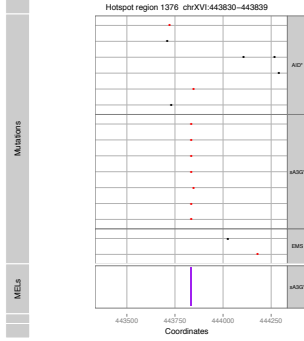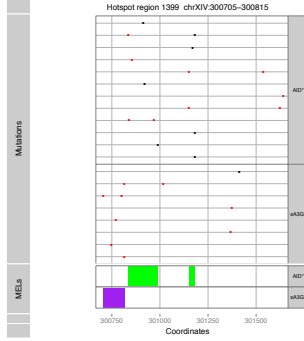

Supplement: Supplementary file 3. — All mutationally enriched regions (MELs). Top panel indicate position of each non-clonal mutation indicated by a dot (at C, black; at G, red), with horizontal lines representing a single genome. Middle panel shows MELs (AID*, green; sA3G*, purple; EMS, grey). Bottom panel displays genomic features (including transcripts, replication origins, centromers), coloured according to feature type, with arrows indicating the direction of transcription. The coordinates of the region are indicated. Regions are ranked according to the number of mutations present. DOI: http://dx.doi.org/10.7554/eLife.03553.023 [file elife03553s003.pdf]
